# Evaluation of Swin Transformer and knowledge transfer for denoising of super-resolution structured illumination microscopy data

**DOI:** 10.1093/gigascience/giad109

**Published:** 2024-01-13

**Authors:** Zafran Hussain Shah, Marcel Müller, Wolfgang Hübner, Tung-Cheng Wang, Daniel Telman, Thomas Huser, Wolfram Schenck

**Affiliations:** Faculty of Engineering and Mathematics, Bielefeld University of Applied Sciences and Arts, 33619 Bielefeld, Germany; Faculty of Physics, Bielefeld University, 33615 Bielefeld, Germany; Faculty of Physics, Bielefeld University, 33615 Bielefeld, Germany; Faculty of Physics, Bielefeld University, 33615 Bielefeld, Germany; Leica Microsystems CMS GmbH, 68165 Mannheim, Germany; Faculty of Engineering and Mathematics, Bielefeld University of Applied Sciences and Arts, 33619 Bielefeld, Germany; Faculty of Physics, Bielefeld University, 33615 Bielefeld, Germany; Faculty of Engineering and Mathematics, Bielefeld University of Applied Sciences and Arts, 33619 Bielefeld, Germany

**Keywords:** Structured illumination microscopy, Fluorescence microscopy, Deep learning, Transformer, Swin Transformer, SwinIR, Convolutional neural networks, Denoising, Image restoration, Transfer learning, Fine-tuning

## Abstract

**Background:**

Convolutional neural network (CNN)–based methods have shown excellent performance in denoising and reconstruction of super-resolved structured illumination microscopy (SR-SIM) data. Therefore, CNN-based architectures have been the focus of existing studies. However, Swin Transformer, an alternative and recently proposed deep learning–based image restoration architecture, has not been fully investigated for denoising SR-SIM images. Furthermore, it has not been fully explored how well transfer learning strategies work for denoising SR-SIM images with different noise characteristics and recorded cell structures for these different types of deep learning–based methods. Currently, the scarcity of publicly available SR-SIM datasets limits the exploration of the performance and generalization capabilities of deep learning methods.

**Results:**

In this work, we present SwinT-fairSIM, a novel method based on the Swin Transformer for restoring SR-SIM images with a low signal-to-noise ratio. The experimental results show that SwinT-fairSIM outperforms previous CNN-based denoising methods. Furthermore, as a second contribution, two types of transfer learning—namely, direct transfer and fine-tuning—were benchmarked in combination with SwinT-fairSIM and CNN-based methods for denoising SR-SIM data. Direct transfer did not prove to be a viable strategy, but fine-tuning produced results comparable to conventional training from scratch while saving computational time and potentially reducing the amount of training data required. As a third contribution, we publish four datasets of raw SIM images and already reconstructed SR-SIM images. These datasets cover two different types of cell structures, tubulin filaments and vesicle structures. Different noise levels are available for the tubulin filaments.

**Conclusion:**

The SwinT-fairSIM method is well suited for denoising SR-SIM images. By fine-tuning, already trained models can be easily adapted to different noise characteristics and cell structures. Furthermore, the provided datasets are structured in a way that the research community can readily use them for research on denoising, super-resolution, and transfer learning strategies.

## Introduction

In optical microscopy, super-resolution structured illumination microscopy (SR-SIM) plays a significant role in the field of biological and biomedical studies to analyze living cells and biological specimens with characteristic features below the resolution limit of classical microscopes (approx. 250 nm for high-end systems with oil-immersion objective lenses). Structures of interest include, for example, the internal organelles of mitochondria, cellular cytoskeleton, virus particles, or small vesicles [1–5]. SR-SIM is an important super-resolution approach to disentangle complex biological cellular structures with an up to 2-fold enhancement of spatial resolution beyond the diffraction limit. During the process of super-resolution imaging, SR-SIM involves the illumination of the biological sample with spatially patterned light following a sinusoidal intensity distribution. A series of raw images with typically 3 or 5 different phase positions and 3 angles of orientation of the illumination pattern are normally acquired [6]. Subsequently, frequency domain–based image reconstruction algorithms (e.g., implemented in software packages such as fairSIM [7], OpenSIM [8], and Python-based packages [9]) are applied to a set of raw SIM images to generate the final 2-fold super-resolved images. SR-SIM has many advantages over other super-resolution methods; for example, it does not require special sample preparation, it permits the use of conventional fluorophores in multiple colors, and simultaneously, it allows imaging at high speed for large fields-of-view (FOVs) while being compatible with live-cell samples by making efficient use of low-illumination intensity levels [10, 11]. The conventional SR-SIM reconstruction algorithms have some limitations whenever the signal-to-noise level of the raw images is poor due to low fluorescence emission or short exposure times [12].

In general, in fluorescence microscopy, weak emission due to, for example, low labeling densities, high light scattering or absorption, or optical aberrations is often encountered [13, 14]. In the case of several super-resolution fluorescence microscopy methodologies, this leads to a low signal-to-noise ratio (SNR) and poor image reconstructions or artifacts. Nevertheless, SR-SIM imaging provides better resolution and optical sectioning abilities than confocal microscopy with a just minimally larger number of raw images to be acquired [15].

In the past decade, deep learning methods have become widely accepted in image processing. In addition, they are being used with increasing success for the restoration of SR-SIM images (e.g., for denoising) [16–20]. Qiao et al. [21] proposed a generative adversarial network (GAN)–based deep Fourier channel attention network method to reconstruct SR-SIM images under low SNR conditions. Xypakis et al. [22] introduced a custom convolutional neural network (CNN) architecture for blind-SIM: BS-CNN. Liu et al. [23] proposed a dual-domain learning strategy for the reconstruction of SIM images. Authors in [24] suggested another GAN-based channel attention generative adversarial network to improve the quality of 3D-SIM reconstruction using fewer raw samples of low SNR.

Transformers have recently shown great success in various natural language-processing tasks [25–29]. Since then, as an alternative to CNNs, Transformer-based architectures have also been adapted to computer vision tasks as well, such as classification [30], detection  [31, 32], and image restoration [33]. Vision Transformers for the image restoration typically divide each image into fixed-size patches and process each patch independently to limit computational complexity [34], resulting in the introduction of border artifacts around each patch in the restored image. The Swin Transformer overcomes this shortcoming by integrating a shifted window–based multihead self-attention (MSA) in the Transformer architecture [35]. Swin serves as the basis for the Shifted window Image Restoration (SwinIR) method [36], which has been proposed especially for various image restoration tasks. Although these latest Transformers outperform CNN-based methods in conventional image restoration to some extent, they were never explored for the restoration of high-resolution SR-SIM microscopy images. Therefore, during this work, we propose a SwinIR-based Transformer architecture named “SwinT-fairSIM” to denoise SR-SIM images under low SNR conditions.

Both CNN- and Transformer-based methods require a large number of images to train the underlying models. In the field of microscopy, the sheer size and storage requirements of datasets of high-resolution reconstructed microscopic images make them difficult to produce and typically not publicly available. The few open-source datasets available are mostly related to wide-field microscopy and contain a relatively small number of images. For example, Zhang et al. [37] collected 3 wide-field microscopy datasets purely for denoising tasks without providing high-resolution ground-truth images obtained by SR-SIM technology. They used image averaging to generate ground-truth data with high SNR images. Zhou et al. [38], on the other hand, published a dataset called “Widefield2SIM” using wide-field fluorescence microscopy. They captured 120 different FOVs with 400 low SNR images for each FOV and generated high-resolution ground-truth data using SR-SIM imaging technology. Qiao et al. [21] presented the “BioSR” dataset consisting of 2,200 pairs of low-resolution (LR) raw data and high-resolution (HR) data. According to Qiao et al., the BioSR dataset covers 4 different biological structures (CCPs, ER, MTs, F-actin), 9 signal levels (15–600 average photon count), and 2 upscaling factors (linear SIM and nonlinear SIM). Similarly, Hagen et al. [39] published a variety of datasets that were collected using wide-field and confocal microscopy. They captured various fluorescently labeled structures such as actin, mitochondria, membrane, and nuclei with low and high SNR. However, their collection of datasets consists of significantly fewer images than the BioSR data collection.

Here, we present a series of datasets that are related to SR-SIM microscopy for image restoration tasks. These datasets cover 2 types of biological structures, tubulin filaments and vesicles, with multiple FOVs for the denoising, super-resolution, and joint denoising and super-resolution tasks. In our datasets, noisy input and reference output images were obtained by using SR-SIM reconstruction algorithms. We believe that our datasets will be helpful to the research community in benchmarking different deep learning–based denoising and super-resolution (SR) methods, not least because of the rather large number of provided samples.

Finally, we show how these datasets can be used to demonstrate the generalization capabilities of image restoration algorithms in the field of SIM microscopy. To this end, we apply the concept of transfer learning to 3 algorithms for the denoising of SR-SIM data. Two of these algorithms are based on CNNs and were proposed by us in [16]. They are called “Red-fairSIM” and “UNet-fairSIM.” The third algorithm is the abovementioned Transformer architecture “SwinT-fairSIM,” which is first proposed in this article. With respect to transfer learning, we compare 2 methods from this area: direct transfer and fine-tuning. In direct transfer, a model pretrained on one type of data is used for inference on a related but different type of data. In fine-tuning, part of the pretrained model is retrained on the new type of data before inference. In previous work, we were already able to show that some CNN-based algorithms for the denoising of SR-SIM images are robust to different noise levels and SIM modes (e.g., varying pattern spacings at different illumination wavelengths) [19]. However, due to the limited amount of SR-SIM data available at that time, we were not able to explore the techniques of transfer learning in more depth. Thus, here, we also aim to answer the following questions related to direct transfer and fine-tuning: (i) If a model is trained on a specific biological structure with a specific type of noise, will it also generalize well to denoising another, different structure with another type of noise? And (ii): Is fine-tuning of a pretrained model (i.e., previously trained on one type of structure and noise) more effective than training the model from scratch? To answer these questions, we have conducted a series of experiments that are discussed in later sections.

In particular, the contribution of this work is 3-fold: first, we present high-resolution SR-SIM datasets for image denoising and super-resolution tasks. Second, we propose a method based on the SwinIR architecture for denoising SR-SIM images. Third, we evaluate the potential of direct transfer and fine-tuning for different Transformer- and CNN-based models.

## Materials and Methods

### SR-SIM microscopy and sample preparation

The raw SIM images for all the datasets were acquired using a DeltaVision OMX V4 (GE Healthcare) 3D-SIM imaging system. The DeltaVision OMX V4 imaging system is an implementation of 3-beam 3D-SIM [15], providing both lateral and axial modulation of the excitation pattern, and thus allows to increase both the lateral and axial resolution of the imaging process (as compared to 2-beam SIM, where only the lateral resolution is increased). The system is equipped with 4 excitation laser lines (405, 488, 561, and 642 nm) and 4 sCMOS cameras to detect the emitted fluorescence light in 4 channels. The camera pixel size of 6.5 μm and an overall magnification of 82× yield a fixed effective pixel size of 80 nm. The magnification cannot be changed by the user and has been set by the manufacturer to fulfill the Nyquist criterion, thus not losing spatial information in the sampling process. While fulfilling the Nyquist sampling criterion only sets a maximum pixel size, pixel sizes much smaller than the limit are not used, as this would compromise both SNR (each pixel contributing a constant amount of read noise) and field of view (as the overall feasible pixel count is finite). To prepare the raw SIM image data (datasets 1–3) of the tubulin cytoskeleton, U2OS cells were cultured in Dulbecco’s modified Eagle medium supplemented with $10\%$ fetal bovine serum and grown on round coverslips of 170 ± 5 μm thickness (No. 1.5H). Cells were fixed with $4\%$ Paraformaldehyde (PFA) for 15 minutes, followed by phosphate-buffered saline (PBS) washes, and permeabilization with $0.5\%$ Triton-X100 for 3 minutes. Another 2 rounds of PBS washes were done before blocking with $3\%$ bovine serum albumin. For immunolabeling of the tubulin microfilaments, cells were stained with antitubulin antibody (Invitrogen, cat. 322500) 1:400 for 2 hours at room temperature, followed by a PBS wash and 1 additional hour of incubation with Alexa 488–conjugated anti-mouse IgG 1:400. Afterward, the cells were then briefly washed with PBS before Vectashield was applied to embed the coverslip onto a standard microscopy glass slide for imaging. For the preparation of raw SIM images with vesicle structures (dataset 4), U2OS cells were transfected with Lipofectamine 3000 according to the manufacturer’s protocol (Thermofisher, cat. L3000-001) together with a plasmid expressing the vesicular Lamp1 protein fused to the fluorescent protein mScarlet. After 24-hour transfection, the cells were fixed with $4\%$ PFA for 10 minutes, followed by PBS washes and Vectashield mounting prior to imaging. The vesicular structures represent lysosomes. During the data collection, the illumination intensity as well as the camera exposure time remained fixed. As the cameras are also actively cooled, thermal- and read-noise components should not vary during time-lapse image acquisition. Also, as typical for modern sCMOS cameras, thermal- and read-noise components are small (in the order of single electrons per readout) compared to the Poisson noise (i.e., the stochastic distribution of detected photons generated by the quantum nature of light propagation). The dramatic drop in SNR during data acquisition is due to photobleaching and phototoxicity in the sample.

### Dataset preprocessing and image reconstruction

**Figure 1:**
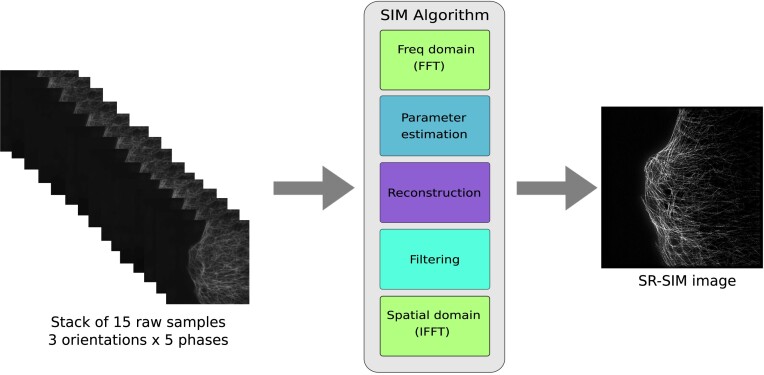
The architecture of the image reconstruction algorithms that are used to generate the reconstructed SR-SIM images in this work. Both reconstruction algorithms (i.e., as implemented in the fairSIM and softWoRx software) are based on the SIM algorithm. During the reconstruction of SR-SIM images, a stack of 15 raw SIM images of size 512 × 512 pixels is processed by the respective software, which generates the high-resolution SR-SIM image of size 1,024 × 1,024 pixels as output.

Dataset 1 is a raw dataset without any image processing or reconstruction applied to the raw SIM images. The images in datasets 2 and 3 are reconstructed by using the open-source fairSIM reconstruction algorithm as shown in Fig. 1. fairSIM implements a single-slice (2-dimensional [2D]) SR-SIM image reconstruction algorithm [7]. It works in 3 steps: parameter estimation, reconstruction, and filtering. The mathematical and algorithmic details of the fairSIM reconstruction method are explained in the original publication [7]. A synthetic optical transfer function, with NA = 1.4, $\lambda ={525}\, \mathrm{nm}, a=0.31$ (*a* is a compensation parameter; see [7, 40]) is used. For the tubulin samples, a background of 500 counts per pixel is subtracted during the reconstruction process. SR-SIM reconstruction parameters (pattern orientation, global phase, etc.) are automatically determined by fairSIM’s standard, iterative cross-correlation approach. Filter parameters are set to a generalized Wiener filter with a strength of *w* = 0.05, and apodization is set at 1.9 × the resolution limit with a *bend* of 0.8. A notch-style filter implemented as *OTF attenuation* with a strength of 0.995 and a full width at half maximum (FWHM) of ${1.2}\, \mu \mathrm{m}^{-1}$ is used. The full information about the functionality of these parameters is explained in [7], and the general guide for using SIM reconstruction parameters is discussed in [40]. The code used to generate the samples of dataset 2 is available at [41]. The samples in dataset 4 (the vLamp1-mScarlet expressing cell) were reconstructed using the commerical software softWoRx v7 (GE Healthcare manufacturer’s software) for 3D-SIM. Here, a full 3-dimensional (3D) volume of data is acquired by the microscope and passed through the reconstruction process as a 3D stack. While both single-slice reconstruction (fairSIM) and full 3D volume reconstruction offer the same increase in lateral resolution, a full 3D volume reconstruction additionally provides an axial resolution increase (when used on 3-beam data as provided by the Delta Vision OMX and other 3-beam SIM systems). This, of course, comes at the expense of requiring full 3D volumes of data to be acquired, largely increasing imaging time and phototoxicity. Thus, depending on the imaging needs, either single-slice data acquisition (and reconstruction) or full 3D volumes are chosen. For an overview of all datasets, see Table 1.

**Table 1:** Description of all the datasets

Dataset	Dataset 1	Dataset 2	Dataset 3	Dataset 4
Structure	Tubulin filaments	Tubulin filaments	Tubulin filaments	Vesicles
Microscope	SR-SIM microscopy	SR-SIM microscopy	SR-SIM microscopy	SR-SIM microscopy
Pixel size, nm	80	40	80 (input)/40 (ref.)	40
Number of timestamps	200	200	200	Max: 99, min: 15
Fields-of-view	101	101	101	175
Input image size, pixels	512 × 512	1,024 × 1,024	15 × 512 × 512	1,024 × 1,024
Output/reference image size, pixels	512 × 512	1,024 × 1,024	1,024 × 1,024	1,024 × 1,024
No. of samples	303,000	2,525	2,525	7,284
Reconstruction	Raw data	fairSIM	fairSIM	softWoRx

### Description of datasets

#### Dataset 1

**Figure 2:**
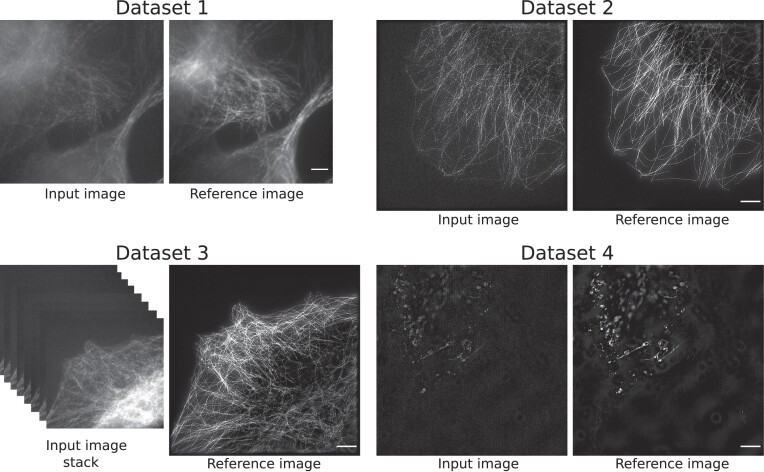
Dataset 1 consists of raw SIM data. The input–reference pairs represent each phase and orientation from different noise levels of the tubulin filaments. The size of the input and reference images are 512 × 512 pixels in dataset 1. Scale bar: $4 {\, \mu {\rm m}}$. Dataset 2 contains the reconstructed SR-SIM images of the tubulin structure, and each input–reference pair has a size of 1,024 × 1,024 pixels. Scale bar: $4 {\, \mu {\rm m}}$. In dataset 3, raw SIM data in the form of an image stack (i.e., 15 × 512 × 512 pixels) are used as input samples, while the reconstructed SR-SIM images (i.e., 1,024 × 1,024 pixels) are used as the corresponding reference samples for joint denoising and super-resolution. Scale bar of the reference image: $4 {\, \mu {\rm m}}$. Dataset 4 is based on the reconstructed SR-SIM images of size 1,024 × 1,024 pixels of fluorescently labeled vesicles. Scale bar: $4 {\, \mu {\rm m}}$.

Dataset 1 contains around 101 FOVs of tubulin filaments, and each FOV further consists of 3,000 images (all in 1 TIF file). In each FOV, a stack of 15 raw SIM images represents the combination of 5 orientations and 3 phases, whereas this full stack is repeatedly captured for 200 timestamps. The SNR decreases with every timestamp. Thus, dataset 1 contains a total of 303,000 raw SIM images of size 512 × 512 (width × height) pixels with 15 combinations of phase and orientation at each timestamp. Each timestamp in the raw images lasts approximately ${25}\, \mathrm{ms}$. Each pixel contains a single 16-bit integer value captured by the microscope’s camera, which is calibrated to provide a signal linear in photon count for each pixel. This is typical for scientific camera systems but dissimilar to standard image processing, where often gamma mapping is applied between light intensity and pixel values. This dataset can be used mainly for image denoising tasks (from noisy input to output with higher SNR). Therefore, the images from timestamp 1 are intended as output images (reference/ground truth), and the rest of the images can be categorized as input images, as shown in Fig. 2.

#### Dataset 2

**Figure 3:**
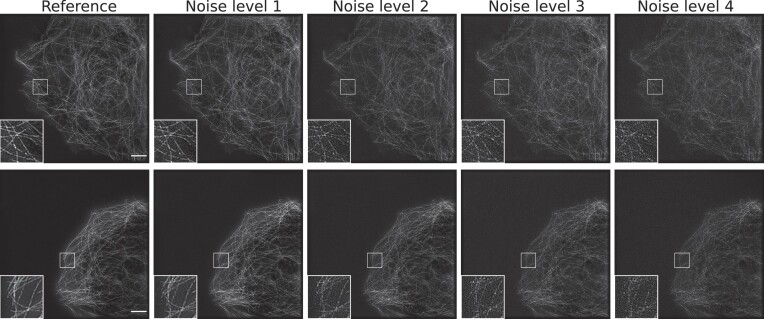
SR-SIM images from 2 different FOVs, each with different noise levels. Each noise level corresponds to a different range of timestamps (e.g., images of noise level 4 are taken at the last timestamps and contain a high level of noise). Similarly, images of noise level 1 represent early timestamps with very low noise. The reference image is recorded at timestamp 1 and has the highest signal-to-noise ratio. Scale bar: $4 {\, \mu {\rm m}}$.

We constructed dataset 2 by applying the fairSIM reconstruction algorithm to the raw SIM images from dataset 1. Dataset 2 contains therefore pairs of reconstructed high-resolution SR-SIM images, each pair consisting of the noisy input and the reference output. The process of the generation of input and output SR-SIM images from the raw SIM images is shown in Fig. 1. The 15 raw SIM images of size 512 × 512 (width × height) pixels of different phases and orientations are propagated into the fairSIM algorithm to reconstruct the SR-SIM images of size 1,024 × 1,024 (width × height) pixels. During the formation of this dataset, the raw samples from timestamp 1 were used to generate the output images (i.e., reference images). We use the term *reference images* instead of *ground-truth images* because of the SIM reconstruction artifacts in the output images of this dataset. The input samples were reconstructed by using the raw SIM images from timestamps 176 to 200. Therefore, dataset 2 is composed of 2,525 reconstructed pairs of SR-SIM images with a size of 1,024 × 1,024 (width × height) pixels extracted from the 101 FOVs. In addition to these 2,525 image pairs, for the last 20 FOVs, we also include image pairs in the data collection where the noisy input is from timestamps 76 to 100, 126 to 150, and 176 to 200. These additional data can be used to create test sets to evaluate the robustness of denoising networks for different noise levels. In our previous work [19], we denoted data from timestamp 26 to 50 as noise level 1. Similarly, noise levels 2, 3, and 4 correspond to the data from the timestamps 76 to 100, 126 to 150, and 176 to 200 (shown in Fig. 3). Overall, dataset 2 is generated mainly for the denoising of SR-SIM images.

#### Dataset 3

Dataset 3 was created mainly for joint denoising and super-resolution tasks. The composition of this dataset is based on the stack of raw noisy input SIM images obtained from dataset 1 of noise level 4 and the reference SR-SIM images from dataset 2 of timestamp 1. The raw noisy SIM images in the input stack represent different illumination phases and orientations. The reference samples are the reconstructed high-resolution SR-SIM images. The size and dimension of each input sample are 15 × 512 × 512 (depth × width × height) pixels. The size of the output or reference sample is 1,024 × 1,024 (width × height) pixels. The input and reference samples in this dataset contain tubulin filaments as biological structures.

#### Dataset 4

Dataset 4 is composed of 3D-SR-SIM images showing vesicles in U2OS cells (i.e., typically round intracellular droplets) and can be used mainly for denoising tasks. Full 3D SIM raw data stacks have been passed through a 3D-enabled SIM reconstruction, extending both lateral and axial resolution of the data [15].

These data are composed of 9 z-stacks, each with a varying number of depth levels (z-planes) and each captured for a different number of timestamps. In our work, we focus on the denoising along the lateral plane; therefore, each z-stack is split into its single z-planes (also called “slices”). The recorded structures in the different z-planes are very different from each other, so we categorize them here as separate FOVs (the filenames in the dataset contain also indices for the original z-stack).

As a result, the raw data of this dataset contain 175 FOVs, and each FOV is recorded for a different number of timestamps. This yields a total of 7,284 pairs of input and output SR-SIM images. The size of the input and reference images is 1,024 × 1,024 (width × height) pixels. In each image pair, the output/reference image is taken from timestamp 1 of the respective FOV, whereas the input images are taken from the following timestamps and exhibit therefore a lower SNR. The rational for splitting 3D data into separate 2D slices for denoising and artifact removal is as follows: the structure of the 3D SIM reconstruction algorithm (shifting and recombining lateral but not axial frequencies) makes it very likely that noise and reconstruction artifacts arise in the lateral directions of the acquired images, not so much in the axial component. Limiting the postprocessing to 2D increases speed and reduces training effort significantly. Also, the height of a 3D stack (number of slices) varies from sample to sample, while the lateral FOV is typically constant. Of course, dataset 4 would allow full 3D implementations to be tested, by recombining the z-sliced data into 3D volumes. The reconstruction of the original raw SIM images into the SR-SIM images in this dataset was performed by using the softWoRx v7 software (GE Healthcare manufacturer’s software).

#### Data partitioning

In our experiments, in the first 3 datasets, the images of the last 20 FOVs were used as test samples, and the remaining 81 FOVs were used for the training set. The training set of dataset 2 is therefore composed of 2,025 samples (image pairs) from 81 FOVs, and the test set is composed of 500 samples from 20 FOVs. Similarly, in dataset 4, we used 5,562 samples from 121 FOVs for the training set and 1,380 samples from 46 FOVs for the test set; the remaining 8 FOVs were discarded because their reference images contain only noise without any meaningful structure. Datasets 1, 2, and 4 can only be used for denoising tasks, but the data from dataset 3 can be used for both joint denoising and super-resolution. To reproduce the results of this work, we shared the source code on the GitHub repository [42], workflowhub [43], as well as the trained models at [44], and the data on GigaDB [45].

### Shifted window Transformer for the denoising of SR-SIM images (SwinT-fairSIM)

**Figure 4:**
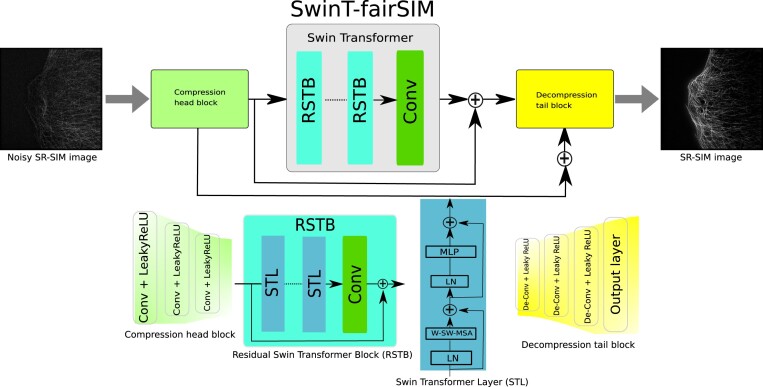
The architecture of the SwinT-fairSIM denoising method. The lower half of the figure provides the description of each block.

The architecture of SwinT-fairSIM is composed of 3 main components: compression head, Swin Transformer, and decompression tail block, as shown in Fig. 4. The architecture of SwinT-fairSIM is an extended version of the SwinIR architecture [36]. The compression head block is responsible for shallow feature extraction and the downsizing of the input images, the Swin Transformer block is based on encoder–decoder components to recover missing information and perform deep feature extraction, and the decompression tail component is used for upsampling and recovering the features in the resulting images. The compression head block is composed of 3 convolution layers to compress the input image features to a size of 256 × 256 (width × height) pixels by using a stride of size 2. The encoder–decoder Swin Transformer block is made up of several residual Swin Transformer blocks (RSTBs), and each RSTB is based on few Swin Transformer layers (STLs) and convolutional layers [36]. The STL is further composed of 2 window (W) and shifted window (SW)–based multihead self-attention (MSA) modules, followed by a multilayer perceptron that has 2 fully connected layers with GELU non-linearity [25, 35]. The shifted window partitioning strategy introduces connections between non-overlapping patches in the preceding layer and is found to be effective in a variety of computer vision tasks [36]. The Swin Transformer layer first reshapes the input (X) of size H × W × C (height × width × channels) into $\frac{HW}{M^2} \times M^2 \times C$, where the $\frac{HW}{M^2}$ is the total number of windows or patches. Two additive-based residual skip connections are also used in each STL unit. The query (Q), key (K), and value (V) matrices are then calculated for each window separately to obtain self-attention.


(1) \begin{eqnarray*}
Q = X P_{Q},\: K=X P_{K},\: V= X P_{V}
\end{eqnarray*}

In equation (1), *P_Q_*, *P_K_*, and *P_V_* are the shared projection matrices across all the windows. The attention matrix is then computed by the self-attention mechanism in a local window as follows:


(2) \begin{eqnarray*}
\mbox{Attention}(Q,K,V) = \mbox{SoftMax}(QK^T / \sqrt{d} + B)V
\end{eqnarray*}

where *B* in equation (2) is the learnable relative positional encoding and *d* is the dimension of query and key features. The consecutive Swin Transformer layers are defined as:


(3) \begin{eqnarray*}
\hat{X}^l &=& \mbox{W-MSA} (\mbox{LN}(X^{l-1})) + X^{l-1}
\end{eqnarray*}

(4) \begin{eqnarray*}
X^{l} &=& \mbox{MLP}(\mbox{LN}(\hat{X}^{l})) + \hat{X}^{l}
\end{eqnarray*}

(5) \begin{eqnarray*}
\hat{X}^{l+1} &=& \mbox{SW-MSA}(\mbox{LN}(X^{l})) + X^{l}
\end{eqnarray*}

(6) \begin{eqnarray*}
X^{l+1} &=& \mbox{MLP}(\mbox{LN}(\hat{X}^{l+1})) + \hat{X}^{l+1}
\end{eqnarray*}

In equations (3) to (6), $\hat{X}^l$ and *X*^*l*^ denote the output features of the (S)W-MSA and MLP modules for layer *l*, where LN represents the LayerNorm operation.

Finally, the decompression tail block accommodates 3 transposed convolutional layers along with the output convolutional layer to transform and upsample the feature maps into the final resulting images. The necessity of head and tail block is to scale down and upsample the SR-SIM images with a size of 1,024 × 1,024 (width × height) pixels. In the SwinT-fairSIM method, we set the RSBT, STL, window size, and the attention head numbers to 5, 5, 8, and 120, respectively. The adjacent layers in the head and tail blocks are connected via additive skip connections and contain the same number of kernels. These blocks differentiate our architecture from the existing SwinIR architecture [36]. These blocks reduce the computational effort in the central Transformer part of the architecture and therefore adapt the Swin Transformer block to images with a high pixel count.

The name “SwinT-fairSIM” was chosen to be consistent with the names of our previously proposed CNN-based denoising methods for SR-SIM data, “Red-fairSIM” and “UNet-fairSIM” [16]. Red-fairSIM and UNet-fairSIM are described in detail in [16]. These algorithms originally worked only in conjunction with the fairSIM software [7] for SR-SIM reconstruction. In contrast, in this article, we also use the softWoRx implementation of the SIM algorithm for dataset 4. However, to be consistent with the naming scheme in [16], we keep the suffix “fairSIM” in all of our deep learning–based approaches.

### Transfer learning and fine-tuning

In transfer learning in general, the knowledge of a trained model from a related task that has been learned is transferred to another task from the same domain [46]. The parameters of the pretrained model, trained on specific data and for a specific task, are transferred to different data but a related task [47, 48]. The use of transfer learning in deep learning is very useful to reduce the computational demands and time complexity [49]. In addition, transfer learning is also very helpful when it comes to alleviating large dataset requirements [50].

**Figure 5:**
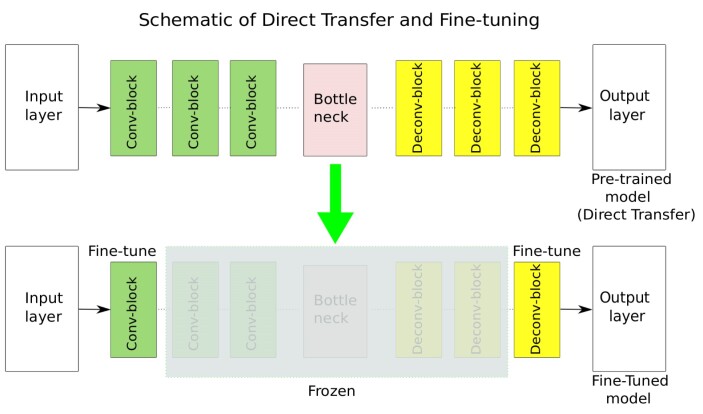
The schematic diagram illustrates the process of direct transfer and fine-tuning. In the first pipeline, the pretrained model is depicted. The highlighted region in the second pipeline represents the frozen part, which remains unchanged during fine-tuning.

The simplest form of transfer learning is to apply a trained model directly to another task without retraining. This direct use of a pretrained model on new test data is called “direct transfer.” Beyond this most basic approach, fine-tuning is one of the most important strategies for transferring model knowledge from one domain to another [51]. During fine-tuning, the weights of some layers of pretrained models are preserved and the rest of the layers are retrained (i.e., “fine-tuned”) [52]. The concepts of direct transfer and fine-tuning are illustrated in Fig. 5. It can be seen from the schematic in Fig. 5 that the weights of the intermediate layers of the pretrained models are frozen while the rest of the layers are retrained in the fine-tuning strategy, whereas no retraining is performed in the direct transfer approach. For our work here, in the direct transfer strategy, the models are trained from scratch on dataset 2, and then the trained model is evaluated with test samples from dataset 4. Similarly, the models trained on dataset 4 are evaluated on the test images from dataset 2. In the fine-tuning strategy, models are trained from scratch on dataset 2; afterward, they are partly retrained on dataset 4 and also tested on dataset 4. Or the other way round: complete initial training on dataset 4, afterward fine-tuning on dataset 2, and finally evaluation on dataset 2. In this work, we performed these different training strategies with the SwinT-fairSIM, Red-fairSIM, and UNet-fairSIM algorithms [19] with datasets 2 and 4.

To perform fine-tuning on the SwinT-fairSIM algorithm, we retrained the head and tail blocks along with 1 adjacent RSTB block each. By doing so, about 1.9 million training parameters stayed frozen out of 4 million parameters. Regarding the CNN-based algorithms from our previous work [19], we used the following approaches to fine-tuning: Red-fairSIM is based on the residual–encoder–decoder network (RED-Net) [53]. In the fine-tuning approach for Red-fairSIM, we retrained the first and last 5 layers of the model instead of all 30 layers. By doing so, about 700,000 training parameters stayed frozen out of more than 1 million parameters. UNet-fairSIM is based on the UNet architecture, which is based on several encoder and decoder blocks [54]. In the fine-tuning of UNet-fairSIM, we simply retrained the first and last 2 encoder and decoder blocks, leaving more than 29 million out of 33 million parameters frozen.

Generally, in the first step, all these algorithms were trained from scratch on datasets 2 and 4 separately for 100 epochs. The mean squared error (MSE) was used as a loss function for all training runs with the Adam optimizer. The learning rate was set to 1 × 10^−4^ for all training runs. In the next step, we applied direct transfer by propagating the test samples from the respective dataset *not* used for training the pretrained models. In direct transfer, the test images of the dataset 2 (i.e., tubulin filaments) were propagated through the pretrained model trained on the dataset 4 (i.e., vesicle structure) and vice versa. In the third step, the pretrained models were fine-tuned using training data from the respective dataset *not* used for initial training. Thirty training epochs were used for fine-tuning. The fine-tuned models were then evaluated on the test samples from the dataset used in the fine-tuning process. When fine-tuning the pretrained models, we initially kept different numbers of layers/blocks frozen. However, we found that the best results were obtained by fine-tuning rather few trainable parameters for 30 epochs (except for SwinT-fairSIM, where a larger part of the network had to be “unfrozen”). In the final step, we compared the results of conventional training with the direct transfer and fine-tuning strategies visually, as well as in terms of peak signal-to-noise ratio (PSNR) [55] and structural similarity index measurement (SSIM) [56] values.

## Results

We collected 4 data sets for denoising and super-resolution tasks. The overview and full characteristics of these datasets are shown in Table 1. The images in dataset 2 contain the tubulin structure along with mixed Poisson–Gaussian (MPG) noise and SR-SIM reconstruction artifacts. The images in dataset 4 contain the vesicle structure along with MPG noise and honeycomb pattern artifacts that occur when raw data carrying predominantly Poisson noise are subjected to the frequency-based SR-SIM reconstruction algorithm, which then introduces reconstruction artifacts [14].

In our previous studies [19], we already had used the first 3 datasets for the denoising and super-resolution tasks. Here, we mainly focus on the denoising of SR-SIM images by SwinTransfomer-fairSIM and compare the results of this new method with the results of Red-fairSIM and UNet-fairSIM [19]. We also analyze 2 transfer learning strategies, direct transfer and fine-tuning of the pretrained models of these network architectures.

**Figure 6:**
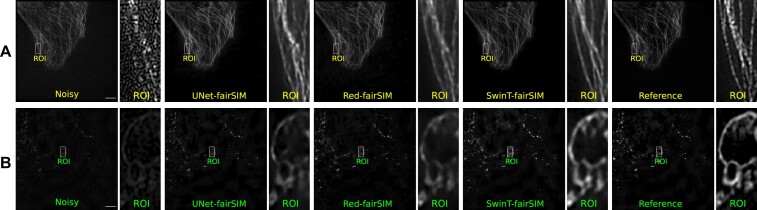
The results of the test samples from datasets 2 and 4 are shown in the first (A) and second (B) rows, respectively. UNet-fairSIM, Red-fairSIM, and SwinT-fairSIM outputs are shown side by side. The cropped and zoomed ROIs extracted from the full-size SR-SIM images with 1,024 × 1,024 pixels are displayed in columns 2, 4, 6, 8, and 10. The cropped ROIs of size 50 × 100 pixels were upsampled to 150 × 300 pixels for demonstration purposes. Scale bar: $4 {\, \mu {\rm m}}$.

To compare the performance of conventional training of these deep learning–based denoising methods with direct transfer and fine-tuning, we first train the SwinT-fairSIM, Red-fairSIM, and UNet-fairSIM networks separately with datasets 2 and 4 for 100 epochs. The results of all methods trained separately with both datasets are shown visually in Fig. 6 and quantitatively in Table 2. The visual and quantitative results of SwinT-fairSIM show superiority over the Red-fairSIM and UNet-fairSIM methods. The resulting regions of interest (ROIs) of both datasets from SwinT-fairSIM are more appealing and sharper than those of the other methods in Fig. 6. The PSNR and SSIM values of SwinT-fairSIM are also slightly higher than those of Red-fairSIM and UNet-fairSIM. Overall, SwinT-fairSIM outperforms its counterparts on both datasets 2 and 4 after conventional training from scratch.

**Table 2:** Mean PSNR and SSIM values along with standard deviations (SDs) of all experiments calculated on the noisy test images of datasets 2 and 4 (for dataset 2, noise level 4 was used). The test data contain 500 images for dataset 2 and 1,380 images for dataset 4. The PSNR and SSIM values are calculated relative to the reference images (i.e., SR-SIM images reconstructed with fairSIM/softWoRx from raw SIM images with the highest signal-to-noise ratio). In the row entitled “fairSIM/softWoRx,” PSNR and SSIM values are calculated for the direct fairSIM/softWoRx reconstruction of the noisy images. The rows entitled “SwinT-fairSIM,” “Red-fairSIM,” and “UNet-fairSIM” show the results for the denoised test images after conventional training from scratch with the respective algorithms. In the direct transfer rows, the mean PSNR and SSIM values in the column “dataset 2” are calculated for models that are initially trained on dataset 4 and afterward tested with the test samples from dataset 2. For the column “dataset 4,” this is the other way round. The same principle holds for the rows for fine-tuning, only that the models are fine-tuned on the respective dataset before testing.

	Mean (SD) PSNR and SSIM values of test data			
	Dataset 2		Dataset 4	
	PSNR	SSIM	PSNR	SSIM
fairSIM/softWoRx	23.61 (1.54)	0.29 (0.07)	35.10 (2.71)	0.86 (0.03)
SwinT-fairSIM	28.19 (2.09)	0.72 (0.09)	38.44 (1.73)	0.89 (0.02)
Direct transfer (SwinT-fairSIM)	26.07 (1.91)	0.48 (0.07)	34.05 (2.24)	0.83 (0.03)
Fine-tuning (SwinT-fairSIM)	28.15 (1.87)	0.72 (0.08)	38.52 (1.50)	0.89 (0.02)
Red-fairSIM	27.97 (2.01)	0.71 (0.09)	38.43 (1.45)	0.89 (0.01)
Direct transfer (Red-fairSIM)	23.31 (1.68)	0.41 (0.07)	33.89 (1.93)	0.81 (0.03)
Fine-tuning (Red-fairSIM)	27.90 (2.14)	0.70 (0.09)	38.30 (1.60)	0.88 (0.02)
UNet-fairSIM	26.80 (1.65)	0.68 (0.10)	37.45 (1.79)	0.88 (0.02)
Direct transfer (UNet-fairSIM)	24.69 (1.54)	0.46 (0.05)	34.47 (2.24)	0.83 (0.04)
Fine-tuning (UNet-fairSIM)	28.02 (1.97)	0.71 (0.07)	38.35 (1.44)	0.89 (0.01)

In the next step, we evaluate the direct transfer strategy by using these pretrained models. In direct transfer, the model initially trained with dataset 2 is tested with the test samples of an alternative dataset (i.e., dataset 4) and vice versa. Similarly, in the assessment of the fine-tuning strategy, we retrained the first and last layers of the pretrained models with the alternative data to see the improvement in the generalization power of the pretrained models. The resulting denoised images obtained by direct transfer of models trained on vesicle structures (dataset 4) to test images containing tubulin filaments (dataset 2) are displayed within block A of Figs. 8 and 9. These images clearly show that the model trained on a specific type of noise and structure (i.e., vesicle images with honeycomb pattern noise) is not able to produce a refined denoised image of another structure (i.e., tubulin filaments with MPG noise). Similarly, the models initially trained on tubulin filaments from dataset 2 are not able to properly produce the denoised images of dataset 4 with different structure and noise (see block B of Figs. 8 and 9). However, it can be noticed that the pretrained models try to replicate the filamentous structure of tubulin in the vesicle data (very prominent in block B of Fig. 8). This is a clear indication that these deep learning–based denoising models are not robust against different types of noise and structure. However, the outcomes of the fine-tuning approach are promising, as shown in Figs. 8 and 9. The ROIs 1 and 2 in column 6 of Fig. 8 and also the ROIs in column 4 of Fig. 9 show that the results of the fine-tuning strategy are very close to the results of training from scratch for the SwinT-fairSIM, Red-fairSIM, and UNet-fairSIM algorithms.

**Figure 7:**
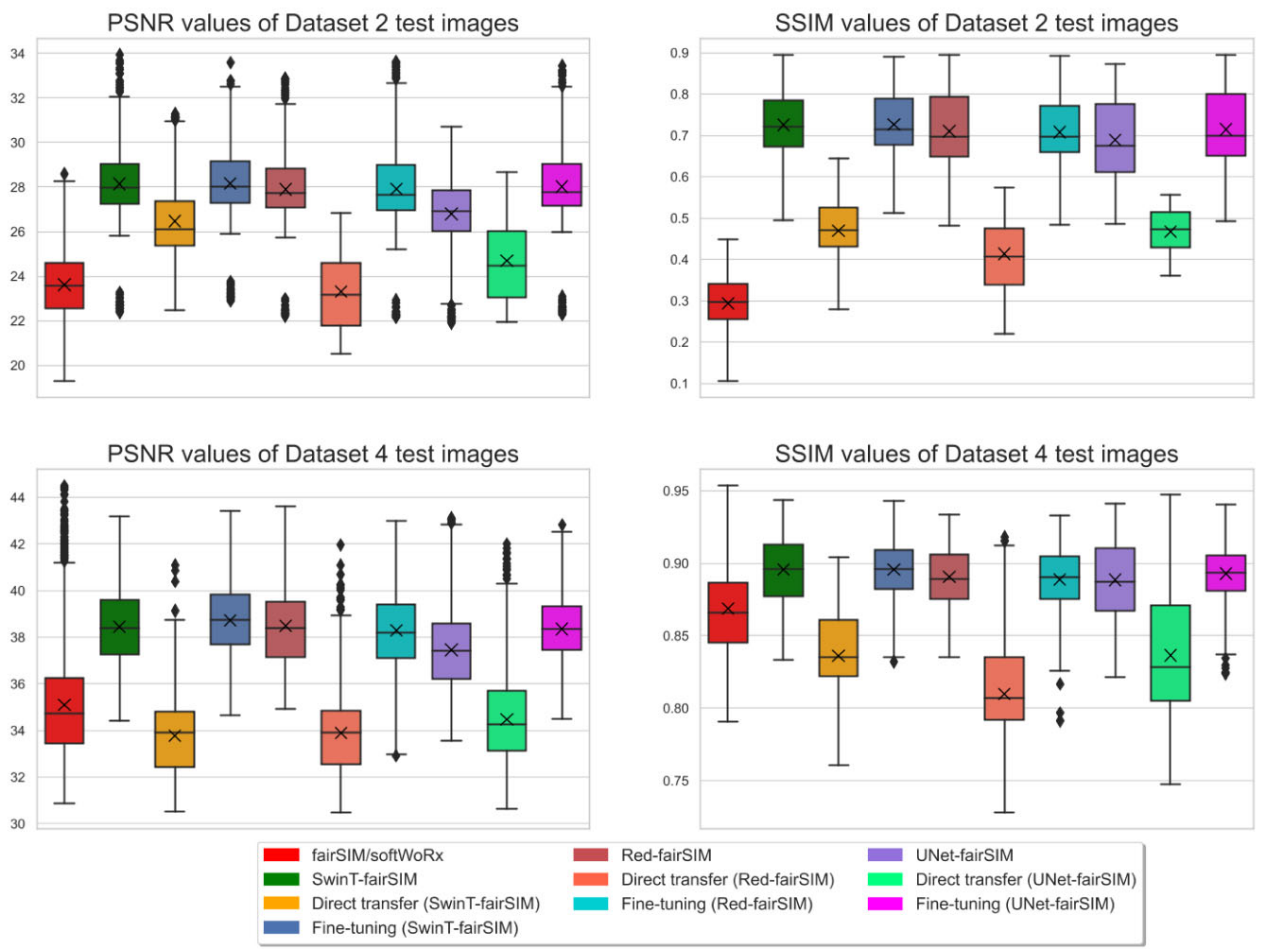
Boxplots of PSNR and SSIM values of test samples of dataset 2 and dataset 4 with different denoising algorithms and training strategies. The test data contain 500 images from noise level 4 for dataset 2 and 1,380 images for dataset 4. The horizontal line inside each boxplot represents the median value, whereas the black cross displays the mean values. The diamond-shaped black markers display the outlier observations. The color of the boxes in each subplot refers to the methods defined in the legend box.

**Figure 8:**
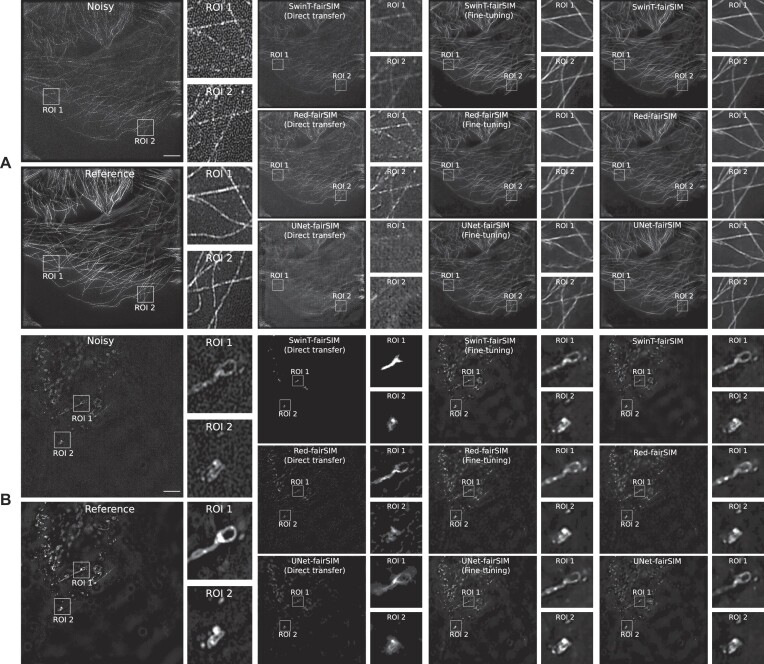
Blocks A and B show the results for test samples from dataset 2 (block A) and dataset 4 (block B) for different algorithms and learning strategies. The first and second columns of blocks A and B show the noisy and reference SR-SIM images, respectively, along with extracted and magnified ROIs. The noisy images are the test input for all algorithms. Columns 3 to 6 show the results of direct transfer and fine-tuning with SwinT-fairSIM, Red-fairSIM, and UNet-fairSIM. In direct transfer, the model is trained with dataset 2 and tested with the test sample of dataset 4 and vice versa. Similarly, in fine-tuning, the model is first pretrained on dataset 2 and then fine-tuned and tested on dataset 4 and vice versa. Columns 7 and 8 show the results of all algorithms when trained from scratch on the respective dataset, which is also used for testing. Columns 4, 6, and 8 show the cropped and enlarged ROIs from the full-size SR-SIM images to the left of each ROI. The ROIs of size 100 × 100 pixels have been upsampled to 300 × 300 pixels for illustration purposes. Scale bar: $4 {\, \mu {\rm m}}$.

**Figure 9:**
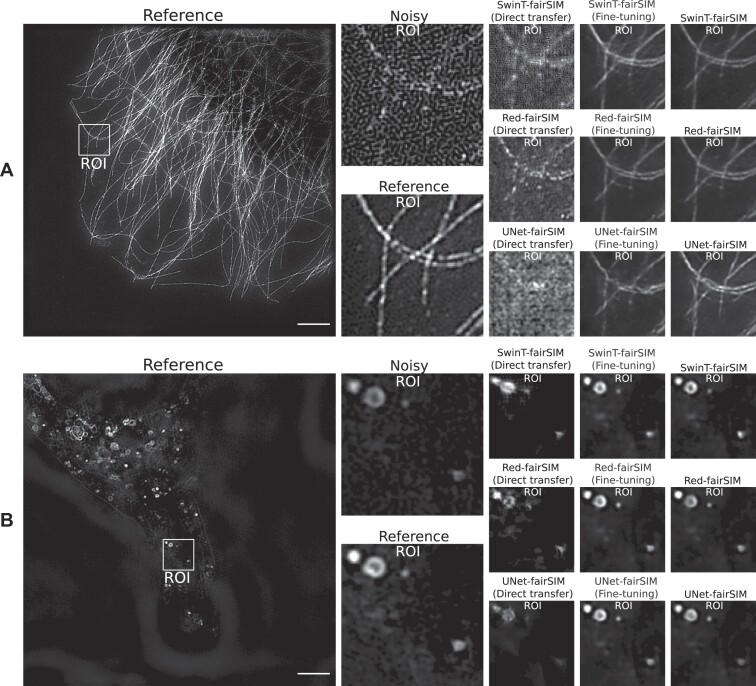
Two more test samples from datasets 2 and 4 are shown in this figure. Both blocks A and B contain the reference SR-SIM images along with the resultant denoised ROIs, which are extracted from the full-size denoised images of all the methods used in this work. The extracted ROIs are upsampled from 100 × 100 pixels to 300 × 300 pixels for illustration purposes. Scale bar: $4 {\, \mu {\rm m}}$.

The overall comparison of all the approaches in Figs. 8 and 9 demonstrates that the fine-tuning method is crucial for the Transformer- and CNN-based denoising algorithms in the case of changes in structure or noise types. Thus, fine-tuning is inevitably required to profit from knowledge transfer. Table 2 lists the average PSNR and SSIM values of the test images of datasets 2 and 4 calculated for all the training and testing strategies of SwinT-fairSIM, Red-fairSIM, and UNet-fairSIM. Table 2 clearly points out the decline in the average PSNR and SSIM values on both datasets 2 and 4 after the application of direct transfer. However, the average PSNR and SSIM values show considerable improvement after fine-tuning. The average PSNR and SSIM values of the fine-tuned models in Table 2 are very close to the models that were trained from scratch (partly slightly better, partly slightly worse). In addition, Fig. 7 shows boxplots of the PSNR and SSIM values of the test samples of both datasets with all different methods/strategies. The boxplots complement the mean values in Table 2 by depicting additional statistics. It can be observed that SwinT-fairSIM often has not only higher mean and median values but also a more consistent interquartile range in all of the boxplots, as shown in Fig. 7. In the SSIM boxplots of dataset 2, very few outliers are noticeable overall.

Compared to conventional training, the fine-tuning strategy retrains only a subset of the entire set of model parameters. In this study, we retrained about 2.1 million parameters out of 4 million parameters of SwinT-fairSIM, while for Red-fairSIM and UNet-fairSIM, out of more than 1 million and 33 million training parameters, only 295,000 and 3.7 million parameters were retrained, respectively. We were able to achieve results comparable to those of conventional training after only 30 epochs as opposed to 100 epochs. Training times for dataset 2 on 2 Nvida V100 GPUs (32 GB) were (training from scratch vs. fine-tuning):


\begin{eqnarray*}
\hbox{SwinT-fairSIM:} & \quad \hbox{26 h vs.3.25 h} \\ \hbox{Red-fairSIM:} & \quad \hbox{24 h vs. 3 h} \\ \hbox{UNet-fairSIM:} & \quad \hbox{18 h vs. 1.5 h}\\ \end{eqnarray*}

Therefore, in terms of computational requirements or memory consumption, it is advantageous to use fine-tuning instead of training from scratch. Fine-tuning is beneficial when the computational resources are limited.

## Discussion

In summary, the contribution of this work is 3-fold: first, we publish novel datasets related to SIM microscopy, and then we explore a Transformer-based algorithm for the restoration of SR-SIM images. Finally, we investigate the potential of the direct transfer and fine-tuning strategies for various deep learning–based denoising algorithms. Regarding datasets, we provide 4 novel datasets for testing denoising and super-resolution image reconstruction strategies with tubulin filaments and vesicle structures. These datasets contain a large number of raw SIM images and reconstructed 2-fold super-resolved SIM images and cover different noise levels, a wide range of fields of view, and varying degrees of structural complexity. High-quality data from the real world of microscopy are highly relevant for benchmarking and evaluating current and upcoming denoising and super-resolution methods.

Regarding our work with Transformers, we suggested the SwinT-fairSIM architecture to produce high-quality SR-SIM images from low SNR inputs. Importantly, we visually and quantitatively showed that the Transformer-based method can achieve mostly better results than the CNN-based Red-fairSIM and UNet-fairSIM methods (i.e., for the quantitative results, PSNR and SSIM values, see Table 2 and the boxplots in Fig. 7). We demonstrated that SwinT-fairSIM can retrieve more well-preserved cell structures and texture information than the CNN-based methods, especially in the ROIs of Fig. 6. This is an important finding, since most existing SR-SIM restoration methods are based on CNNs [17–19, 21–23, 38, 39]. An explanation for the better performance of SwinT-fairSIM may be the size of the receptive fields of the different network architectures. In CNNs, the size of their receptive fields is limited by the size and stride of the filter kernels and the depth of the network. In contrast, Swin Transformers may obtain a larger receptive field by the shifted windows operation [57, 58].

In the last section of this work, we showed the limitations of Transformer- and CNN-based deep learning methods in the area of knowledge transfer when it comes to different types of noise and structure. In the direct transfer strategy, we simply evaluated the pretrained networks with new test data. The direct transfer strategy exhibits very downgraded visual (in Figs. 8 and 9) and quantitative (in Table 2 and Fig. 7) results. However, the approach of fine-tuning enables these deep learning models to generalize well to other noise types and structures after retraining few layers. This holds for all learning algorithms and datasets in a similar way, as shown in Figs. 7, 8, and 9 and in Table 2. All of the deep neural network architectures tested here are, at their core, encoder–decoder architectures (for SwinT-fairSIM, this applies at least to the compression head block and the decompression tail block). In these architectures, the initial and final layers are responsible for handling low-level features. Noise patterns can be interpreted as low-level features that are separated from the valuable input during the encoding process so that they can be selectively suppressed at a later stage during decoding.

Accordingly, we retrained only the initial and final layers of all 3 types of neural networks to achieve results on par with training from scratch. It is worth mentioning that we also attempted to retrain only the first, middle, or last layers during first exploratory research and also varied the number of retrained layers, but the results obtained in this way were not competitive. Since fine-tuning often saves computation time and often requires fewer learning samples, we conclude that fine-tuning of pretrained models has advantages over conventional training from scratch, at least when the difference between the task domains is not huge. In contrast, direct transfer obviously fails because the learned detectors for low-level features (noise patterns and typical simple visual features of the main cell structures) are too much tuned to the original training data.

There are still some limitations of this work. First, while our datasets are useful for various image restoration tasks and contain more samples than many of the datasets previously published in the field, they are limited to 2 structures, tubulin filaments and vesicle structures. Second, our results indicate the superiority of SwinT-fairSIM over Red-fairSIM and UNet-fairSIM. However, this improvement leads to higher computational costs. Third, fine-tuning strategies provide similar results to training from scratch at lower cost, but it is often difficult to find task-specific pretrained networks. Finally, slice-by-slice denoising of 3D SIM images focuses exclusively on the lateral plane; despite this limitation, it was shown that the presented approach can still be very useful for denoising 3D SIM structures.

## Conclusion

In this work, we first presented a series of datasets for SIM image restoration tasks such as denoising and super-resolution. Additionally, we proposed a new algorithm based on the Swin Transformer, “SwinT-fairSIM,” for the denoising of SR-SIM images. We showed that this Transformer-based algorithm outperforms CNN-based denoising algorithms both visually and quantitatively. This suggests that Transformer-based denoising algorithms can play an important role in the field of SR-SIM microscopy. Similarly, we evaluated different knowledge transfer strategies such as direct transfer and fine-tuning for Transformer- and CNN-based denoising algorithms. For the direct transfer strategy, we noticed a decline in the performance of these algorithms. However, we were able to recover the declined performance by the fine-tuning strategy. This clearly indicates that the Transformer- and CNN-based denoising methods require retraining of some initial and final layers of the pretrained models when applied to new biological structures and noise types. This retraining requires fewer epochs than the conventional training from scratch. This holds also for the novel SwinT-fairSIM denoising algorithm, which outperforms all CNN-based algorithm also in the fine-tuning regime.

## Potential Implications

The implementation of different deep learning methods requires a large quantity of images in order to train the underlying models. We believe that our published datasets will help the research community to develop new deep learning–based methods and evaluate the existing methods by either training from scratch or by applying fine-tuning. These datasets can be especially used for image denoising and super-resolution tasks.

## Availability of Supporting Source Code and Requirements

- Project name: SwinT-fairSIM-and-knowledge-transfer [42]
- Project homepage: https://github.com/ZafranShah/SwinT-fairSIM-and-knowledge-transfer
- Operating system(s): Platform independent
- Programming language: Python
- Other requirements: Python 3.7.9 or higher, tensorflow-gpu 2.5.0 or higher, pillow 8.1.0, opencv-python 4.5.5.64
- License: GNU GPL
- Workflowhub.eu: https://doi.org/10.48546/WORKFLOWHUB.WORKFLOW.675.1
- RRID: SCR_024715

## Supplementary Material

giad109_GIGA-D-23-00044_Original_Submission

giad109_GIGA-D-23-00044_Revision_1

giad109_GIGA-D-23-00044_Revision_2

giad109_GIGA-D-23-00044_Revision_3

giad109_Response_to_Reviewer_Comments_Original_Submission

giad109_Response_to_Reviewer_Comments_Revision_1

giad109_Response_to_Reviewer_Comments_Revision_2

giad109_Reviewer_1_Report_Original_Submission Xiaoshuai Huang -- 3/29/2023 Reviewed

giad109_Reviewer_1_Report_Revision_1 Xiaoshuai Huang -- 8/4/2023 Reviewed

giad109_Reviewer_2_Report_Original_Submission Jun Li -- 3/31/2023 Reviewed

giad109_Reviewer_2_Report_Revision_1 Jun Li -- 7/23/2023 Reviewed

giad109_Reviewer_3_Report_Original_Submission Yicong Wu -- 4/4/2023 Reviewed

giad109_Reviewer_3_Report_Revision_1 Yicong Wu -- 7/21/2023 Reviewed

## Data Availability

The raw and reconstructed SIM datasets described in this article are available via the *GigaScience* database, GigaDB [45]. All the datasets are distributed under the Creative Commons CC0 license. Similarly, all of the models trained during this work are freely accessible through Zenodo [44].

## Abbreviations

CNN: convolutional neural network; fairSIM: free analysis and interactive reconstruction for structured illumination microscopy; GAN: generative adversarial network; MSA: multihead self-attention; MSE: mean square error; NA: numerical aperture; PSNR: peak-signal-to-noise ratio; SIM: structured illumination microscopy; SR-SIM: super-resolution structured illumination microscopy; SSIM: structural similarity index measurement; SNR: signal-to-noise ratio; W2S: wide-field2SIM.
