# Supplementary material for: Evaluation of Swin Transformer and knowledge transfer for denoising of super-resolution structured illumination microscopy data
Source: Gigascience. 2024 Jan 13;13:giad109. doi: 10.1093/gigascience/giad109 (PMC10787368; doi:10.1093/gigascience/giad109)
Supplement: giad109_GIGA-D-23-00044_Original_Submission [file giad109_giga-d-23-00044_original_submission.pdf]

GigaScience

GigaScience , 2023, 1–9 doi: xx.xxxx/xxxx

Evaluation of knowledge transfer for the denoising of super-resolution structured illumination microscopy data

--Manuscript Draft--

**Manuscript Number:**	GIGA-D-23-00044
**Full Title:**	GigaScience , 2023, 1–9 doi: xx.xxxx/xxxx<br>Evaluation of knowledge transfer for the denoising of super-resolution structured illumination microscopy data
**Article Type:**	Technical Note
**Funding Information:**	Bundesministerium für Bildung und Forschung (01IS18041C) — Mr. Wolfram Schenck<br>H2020 Marie Skłodowska-Curie Actions (642157) — Mr. Thomas Huser<br>Deutsche Forschungsgemeinschaft (415832635) — Mr. Thomas Huser
**Abstract:**	In recent years, convolutional neural network (CNN) based methods have shown remarkable performance in the denoising and reconstruction of super-resolved structured illumination microscopy (SR-SIM) data. The potential for the generalization of these deep-learning models to different real-world fluorescence microscopy data has, however, not yet been completely explored. The question arises whether such CNN-based denoising methods are structure- and noise-specific. Here, we apply transfer learning and fine-tuning strategies to assess the generalization capability of these methods to different domains, in particular super-resolved microscopy images of different biological structures, before and after knowledge transfer. We first provide four novel real-world microscopy datasets with different noise levels to train and evaluate the deep learning based denoising and super-resolution approaches. Secondly, we extensively investigate the performance of CNN-based denoising networks on data from different domains (i.e. different biological structures) by applying transfer learning strategies, such as direct transfer and fine-tuning. In the last step, we demonstrate that the fine-tuning approach is more advantageous than the conventional training of CNN-based denoising methods to avoid computational overhead.
**Corresponding Author:**	Zafran Hussain Shah<br>Bielefeld University of Applied Sciences: Fachhochschule Bielefeld<br>Bielefeld, North Rhine-Westphalia GERMANY
**Corresponding Author Secondary Information:**	
**Corresponding Author's Institution:**	Bielefeld University of Applied Sciences: Fachhochschule Bielefeld
**Corresponding Author's Secondary Institution:**	
**First Author:**	Zafran Hussain Shah
**First Author Secondary Information:**	
**Order of Authors:**	Zafran Hussain Shah
	Marcel Müller, Doctorate
	Wolfgang Hübner, Doctorate
	Tung-Cheng Wang, Doctorate
	Daniel Telman, Bachelor
	Thomas Huser, Doctorate
	Wolfram Schenck, Doctorate

Powered by Editorial Manager® and ProduXion Manager® from Aries Systems Corporation

Order of Authors Secondary Information:	
Additional Information:	
Question	Response
Are you submitting this manuscript to a special series or article collection?	No
**Experimental design and statistics**<br><br>Full details of the experimental design and statistical methods used should be given in the Methods section, as detailed in our Minimum Standards Reporting Checklist. Information essential to interpreting the data presented should be made available in the figure legends.<br><br>Have you included all the information requested in your manuscript?	Yes
**Resources**<br><br>A description of all resources used, including antibodies, cell lines, animals and software tools, with enough information to allow them to be uniquely identified, should be included in the Methods section. Authors are strongly encouraged to cite Research Resource Identifiers (RRIDs) for antibodies, model organisms and tools, where possible.<br><br>Have you included the information requested as detailed in our Minimum Standards Reporting Checklist?	Yes
**Availability of data and materials**<br><br>All datasets and code on which the conclusions of the paper rely must be either included in your submission or deposited in publicly available repositories (where available and ethically appropriate), referencing such data using a unique identifier in the references and in the "Availability of Data and Materials" section of your manuscript.	Yes

Powered by Editorial Manager® and ProduXion Manager® from Aries Systems Corporation

Have you have met the above requirement as detailed in our Minimum Standards Reporting Checklist?	

Powered by Editorial Manager® and ProduXion Manager® from Aries Systems Corporation

Manuscript Click here to access/download;Manuscript;GigaScience_Technical_Note_M

OXFORD (GIGA)^n SCIENCE

GigaScience, 2023, 1–9

doi: xx.xxxx/xxxx
Manuscript in Preparation
Paper

PAPER

Evaluation of knowledge transfer for the denoising of super-resolution structured illumination microscopy data

Zafran Hussain Shah[1,*], Marcel Müller[2], Wolfgang Hübner[2], Tung-Cheng Wang[2,3], Daniel Telman[1], Thomas Huser[2] and Wolfram Schenck[1,*]

[1]Faculty of Engineering and Mathematics, Bielefeld University of Applied Sciences, Interaktion 1, 33619 Bielefeld, Germany and [2]Faculty of Physics, Bielefeld University, Universitätsstr. 25, 33615 Bielefeld, Germany and [3]Leica Microsystems CMS GmbH, Am Friedensplatz 3, 68165 Mannheim, Germany

[*]zafran_hussain.shah@fh-bielefeld.de; wolfram.schenck@fh-bielefeld.de

Abstract

In recent years, convolutional neural network (CNN) based methods have shown remarkable performance in the denoising and reconstruction of super-resolved structured illumination microscopy (SR-SIM) data. The potential for the generalization of these deep-learning models to different real-world fluorescence microscopy data has, however, not yet been completely explored. The question arises whether such CNN-based denoising methods are structure- and noise-specific. Here, we apply transfer learning and fine-tuning strategies to assess the generalization capability of these methods to different domains, in particular super-resolved microscopy images of different biological structures, before and after knowledge transfer. We first provide four novel real-world microscopy datasets with different noise levels to train and evaluate the deep learning based denoising and super-resolution approaches. Secondly, we extensively investigate the performance of CNN-based denoising networks on data from different domains (i.e. different biological structures) by applying transfer learning strategies, such as direct transfer and fine-tuning. In the last step, we demonstrate that the fine-tuning approach is more advantageous than the conventional training of CNN-based denoising methods to avoid computational overhead.

Key words: Structured illumination microscopy; Super-resolution; Fluorescence microscopy; Deep learning; Convolutional neural networks; Denoising; Image restoration; Image reconstruction; Transfer learning; Fine-tuning

Introduction

In optical microscopy, super-resolution structured illumination microscopy (SR-SIM) plays a significant role in the field of biological and biomedical studies to analyze living cells and biological specimens with characteristic features below the resolution limit of classical microscopes (approx. 250 nm for high-end systems with oil-immersion objective lenses). Structures of interest include e.g. the internal organelles of mitochondria, cellular cytoskeleton, virus particles, or small vesicles [1, 2, 3, 4, 5]. SR-SIM is an important super-resolution approach to disentangle complex biological cellular structures with an up to twofold enhancement of spatial resolution beyond the diffraction limit. During the process of super-resolution imaging, SR-SIM involves the illumination of the biological sample with spatially patterned light following a sinusoidal intensity distribution. A series of raw images with typically 3 or 5 different phase positions and 3 angles of orientation of the illumination pattern is typically acquired [6]. Subsequently, frequency-domain based image reconstruction algorithms, e.g. implemented in software packages such as fairSIM [7], Open-SIM [8], and Python-based packages [9] are applied to a set

Compiled on: February 17, 2023.
Draft manuscript prepared by the author.

of raw SR-SIM images to generate the final twofold super-resolved images. SR-SIM has many advantages over other super-resolution methods e.g., it does not require special sample preparation, it permits the use of conventional fluorophores in multiple colors, simultaneously, and it allows for imaging at high speed and large fields-of-view (FOV) while being compatible with live cell samples by making efficient use of low illumination intensity levels [10, 11]. The conventional SR-SIM reconstruction algorithms have some limitations whenever the signal-to-noise level of the raw images is poor due to low fluorescence emission or short exposure times [12]. In general, in fluorescence microscopy weak emission due to, e.g. low labeling densities, high light scattering or absorption, or optical aberrations is often encountered [13, 14]. In the case of several super-resolution fluorescence microscopy methodologies, this leads to low SNR, and poor image reconstructions or artifacts. Nevertheless, SR-SIM imaging provides better resolution and optical sectioning abilities than confocal microscopy with a just minimally larger number of raw images to be acquired [15].

Recently, several deep-learning based methods have been developed to successfully reconstruct and denoise SR-SIM images by using noisy raw images or noisy reconstructed SR-SIM data [16, 17, 18, 19]. These deep learning based methods require a rather large number of images to train the underlying models and the sheer size and storage demand of such high-resolution reconstructed microscopic datasets is creating difficulties in their preparation, such that they are typically not publicly available. The very few open-source datasets that are available are mostly related to wide-field microscopy and contain a relatively small number of images. For instance, Zhang et al. [20] collected three wide-field microscopy datasets purely for the denoising tasks without providing high-resolution ground truth images obtained by SR-SIM technology. They used image averaging to generate ground truth data with high signal-to-noise ratio (SNR). Zhou et al. [21], on the other hand, published a dataset called "Widefield2SIM" using wide-field fluorescence microscopy. They captured 120 different fields-of-view (FOV) with 400 low SNR images for each FOV and generated high-resolution ground truth data using SR-SIM imaging technology. Qiao et al. [22] presented the "BioSR" dataset that consists of 2200 pairs of raw low-resolution (LR) and high-resolution (HR) data. Similarly, Hagen et al. [23] proposed a variety of datasets, which were collected by using wide-field and confocal microscopy. They captured various fluorescently labelled structures such as actin, mitochondria, membrane, and nuclei in the low and high SNR. However, their collection of datasets consists of considerably fewer images and does not contain any super-resolution SIM images.

Here, we present a series of datasets that are related to SR-SIM microscopy. These datasets cover two types of biological structures, tubulin filaments, and vesicles, with several fields-of-view for the denoising, super-resolution, and joint denoising and super-resolution tasks. In our datasets, noisy input and reference output images were obtained by using SR-SIM reconstruction algorithms. We also believe that our high-resolution SR-SIM datasets will be helpful for the research community to benchmark different deep-learning based denoising and super-resolution (SR) methods.

In particular, here we also evaluate the potential benefits of direct transfer and fine-tuning based on pretrained models by reconstructing datasets of biological structures different from the pre-trained models. Previously, we have shown that deep-learning based denoising methods work well for denoising and super-resolution tasks [19]. In [19] only one type of dataset with a specific structure and noise type was used to demonstrate the performance of deep-learning based denoising methods. There, we demonstrated that these methods are robust to different noise levels and SIM modes, e.g. varying pattern spacings at different illumination wavelengths. However, due to the limited amount of SR-SIM data available, we were not able to explore the techniques of knowledge transfer in the deep-learning methods. Therefore, here, we aim to answer the following questions: (1) If the model is trained on a specific biological structure with a specific type of noise, will it also generalize well to reconstructing another, different structure with another type of noise? and (2) Is the fine-tuning of the pretrained model (i.e., previously trained on one type of structure and noise) more useful than training the model from scratch? To answer these questions, we have conducted a series of experiments which we will discuss in later sections.

Materials and Methods

SR-SIM microscopy and sample preparation

The raw SR-SIM images for all the datasets were acquired using a DeltaVision OMX V4 (GE Healthcare, Chicago, IL, USA) 3D-SIM imaging system. To prepare the raw SR-SIM image data (datasets 1-3) of the tubulin cytoskeleton, U2OS cells were cultured in DMEM supplemented with 10%FBS and grown on round coverslips of 170 ± 5 μ m thickness (No. 1.5H). Cells were fixed with 4% PFA for 15 min., followed by PBS washes, and permeabilization with 0.5% Triton-X100 for 3 min. Another two rounds of PBS washes were done before blocking with 3% BSA. For immunolabeling of the tubulin microfilaments, cells were stained with anti-tubulin antibody (Invitrogen Cat. No. 322500) 1:400 for 2 hr at room temperature, followed by a PBS wash and one additional hour of incubation with Alexa 488-conjugated anti-mouse IgG 1:400. Afterwards the cells were then briefly washed with PBS before Vectashield was applied to embed the coverslip onto a standard microscopy glass slide for imaging. For the preparation of raw SR-SIM images with vesicle structures (dataset 4), U2OS cells were transfected with Lipofectamine 3000 according to the manufacturers protocol (Thermofisher Cat. No. L3000-001) together with a plasmid expressing the vesicular Lamp1 protein fused to the fluorescent protein mScarlet. After 24 hr transfection the cells were fixed with 4% PFA for 10 min., followed by PBS washes and Vectashield mounting prior to imaging. The vesicular structures represent lysosomes.

Dataset preprocessing and image reconstruction

Dataset 1 is a raw dataset without any image processing or reconstruction applied to the raw SIM images. The images in the datasets 2 and 3 are reconstructed by using the open-source fairSIM reconstruction algorithm as shown in Figure 1. FairSIM implements a single-slice (2D) SR-SIM image reconstruction algorithm [7]. It works in three steps: parameter estimation, reconstruction, and filtering. The mathematical and algorithmic details of the fairSIM reconstruction method are explained in the original publication [7]. A synthetic optical transfer function, with NA = 1.4, λ = 525 nm, a = 0.31 (a is a compensation parameter,see [7, 24]) is used. For the tubulin samples, a background of 500 counts per pixel is subtracted during the reconstruction process. SR-SIM reconstruction parameters (pattern orientation, global phase, etc.) are automatically determined by fairSIM's standard, iterative cross-correlation approach. Filter parameters are set to a generalized Wiener filter with a strength of w = 0.05, apodization is set at 1.9× the resolution limit with a *bend* of 0.8. A notch-style filter implemented as *OTF attenuation* with a strength of 0.995 and a FWHM of 1.2 μm^{-1} is used. The full information about the functionality of these parameters is explained in [7],

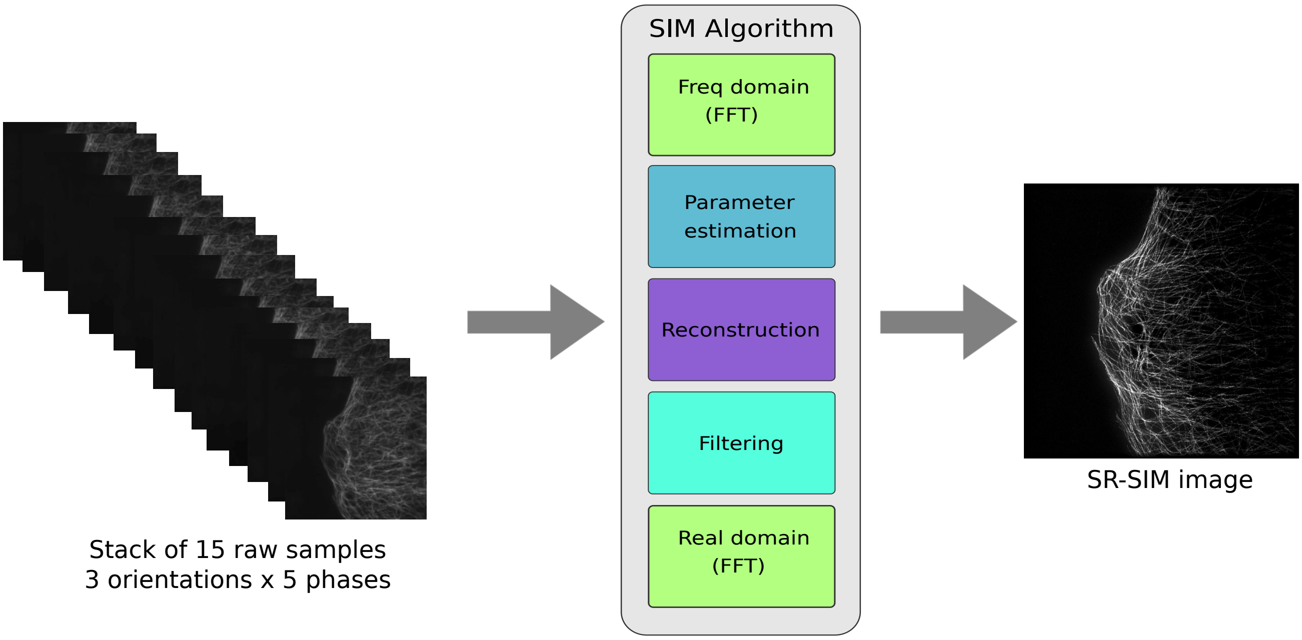

Figure 1. The architecture of the image reconstruction algorithm which is used to generate the reconstructed SR-SIM images. Both reconstruction algorithms (i.e., fairSIM and Softworx software) that were used in this work are based on the SIM algorithm. During the reconstruction of SR-SIM images, a stack of 15 raw SIM samples of size 512 × 512 are propagated into the SIM algorithm software which generates the high-resolution SR-SIM image of size 1024 × 1024 pixels.

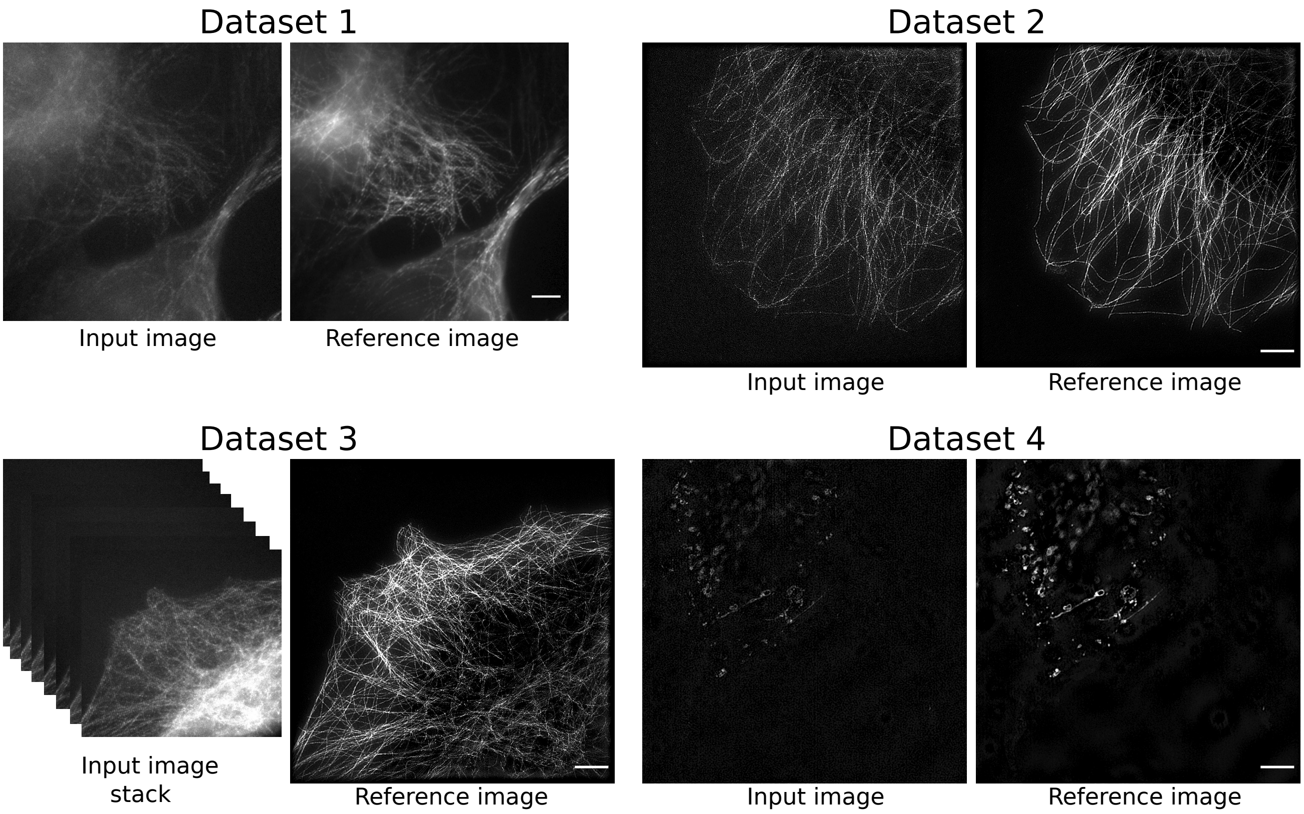

Figure 2. Dataset 1 consists of raw SR-SIM data. The input-reference pairs represent each phase and orientation from different noise levels of tubulin filaments. The size of the input and reference images is 512 × 512 pixels in dataset 1. Scale bar: 8 µm. Dataset 2 contains the reconstructed SR-SIM images of tubulin structure, and each input-reference pair has a size of 1024 × 1024 pixels. Scale bar: 4 µm. In dataset 3, raw SR-SIM data in the form of an image stack (i.e., 15 × 512 × 512 pixels) are used as input samples, whereas the reconstructed SR-SIM images (1024 × 1024 pixels) are used as the corresponding reference samples for joint denoising and super-resolution. Scale bar of input and reference images: 8 and 4 µm. Dataset 4 is based on the reconstructed SR-SIM images of size 1024 × 1024 pixels of fluorescently labeled vesicles.

and the general guide for using SIM reconstruction parameters is discussed in [24]. The code used to generate the samples of dataset 2 is available at [25]. The samples in dataset 4 (the vLamp1-mScarlet expressing cell) were reconstructed using the commerical software 'SoftWorx v7' (GE Healthcare) for 3D-SIM.

Table 1. Description of all the datasets

Dataset	Dataset 1	Dataset 2	Dataset 3	Dataset 4
Structure	tubulin filaments	tubulin filaments	tubulin filaments	vesicles
Microscope	SR-SIM microscopy	SR-SIM microscopy	SR-SIM microscopy	SR-SIM microscopy
Pixel Size, nm	80	40	80 (input) / 40 (ref.)	40
Number of timestamps	200	200	200	max: 99, min: 15
Fields-of-view	101	101	101	175
Input image size, pixels	512x512	1024x1024	15x512x512	1024x1024
Output/Reference image size, pixels	512x512	1024x1024	1024x1024	1024x1024
No of samples	303000	2525	2525	7284
Reconstruction	raw data	fairSIM	fairSIM	SoftWORX

Description of datasets

Dataset 1

Dataset 1 contains around 101 fields-of-view (FOV) of tubulin filaments and each FOV further consists of 3000 images (all in one TIF file for each FOV). In each FOV, a stack of 15 raw SIM images represents the combination of 5 orientations and 3 phases, whereas this full stack is repeatedly captured for 200 timestamps. The signal-to-noise ratio decreases with every timestamp. Thus, dataset 1 contains a total of 303000 raw SIM images of size 512×512 (width $\times$height) pixels with 15 combinations of phase and orientation at each timestamp. Each timestamp in the raw images lasts approximately 25 ms. Each pixel contains a single 16-bit integer value captured by the microscope's camera, which is calibrated to provide a signal linear in photon count for each pixel. This is typical for scientific camera systems, but dissimilar to standard image processing, where often gamma mapping is applied between light intensity and pixel values. This dataset can be used mainly for image denoising tasks (from noisy input to output with higher signal-to-noise ratio). Therefore, the images from timestamp 1 are intended as output images (ground truth), and the rest of the images can be categorized as input images as shown in Figure 2.

Dataset 2

We constructed dataset 2 by applying the fairSIM reconstruction algorithm to the raw SIM images from dataset 1. Dataset 2 contains pairs of SR-SIM reconstructed noisy input and high-resolution output images. The process of the generation of input and output SIM images from the raw SIM images is shown in Figure 1. The 15 raw SIM images of size 512×512 (width $\times$ height) pixels of different phases and orientations are propagated into the fairSIM algorithm to reconstruct the SR-SIM images of size 1024×1024 (width $\times$ height) pixels. During the formation of this dataset, the raw samples from timestamp 1 were used to generate the output images (i.e., reference images). We use the term 'reference images' instead of 'ground truth images' because of the SIM reconstruction artifacts in the output images of this dataset. The input samples were reconstructed by using the raw SIM samples from timestamps 176 – 200. Therefore, dataset 2 is composed of 2525 reconstructed pairs of SR-SIM images with a size of 1024×1024 (width $\times$ height) pixels extracted from the 101 FOV. In addition to these 2525 image pairs, for the last 20 FOV we also include image pairs in the data collection where the noisy input is from timestamps 76 – 100, 126 – 150 and 176 – 200. This additional data can be used to create test sets to evaluate the robustness of denoising networks for different noise levels. In our previous work [19], we denoted data from timestamp 26 – 50 as noise level 1. Similarly, noise level 2, 3, and 4 correspond to the data from the timestamps 76 – 100, 126 – 150 and 176 – 200 (shown in Figure 3). Overall, dataset 2 is generated mainly for the denoising of SR-SIM images.

Dataset 3

Dataset 3 was created mainly for joint denoising and super-resolution tasks. The composition of this dataset is based on a mixture of images from dataset 1 and 2. This dataset is formed by combining the raw noisy SIM samples of noise level 4 from dataset 1 with the high-resolution reconstructed reference SR-SIM samples from dataset 2. The input samples consist of a stack of noisy raw SIM images; each single image in the stack represents different illumination phases and orientations. The reference samples are the reconstructed high-resolution SR-SIM images. The size and dimension of each input sample is $15 \times 512 \times 512$ (depth $\times$ width $\times$ height) pixels. The size of the output or reference sample is 1024×1024 (width $\times$ height) pixels. The input and reference images in this dataset contain tubulin filaments as biological structure.

Dataset 4

Dataset 4 is composed of 3D-SR-SIM images showing vesicles in U2OS cells (i.e. typically round intracellular droplets) and can be used mainly for denoising tasks. This data is composed of several z-stack slices and each slice is captured for different timestamps. The raw data of this dataset contains 175 FOV, and each field-of-view is recorded for a different number of timestamps.[1] We acquired a total of 7284 pairs of input and output SR-SIM images. The size of the input and reference images is 1024×1024 (width $\times$ height) pixels. In each image pair, the output/reference image is taken from timestamp 1 of the respective FOV, whereas the input images are taken from the following time stamps and exhibit therefore a lower signal-to-noise ratio. The reconstruction of the original raw SIM images into the SR-SIM images in this dataset was performed by using the softWoRx v7 software (GE Healthcare).

Data partitioning

In our experiments, in the first three datasets, the images of the last 20 FOV were used as test samples, and the remaining 81 FOV were used for the training set. The training set of dataset 2 is therefore composed of 2025 samples (image pairs) from 81 FOV and the test set is composed of 500 samples from 20 FOV. Similarly, in dataset 4 we used 5562 samples of 121 FOV for the training set and 1380 samples from the 46 FOV for the test set and the remaining 8 FOV were discarded because their reference images contain only noise without any meaningful structure. Datasets 1, 2, and 4 can only be used for denoising

1 This is a slight simplification: Originally, nine different fields-of-view were recorded, each at a varying number of depth levels (z-planes). However, the recorded structures in the different z planes are very different from each other, so we categorize them here as separate FOV. The file names in the dataset contain also indices for these original fields-of-view and for the respective z-plane.

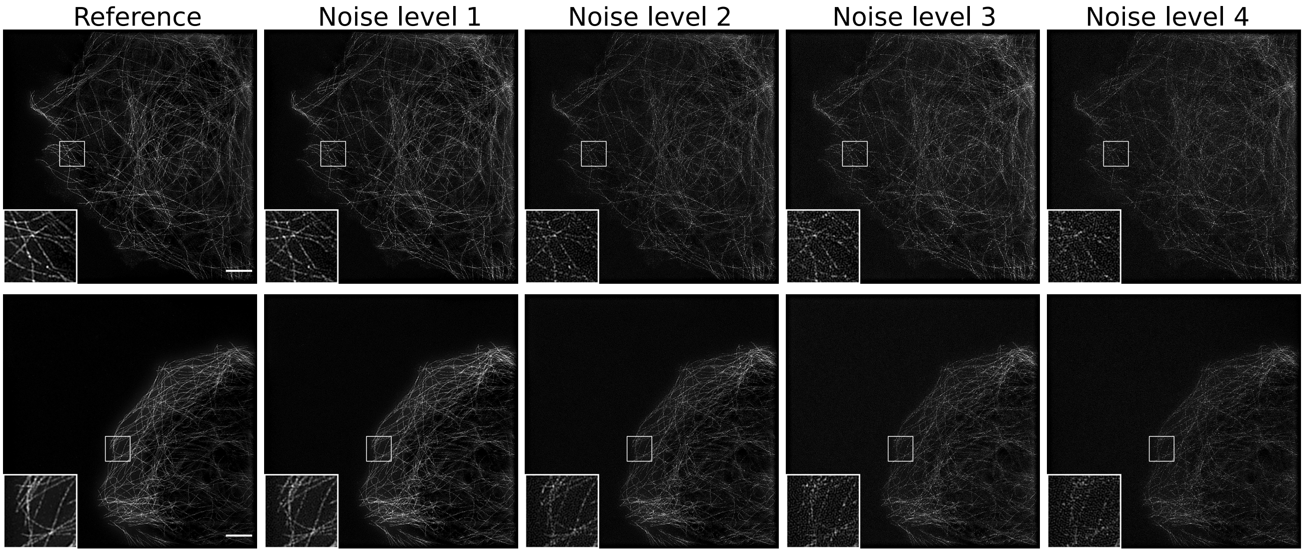

Figure 3. SR-SIM images from two different FOV, each shown at several noise levels. Each noise level corresponds to a different range of timestamps, e.g., images of noise level 4 are taken at the last timestamps and contain a high level of noise. Similarly, images of noise level 1 represent early timestamps with very low noise. The reference image is recorded at timestamp 0 and has the highest signal-to-noise ratio.

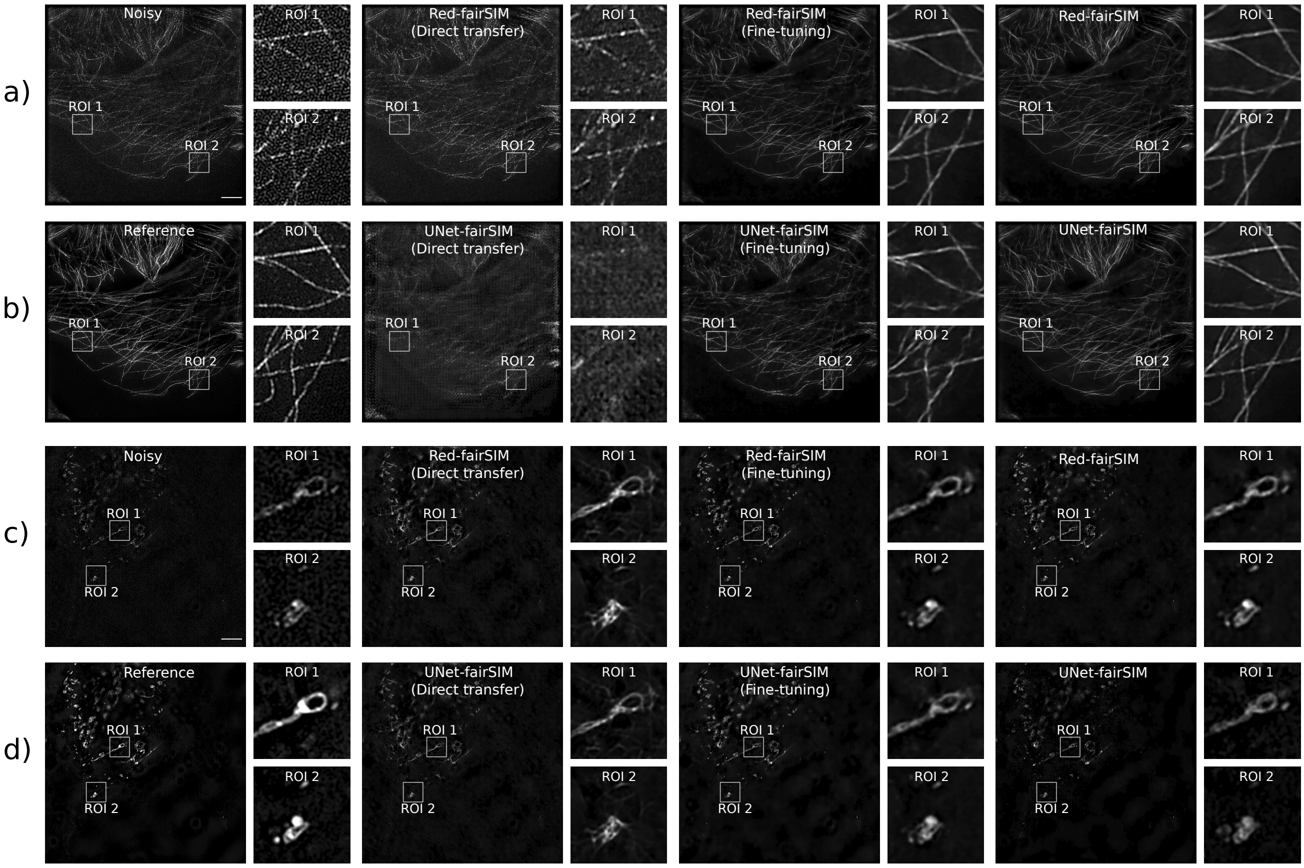

Figure 4. Results of test samples from datasets 2 and 4 are shown in this figure. The first and second row (**a** and **b**) show the outcome of dataset 2 with different architectures, whereas, the test samples with the vesicle structure from dataset 4 are displayed in the third and fourth row (**c** and **d**). The row **a** in this figure first contains the noisy SR-SIM image along with the denoised images of Red-fairSIM (direct transfer), i.e. model initially pretrained on vesicle structure, Red-fairSIM (fine-tuning), i.e. retraining of few layers of model initially pretrained on vesicle structure, and Red-fairSIM method, i.e. model trained on tubulin filaments from scratch. The second row **b** consists of the reference image together with the results of direct transfer, fine-tuning as well as the conventional training of the UNet-fairSIM algorithm. Similarly, rows **c** and **d** show the results of Red-fairSIM and UNet-fairSIM for all the strategies for dataset 4. The second, fourth, sixth and eighth column depict the cropped and zoomed regions of interest (ROIs) from the full-size SR-SIM images. The cropped ROIs of size 100 pixels $\times$ 100 pixels were upsampled to 300 pixels $\times$ 300 pixels using bicubic interpolation for demonstration purposes. Scale bar: 4 µm.

Table 2. Mean PSNR and SSIM values along with standard deviations (STD) of all experiments calculated on the noisy test images of datasets 2 and 4 (for dataset 2, noise level 4 was used). These values are calculated relative to the reference images, i.e. SR-SIM images reconstructed with fairSIM from raw SIM images with the highest signal-to-noise ratio. In the row entitled "fairSIM", PSNR and SSIM values are calculated for the direct fairSIM reconstruction of the noisy images. "Red-fairSIM" and "UNet-fairSIM" represent the methods in which the denoised test images are generated by the respective convolutional neural networks (CNNs) after conventional training from scratch. In the direct transfer rows, the mean PSNR and SSIM values in the column "dataset 2" are calculated for CNNs which are initially trained on dataset 4 and afterwards tested with test samples from dataset 2. For the column "dataset 4", this is the other way round. The same principle holds for the rows for fine-tuning, only that the CNNs are fine-tuned on the respective dataset before testing.

	Mean PSNR (STD) and SSIM (STD) values of test data			
	**dataset =2**		**dataset =4**	
	PSNR (STD)	SSIM (STD)	PSNR (STD)	SSIM (STD)
fairSIM	23.61 (1.54)	0.29 (0.07)	35.10 (2.71)	0.86 (0.03)
Red-fairSIM	27.97 (2.01)	0.71 (0.09)	38.43 (1.45)	0.89 (0.01)
Direct transfer (Red-fairSIM)	23.31 (1.68)	0.41 (0.07)	33.89 (1.93)	0.81 (0.03)
Fine-tuning (Red-fairSIM)	27.90 (2.14)	0.70 (0.09)	38.30 (1.60)	0.88 (0.02)
UNet-fairSIM	26.80 (1.65)	0.68 (0.10)	37.45 (1.79)	0.88 (0.02)
Direct transfer (UNet-fairSIM)	24.69 (1.54)	0.46 (0.05)	34.47 (2.24)	0.83 (0.04)
Fine-tuning (UNet-fairSIM)	28.02 (1.97)	0.71 (0.07)	38.35 (1.44)	0.89 (0.01)

tasks, however, the data from dataset 3 can be used for both joint denoising and super-resolution. To reproduce the results of this work, we share the source code on the GitHub repository [26] and the trained models on [27].

Transfer learning and fine-tuning

In the transfer learning technique in general, the knowledge of a trained model is transferred from one related task that has been learned to another task from the same domain [28]. The weights of the pretrained model that is trained on specific data and for a specific task are transferred to the different data but related task [29, 30]. The utilization of transfer learning in the deep learning field is very useful in reducing computational power and time complexity [31]. Furthermore, transfer learning is also very helpful when it comes to large dataset requirements [32]. The simplest variant of transfer learning is to directly apply a trained model to another task without retraining. In this contribution, we train the Red-fairSIM method [19] on dataset 2 and then evaluate the test samples of dataset 4 with the pretrained model and vice versa. Similarly, we train the UNet-fairSIM algorithm [19] on dataset 4 and then try to denoise the test images of dataset 2 and vice versa. This approach of training a network on one dataset and later testing with another dataset is called "direct transfer" here.

Furthermore, within the field of transfer learning, fine-tuning is one of the most important strategies for transferring the model knowledge from one domain into another domain [33]. In the fine-tuning approach, the weights of some layers of pretrained models are preserved and the rest of the layers are retrained or fined-tuned [34].

In our previous studies [19], we already showed that deep-learning-based models are robust to different noise levels and microscopic settings. However, here we want to answer the following questions: 1. are the pretrained deep learning-based denoising models robust to different types of structures and noise, and 2. how do they compare to training strategies such as fine-tuning and conventional training? To investigate these questions, we thoroughly performed direct transfer and fine-tuning on different pretrained models.

Here, we used the Red-fairSIM and UNet-fairSIM architectures from our previous work [19]. Red-fairSIM is based on the residual-encoder-decoder network (RED-Net) [35]. In the fine-tuning approach for Red-fairSIM, we retrained the first and last 5 layers of the model instead of all 30 layers. In other words, we retrain approx. 295K trainable parameters out of more than 1 million parameters of the Red-fairSIM network. The other architecture used in the study is a combination of UNet with the fairSIM algorithm. UNet is also based on several encoder and decoder blocks [36]. In the fine-tuning of UNet-fairSIM, we simply retrained the first and last two encoding and decoding blocks which contains approx. 3.7 million learnable parameters out of more than 33 million parameters. In the first step, we trained these two CNN-based denoising algorithms from scratch on the datasets 2 and 4 separately for 100 epochs. The mean square error (MSE) loss function was used to carry out these and all other training runs in this work. In the next step, we applied direct transfer by propagating the test samples from the respective dataset that was not used for training through the pretrained models. During direct transfer, the test images of dataset 2 (i.e., tubulin filaments) were propagated through the pretrained model which was trained on dataset 4 (i.e., vesicles structure) and vice versa. In the third step, we fine-tuned both pretrained models with training data from the respective dataset that was not used for the initial training. 30 training epochs were used for fine-tuning. The fine-tuned models were afterwards evaluated on the test samples from the dataset that was used during the fine-tuning process. In the fine-tuning of the pretrained models, we initially preserved varying numbers of layers. However, we noticed that we can achieve the best results by fine-tuning very few trainable parameters for 30 Epochs. In the final step, we compared the results of conventional training with the direct transfer and fine-tuning strategies visually as well as in terms of the peak-signal-to-noise ratio (PSNR) [37] and structural similarity index measurement (SSIM) [38] values.

Results and discussion

We collected four datasets for denoising and super-resolution tasks. The overview and the full characteristics of these datasets are shown in Table 1. In our previous studies [19], we

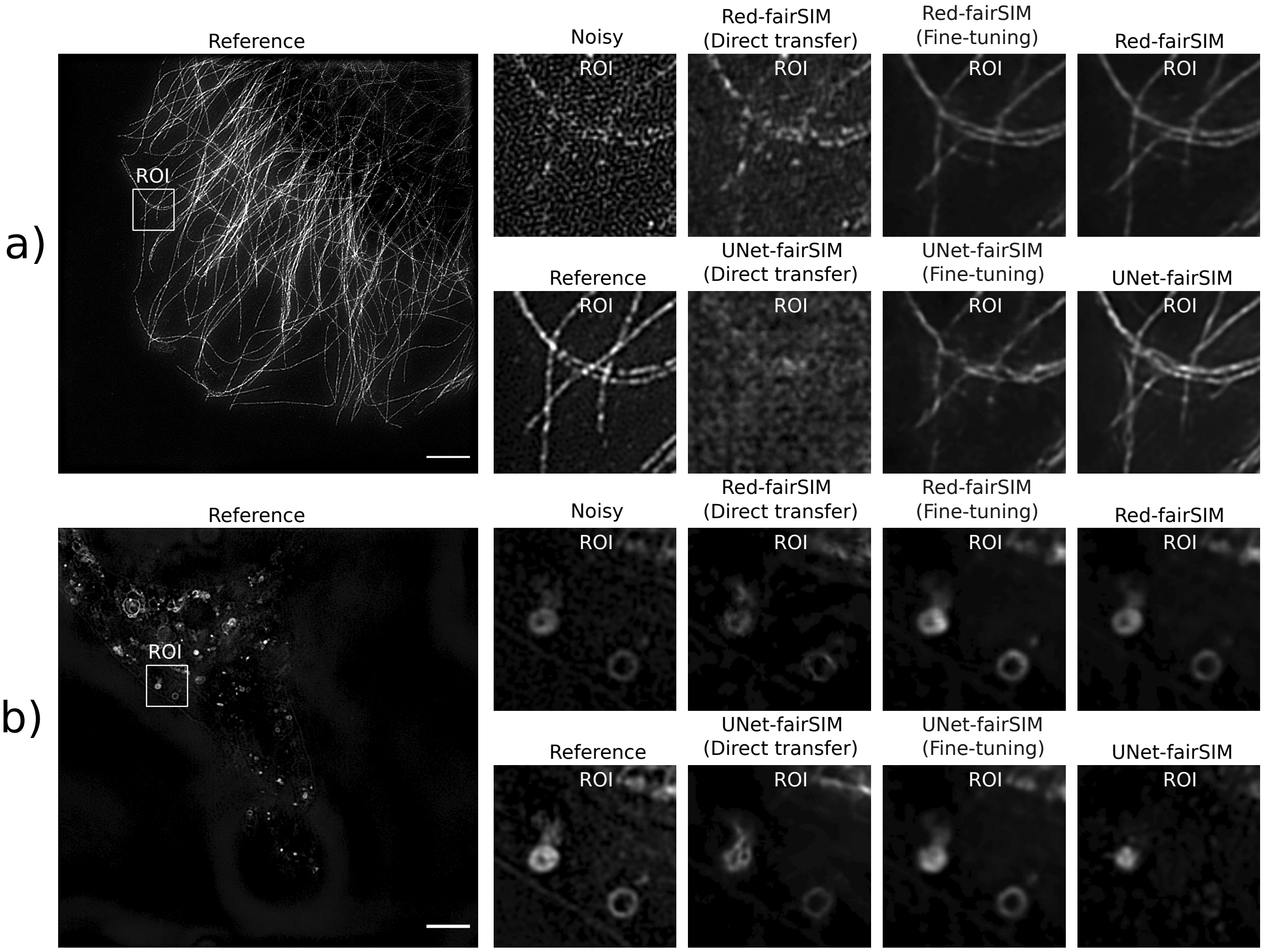

Figure 5. Two more test samples from datasets 2 and 4 are shown in this figure. Both rows **a** and **b** contain the reference SR-SIM image along with the resultant denoised ROIs which are extracted from the full-size denoised images of all the methods used in this work. The extracted ROIs are upsampled from 100 pixels × 100 pixels to 300 pixels × 300 pixels using bicubic interpolation. Scale bar: 4 µm.

already used the first three datasets for the tasks of denoising and super-resolution. Here, we mainly focused on two transfer learning strategies, direct transfer and fine-tuning of the pretrained models. The Red-fairSIM and UNet-fairSIM models were trained on the datasets 2 and 4, separately. The images of dataset 2 contain the tubulin structure along with mixed Poisson-Gaussian (MPG) noise and SR-SIM reconstruction artifacts. The images in dataset 4 contain the vesicles structure together with MPG noise and honeycomb pattern artifacts, which arise as raw data carrying predominantly Poisson-Noise is subjected to the frequency-based SR-SIM reconstruction algorithm, which then gives rise to reconstruction artifacts [14]. To compare the performance of conventional training of deep learning based denoising methods with direct transfer and fine-tuning, we first trained both models with datasets 2 and 4, independently. In the next step we evaluated the direct transfer strategy by using these pretrained models. In direct transfer, the model initially trained with dataset 2 is tested with the test samples of an alternative dataset (i.e., dataset 4). Similarly, in the assessment of the fine-tuning strategy, we retrained the first and last few layers of the pretrained models with the alternative data to see the improvement in the generalization power of the pretrained models.

The resulting denoised images obtained by direct transfer from models trained on vesicle images (dataset 4) to test images containing tubulin structures (dataset 2) are displayed within rows **a**, **b** of Figure 4 and row **a** of Figure 5. These images clearly show that the model trained on a specific type of noise and structure (i.e., vesicle images with honey comb pattern noise) is not able to produce a refined denoised image of another structure (i.e., tubulin filaments with MPG noise). Similarly, the models initially trained on tubulin filaments from dataset 2 are not able to properly produce the denoised images of dataset 4 with different structure and noise (see rows **c**, **d** of Figure 4 and row **d** of Figure 5). It can be noted that the pretrained models try to replicate the filamentous structure of tubulin in the vesicle data (very prominent in rows **c** and **d** of Figure 4). This is a clear indication that the CNN-based denoising models are not robust against different types of noise and structure. However, the outcomes of the fine-tuning approach are promising as seen in Figures 4 and 5 as well. The ROIs 1 and 2 in all rows of Figure 4 and the ROI in all rows of Figure 5 show that the results of the fine-tuning strategy are comparable with denoised images from the conventional Red-fairSIM and UNet-fairSIM approaches (i.e., trained with one type of dataset from scratch). The overall comparison of all the approaches in Figures 4 and 5 clearly demonstrates that the fine-tuning method is crucial for CNN-based denoising algorithms in the case of changes in structure or noise types. Thus, fine-tuning is inevitably required to profit from knowledge transfer.

Table 2 lists the average PSNR and SSIM values of the test images of the datasets 2 and 4 calculated by all the training and testing strategies of Red-fairSIM and UNet-fairSIM. Table 2 clearly points out the decline in the average PSNR and

SSIM values on both datasets 2 and 4 after the application of direct transfer. However, the average PSNR and SSIM values show considerable improvement after fine-tuning. The average PSNR and SSIM values of the fine-tuned models in Table 2 are very close to the models that were trained from scratch (partly slightly better, partly slightly worse). Because the fine-tuning method retrain less trainable parameters instead of the whole set of model parameters compared to conventional training. In this study, we retrain approx. $296K$ parameters out of more than 1 million parameters of Red-fairSIM algorithm, whereas, in UNet-fairSIM out of more than 33 million trainable parameters only 3.7 million parameters were retrained. It is therefore advantageous regarding computational requirements or memory consumption. Moreover, we were able to achieve comparable results to conventional training after only 30 epochs compared to 100 epochs. These advantages are very beneficial whenever computational resources are limited (or the amount of training data for the new task is small). In summary, in this work, we provided four novel datasets for testing denoising and super-resolution image reconstruction strategies. These datasets cover a large number of samples, different noise levels, a wide range of FOV as well as structural complexity and reconstructed twofold super-resolved images. High-quality data from the real-world domain of microscopy is more significant for benchmarking and for the evaluation of current and forthcoming deep learning based denoising and super-resolution methods than synthetic data. Furthermore, we showed that knowledge transfer in the area of CNN-based denoising approaches has limitations when it comes to different types of noise and structure. However, the approach of fine-tuning empowers the CNNs used for denoising to generalize well to other noise types and structures. We noted that the strategy of fine-tuning of pretrained models has advantages over conventional training of models from scratch especially when the difference between the task domains is not huge.

Potential implications

The implementation of different deep learning methods requires a large quantity of images in order to train the underlying models. We believe that our published datasets will help the research community to develop new deep learning based methods and evaluate the existing methods by either training from scratch or by applying fine-tuning. These datasets can be especially used for image denoising and super-resolution tasks.

Data and code Availability

All the raw and reconstructed datasets which are discussed in this article are available on GigaDB ["link will be uploaded after the allocation of space from Giga Database"]. All the datasets are distributed under the Creative Commons CCO waiver, with a request for attribution. The CCO permits the unrestricted reuse, distribution and reproduction, provided the original work is properly cited. Similarly, all the codes which are used during this work are publicly available [26].

Declarations

Abbreviations

SIM: structured illumination microscopy; SR-SIM: super-resolution structured illumination microscopy; NA: numerical aperture; PSNR: peak-signal-to-noise ratio; SSIM: structural similarity index measurement; MSE: mean square error; SNR: signal-to-noise ratio; W2S: wide-field2SIM; fairSIM: free analysis and interactive reconstruction for structured illumination microscopy.

Competing Interests

The authors declare no competing interests.

Funding

This work was funded by the BMBF (German Federal Ministry of Education and Research) via grant 01IS18041C (consortium project “ITS.ML: Intelligent technical systems via machine learning”). T.-C.W. and T.H. were supported by funding from the European Union’s Horizon 2020 research and innovation program under the Marie Sklodowska-Curie Grant Agreements No. 642157, project “TOLLerant”, and No. 766181. project "DeLIVER". T.H. also acknowledges funding by the Deutsche Forschungsgemeinschaft (DFG, German Science Foundation)—project number 415832635.

Author’s Contributions

Z.H.S. carried out the preprocessing of datasets for the machine learning work, created the figures, and wrote a large part of the manuscript. D.T. conducted experiments to find the optimal hyperparameters. T.-C.W. recorded the raw SIM images of dataset 1. M.M. supported Z.H.S in the reconstruction of data from datasets 2 and 3 with fairSIM. W.H. recorded and reconstructed the images of dataset 4. T.H., and W.S. supervised the research. In addition, they discussed the experimental results together with Z.H.S. and M.M., and W.H. All authors discussed and agreed on the final manuscript.

Acknowledgements

The authors would like to thank Dr. Matthias Fricke from the Center for Applied Data Science (CfADS) at Bielefeld University of Applied Sciences for providing access to their GPU compute cluster. We would also like to thank Dr. Olaf Kaczmarek and Markus Klappenback for providing access to the GPU compute cluster at Bielefeld University.

References

1. Hirvonen LM, Wicker K, Mandula O, Heintzmann R. Structured illumination microscopy of a living cell. European Biophysics Journal 2009;38(6):807–812.
2. Hell SW, Sahl SJ, Bates M, Zhuang X, Heintzmann R, Booth MJ, et al. The 2015 super-resolution microscopy roadmap. Journal of Physics D: Applied Physics 2015;48(44):443001.
3. Heintzmann R, Huser T. Super-resolution structured illumination microscopy. Chemical reviews 2017;117(23):13890–13908.
4. Demmerle J, Innocent C, North AJ, Ball G, Müller M, Miron E, et al. Strategic and practical guidelines for successful structured illumination microscopy. Nature protocols 2017;12(5):988–1010.
5. Schermelleh L, Ferrand A, Huser T, Eggeling C, Sauer M, Biehlmaier O, et al. Super-resolution microscopy demystified. Nature cell biology 2019;21(1):72–84.
6. Gustafsson MG. Surpassing the lateral resolution limit by a factor of two using structured illumination microscopy. Journal of microscopy 2000;198(2):82–87.

7. Müller M, Mönkemöller V, Hennig S, Hübner W, Huser T. Open-source image reconstruction of super-resolution structured illumination microscopy data in ImageJ. Nature communications 2016;7(1):1–6.
8. Lal A, Shan C, Xi P. Structured illumination microscopy image reconstruction algorithm. IEEE Journal of Selected Topics in Quantum Electronics 2016;22(4):50–63.
9. Brown PT, Kruithoff R, Seedorf GJ, Shepherd DP. Multicolor structured illumination microscopy and quantitative control of polychromatic light with a digital micromirror device. Biomedical Optics Express 2021;12(6):3700–3716.
10. Ströhl F, Kaminski CF. Frontiers in structured illumination microscopy. Optica 2016;3(6):667–677.
11. Zheng X, Zhou J, Wang L, Wang M, Wu W, Chen J, et al. Current challenges and solutions of super-resolution structured illumination microscopy. APL Photonics 2021;6(2):020901.
12. Huang X, Fan J, Li L, Liu H, Wu R, Wu Y, et al. Fast, long-term, super-resolution imaging with Hessian structured illumination microscopy. Nature biotechnology 2018;36(5):451–459.
13. Hoffman DP, Betzig E. Tiled reconstruction improves structured illumination microscopy. BioRxiv 2020;.
14. Smith CS, Slotman JA, Schermelleh L, Chakrova N, Hari S, Vos Y, et al. Structured illumination microscopy with noise-controlled image reconstructions. Nature methods 2021;18(7):821–828.
15. Gustafsson MG, Shao L, Carlton PM, Wang CR, Golubovskaya IN, Cande WZ, et al. Three-dimensional resolution doubling in wide-field fluorescence microscopy by structured illumination. Biophysical journal 2008;94(12):4957–4970.
16. Shah ZH, Müller M, Hammer B, Huser T, Schenck W. Impact of different loss functions on denoising of microscopic images. In: 2022 International Joint Conference on Neural Networks (IJCNN) IEEE; 2022. p. 1–10.
17. Jin L, Liu B, Zhao F, Hahn S, Dong B, Song R, et al. Deep learning enables structured illumination microscopy with low light levels and enhanced speed. Nature communications 2020;11(1):1–7.
18. Chen X, Li B, Jiang S, Zhang T, Zhang X, Qin P, et al. Accelerated Phase Shifting for Structured Illumination Microscopy based on Deep Learning. IEEE Transactions on Computational Imaging 2021;7:700–712.
19. Shah ZH, Müller M, Wang TC, Scheidig PM, Schneider A, Schüttpelz M, et al. Deep-learning based denoising and reconstruction of super-resolution structured illumination microscopy images. Photonics Research 2021;9(5):B168–B181.
20. Zhang Y, Zhu Y, Nichols E, Wang Q, Zhang S, Smith C, et al. A poisson-gaussian denoising dataset with real fluorescence microscopy images. In: Proceedings of the IEEE/CVF Conference on Computer Vision and Pattern Recognition; 2019. p. 11710–11718.
21. Zhou R, Helou ME, Sage D, Laroche T, Seitz A, Süsstrunk S. W2S: microscopy data with joint denoising and super-resolution for widefield to SIM mapping. In: European Conference on Computer Vision Springer; 2020. p. 474–491.
22. Qiao C, Li D, Guo Y, Liu C, Jiang T, Dai Q, et al. Evaluation and development of deep neural networks for image super-resolution in optical microscopy. Nature Methods 2021;18(2):194–202.
23. Hagen GM, Bendesky J, Machado R, Nguyen TA, Kumar T, Ventura J. Fluorescence microscopy datasets for training deep neural networks. GigaScience 2021;10(5):giab032.
24. Karras C, Smedh M, Förster R, Deschout H, Fernandez-Rodriguez J, Heintzmann R. Successful optimization of reconstruction parameters in structured illumination microscopy–a practical guide. Optics Communications 2019;436:69–75.
25. Mueller M, Free Analysis and Interactive Reconstruction for Structured Illumination Microscopy. GitHub; 2016. https://github.com/fairSIM/fairSIM.git.
26. Shah ZH, Evaluation of knowledge transfer for the denoising of super-resolution structured illumination microscopy data. GitHub; 2023. https://github.com/ZafranShah/Denoising_and_fine_tuning_of_SR-SIM_data.
27. Shah ZH, Evaluation of knowledge transfer for the denoising of super-resolution structured illumination microscopy data. zenodo; 2023. https://doi.org/10.5281/zenodo.7626173.
28. Ching JY, Wong AKC, Chan KCC. Class-dependent discretization for inductive learning from continuous and mixed-mode data. IEEE Transactions on Pattern Analysis and Machine Intelligence 1995;17(7):641–651.
29. Hussain M, Bird JJ, Faria DR. A study on cnn transfer learning for image classification. In: UK Workshop on computational Intelligence Springer; 2018. p. 191–202.
30. Tan C, Sun F, Kong T, Zhang W, Yang C, Liu C. A survey on deep transfer learning. In: International conference on artificial neural networks Springer; 2018. p. 270–279.
31. Bengio Y. Deep learning of representations for unsupervised and transfer learning. In: Proceedings of ICML workshop on unsupervised and transfer learning JMLR Workshop and Conference Proceedings; 2012. p. 17–36.
32. Ng HW, Nguyen VD, Vonikakis V, Winkler S. Deep learning for emotion recognition on small datasets using transfer learning. In: Proceedings of the 2015 ACM on international conference on multimodal interaction; 2015. p. 443–449.
33. Nogueira K, Penatti OA, Dos Santos JA. Towards better exploiting convolutional neural networks for remote sensing scene classification. Pattern Recognition 2017;61:539–556.
34. Yosinski J, Clune J, Bengio Y, Lipson H. How transferable are features in deep neural networks? Advances in neural information processing systems 2014;27.
35. Mao X, Shen C, Yang YB. Image restoration using very deep convolutional encoder-decoder networks with symmetric skip connections. Advances in neural information processing systems 2016;29.
36. Ronneberger O, Fischer P, Brox T. U-net: Convolutional networks for biomedical image segmentation. In: International Conference on Medical image computing and computer-assisted intervention Springer; 2015. p. 234–241.
37. Hore A, Ziou D. Image quality metrics: PSNR vs. SSIM. In: 2010 20th international conference on pattern recognition IEEE; 2010. p. 2366–2369.
38. Setiadi DRIM. PSNR vs SSIM: imperceptibility quality assessment for image steganography. Multimedia Tools and Applications 2021;80(6):8423–8444.
