# Supplementary material for: Evaluation of Swin Transformer and knowledge transfer for denoising of super-resolution structured illumination microscopy data
Source: Gigascience. 2024 Jan 13;13:giad109. doi: 10.1093/gigascience/giad109 (PMC10787368; doi:10.1093/gigascience/giad109)
Supplement: giad109_GIGA-D-23-00044_Revision_1 [file giad109_giga-d-23-00044_revision_1.pdf]

GigaScience

Evaluation of Swin Transformer and knowledge transfer for denoising of super-resolution structured illumination microscopy data

--Manuscript Draft--

**Manuscript Number:**	GIGA-D-23-00044R1
**Full Title:**	Evaluation of Swin Transformer and knowledge transfer for denoising of super-resolution structured illumination microscopy data
**Article Type:**	Research
**Funding Information:**	Bundesministerium für Bildung und Forschung (01IS18041C) — Mr. Wolfram Schenck<br>H2020 Marie Skłodowska-Curie Actions (642157) — Mr. Thomas Huser<br>Deutsche Forschungsgemeinschaft (415832635) — Mr. Thomas Huser
**Abstract:**	In recent years, convolutional neural network (CNN)-based methods have shown remarkable performance in the denoising and reconstruction of super-resolved structured illumination microscopy (SR-SIM) data. Therefore, CNN-based architectures have been the main focus of existing studies. Recently, however, an alternative and highly competitive deep learning architecture, Swin Transformer, has been proposed for image restoration tasks. In this work, we present SwinT-fairSIM, a novel method for restoring SR-SIM images with low signal-to-noise ratio (SNR) based on Swin Transformer. The experimental results show that SwinT-fairSIM outperforms previous CNN-based denoising methods. Furthermore, the generalization capabilities of deep learning methods for image restoration tasks on real fluorescence microscopy data have not been fully explored yet, i.e., the extent to which trained artificial neural networks are limited to specific types of cell structures and noise. Therefore, as a second contribution, we benchmark two types of transfer learning, i.e., direct transfer and fine-tuning, in combination with SwinT-fairSIM and two CNN-based methods for denoising SR-SIM data. Direct transfer does not prove to be a viable strategy, but fine-tuning achieves results comparable to conventional training from scratch, while saving computational time and potentially reducing the amount of required training data. As a third contribution, we publish four datasets of raw SIM images and already reconstructed SR-SIM images. These datasets cover two different types of cell structures, tubulin filaments and vesicle structures. Different noise levels are available for the tubulin filaments. These datasets are structured in such a way that they can be easily used by the research community for research on denoising, super-resolution, and transfer learning strategies.
**Corresponding Author:**	Zafran Hussain Shah<br>Bielefeld University of Applied Sciences: Fachhochschule Bielefeld<br>Bielefeld, North Rhine-Westphalia GERMANY
**Corresponding Author Secondary Information:**	
**Corresponding Author's Institution:**	Bielefeld University of Applied Sciences: Fachhochschule Bielefeld
**Corresponding Author's Secondary Institution:**	
**First Author:**	Zafran Hussain Shah
**First Author Secondary Information:**	
**Order of Authors:**	Zafran Hussain Shah<br>Marcel Müller, Doctorate<br>Wolfgang Hübner, Doctorate<br>Tung-Cheng Wang, Doctorate

Powered by Editorial Manager® and ProduXion Manager® from Aries Systems Corporation

	Daniel Telman, Bachelor
	Thomas Huser, Doctorate
	Wolfram Schenck, Doctorate
**Order of Authors Secondary Information:**	
**Response to Reviewers:**	Dear Editor,<br><br>We thank you and the reviewers for the comments on our manuscript entitled, “Evaluation of Swin Transformer and knowledge transfer for denoising of super-resolution structured illumination microscopy data”. We appreciate the suggestions of the reviewers to strengthen our revised manuscript. We also hope that our edits and the responses we provide below satisfactorily address all your major and minor concerns. Furthermore, we have added in response to one of the reviewers a new Transformer-based SR-SIM image denoising algorithm. Therefore, we have updated the title of the manuscript accordingly. Because of this considerable extension of the presented work, we would like to request to change the category of the manuscript from technical note to research.<br><br>All the results at GigaDB are updated and the new code is uploaded to the GitHub repository. We believe that the manuscript is now suitable for publication in GigaScience.<br><br>##############################Reviewer # 1<br>#################################<br><br>Comment-1. To our knowledge, the "BioSR" dataset presented in reference [22] contains quite a few SIM reconstructions. While, the authors declare that "However, their collection of datasets consists of considerably fewer images and does not contain any super-resolution SIM images". The authors may do a detailed survey of the "BioSR" data set.<br><br>Response-1. We revised the text in this paragraph to clarify our statements and to provide some additional details about the BioSR dataset. Actually, the sentence "However, their collection of datasets consists of considerably fewer images and does not contain any super-resolution SIM images" was not intended to refer to the BioSR dataset. Hopefully, our writing is now better understandable.<br>As a side note: In comparison to the BioSR dataset collection with 2200 image pairs, our datasets consist of more than 9800 pairs of low- and high-resolution samples of 1024 x 1024 (width x height) pixels with different time stamps and noise levels. Furthermore, our datasets are ready to be used for different image restoration problems.<br><br>Comment -2. The authors may modify the bottom "(FFT)" in the "SIM Algorithm" box of Figure 1 as "(Inverse FFT)".<br><br>Response-2. We thank the reviewer and changed the respective figure.<br><br>Comment -3. We can see obvious artifacts and backgrounds in the reference images (Figure 2, magnified). Can the reference image quality be promoted further?<br><br>Response-3. All the reference images are generated using the fairSIM and SoftWorX reconstruction algorithms. These artifacts are generated by these SIM reconstruction algorithms, that is the reason we have developed our machine learning based tools that can be trained to remove such artifacts. The standard reference images can, however, not be further improved based on the traditionally used purely Wiener-filter-based algorithms. Due to this, we labeled our high-resolution output images as reference images instead of ground truth.<br><br>Comment -4. The UNet-fairSIM output in Figure 5b seems to be unreasonable and the

authors may try to explain this phenomenon and solve the problem.

Response-4. The UNet-fairSIM method was not able to denoise that specific image properly but we have replaced that sample with another test sample in Figure 9 of the revised manuscript which is slightly more in favor of UNet. However, considering original Figure 5b and new Figure 9, the only condition where the UNet is completely lacking is a direct transfer from training on dataset 4 (vesicles) to inference on dataset 2 (tubulin). In our view, this is not unreasonable. As the good results for this UNet for training from scratch on dataset 4 show, this UNet was properly trained before direct transfer. Therefore, the bad results for direct transfer mainly show that the UNet is not suited for this strategy.

Comment -5. Datasets 1-3 in Table 1 contain the same sample structures and may be combined into one dataset. By the way, the authors may provide other sample structures besides tubulin filaments and vesicles.

Response-5. The idea behind datasets 1-3 is to use these datasets of real world biological structures for different image restoration tasks. For example, dataset 1 can be used to denoise raw SIM data, dataset 2 can be used to denoise SR-SIM data, and dataset 3 can be used for super-resolution tasks or joint denoising and super-resolution tasks. In our view, this is a service to the potential user. The datasets are pre-structured in such a way that they can easily be used for these different tasks.

Comment -6. The authors can transfer Table 2 into the statistical boxplot for an intuitive representation.

Response-6. We thank the reviewer for this suggestion, we added the boxplots of the respective PSNR and SSIM values of all the findings in Figure 7 of the revised manuscript.

Comment -7. There are many deep learning-based SIM reconstruction methods like DFCAN/DFGAN, the authors may try these methods besides Red-fairSIM/Unet-fairSIM.

Response-7. In our revised manuscript, we propose a novel SwinT-fairSIM architecture based on the Swin Image Restoration (SwinIR) algorithm to denoise the SR-SIM images. Further details are explained in the revised manuscript (for details please see section “Shifted window Transformer for the denoising of SR-SIM images (SwinT-fairSIM)”). In this way, we hope to meet the demands of the reviewer by including an additional state-of-the-art deep learning-based image restoration technique in our comparison.

Comment -8. The authors may provide some discussions about "why the fine-tuning output is better than direct transfer?". Does this mean that the first/last few layers of the network are critical to its output?

Response-8. “Direct transfer” is the transfer of trained weights from one dataset to another dataset of the related task, whereas, in fine-tuning some of the layers of the pretrained models are retrained instead of training the entire model from scratch. We showed in the manuscript that direct transfer of weights from tubulin to vesicle data or vice versa results in a poor outcome. Fine-tuning is crucial to achieve competitive results. In the discussion section, we added a paragraph in which the critical role of the first/last few layers is discussed in light of these results.Comment -9. The authors may provide a summary and conclusion about the work.

Response-9. We added the conclusion section in the revised manuscript.

######################## Reviewer # 2
##

Comment-1. In the abstract, on the computer vision side, there is a large number of methods that can blindly reduce the image noise level. Also, for SIM data, there are already methods for denoising multiple structures.

Response-1. In response to this comment, we added a sentence about a Blind-SIM method (BS-CNN architecture) in the second paragraph of the introduction section with reference number [22] to the revised manuscript. We agree that blind denoising is an interesting and relevant area on its own, but we think that it is beyond the scope of the presented work. Actually, in our previous work [19], we used a blind denoising algorithm from classical computer vision in comparison to deep learning-based approaches. The deep-learning approaches, trained to a specific task and data, were clearly superior. Therefore, we decided to focus here on algorithms which are trained in the first place with specific data and tackle the research questions, how well they generalize, and what can help in improving their generalization capabilities (i.e., fine-tuning).

Comment-2. On page 2, 'leads to low SNR', the abbreviation needs to be explained.

Response-2. We thank the reviewer, and we have addressed this concern in the revised manuscript.

Comment -3. On page 2, the authors compare existing open-source datasets and enumerate the amount of data in them, so it is better to introduce the dataset presented in this paper with the amount as well.

Response-3. We describe the characteristics of the datasets along with the total number of samples in Table 1 of the revised manuscript.

Comment -4. In SIM data, the data type is 16bit per pixel, however, the denoising method, i.e. RED was originally designed for 8bit images. In addition, the PSNR calculation in the author's code calls TensorFlow's built-in functions (tf_psnr) , has the corresponding modification been made for dynamic range, or has the data been pre-processed?

Response-4. We scaled the images from 16-bit to floating values (i.e., all the pixel values are between 0.0 and 1.0) before the training process. The PSNR values are calculated in the floating point representation. The maximum intensity required as a parameter for the PSNR calculation is adjusted accordingly. After the prediction, the images are converted back to 16-bit for storage and visualization,
etc.

Comment-5. The code is publicly available, but the data is not, and it is recommended that some data be made available for testing.

Response-5. We already submitted our data to the GigaScience Database, so that they are publicly available for the research community after acceptance of the manuscript.

Comment-6. How is the distance represented by each pixel (scale bar) derived in the image? For example, 80 nm in Table 1 and 8 μm in Fig.1 and 2. I suggest adding the corresponding explanation.

Response-6. 80nm and 40nm refers to the pixel sizes in the raw images (80 nm/pixel) and reconstructed images (40 nm/pixel) which are mentioned in Table 1 of the manuscript. 80 nm/pixel arises as the combination of the magnification of the optical system (microscope) and the physical pixel size of the camera. The SIM reconstruction process then doubles the number of pixels, which halves their size, as
part of the frequency-spaced reconstruction step. 4μm refers to the length of the scale bars, which is set in relation to the pixel size (by a built-in ImageJ function). Scale bars are now consistently set to 4μm.

Powered by Editorial Manager® and ProduXion Manager® from Aries Systems Corporation

	Comment -7. How is noise level defined? In the domain of machine learning, level denotes the standard deviation of Gaussian noise, which needs to be disambiguated here.<br><br>Response-7. The datasets which are presented in this manuscript are collected from a real biological sample and contain real noise which is a mixture of Gaussian-Poisson noise plus the reconstruction artifacts created by the SIM reconstruction algorithm. Therefore, these real datasets do not have any artificial or randomly added noise (such as Gaussian or Poisson with some specific intensity level). The real noise occurs due to the detection of fewer photons from biological samples, resulting in a bad signal-to-noise ratio. However, the timestamp is considered to be a relevant factor in these datasets to distinguish the level of noise in the images. For example, in datasets 1-3, small timestamps represent low noise levels, whereas large timestamps represent high noise levels as shown in Figure 3. The noise levels are explained in the subsection on “Dataset 2” in the revised manuscript.<br><br>############################## Reviewer # 3 ##############################<br><br>Comment -1. Firstly, datasets 1-3 are based on 2D SIM reconstruction, whereas dataset 4 is based on 3D SIM reconstruction, which leads to different axial resolutions, making it difficult to test the generalization capability of the model.Response-1. The raw data of all data sets are true 3D-SIM data acquisitions. The only difference is in the way how the reconstructions are obtained. fairSIM-based reconstructions use the 15 raw images of a particular focus position to reconstruct a single slice (which we call a 2D reconstruction), while the softWoRx reconstruction always reconstructs a full 3D data set. While a full 3D reconstruction provides higher axial resolution, optical sectioning, i.e. the capability of removing blurred out-of-focus light, can be and is achieved by both 2D and 3D reconstruction. More importantly, the lateral resolution increase is the same for 2D and 3D SIM reconstruction. This makes single-slice (2D) SIM reconstructions and full 3D SIM reconstructions similar enough to work in the same processing pipeline. During this work, we train only 2D models with both datasets 2 and 4. Each z-plane in dataset 4 is treated as a different field-of-view (FOV). This terminology results in the conversion of dataset 4 from a 3D to a 2D dataset. Furthermore, 15 raw SIM images were taken for each plane during the collection of all data, which consists of three pattern orientations and five equally distributed phases for each angle. This rules out the different axial resolution point.<br><br>Comment -2. Secondly, the noise level has only four levels, and the sample types are limited to two structures, which is significantly less than the 'BioSR' dataset cited as a reference in the manuscript.<br><br>Response-2. This concern is already addressed above (see Reviewer # 1, comment-1, and comment-5). The datasets provided by us have their own merits such as the large number of samples and being especially structured for common use cases in benchmarking and research.<br><br>Comment -3. Lastly, the authors need to provide a schematic of the fine-tuning strategies they used.<br><br>Response-3. We thank the reviewer for this suggestion; we now show the schematic of direct transfer and fine-tuning in Figure 5 of the revised manuscript.<br><br>Note: A pdf file attached named 'Cover_Rebuttal_GIGA-D-23-00044.pdf file' also contains all the responses.
**Additional Information:**	
**Question**	**Response**

Powered by Editorial Manager® and ProduXion Manager® from Aries Systems Corporation

Are you submitting this manuscript to a special series or article collection?	No
**Experimental design and statistics**<br><br>Full details of the experimental design and statistical methods used should be given in the Methods section, as detailed in our Minimum Standards Reporting Checklist. Information essential to interpreting the data presented should be made available in the figure legends.<br><br>Have you included all the information requested in your manuscript?	Yes
**Resources**<br><br>A description of all resources used, including antibodies, cell lines, animals and software tools, with enough information to allow them to be uniquely identified, should be included in the Methods section. Authors are strongly encouraged to cite Research Resource Identifiers (RRIDs) for antibodies, model organisms and tools, where possible.<br><br>Have you included the information requested as detailed in our Minimum Standards Reporting Checklist?	Yes
**Availability of data and materials**<br><br>All datasets and code on which the conclusions of the paper rely must be either included in your submission or deposited in publicly available repositories (where available and ethically appropriate), referencing such data using a unique identifier in the references and in the "Availability of Data and Materials" section of your manuscript.<br><br>Have you have met the above requirement as detailed in our Minimum	Yes

Standards Reporting Checklist?	

Powered by Editorial Manager® and ProduXion Manager® from Aries Systems Corporation

Manuscript

Click here to access/download;Manuscript;Manuscript_Revised_GIGA-D-

OXFORD (GIGA)^n SCIENCE

GigaScience, 2023, 1–14

doi: xx.xxxx/xxxx
Manuscript in Preparation
Paper

PAPER

Evaluation of Swin Transformer and knowledge transfer for denoising of super-resolution structured illumination microscopy data

Zafran Hussain Shah[1,*], Marcel Müller[2], Wolfgang Hübner[2], Tung-Cheng Wang[2,3], Daniel Telman[1], Thomas Huser[2] and Wolfram Schenck[1,*]

[1]Faculty of Engineering and Mathematics, Bielefeld University of Applied Sciences and Arts, Interaktion 1, 33619 Bielefeld, Germany and [2]Faculty of Physics, Bielefeld University, Universitätsstr. 25, 33615 Bielefeld, Germany and [3]Leica Microsystems CMS GmbH, Am Friedensplatz 3, 68165 Mannheim, Germany

*zafran_hussain.shah@hsbi.de; wolfram.schenck@hsbi.de

Abstract

In recent years, convolutional neural network (CNN)-based methods have shown remarkable performance in the denoising and reconstruction of super-resolved structured illumination microscopy (SR-SIM) data. Therefore, CNN-based architectures have been the main focus of existing studies. Recently, however, an alternative and highly competitive deep learning architecture, Swin Transformer, has been proposed for image restoration tasks. In this work, we present SwinT-fairSIM, a novel method for restoring SR-SIM images with low signal-to-noise ratio (SNR) based on Swin Transformer. The experimental results show that SwinT-fairSIM outperforms previous CNN-based denoising methods. Furthermore, the generalization capabilities of deep learning methods for image restoration tasks on real fluorescence microscopy data have not been fully explored yet, i.e., the extent to which trained artificial neural networks are limited to specific types of cell structures and noise. Therefore, as a second contribution, we benchmark two types of transfer learning, i.e., direct transfer and fine-tuning, in combination with SwinT-fairSIM and two CNN-based methods for denoising SR-SIM data. Direct transfer does not prove to be a viable strategy, but fine-tuning achieves results comparable to conventional training from scratch, while saving computational time and potentially reducing the amount of required training data. As a third contribution, we publish four datasets of raw SIM images and already reconstructed SR-SIM images. These datasets cover two different types of cell structures, tubulin filaments and vesicle structures. Different noise levels are available for the tubulin filaments. These datasets are structured in such a way that they can be easily used by the research community for research on denoising, super-resolution, and transfer learning strategies.

Key words: Structured illumination microscopy; Fluorescence microscopy; Deep learning; Tranformers; Swin Transformer; SwinIR; Convolutional neural networks; Denoising; Image restoration; Transfer learning; Fine-tuning

Introduction

In optical microscopy, super-resolution structured illumination microscopy (SR-SIM) plays a significant role in the field of biological and biomedical studies to analyze living cells and biological specimens with characteristic features below the resolution limit of classical microscopes (approx. 250 nm for high-end systems with oil-immersion objective lenses). Structures of interest include e.g. the internal organelles of mitochondria, cellular cytoskeleton, virus particles, or small vesicles [1, 2, 3, 4, 5]. SR-SIM is an important super-resolution approach to disentangle complex biological cellular structures with an up to twofold enhancement of spatial resolution beyond the

Compiled on: July 13, 2023.
Draft manuscript prepared by the author.

diffraction limit. During the process of super-resolution imaging, SR-SIM involves the illumination of the biological sample with spatially patterned light following a sinusoidal intensity distribution. A series of raw images with typically 3 or 5 different phase positions and 3 angles of orientation of the illumination pattern are typically acquired [6]. Subsequently, frequency-domain based image reconstruction algorithms, e.g. implemented in software packages such as fairSIM [7], OpenSIM [8], and Python-based packages [9] are applied to a set of raw SIM images to generate the final twofold super-resolved images. SR-SIM has many advantages over other super-resolution methods e.g., it does not require special sample preparation, it permits the use of conventional fluorophores in multiple colors, simultaneously, and it allows for imaging at high speed and large fields-of-view (FOV) while being compatible with live cell samples by making efficient use of low illumination intensity levels [10, 11]. The conventional SR-SIM reconstruction algorithms have some limitations whenever the signal-to-noise level of the raw images is poor due to low fluorescence emission or short exposure times [12]. In general, in fluorescence microscopy weak emission due to, e.g. low labeling densities, high light scattering or absorption, or optical aberrations is often encountered [13, 14]. In the case of several super-resolution fluorescence microscopy methodologies, this leads to low signal-to-noise ratio (SNR), and poor image reconstructions or artifacts. Nevertheless, SR-SIM imaging provides better resolution and optical sectioning abilities than confocal microscopy with a just minimally larger number of raw images to be acquired [15].

In the last decade, deep learning methods have become generally accepted in image processing. In addition, they are being used with increasing success for the restoration of SR-SIM images, e.g. for denoising [16, 17, 18, 19, 20]. Qiao et al. proposed a generative adversarial network (GAN) based deep Fourier channel attention network (DFCAN) method to reconstruct SR-SIM images under low SNR conditions [21]. Xypakis et al. introduced a custom convolutional neural network (CNN) architecture for blind-SIM: BS-CNN [22]. Liu et al. proposed a dual-domain learning strategy for the reconstruction of SIM images [23]. Authors in [24] suggested another GAN based channel attention generative adversarial network (caGAN) to improve the quality of 3D-SIM reconstruction using fewer raw samples of low SNR.

Recently, so-called Transformers have shown great success in various natural language processing tasks [25, 26, 27, 28, 29]. Since then, as an alternative to CNNs, Transformer-based architectures have been adapted to computer vision tasks as well, such as classification [30], detection [31, 32], and image restoration [33]. Vision Transformers for the image restoration typically divide each image into fixed-size patches and process each patch independently to limit computational complexity [34], which results in the introduction of border artifacts around each patch in the restored image. The Swin Transformer overcomes this shortcoming by integrating a shifted window operation along with a few CNN layers in the Transformer architecture [35]. Swin serves as the basis for the Shifted window Image Restoration (SwinIR) method [36], which has been proposed especially for various image restoration tasks. Although these latest Transformers outperform CNN-based methods in conventional image restoration to some extent, they were never explored for the restoration of high-resolution SR-SIM microscopy images. Therefore, during this work, we propose a SwinIR based Transformer architecture named "SwinT-fairSIM" to denoise SR-SIM images under low SNR conditions.

Both CNN- and Transformer-based methods require a large number of images to train the underlying models. In the field of microscopy, the sheer size and storage requirements of datasets of high-resolution reconstructed microscopic images make them difficult to produce and typically not publicly available. The few open source datasets available are mostly related to wide-field microscopy and contain a relatively small number of images. For example, Zhang et al. [37] collected three wide-field microscopy datasets purely for denoising tasks without providing high-resolution ground truth images obtained by SR-SIM technology. They used image averaging to generate ground truth data with high SNR images. Zhou et al. [38], on the other hand, published a dataset called "Widefield2SIM" using wide-field fluorescence microscopy. They captured 120 different fields of view (FOV) with 400 low SNR images for each FOV and generated high-resolution ground truth data using SR-SIM imaging technology. Qiao et al. [21] presented the "BioSR" dataset consisting of 2200 pairs of low-resolution (LR) raw data and high-resolution (HR) data. Four different biological structures (CCPs, ER, MTs, F-actin), nine signal levels (15-600 average photon count), and two upscaling-factors (linear SIM and non-linear SIM) are covered in the BioSR dataset according to Qiao et al. Similarly, Hagen et al. [39] published a variety of datasets which were collected using wide-field and confocal microscopy. They captured various fluorescently labeled structures such as actin, mitochondria, membrane, and nuclei with low and high SNR. However, their collection of datasets consists of significantly fewer images than the BioSR data collection.

Here, we present a series of datasets that are related to SR-SIM microscopy for image restoration tasks. These datasets cover two types of biological structures, tubulin filaments, and vesicles, with several fields-of-view for the denoising, super-resolution, and joint denoising and super-resolution tasks. In our datasets, noisy input and reference output images were obtained by using SR-SIM reconstruction algorithms. We believe that our datasets will be helpful for the research community to benchmark different deep-learning based denoising and super-resolution (SR) methods, not least because of the rather large number of provided samples.

Finally, we show how some of these datasets can be used to demonstrate the generalization capabilities of image restoration algorithms in the field of SIM microscopy. To this end, we apply the concept of transfer learning to three algorithms for the denoising of SR-SIM data. Two of these algorithms are based on CNNs and were proposed by us in [16]. They are called "Red-fairSIM" and "UNet-fairSIM". The third algorithm is the above-mentioned Transformer architecture "SwinT-fairSIM", which is first proposed in this paper. With respect to transfer learning, we compare two methods from this area: Direct transfer and fine-tuning. In direct transfer, a model pre-trained on one type of data is used for inference on a related but different type of data. In fine-tuning, part of the pre-trained model is retrained on the new type of data before inference. In previous work, we were already able to show that some CNN-based algorithms for the denoising of SR-SIM images are robust to different noise levels and SIM modes, e.g. varying pattern spacings at different illumination wavelengths [19]. However, due to the limited amount of SR-SIM data available at that time, we were not able to explore the techniques of transfer learning in more depth. Thus, here, we also aim to answer the following questions related to direct transfer and fine-tuning: (1) If a model is trained on a specific biological structure with a specific type of noise, will it also generalize well to denoising another, different structure with another type of noise? And: (2) Is fine-tuning of a pre-trained model (i.e., previously trained on one type of structure and noise) more effective than training the model from scratch? To answer these questions, we have conducted a series of experiments which are discussed in later sections.

In particular, the contribution of this work is threefold:

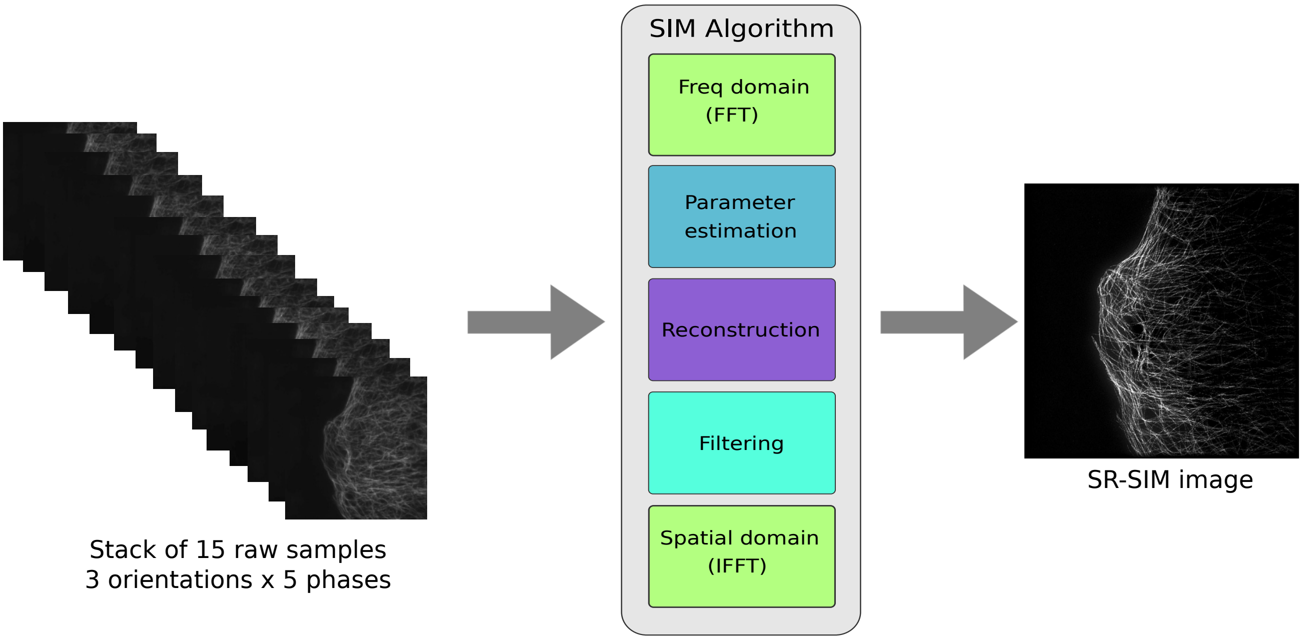

Figure 1. The architecture of the image reconstruction algorithms which are used to generate the reconstructed SR-SIM images in this work. Both reconstruction algorithms (i.e., as implemented in the fairSIM and softWoRx software) are based on the SIM algorithm. During the reconstruction of SR-SIM images, a stack of 15 raw SIM images of size 512 × 512 pixels is processed by the respective software which generates the high-resolution SR-SIM image of size 1024 × 1024 pixels as output.

First, we present high-resolution SR-SIM datasets for image denoising and super-resolution tasks. Second, we propose a method based on the SwinIR architecture for denoising SR-SIM images. Third, we evaluate the potential of direct transfer and fine-tuning for different Transformer- and CNN-based models.

Materials and Methods

SR-SIM microscopy and sample preparation

The raw SIM images for all the datasets were acquired using a DeltaVision OMX V4 (GE Healthcare, Chicago, IL, USA) 3D-SIM imaging system. To prepare the raw SIM image data (datasets 1-3) of the tubulin cytoskeleton, U2OS cells were cultured in DMEM supplemented with 10%FBS and grown on round coverslips of 170±5 μ m thickness (No. 1.5H). Cells were fixed with 4% PFA for 15 min., followed by PBS washes, and permeabilization with 0.5% Triton-X100 for 3 min. Another two rounds of PBS washes were done before blocking with 3% BSA. For immunolabeling of the tubulin microfilaments, cells were stained with anti-tubulin antibody (Invitrogen Cat. No. 322500) 1:400 for 2 hr at room temperature, followed by a PBS wash and one additional hour of incubation with Alexa 488-conjugated anti-mouse IgG 1:400. Afterwards the cells were then briefly washed with PBS before Vectashield was applied to embed the coverslip onto a standard microscopy glass slide for imaging. For the preparation of raw SIM images with vesicle structures (dataset 4), U2OS cells were transfected with Lipofectamine 3000 according to the manufacturers protocol (Thermofisher Cat. No. L3000-001) together with a plasmid expressing the vesicular Lamp1 protein fused to the fluorescent protein mScarlet. After 24 hr transfection the cells were fixed with 4% PFA for 10 min., followed by PBS washes and Vectashield mounting prior to imaging. The vesicular structures represent lysosomes.

Dataset preprocessing and image reconstruction

Dataset 1 is a raw dataset without any image processing or reconstruction applied to the raw SIM images. The images in the datasets 2 and 3 are reconstructed by using the open-source fairSIM reconstruction algorithm as shown in Figure 1. fairSIM implements a single-slice (2D) SR-SIM image reconstruction algorithm [7]. It works in three steps: parameter estimation, reconstruction, and filtering. The mathematical and algorithmic details of the fairSIM reconstruction method are explained in the original publication [7]. A synthetic optical transfer function, with NA = 1.4, $\lambda = 525\,\text{nm}, a = 0.31$ (a is a compensation parameter,see [7, 40]) is used. For the tubulin samples, a background of 500 counts per pixel is subtracted during the reconstruction process. SR-SIM reconstruction parameters (pattern orientation, global phase, etc.) are automatically determined by fairSIM's standard, iterative cross-correlation approach. Filter parameters are set to a generalized Wiener filter with a strength of $w = 0.05$, apodization is set at 1.9× the resolution limit with a *bend* of 0.8. A notch-style filter implemented as *OTF attenuation* with a strength of 0.995 and a FWHM of $1.2\,\mu\text{m}^{-1}$ is used. The full information about the functionality of these parameters is explained in [7], and the general guide for using SIM reconstruction parameters is discussed in [40]. The code used to generate the samples of dataset 2 is available at [41]. The samples in dataset 4 (the vLamp1-mScarlet expressing cell) were reconstructed using the commerical software 'softWoRx v7' (GE Healthcare manufacturer's software) for 3D-SIM. For an overview of all datasets, see Table 1.

Description of datasets

Dataset 1

Dataset 1 contains around 101 fields-of-view (FOV) of tubulin filaments and each FOV further consists of 3000 images (all in one TIF file for each FOV). In each FOV, a stack of 15 raw SIM images represents the combination of 5 orientations and

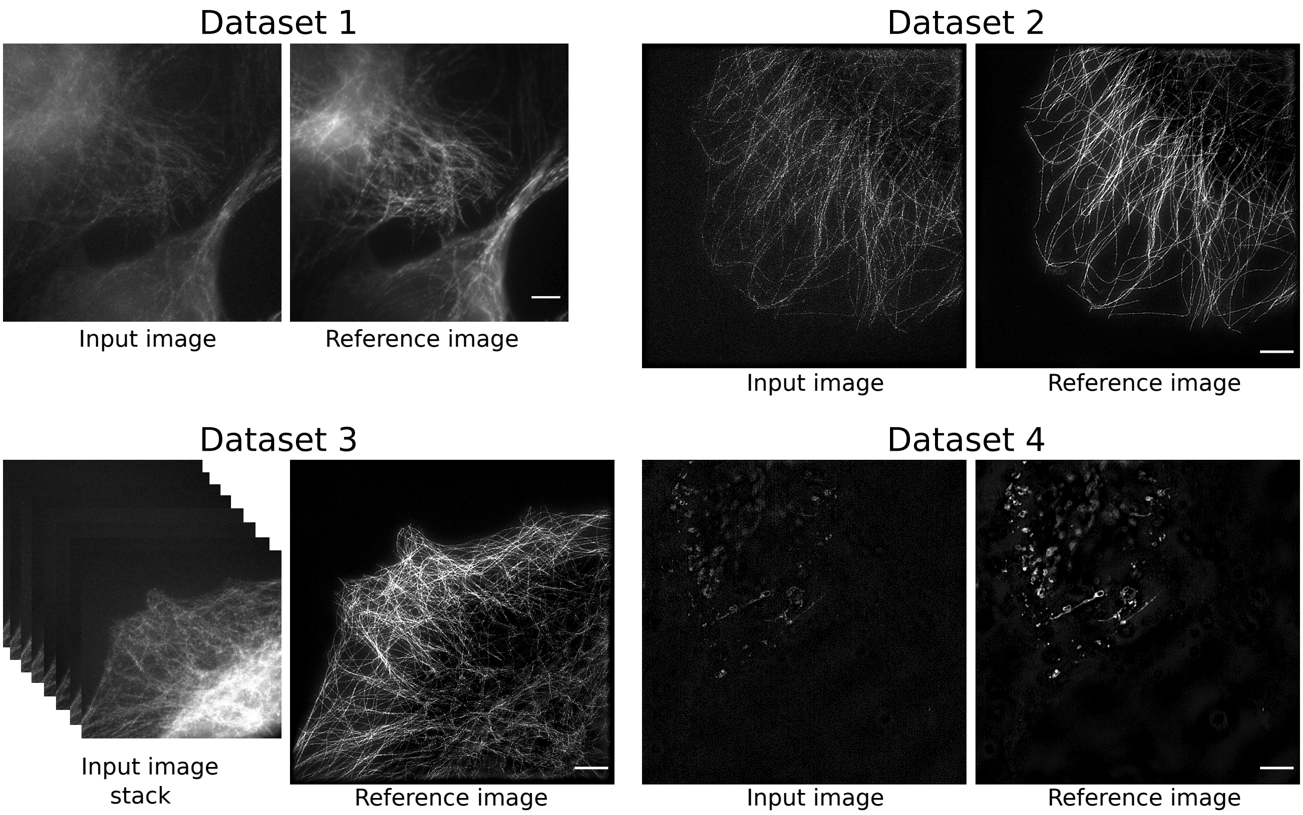

Figure 2. Dataset 1 consists of raw SIM data. The input-reference pairs represent each phase and orientation from different noise levels of tubulin filaments. The size of the input and reference images is 512 × 512 pixels in dataset 1. Scale bar: 4 µm. Dataset 2 contains the reconstructed SR-SIM images of tubulin structure, and each input-reference pair has a size of 1024 × 1024 pixels. Scale bar: 4 µm. In dataset 3, raw SIM data in the form of an image stack (i.e., 15 × 512 × 512 pixels) are used as input samples, whereas the reconstructed SR-SIM images (1024 × 1024 pixels) are used as the corresponding reference samples for joint denoising and super-resolution. Scale bar of reference image: 4 µm. Dataset 4 is based on the reconstructed SR-SIM images of size 1024 × 1024 pixels of fluorescently labeled vesicles. Scale bar: 4 µm.

3 phases, whereas this full stack is repeatedly captured for 200 timestamps. The signal-to-noise ratio decreases with every timestamp. Thus, dataset 1 contains a total of 303000 raw SIM images of size 512 × 512 (width ×height) pixels with 15 combinations of phase and orientation at each timestamp. Each timestamp in the raw images lasts approximately 25 ms. Each pixel contains a single 16-bit integer value captured by the microscope's camera, which is calibrated to provide a signal linear in photon count for each pixel. This is typical for scientific camera systems, but dissimilar to standard image processing, where often gamma mapping is applied between light intensity and pixel values. This dataset can be used mainly for image denoising tasks (from noisy input to output with higher signal-to-noise ratio). Therefore, the images from timestamp 1 are intended as output images (reference/ground truth), and the rest of the images can be categorized as input images as shown in Figure 2.

Dataset 2

We constructed dataset 2 by applying the fairSIM reconstruction algorithm to the raw SIM images from dataset 1. Dataset 2 contains therefore pairs of reconstructed high-resolution SR-SIM images, each pair consisting of the noisy input and the reference output. The process of the generation of input and output SIM images from the raw SIM images is shown in Figure 1. The 15 raw SIM images of size 512 × 512 (width × height) pixels of different phases and orientations are propagated into the fairSIM algorithm to reconstruct the SR-SIM images of size 1024 × 1024 (width × height) pixels. During the formation of this dataset, the raw samples from timestamp 1 were used to generate the output images (i.e., reference images). We use the term 'reference images' instead of 'ground truth images' because of the SIM reconstruction artifacts in the output images of this dataset. The input samples were reconstructed by using the raw SIM images from timestamps 176 – 200. Therefore, dataset 2 is composed of 2525 reconstructed pairs of SR-SIM images with a size of 1024 × 1024 (width × height) pixels extracted from the 101 FOV. In addition to these 2525 image pairs, for the last 20 FOV we also include image pairs in the data collection where the noisy input is from timestamps 76 – 100, 126 – 150 and 176 – 200. This additional data can be used to create test sets to evaluate the robustness of denoising networks for different noise levels. In our previous work [19], we denoted data from timestamp 26 – 50 as noise level 1. Similarly, noise level 2, 3, and 4 correspond to the data from the timestamps 76 – 100, 126 – 150 and 176 – 200 (shown in Figure 3). Overall, dataset 2 is generated mainly for the denoising of SR-SIM images.

Dataset 3

Dataset 3 was created mainly for joint denoising and super-resolution tasks. The composition of this dataset is based on the stack of raw noisy input SIM images obtained from Dataset 1 of noise level 4 and the reference SR-SIM images from Dataset 2 of timestamp 0. The raw noisy SIM images in the input stack represents different illumination phases and orientations. The reference samples are the reconstructed high-resolution SR-SIM images. The size and dimension of each input sample are 15 × 512 × 512 (depth × width × height) pixels. The size of the output or reference sample is 1024 × 1024 (width × height) pixels. The input and reference samples in this dataset contain tubulin filaments as biological structures.

Table 1. Description of all the datasets

Dataset	Dataset 1	Dataset 2	Dataset 3	Dataset 4
Structure	tubulin filaments	tubulin filaments	tubulin filaments	vesicles
Microscope	SR-SIM microscopy	SR-SIM microscopy	SR-SIM microscopy	SR-SIM microscopy
Pixel Size, nm	80	40	80 (input) / 40 (ref.)	40
Number of timestamps	200	200	200	max: 99, min: 15
Fields-of-view	101	101	101	175
Input image size, pixels	512x512	1024x1024	15x512x512	1024x1024
Output/Reference image size, pixels	512x512	1024x1024	1024x1024	1024x1024
No of samples	303000	2525	2525	7284
Reconstruction	raw data	fairSIM	fairSIM	softWoRx

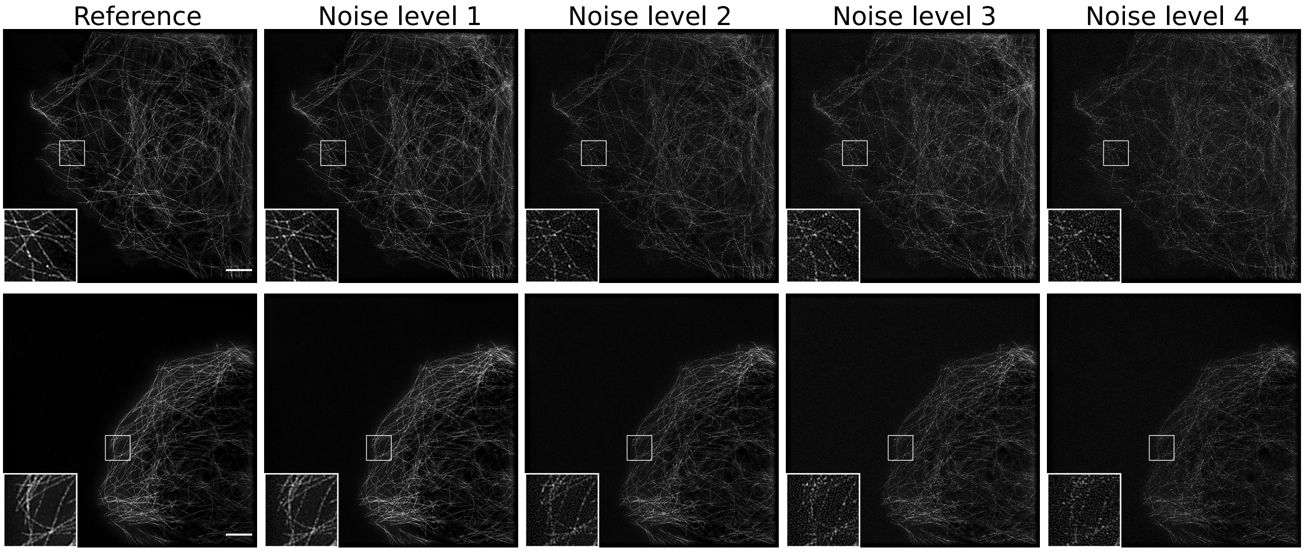

Figure 3. SR-SIM images from two different FOV, each shown at several noise levels. Each noise level corresponds to a different range of timestamps, e.g., images of noise level 4 are taken at the last timestamps and contain a high level of noise. Similarly, images of noise level 1 represent early timestamps with very low noise. The reference image is recorded at timestamp 0 and has the highest signal-to-noise ratio. Scale bar: 4 µm.

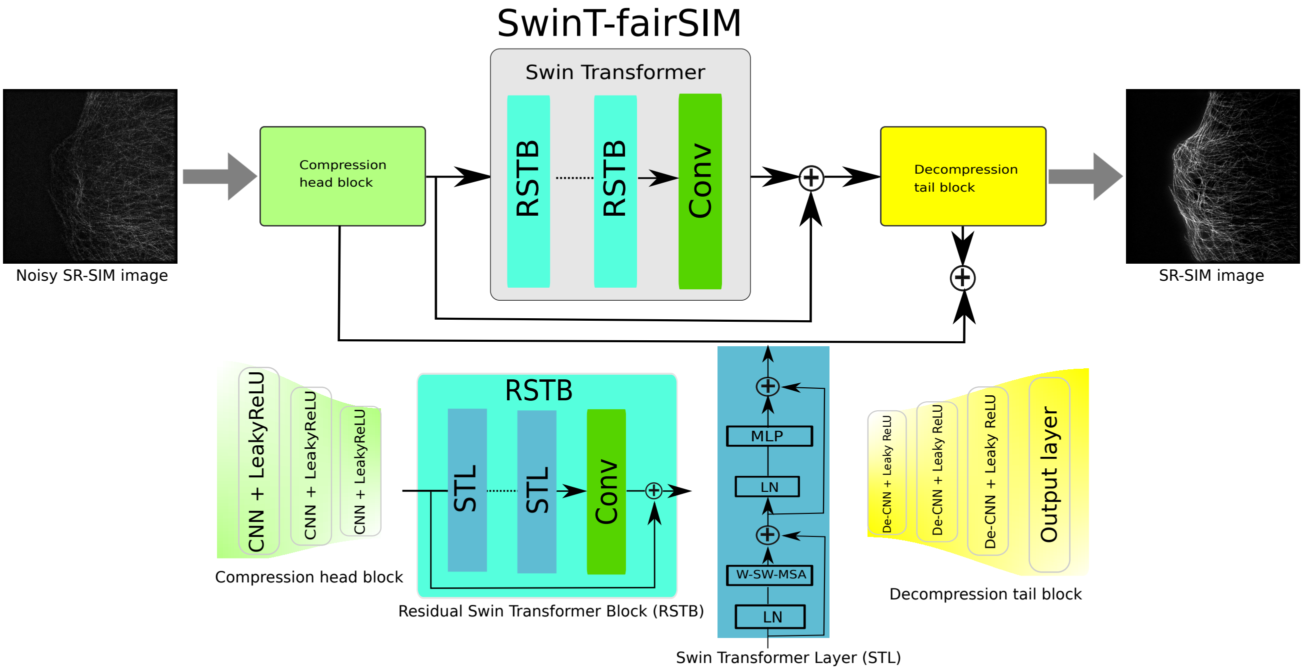

Figure 4. The architecture of the SwinT-fairSIM denoising method. A more detailed view into each block of the pipeline is shown in the lower part of the figure.

Dataset 4

Dataset 4 is composed of 3D-SR-SIM images showing vesicles in U2OS cells (i.e. typically round intracellular droplets) and can be used mainly for denoising tasks. This data is composed of several z-stack slices and each slice is captured for different timestamps. The raw data of this dataset contains 175 FOV,

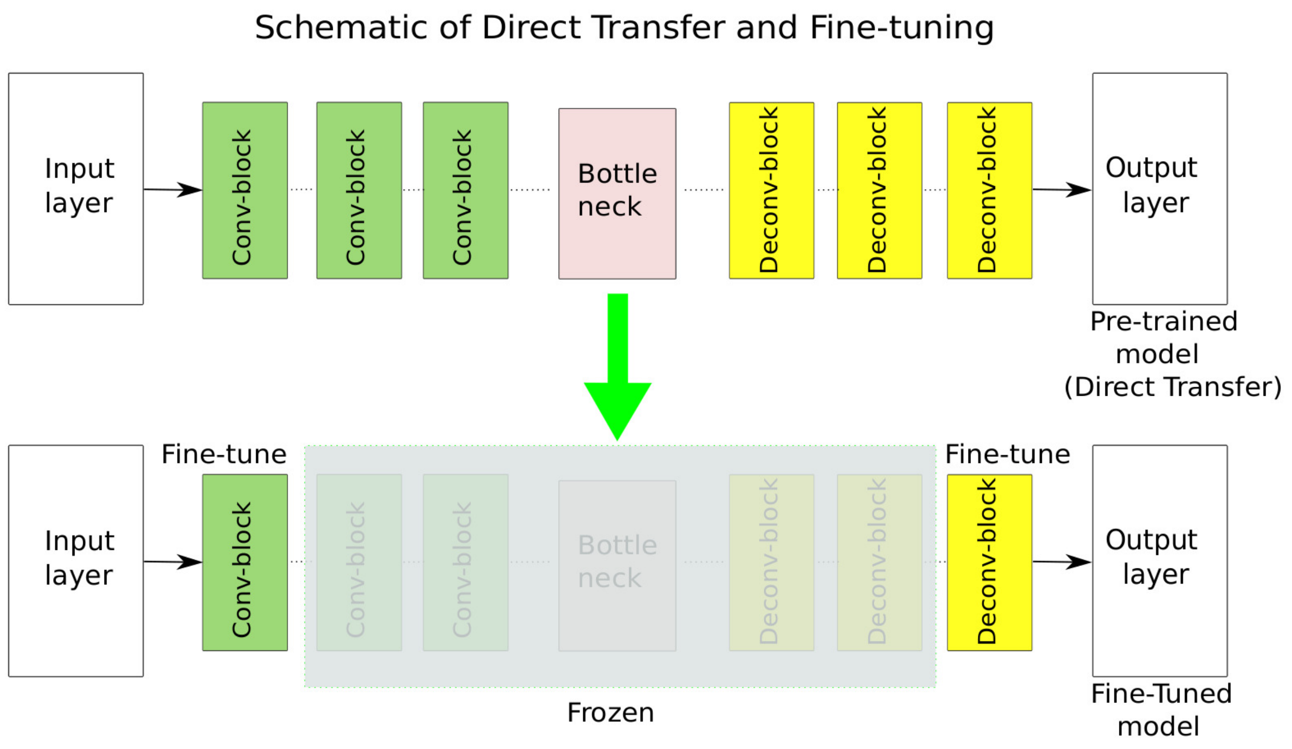

Figure 5. Schematic diagram of direct transfer and fine-tuning. The first pipeline shows the pre-trained model. The highlighted region in the second pipeline shows the frozen part; all parts of the model that are not frozen are retrained in the fine-tuning strategy.

and each field-of-view is recorded for a different number of timestamps.[1] We acquired a total of 7284 pairs of input and output SR-SIM images. The size of the input and reference images is 1024 × 1024 (width × height) pixels. In each image pair, the output/reference image is taken from timestamp 1 of the respective FOV, whereas the input images are taken from the following time stamps and exhibit therefore a lower signal-to-noise ratio. The reconstruction of the original raw SIM images into the SR-SIM images in this dataset was performed by using the softWoRx v7 software (GE Healthcare manufacturer's software).

Data partitioning

In our experiments, in the first three datasets, the images of the last 20 FOV were used as test samples, and the remaining 81 FOV were used for the training set. The training set of dataset 2 is therefore composed of 2025 samples (image pairs) from 81 FOV and the test set is composed of 500 samples from 20 FOV. Similarly, in dataset 4 we used 5562 samples from 121 FOV for the training set and 1380 samples from 46 FOV for the test set; the remaining 8 FOV were discarded because their reference images contain only noise without any meaningful structure. Datasets 1, 2, and 4 can only be used for denoising tasks, however, the data from dataset 3 can be used for both joint denoising and super-resolution. To reproduce the results of this work, we share the source code on the GitHub repository [42], the trained models [43] and the data on GigaDB ["link will be uploaded after the allocation of space from Giga Database"].

1 This is a slight simplification: Originally, nine different fields-of-view were recorded, each at a varying number of depth levels (z-planes). However, the recorded structures in the different z planes are very different from each other, so we categorize them here as separate FOV. The file names in the dataset contain also indices for these original fields-of-view and for the respective z-plane.

Shifted window Transformer for the denoising of SR-SIM images (SwinT-fairSIM)

The architecture of SwinT-fairSIM is composed of three main components: Compression head, Swin Transformer, and decompression tail block as shown in Figure 4. The architecture of SwinT-fairSIM is an extended version of the SwinIR architecture [36]. The compression head block is responsible for shallow feature extraction and the downsizing of the input images, the Swin Transformer block is based on encoder-decoder components to recover missing information and for deep feature extraction, and the decompression tail component is used for upsampling and restoring the features in the resultant images. The compression head block is composed of three convolution layers to compress the input image features to a size of 256×256 (width × height) pixels by using a stride of size 2. The encoder-decoder Swin Transformer block is made up of several Residual Swin Transformer blocks (RSTB), and each RSTB is based on few Swin Transformer layers (STL) and convolutional layers [36]. The STL is further composed of two window (W) and shifted window (SW) based multi-head self-attention (MSA) modules, followed by a multi-layer perceptron that has two fully-connected layers with GELU non-linearity [25, 35]. The shifted window partitioning strategy introduces connections between non-overlapping patches in the preceding layer and is found to be effective in a variety of computer vision tasks [36]. The Swin Transformer layer first reshapes the input (X) of size $H \times W \times C$ (height × width × channels) into $\frac{HW}{M^2} \times M^2 \times C$, where the $\frac{HW}{M^2}$ is the total number of windows or patches. Two additive based residual skip connections are also used in each STL unit. The query (Q), key (K), and value (V) matrices are then calculated for each window separately to obtain self-attention.

$$Q = XP_Q, \; K = XP_K, \; V = XP_V \qquad (1)$$

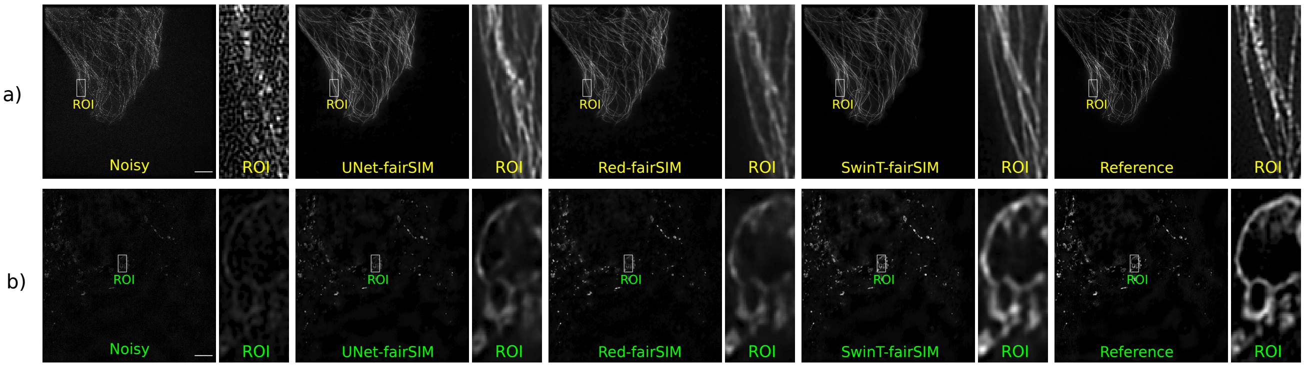

Figure 6. Results for test samples from datasets 2 and 4 are shown in this figure in the first row **(a)** and second row **(b)**. The outputs of UNet-fairSIM, Red-fairSIM, and SwinT-fairSIM are shown next to each other. The second, fourth, sixth, eighth, and tenth column depict the cropped and zoomed regions of interests (ROIs) from the full-size SR-SIM images (1024 × 1024 pixels). The cropped ROIs of size 50 × 100 pixels were upsampled to 150 × 300 pixels for demonstration purposes. Scale bar: 4 µm.

In equation (1), P_Q, P_K, and P_V are the shared projection matrices across all the windows. The attention matrix is then computed by the self-attention mechanism in a local window as follows:

$$\text{Attention}(Q, K, V) = \text{SoftMax}(QK^T/\sqrt{d} + B)V \quad (2)$$

where B in equation (2) is the learnable relative positional encoding and d is the dimension of query and key features. The consecutive Swin Transformer layers are defined as:

$$\hat{X}^l = \text{W-MSA}(\text{LN}(X^{l-1})) + X^{l-1} \quad (3)$$
$$X^l = \text{MLP}(\text{LN}(\hat{X}^l)) + \hat{X}^l \quad (4)$$
$$\hat{X}^{l+1} = \text{SW-MSA}(\text{LN}(X^l)) + X^l \quad (5)$$
$$X^{l+1} = \text{MLP}(\text{LN}(\hat{X}^{l+1})) + \hat{X}^{l+1} \quad (6)$$

In equations (3)-(6), $\hat{X}^l$ and X^l denote the output features of the (S)W-MSA and MLP modules for layer l, where LN represents the LayerNorm operation.

Finally, the decompression tail block accommodates 3 transposed convolutional layers along with the output convolutional layer to transform and upsample the feature maps into the final resultant images. The necessity of head and tail block is to scale down and upsample the SR-SIM images with a size of 1024 × 1024 (height × width) pixels. In the SwinT-fairSIM method, we set the RSBT, STL, window size, and the attention head numbers to 5, 5, 8, and 120 respectively. The adjacent layers in the head and tail blocks are connected via additive skip connection and contain the same number of kernels. These blocks differentiate our architecture from the existing SwinIR architecture [36]. These blocks reduce the computational effort in the central Transformer part of the architecture and therefore adapt the Swin Transformer block to images with a high pixel count.

The name “SwinT-fairSIM” was chosen to be consistent with the names of our previously proposed CNN-based denoising methods for SR-SIM data, “Red-fairSIM” and “UNet-fairSIM” [16]. Red-fairSIM and UNet-fairSIM are described in detail in [16]. These algorithms originally worked only in conjunction with the fairSIM software [7] for SR-SIM reconstruction. In contrast, in this paper, we also use the softWoRx implementation of the SIM algorithm for dataset 4. However, to be consistent with the naming scheme in [16], we keep the suffix “fairSIM” in all of our deep learning based approaches.

Transfer learning and fine-tuning

In transfer learning in general, the knowledge of a trained model from a related task that has been learned is transferred to another task from the same domain [44]. The parameters of the pre-trained model, trained on specific data and for a specific task, are transferred to the different data but related task [45, 46]. The use of transfer learning in deep learning is very useful to reduce the computational demands and time complexity [47]. In addition, transfer learning is also very helpful when it comes to alleviating large dataset requirements [48].

The simplest form of transfer learning is to apply a trained model directly to another task without retraining. This direct use of a pre-trained model on new test data is called “direct transfer”. Beyond this most basic approach, fine-tuning is one of the most important strategies for transferring model knowledge from one domain to another [49]. During fine-tuning, the weights of some layers of pre-trained models are preserved and the rest of the layers are retrained (i.e., “fine-tuned”) [50]. The concepts of direct transfer and fine-tuning are illustrated in Figure 5. It can be seen from the schematic in Figure 5 that the weights of the intermediate layers of the pre-trained models are frozen while the rest of the layers are retrained in the fine-tuning strategy, whereas no retraining is performed in the direct transfer approach. For our work here, in the direct transfer strategy, the models are trained from scratch on dataset 2, and then the trained model is evaluated with test samples from dataset 4. Similarly, the models trained on dataset 4 are evaluated on the test images from dataset 2. In the fine-tuning strategy, models are trained from scratch on dataset 2, afterwards they are partly retrained on dataset 4 and also tested on dataset 4. Or the other way round: Complete initial training on dataset 4, afterwards fine-tuning on dataset 2 and finally evaluation on dataset 2. In this work, we performed these different training strategies with the SwinT-fairSIM, Red-fairSIM, and UNet-fairSIM algorithms [19] with datasets 2 and 4.

To perform fine-tuning on the SwinT-fairSIM algorithm, we retrained the head and tail blocks along with one adjacent RSTB block each. By doing so, about 1.9 million training parameters stayed frozen out of 4 million parameters. Regarding the CNN-based algorithms from our previous work [19], we used the following approaches to fine-tuning: Red-fairSIM is based on the residual-encoder-decoder network (RED-Net) [51]. In the fine-tuning approach for Red-fairSIM, we retrained the first and last 5 layers of the model instead of all 30 layers. By doing so, about 700K training parameters stayed frozen out of more than 1 million parameters. UNet-fairSIM is based on the

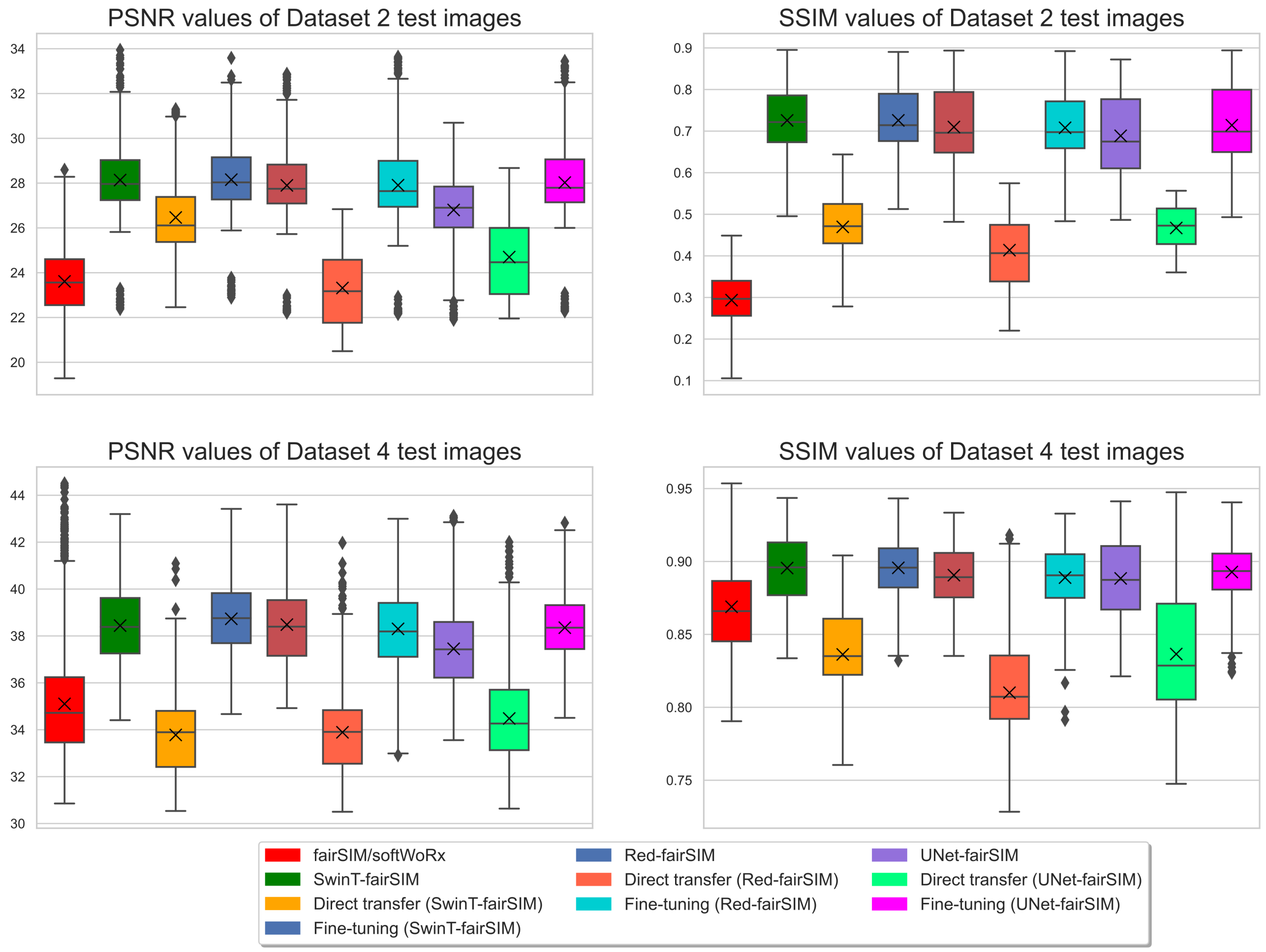

Figure 7. Boxplots of PSNR and SSIM values of test samples of dataset 2 and dataset 4 with different denoising algorithms and training strategies. The test data contains 500 images from noise level 4 for dataset 2 and 1380 images for dataset 4. The horizontal line inside each box plot represent the median value, whereas, the black cross display the mean values. The diamond-shaped black markers display the outlier observations. The color of the boxes in each subplot refers to the methods defined in the legend box.

UNet architecture which is based on several encoder and decoder blocks [52]. In the fine-tuning of UNet-fairSIM, we simply retrained the first and last two encoder and decoder blocks; in doing so, more than 29 million parameters out of 33 million parameters stayed frozen.

Generally, in the first step, all these algorithms were trained from scratch on datasets 2 and 4 separately for 100 epochs. The mean squared error (MSE) was used as a loss function for all training runs with the Adam optimizer. The learning rate was set to 1×10^{-4} for all training runs. In the next step, we applied direct transfer by propagating the test samples from the respective dataset not used for training the pre-trained models. In direct transfer, the test images of the dataset 2 (i.e., tubulin filaments) were propagated through the pre-trained model trained on the dataset 4 (i.e., vesicle structure) and vice versa. In the third step, the pre-trained models were fine-tuned using training data from the respective dataset not used for initial training. 30 training epochs were used for fine-tuning. The fine-tuned models were then evaluated on the test samples from the dataset used in the fine-tuning process. When fine-tuning the pre-trained models, we initially kept different numbers of layers/blocks frozen. However, we found that the best results were obtained by fine-tuning rather few trainable parameters for 30 epochs (except for SwinT-fairSIM, where a larger part of the network had to be "unfrozen"). In the final step, we compared the results of conventional training with the direct transfer and fine-tuning strategies visually, as well as in terms of peak signal-to-noise ratio (PSNR) [53] and structural similarity index measurement (SSIM) [54] values.

Results

We collected four data sets for denoising and super-resolution tasks. The overview and full characteristics of these datasets are shown in Table 1. The images in dataset 2 contain the tubulin structure along with mixed Poisson-Gaussian (MPG) noise and SR-SIM reconstruction artifacts. The images in dataset 4 contain the vesicle structure along with MPG noise and honeycomb pattern artifacts that arise when raw data carrying predominantly Poisson noise is subjected to the frequency-based SR-SIM reconstruction algorithm, which then introduces reconstruction artifacts [14]. In our previous studies [19], we already used the first three datasets for the denoising and super-resolution tasks. Here, we mainly focus on the denoising of SR-SIM images by SwinTransfomer-fairSIM and compare the results of this new method with the results of Red-fairSIM and UNet-fairSIM [19]. We also analyze two transfer learning strategies, direct transfer and fine-tuning of the pre-trained models of these network architectures.

To compare the performance of conventional training of these deep learning-based denoising methods with direct transfer and fine-tuning, we first train the SwinT-fairSIM,

Table 2. Mean PSNR and SSIM values along with standard deviations (STD) of all experiments calculated on the noisy test images of datasets 2 and 4 (for dataset 2, noise level 4 was used). The test data contains 500 images for dataset 2 and 1380 images for dataset 4. The PSNR and SSIM values are calculated relative to the reference images, i.e. SR-SIM images reconstructed with fairSIM/softWoRx from raw SIM images with the highest signal-to-noise ratio. In the row entitled "fairSIM/softWoRx", PSNR and SSIM values are calculated for the direct fairSIM/softWoRx reconstruction of the noisy images. The rows entitled "SwinT-fairSIM","Red-fairSIM" and "UNet-fairSIM" show the results for the denoised test images after conventional training from scratch with the respective algorithms. In the direct transfer rows, the mean PSNR and SSIM values in the column "dataset 2" are calculated for models which are initially trained on dataset 4 and afterwards tested with the test samples from dataset 2. For the column "dataset 4", this is the other way round. The same principle holds for the rows for fine-tuning, only that the models are fine-tuned on the respective dataset before testing.

	Mean PSNR (STD) and SSIM (STD) values of test data			
	**dataset =2**		**dataset =4**	
	PSNR (STD)	SSIM (STD)	PSNR (STD)	SSIM (STD)
fairSIM/softWoRx	23.61 (1.54)	0.29 (0.07)	35.10 (2.71)	0.86 (0.03)
SwinT-fairSIM	28.19 (2.09)	0.72 (0.09)	38.44 (1.73)	0.89 (0.02)
Direct transfer (SwinT-fairSIM)	26.07 (1.91)	0.48 (0.07)	34.05 (2.24)	0.83 (0.03)
Fine-tuning (SwinT-fairSIM)	28.15 (1.87)	0.72 (0.08)	38.52 (1.50)	0.89 (0.02)
Red-fairSIM	27.97 (2.01)	0.71 (0.09)	38.43 (1.45)	0.89 (0.01)
Direct transfer (Red-fairSIM)	23.31 (1.68)	0.41 (0.07)	33.89 (1.93)	0.81 (0.03)
Fine-tuning (Red-fairSIM)	27.90 (2.14)	0.70 (0.09)	38.30 (1.60)	0.88 (0.02)
UNet-fairSIM	26.80 (1.65)	0.68 (0.10)	37.45 (1.79)	0.88 (0.02)
Direct transfer (UNet-fairSIM)	24.69 (1.54)	0.46 (0.05)	34.47 (2.24)	0.83 (0.04)
Fine-tuning (UNet-fairSIM)	28.02 (1.97)	0.71 (0.07)	38.35 (1.44)	0.89 (0.01)

Red-fairSIM, and UNet-fairSIM networks separately with datasets 2 and 4 for 100 epochs. The results of all methods trained separately with both datasets are shown visually in Figure 6 and quantitatively in Table 2. The visual and quantitative results of SwinT-fairSIM show superiority over the Red-fairSIM and UNet-fairSIM methods. The resulting ROIs of both datasets from SwinT-fairSIM are more appealing and sharper than those of the other methods in Figure 6. The PSNR and SSIM values of SwinT-fairSIM are also slightly higher than those of Red-fairSIM and UNet-fairSIM. Overall, SwinT-fairSIM outperforms its counterparts on both datasets 2 and 4 after conventional training from scratch.

In the next step, we evaluate the direct transfer strategy by using these pre-trained models. In direct transfer, the model initially trained with dataset 2 is tested with the test samples of an alternative dataset (i.e., dataset 4), and vice versa. Similarly, in the assessment of the fine-tuning strategy, we retrained the first and last layers of the pre-trained models with the alternative data to see the improvement in the generalization power of the pre-trained models. The resulting denoised images obtained by direct transfer of models trained on vesicle structures (dataset 4) to test images containing tubulin filaments (dataset 2) are displayed within block **(a)** of Figures 8 and 9. These images clearly show that the model trained on a specific type of noise and structure (i.e., vesicle images with honey comb pattern noise) is not able to produce a refined denoised image of another structure (i.e., tubulin filaments with MPG noise). Similarly, the models initially trained on tubulin filaments from dataset 2 are not able to properly produce the denoised images of dataset 4 with different structure and noise (see block **(b)** of Figures 8 and 9). However, it can be noticed that the pre-trained models try to replicate the filamentous structure of tubulin in the vesicle data (very prominent in block **(b)** of Figure 8). This is a clear indication that these deep learning based denoising models are not robust against different types of noise and structure. However, the outcomes of the fine-tuning approach are promising as shown in Figures 8 and 9. The ROIs 1 and 2 in column 6 of Figure 8 and also the ROIs in column 4 of Figure 9 show that the results of the fine-tuning strategy are very close to the results of training from scratch for the SwinT-fairSIM, Red-fairSIM and UNet-fairSIM algorithms. The overall comparison of all the approaches in Figures 8 and 9 demonstrates that the fine-tuning method is crucial for the Transformer and CNN-based denoising algorithms in the case of changes in structure or noise types. Thus, fine-tuning is inevitably required to profit from knowledge transfer.

Table 2 lists the average PSNR and SSIM values of the test images of the datasets 2 and 4 calculated for all the training and testing strategies of SwinT-fairSIM, Red-fairSIM, and UNet-fairSIM. Table 2 clearly points out the decline in the average PSNR and SSIM values on both datasets 2 and 4 after the application of direct transfer. However, the average PSNR and SSIM values show considerable improvement after fine-tuning. The average PSNR and SSIM values of the fine-tuned models in Table 2 are very close to the models that were trained from scratch (partly slightly better, partly slightly worse). In addition, Figure 7 shows boxplots of the PSNR and SSIM values of the test samples of both datasets with all different methods/strategies. The boxplots complement the mean values in Table 2 by depicting additional statistics. It can be observed that SwinT-fairSIM not only has often higher mean and median values, but also a more consistent interquartile range in all of the boxplots in Figure 7. In the SSIM boxplots of dataset 2, very few outliers are noticable overall.

Compared to conventional training, the fine-tuning strategy retrains only a subset of the entire set of model parameters. In this study, we retrained about 2.1 million parameters out of 4 million parameters of SwinT-fairSIM, while for Red-fairSIM and UNet-fairSIM, out of more than 1 million and 33 million training parameters, only 295K and 3.7 million parameters were retrained, respectively. We were able to achieve re-

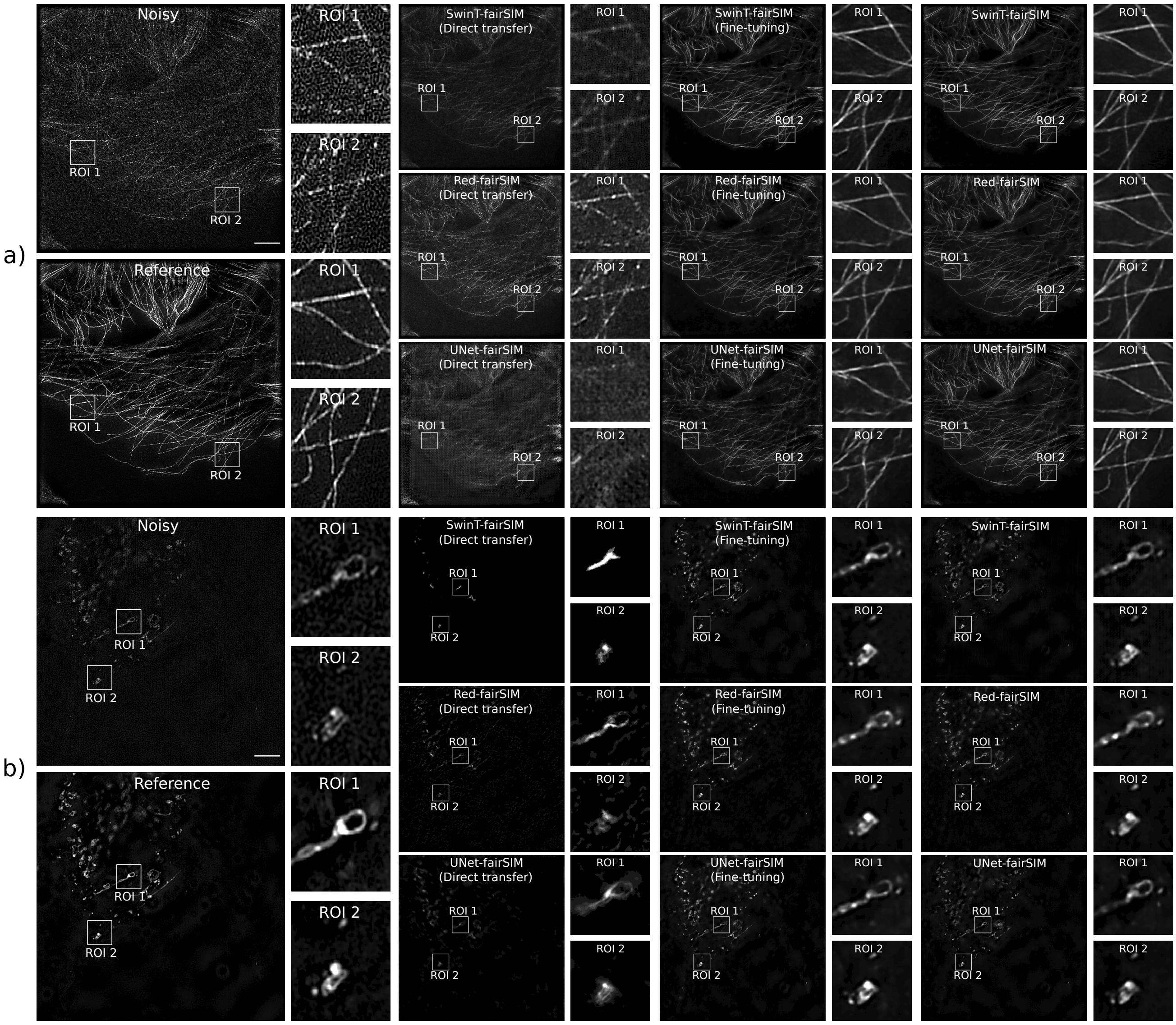

Figure 8. Blocks (**a**) and (**b**) show the results for test samples from dataset 2 (block **a**) and dataset 4 (block **b**) for different algorithms and learning strategies. The first and second columns of blocks **(a)** and **(b)** show the noisy and reference SR-SIM images, respectively, along with extracted and magnified regions of interest (ROIs). The noisy images are the test input for all algorithms. Columns 3 to 6 show the results of direct transfer and fine-tuning with SwinT-fairSIM, Red-fairSIM, and UNet-fairSIM. In direct transfer, the model is trained with dataset 2 and tested with the test sample of dataset 4 and vice versa. Similarly, in fine-tuning, the model is first pre-trained on dataset 2 and then fine-tuned and tested on dataset 4 and vice versa. Columns 7 and 8 show the results of all algorithms when trained from scratch on the respective dataset, which is also used for testing. The fourth, sixth, and eighth columns show the cropped and enlarged ROIs from the full-size SR-SIM images to the left of each ROI. The ROIs of size 100 pixels × 100 pixels have been upsampled to 300 pixels × 300 pixels for illustration purposes. Scale bar: 4 µm.

sults comparable to those of conventional training after only 30 epochs as opposed to 100 epochs. Training times for dataset 2 on two Nvida V100 GPUs (32 GB) were (training from scratch vs. fine-tuning):

SwinT-fairSIM: 26 h vs. 3.25 h
Red-fairSIM: 24 h vs. 3 h
UNet-fairSIM: 18 h vs. 1.5 h

Therefore, in terms of computational requirements or memory consumption, it is advantageous to use fine-tuning instead of training from scratch. Fine-tuning is beneficial when the computational resources are limited.

Discussion

In summary, the contribution of this work is threefold: First, we publish novel datasets related to SIM microscopy, then we explore a Transformer-based algorithm for the restoration of SR-SIM images. Finally, we investigate the potential of the direct transfer and fine-tuning strategies for various deep learning-based denoising algorithms. Regarding datasets, we provide four novel datasets for testing denoising and super-resolution image reconstruction strategies with tubulin filaments and vesicle structures. These datasets contain a large number of raw SIM images and reconstructed twofold super-resolved SIM images and cover different noise levels, a wide range of fields of view, and varying degrees of structural complexity. High-quality data from the real world of microscopy are highly relevant for benchmarking and evaluating current and upcoming denoising and super-resolution methods.

Regarding our work with Transformers, we suggested the SwinT-fairSIM architecture to produce high-quality SR-SIM images from low SNR inputs. Importantly, we visually and quantitatively showed that the Transformer-based method can achieve mostly better results than the CNN-based Red-fairSIM

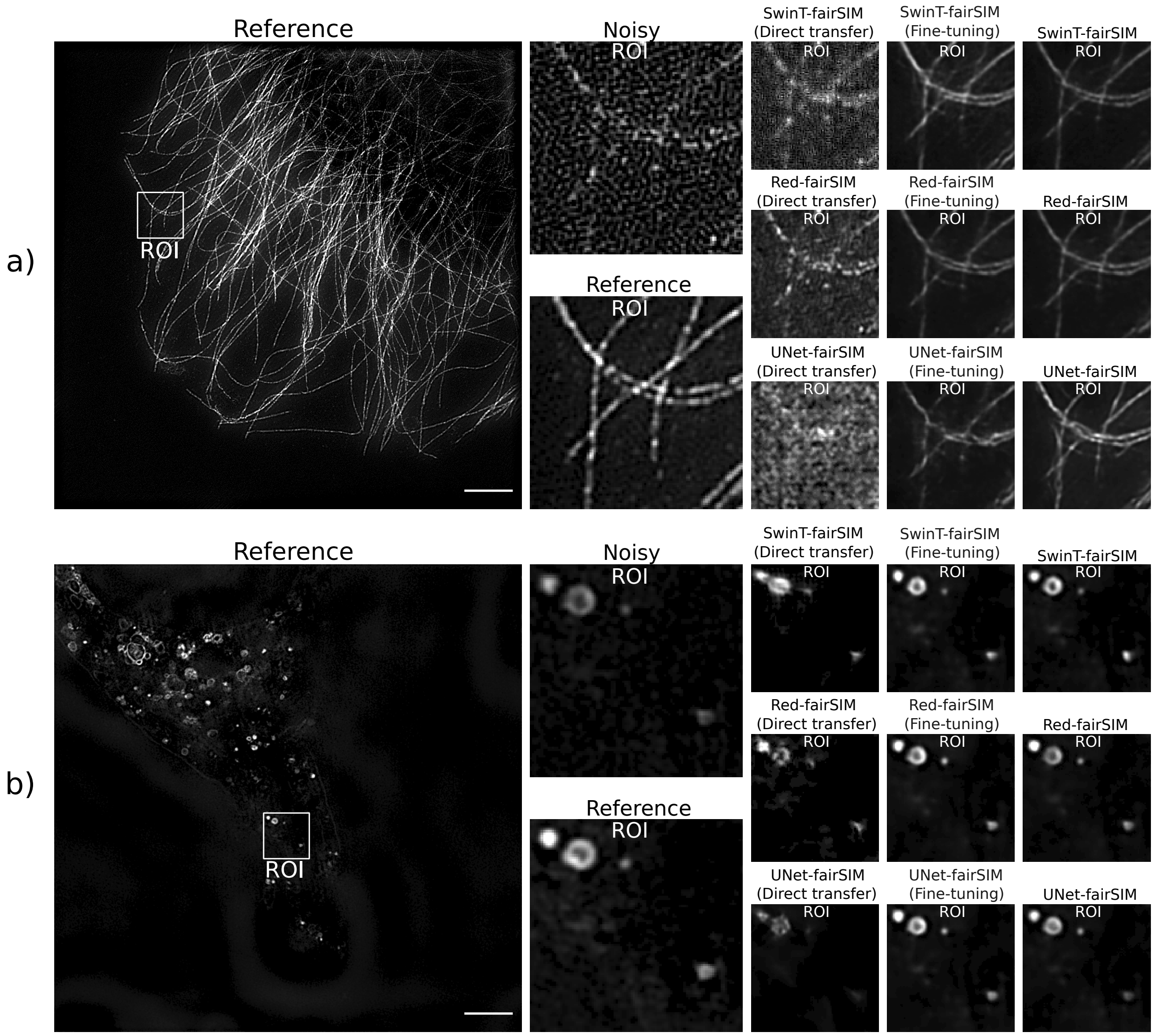

Figure 9. Two more test samples from datasets 2 and 4 are shown in this figure. Both blocks (**a**) and (**b**) contain the reference SR-SIM images along with the resultant denoised ROIs which are extracted from the full size denoised images of all the methods used in this work. The extracted ROIs are upsampled from 100 pixels × 100 pixels to 300 pixels × 300 pixels for illustration purposes. Scale bar: 4 µm.

and UNet-fairSIM methods (i.e., for the quantitative results, PSNR and SSIM values, see Table 2 and the boxplots in Figure 7). We demonstrated that SwinT-fairSIM can retrieve more well-preserved cell structures and texture information than the CNN-based methods, especially in the ROIs of Figure 6. This is an important finding, since most existing SR-SIM restoration methods are based on CNNs [17, 18, 19, 21, 22, 23, 38, 39]. An explanation for the better performance of SwinT-fairSIM may be the size of the receptive fields of the different network architectures. In CNNs, the size of their receptive fields is limited by the size and stride of the filter kernels and the depth of the network. In contrast, Swin Transformers may obtain a larger receptive field by the shifted windows operation [55, 56].

In the last section of this work, we showed the limitations of Transformer- and CNN-based deep learning methods in the area of knowledge transfer when it comes to different types of noise and structure. In the direct transfer strategy, we simply evaluated the pre-trained networks with new test data. The direct transfer strategy exhibits very downgraded visual (in Figures 8 and 9) and quantitative (in Table 2 and Figure 7) results. However, the approach of fine-tuning enables these deep learning models to generalize well to other noise types and structures after retraining few layers. This holds for all learning algorithms and datasets in a similar way as shown in Figures 7, 8, 9 and in Table 2. All of the deep neural network architectures tested here are, at their core, encoder-decoder architectures (for SwinT-fairSIM, this applies at least to the compression head block and the decompression tail block). In these architectures, the initial and final layers are responsible for handling low-level features. Noise patterns can be interpreted as low-level features that are separated from the valuable input during the encoding process so that they can be selectively suppressed at a later stage during decoding. Accordingly, we retrained only the initial and final layers of all three types of neural networks to achieve results on par with training from scratch. It is worth mentioning that we also attempted to retrain only the first, middle, or last layers during first exploratory research, and also varied the number of retrained layers, but the results obtained in this way were not competitive. Since fine-tuning often saves computation time and often requires fewer learning samples, we conclude that fine-tuning of pre-trained models has advantages over conventional train-

ing from scratch, at least when the difference between the task domains is not huge. In contrast, direct transfer obviously fails because the learned detectors for low-level features (noise patterns and typical simple visual features of the main cell structures) are too much tuned to the original training data.

There are still some limitations of this work. First, while our datasets are useful for various image restoration tasks and contain more samples than many of the datasets previously published in the field, they are limited to two structures, tubulin filaments and vesicle structures. Second, our results indicate the superiority of SwinT-fairSIM over Red-fairSIM and UNet-fairSIM. However, this improvement leads to higher computational costs. Third, fine-tuning strategies provide similar results to training from scratch at lower cost, but it is often difficult to find task-specific pre-trained networks.

Conclusion

In this work, we first presented a series of datasets for SIM image restoration tasks such as denoising and super-resolution. Additionally, we proposed a new algorithm based on the Swin Transformer, "SwinT-fairSIM", for the denoising of SR-SIM images. We showed that this Transformer-based algorithm outperforms CNN-based denoising algorithms both visually and quantitatively. This suggests that Transformer-based denoising algorithms can play an important role in the field of SR-SIM microscopy. Similarly, we evaluated different knowledge transfer strategies such as direct transfer and fine-tuning for Transformer- and CNN-based denoising algorithms. For the direct transfer strategy, we noticed a decline in the performance of these algorithms. However, we were able to recover the declined performance by the fine-tuning strategy. This clearly indicates that the Transformer- and CNN-based denoising methods require retraining of some initial and final layers of the pre-trained models when applied to new biological structures and noise types. This retraining requires fewer epochs than the conventional training from scratch. This holds also for the novel SwinT-fairSIM denoising algorithm which outperforms all CNN-based algorithm also in the fine-tuning regime.

Potential implications

The implementation of different deep learning methods requires a large quantity of images in order to train the underlying models. We believe that our published datasets will help the research community to develop new deep learning based methods and evaluate the existing methods by either training from scratch or by applying fine-tuning. These datasets can be especially used for image denoising and super-resolution tasks.

Data and code Availability

All the raw and reconstructed datasets which are discussed in this article are available on GigaDB ["link will be uploaded after the allocation of space from Giga Database"]. All the datasets are distributed under the Creative Commons CCO waiver, with a request for attribution. The CCO permits the unrestricted reuse, distribution and reproduction, provided the original work is properly cited. Similarly, all the codes which are used during this work are publicly available [42].

Declarations

Abbreviations

SIM: structured illumination microscopy; SR-SIM: super-resolution structured illumination microscopy; NA: numerical aperture; PSNR: peak-signal-to-noise ratio; SSIM: structural similarity index measurement; MSE: mean square error; SNR: signal-to-noise ratio; W2S: wide-field2SIM; fairSIM: free analysis and interactive reconstruction for structured illumination microscopy.

Competing Interests

The authors declare no competing interests.

Funding

This work was supported by the EFRE-NRW funding programme "Forschungsinfrastrukturen" (grant no. 34.EFRE-0300180) and partly conducted within the framework of the project "SAIL: SustAInable Lifecycle of Intelligent SocioTechnical Systems" (grant no. NW21-059B). SAIL is receiving funding from the programme "Netzwerke 2021", an initiative of the Ministry of Culture and Science of the State of Northrhine Westphalia. T.-C.W. and T.H. were supported by funding from the European Union's Horizon 2020 research and innovation program under the Marie Sklodowska-Curie Grant Agreements No. 642157, project "TOLLerant", and No. 766181. project "DeLIVER". T.H. also acknowledges funding by the Deutsche Forschungsgemeinschaft (DFG, German Science Foundation)—project number 415832635.

Author's Contributions

Z.H.S. carried out the preprocessing of datasets for the machine learning work, created the figures, and wrote the manuscript. D.T. conducted experiments to find the optimal hyperparameters for the CNN-based approaches. T.-C.W. recorded the raw SIM images of dataset 1. M.M. supported Z.H.S. in the reconstruction of data for datasets 2 and 3 with fairSIM. W.H. recorded and reconstructed the images of dataset 4. W.S. and T.H. supervised the research. W.S., T.H., M.M., and W.H. contributed to the editing of the manuscript. In addition, they discussed the experimental results together with Z.H.S. All authors discussed and agreed on the final manuscript.

Acknowledgements

The authors would like to thank Dr. Matthias Fricke from the Center for Applied Data Science (CfADS) at Bielefeld University of Applied Sciencesn and Arts for providing access to their GPU compute cluster. We would also like to thank Dr. Olaf Kaczmarek and Markus Klappenback for providing access to the GPU compute cluster at Bielefeld University.

References

1. Hirvonen LM, Wicker K, Mandula O, Heintzmann R. Structured illumination microscopy of a living cell. European Biophysics Journal 2009;38(6):807–812.
2. Hell SW, Sahl SJ, Bates M, Zhuang X, Heintzmann R, Booth MJ, et al. The 2015 super-resolution microscopy roadmap. Journal of Physics D: Applied Physics 2015;48(44):443001.

3. Heintzmann R, Huser T. Super-resolution structured illumination microscopy. Chemical reviews 2017;117(23):13890–13908.
4. Demmerle J, Innocent C, North AJ, Ball G, Müller M, Miron E, et al. Strategic and practical guidelines for successful structured illumination microscopy. Nature protocols 2017;12(5):988–1010.
5. Schermelleh L, Ferrand A, Huser T, Eggeling C, Sauer M, Biehlmaier O, et al. Super-resolution microscopy demystified. Nature cell biology 2019;21(1):72–84.
6. Gustafsson MG. Surpassing the lateral resolution limit by a factor of two using structured illumination microscopy. Journal of microscopy 2000;198(2):82–87.
7. Müller M, Mönkemöller V, Hennig S, Hübner W, Huser T. Open-source image reconstruction of super-resolution structured illumination microscopy data in ImageJ. Nature communications 2016;7(1):1–6.
8. Lal A, Shan C, Xi P. Structured illumination microscopy image reconstruction algorithm. IEEE Journal of Selected Topics in Quantum Electronics 2016;22(4):50–63.
9. Brown PT, Kruithoff R, Seedorf GJ, Shepherd DP. Multicolor structured illumination microscopy and quantitative control of polychromatic light with a digital micromirror device. Biomedical Optics Express 2021;12(6):3700–3716.
10. Ströhl F, Kaminski CF. Frontiers in structured illumination microscopy. Optica 2016;3(6):667–677.
11. Zheng X, Zhou J, Wang L, Wang M, Wu W, Chen J, et al. Current challenges and solutions of super-resolution structured illumination microscopy. APL Photonics 2021;6(2):020901.
12. Huang X, Fan J, Li L, Liu H, Wu R, Wu Y, et al. Fast, long-term, super-resolution imaging with Hessian structured illumination microscopy. Nature biotechnology 2018;36(5):451–459.
13. Hoffman DP, Betzig E. Tiled reconstruction improves structured illumination microscopy. BioRxiv 2020;.
14. Smith CS, Slotman JA, Schermelleh L, Chakrova N, Hari S, Vos Y, et al. Structured illumination microscopy with noise-controlled image reconstructions. Nature methods 2021;18(7):821–828.
15. Gustafsson MG, Shao L, Carlton PM, Wang CR, Golubovskaya IN, Cande WZ, et al. Three-dimensional resolution doubling in wide-field fluorescence microscopy by structured illumination. Biophysical journal 2008;94(12):4957–4970.
16. Shah ZH, Müller M, Hammer B, Huser T, Schenck W. Impact of different loss functions on denoising of microscopic images. In: 2022 International Joint Conference on Neural Networks (IJCNN) IEEE; 2022. p. 1–10.
17. Jin L, Liu B, Zhao F, Hahn S, Dong B, Song R, et al. Deep learning enables structured illumination microscopy with low light levels and enhanced speed. Nature communications 2020;11(1):1–7.
18. Chen X, Li B, Jiang S, Zhang T, Zhang X, Qin P, et al. Accelerated Phase Shifting for Structured Illumination Microscopy based on Deep Learning. IEEE Transactions on Computational Imaging 2021;7:700–712.
19. Shah ZH, Müller M, Wang TC, Scheidig PM, Schneider A, Schüttpelz M, et al. Deep-learning based denoising and reconstruction of super-resolution structured illumination microscopy images. Photonics Research 2021;9(5):B168–B181.
20. Belthangady C, Royer LA. Applications, promises, and pitfalls of deep learning for fluorescence image reconstruction. Nature methods 2019;16(12):1215–1225.
21. Qiao C, Li D, Guo Y, Liu C, Jiang T, Dai Q, et al. Evaluation and development of deep neural networks for image super-resolution in optical microscopy. Nature Methods 2021;18(2):194–202.
22. Xypakis E, Gosti G, Giordani T, Santagati R, Ruocco G, Leonetti M. Deep learning for blind structured illumination microscopy. Scientific Reports 2022;12(1):8623.
23. Liu T, Liu J, Li D, Tan S. Improving Reconstruction of Structured Illumination Microscopy Images Via Dual-Domain Learning. IEEE Journal of Selected Topics in Quantum Electronics 2023;.
24. Qiao C, Chen X, Zhang S, Li D, Guo Y, Dai Q, et al. 3D structured illumination microscopy via channel attention generative adversarial network. IEEE Journal of Selected Topics in Quantum Electronics 2021;27(4):1–11.
25. Vaswani A, Shazeer N, Parmar N, Uszkoreit J, Jones L, Gomez AN, et al. Attention is all you need. Advances in neural information processing systems 2017;30.
26. Sutskever I, Vinyals O, Le QV. Sequence to sequence learning with neural networks. Advances in neural information processing systems 2014;27.
27. Dehghani M, Gouws S, Vinyals O, Uszkoreit J, Kaiser L. Universal Transformers. In: International Conference on Learning Representations;. .
28. Vyas A, Katharopoulos A, Fleuret F. Fast transformers with clustered attention. Advances in Neural Information Processing Systems 2020;33:21665–21674.
29. Wang B, Shang L, Lioma C, Jiang X, Yang H, Liu Q, et al. On position embeddings in bert. In: International Conference on Learning Representations; 2021. .
30. Cheng X, Lin H, Wu X, Shen D, Yang F, Liu H, et al. Mltr: Multi-label classification with transformer. In: 2022 IEEE International Conference on Multimedia and Expo (ICME) IEEE; 2022. p. 1–6.
31. Carion N, Massa F, Synnaeve G, Usunier N, Kirillov A, Zagoruyko S. End-to-end object detection with transformers. In: Computer Vision–ECCV 2020: 16th European Conference, Glasgow, UK, August 23–28, 2020, Proceedings, Part I 16 Springer; 2020. p. 213–229.
32. Sun Z, Cao S, Yang Y, Kitani KM. Rethinking transformer-based set prediction for object detection. In: Proceedings of the IEEE/CVF international conference on computer vision; 2021. p. 3611–3620.
33. Chen H, Wang Y, Guo T, Xu C, Deng Y, Liu Z, et al. Pre-trained image processing transformer. In: Proceedings of the IEEE/CVF Conference on Computer Vision and Pattern Recognition; 2021. p. 12299–12310.
34. Ali AM, Benjdira B, Koubaa A, El-Shafai W, Khan Z, Boulila W. Vision transformers in image restoration: A survey. Sensors 2023;23(5):2385.
35. Liu Z, Lin Y, Cao Y, Hu H, Wei Y, Zhang Z, et al. Swin transformer: Hierarchical vision transformer using shifted windows. In: Proceedings of the IEEE/CVF international conference on computer vision; 2021. p. 10012–10022.
36. Liang J, Cao J, Sun G, Zhang K, Van Gool L, Timofte R. Swinir: Image restoration using swin transformer. In: Proceedings of the IEEE/CVF international conference on computer vision; 2021. p. 1833–1844.
37. Zhang Y, Zhu Y, Nichols E, Wang Q, Zhang S, Smith C, et al. A poisson-gaussian denoising dataset with real fluorescence microscopy images. In: Proceedings of the IEEE/CVF Conference on Computer Vision and Pattern Recognition; 2019. p. 11710–11718.
38. Zhou R, Helou ME, Sage D, Laroche T, Seitz A, Süsstrunk S. W2S: microscopy data with joint denoising and super-resolution for widefield to SIM mapping. In: European Conference on Computer Vision Springer; 2020. p. 474–491.
39. Hagen GM, Bendesky J, Machado R, Nguyen TA, Kumar T, Ventura J. Fluorescence microscopy datasets for training deep neural networks. GigaScience 2021;10(5):giab032.

40. Karras C, Smedh M, Förster R, Deschout H, Fernandez-Rodriguez J, Heintzmann R. Successful optimization of reconstruction parameters in structured illumination microscopy–a practical guide. Optics Communications 2019;436:69–75.
41. Mueller M, Free Analysis and Interactive Reconstruction for Structured Illumination Microscopy. GitHub; 2016. https://github.com/fairSIM/fairSIM.git.
42. Shah ZH, Evaluation of Swin Transformer and knowledge transfer for denoising of super-resolution structured illumination microscopy data. GitHub; 2023. https://github.com/ZafranShah/Denoising_and_fine_tuning_of_SR-SIM_data.
43. Shah ZH, Evaluation of Swin Transformer and knowledge transfer for denoising of super-resolution structured illumination microscopy data. zenodo; 2023. https://doi.org/10.5281/zenodo.7626173.
44. Ching JY, Wong AKC, Chan KCC. Class-dependent discretization for inductive learning from continuous and mixed-mode data. IEEE Transactions on Pattern Analysis and Machine Intelligence 1995;17(7):641–651.
45. Hussain M, Bird JJ, Faria DR. A study on cnn transfer learning for image classification. In: UK Workshop on computational Intelligence Springer; 2018. p. 191–202.
46. Tan C, Sun F, Kong T, Zhang W, Yang C, Liu C. A survey on deep transfer learning. In: International conference on artificial neural networks Springer; 2018. p. 270–279.
47. Bengio Y. Deep learning of representations for unsupervised and transfer learning. In: Proceedings of ICML workshop on unsupervised and transfer learning JMLR Workshop and Conference Proceedings; 2012. p. 17–36.
48. Ng HW, Nguyen VD, Vonikakis V, Winkler S. Deep learning for emotion recognition on small datasets using transfer learning. In: Proceedings of the 2015 ACM on international conference on multimodal interaction; 2015. p. 443–449.
49. Nogueira K, Penatti OA, Dos Santos JA. Towards better exploiting convolutional neural networks for remote sensing scene classification. Pattern Recognition 2017;61:539–556.
50. Yosinski J, Clune J, Bengio Y, Lipson H. How transferable are features in deep neural networks? Advances in neural information processing systems 2014;27.
51. Mao X, Shen C, Yang YB. Image restoration using very deep convolutional encoder-decoder networks with symmetric skip connections. Advances in neural information processing systems 2016;29.
52. Ronneberger O, Fischer P, Brox T. U-net: Convolutional networks for biomedical image segmentation. In: International Conference on Medical image computing and computer-assisted intervention Springer; 2015. p. 234–241.
53. Hore A, Ziou D. Image quality metrics: PSNR vs. SSIM. In: 2010 20th international conference on pattern recognition IEEE; 2010. p. 2366–2369.
54. Setiadi DRIM. PSNR vs SSIM: imperceptibility quality assessment for image steganography. Multimedia Tools and Applications 2021;80(6):8423–8444.
55. Parmar N, Vaswani A, Uszkoreit J, Kaiser L, Shazeer N, Ku A, et al. Image transformer. In: International conference on machine learning PMLR; 2018. p. 4055–4064.
56. Ranftl R, Bochkovskiy A, Koltun V. Vision transformers for dense prediction. In: Proceedings of the IEEE/CVF International Conference on Computer Vision; 2021. p. 12179–12188.

Cover Letter for Editor Click here to access/download;Personal Cover;Cover_Rebuttal_GIGA-D-23-00044.pdf

Title: <u>Evaluation of Swin Transformer and knowledge transfer for denoising of super-resolution structured illumination microscopy data</u>

Journal Name: GigaScience

Manuscript Number: GIGA-D-23-00044

Dear Editor,

We thank you and the reviewers for the comments on our manuscript entitled, **"Evaluation of Swin Transformer and knowledge transfer for denoising of super-resolution structured illumination microscopy data"**. We appreciate the suggestions of the reviewers to strengthen our revised manuscript. We also hope that our edits and the responses we provide below satisfactorily address all your major and minor concerns. Furthermore, we have added in response to one of the reviewers a new Transformer-based SR-SIM image denoising algorithm. Therefore, we have updated the title of the manuscript accordingly. Because of this considerable extension of the presented work, we would like to request to change the category of the manuscript from technical note to research.

All the results at GigaDB are updated and the new code is uploaded to the GitHub repository. We believe that the manuscript is now suitable for publication in GigaScience.

Reviewer # 1

Comment-1. To our knowledge, the "BioSR" dataset presented in reference [22] contains quite a few SIM reconstructions. While, the authors declare that "However, their collection of datasets consists of considerably fewer images and does not contain any super-resolution SIM images". The authors may do a detailed survey of the "BioSR" data set.

Response-1. We revised the text in this paragraph to clarify our statements and to provide some additional details about the BioSR dataset. Actually, the sentence "However, their collection of datasets consists of considerably fewer images and does not contain any super-resolution SIM images" was not intended to refer to the BioSR dataset. Hopefully, our writing is now better understandable.
As a side note: In comparison to the BioSR dataset collection with 2200 image pairs, our datasets consist of more than 9800 pairs of low- and high-resolution samples of 1024 x 1024 (width x height) pixels with different time stamps and noise levels. Furthermore, our datasets are ready to be used for different image restoration problems.

Comment -2. The authors may modify the bottom "(FFT)" in the "SIM Algorithm" box of Figure 1 as "(Inverse FFT)".

Response-2. We thank the reviewer and changed the respective figure.

Comment -3. We can see obvious artifacts and backgrounds in the reference images (Figure 2, magnified). Can the reference image quality be promoted further?

Response-3. All the reference images are generated using the fairSIM and SoftWorX reconstruction algorithms. These artifacts are generated by these SIM reconstruction algorithms, that is the reason we have developed our machine learning based tools that can be trained to remove such artifacts. The standard reference images can, however, not be further improved based on the traditionally used purely Wiener-filter-based algorithms. Due to this, we labeled our high-resolution output images as reference images instead of ground truth.

Comment -4. The UNet-fairSIM output in Figure 5b seems to be unreasonable and the authors may try to explain this phenomenon and solve the problem.

Response-4. The UNet-fairSIM method was not able to denoise that specific image properly but we have replaced that sample with another test sample in Figure 9 of the revised manuscript which is slightly more in favor of UNet. However, considering original Figure 5b and new Figure 9, the only condition

where the UNet is completely lacking is a direct transfer from training on dataset 4 (vesicles) to inference on dataset 2 (tubulin). In our view, this is not unreasonable. As the good results for this UNet for training from scratch on dataset 4 show, this UNet was properly trained before direct transfer. Therefore, the bad results for direct transfer mainly show that the UNet is not suited for this strategy.

Comment -5. Datasets 1-3 in Table 1 contain the same sample structures and may be combined into one dataset. By the way, the authors may provide other sample structures besides tubulin filaments and vesicles.

Response-5. The idea behind datasets 1-3 is to use these datasets of real world biological structures for different image restoration tasks. For example, dataset 1 can be used to denoise raw SIM data, dataset 2 can be used to denoise SR-SIM data, and dataset 3 can be used for super-resolution tasks or joint denoising and super-resolution tasks. In our view, this is a service to the potential user. The datasets are pre-structured in such a way that they can easily be used for these different tasks.

Comment -6. The authors can transfer Table 2 into the statistical boxplot for an intuitive representation.

Response-6. We thank the reviewer for this suggestion, we added the boxplots of the respective PSNR and SSIM values of all the findings in Figure 7 of the revised manuscript.

Comment -7. There are many deep learning-based SIM reconstruction methods like DFCAN/DFGAN, the authors may try these methods besides Red-fairSIM/Unet-fairSIM.

Response-7. In our revised manuscript, we propose a novel SwinT-fairSIM architecture based on the Swin Image Restoration (SwinIR) algorithm to denoise the SR-SIM images. Further details are explained in the revised manuscript (for details please see section "Shifted window Transformer for the denoising of SR-SIM images (SwinT-fairSIM)"). In this way, we hope to meet the demands of the reviewer by including an additional state-of-the-art deep learning-based image restoration technique in our comparison.

Comment -8. The authors may provide some discussions about "why the fine-tuning output is better than direct transfer?". Does this mean that the first/last few layers of the network are critical to its output?

Response-8. "Direct transfer" is the transfer of trained weights from one dataset to another dataset of the related task, whereas, in fine-tuning some of the layers of the pretrained models are retrained instead of training the entire model from scratch. We showed in the manuscript that direct transfer of weights from tubulin to vesicle data or vice versa results in a poor outcome. Fine-tuning is crucial to achieve competitive results. In the discussion section, we added a paragraph in which the critical role of the first/last few layers is discussed in light of these results.

Comment -9. The authors may provide a summary and conclusion about the work.

Response-9. We added the conclusion section in the revised manuscript.

Reviewer # 2:

Comment-1. In the abstract, on the computer vision side, there is a large number of methods that can blindly reduce the image noise level. Also, for SIM data, there are already methods for denoising multiple structures.

Response-1. In response to this comment, we added a sentence about a Blind-SIM method (BS-CNN architecture) in the second paragraph of the introduction section with reference number [22] to the revised manuscript. We agree that blind denoising is an interesting and relevant area on its own, but we think that it is beyond the scope of the presented work. Actually, in our previous work [19], we used a blind denoising algorithm from classical computer vision in comparison to deep learning-based approaches. The deep-learning approaches, trained to a specific task and data, were clearly superior. Therefore, we decided to focus here on algorithms which are trained in the first place with specific data and tackle the research questions, how well they generalize, and what can help in improving their generalization capabilities (i.e., fine-tuning).

Comment-2. On page 2, 'leads to low SNR', the abbreviation needs to be explained.

Response-2. We thank the reviewer, and we have addressed this concern in the revised manuscript.

Comment -3. On page 2, the authors compare existing open-source datasets and enumerate the amount of data in them, so it is better to introduce the dataset presented in this paper with the amount as well.

Response-3. We describe the characteristics of the datasets along with the total number of samples in Table 1 of the revised manuscript.

Comment -4. In SIM data, the data type is 16bit per pixel, however, the denoising method, i.e. RED was originally designed for 8bit images. In addition, the PSNR calculation in the author's code calls TensorFlow's built-in functions (tf_psnr）, has the corresponding modification been made for dynamic range, or has the data been pre-processed?

Response-4. We scaled the images from 16-bit to floating values (i.e., all the pixel values are between 0.0 and 1.0) before the training process. The PSNR values are calculated in the floating point

representation. The maximum intensity required as a parameter for the PSNR calculation is adjusted accordingly. After the prediction, the images are converted back to 16-bit for storage and visualization, etc.

Comment-5. The code is publicly available, but the data is not, and it is recommended that some data be made available for testing.

Response-5. We already submitted our data to the GigaScience Database, so that they are publicly available for the research community after acceptance of the manuscript.

Comment-6. How is the distance represented by each pixel (scale bar) derived in the image? For example, 80 nm in Table 1 and 8 μm in Fig.1 and 2. I suggest adding the corresponding explanation.

Response-6. 80nm and 40nm refers to the pixel sizes in the raw images (80 nm/pixel) and reconstructed images (40 nm/pixel) which are mentioned in Table 1 of the manuscript. 80 nm/pixel arises as the combination of the magnification of the optical system (microscope) and the physical pixel size of the camera. The SIM reconstruction process then doubles the number of pixels, which halves their size, as part of the frequency-spaced reconstruction step. 4μm refers to the length of the scale bars, which is set in relation to the pixel size (by a built-in ImageJ function). Scale bars are now consistently set to 4μm.

Comment -7. How is noise level defined? In the domain of machine learning, level denotes the standard deviation of Gaussian noise, which needs to be disambiguated here.

Response-7. The datasets which are presented in this manuscript are collected from a real biological sample and contain real noise which is a mixture of Gaussian-Poisson noise plus the reconstruction artifacts created by the SIM reconstruction algorithm. Therefore, these real datasets do not have any artificial or randomly added noise (such as Gaussian or Poisson with some specific intensity level). The real noise occurs due to the detection of fewer photons from biological samples, resulting in a bad signal-to-noise ratio. However, the timestamp is considered to be a relevant factor in these datasets to distinguish the level of noise in the images. For example, in datasets 1-3, small timestamps represent low noise levels, whereas large timestamps represent high noise levels as shown in Figure 3. The noise levels are explained in the subsection on “Dataset 2” in the revised manuscript.

Reviewer # 3:

Comment -1. Firstly, datasets 1-3 are based on 2D SIM reconstruction, whereas dataset 4 is based on 3D SIM reconstruction, which leads to different axial resolutions, making it difficult to test the generalization capability of the model.

Response-1. The raw data of all data sets are true 3D-SIM data acquisitions. The only difference is in the way how the reconstructions are obtained. fairSIM-based reconstructions use the 15 raw images of a particular focus position to reconstruct a single slice (which we call a 2D reconstruction), while the softWoRx reconstruction always reconstructs a full 3D data set. While a full 3D reconstruction provides higher axial resolution, optical sectioning, i.e. the capability of removing blurred out-of-focus light, can be and is achieved by both 2D and 3D reconstruction. More importantly, the lateral resolution increase is the same for 2D and 3D SIM reconstruction. This makes single-slice (2D) SIM reconstructions and full 3D SIM reconstructions similar enough to work in the same processing pipeline. During this work, we train only 2D models with both datasets 2 and 4. Each z-plane in dataset 4 is treated as a different field-of-view (FOV). This terminology results in the conversion of dataset 4 from a 3D to a 2D dataset. Furthermore, 15 raw SIM images were taken for each plane during the collection of all data, which consists of three pattern orientations and five equally distributed phases for each angle. This rules out the different axial resolution point.

Comment -2. Secondly, the noise level has only four levels, and the sample types are limited to two structures, which is significantly less than the 'BioSR' dataset cited as a reference in the manuscript.

Response-2. This concern is already addressed above (see Reviewer # 1, comment-1, and comment-5). The datasets provided by us have their own merits such as the large number of samples and being especially structured for common use cases in benchmarking and research.

Comment -3. Lastly, the authors need to provide a schematic of the fine-tuning strategies they used.

Response-3. We thank the reviewer for this suggestion; we now show the schematic of direct transfer and fine-tuning in Figure 5 of the revised manuscript.
