# Supplementary material for: Evaluation of Swin Transformer and knowledge transfer for denoising of super-resolution structured illumination microscopy data
Source: Gigascience. 2024 Jan 13;13:giad109. doi: 10.1093/gigascience/giad109 (PMC10787368; doi:10.1093/gigascience/giad109)
Supplement: giad109_GIGA-D-23-00044_Revision_2 [file giad109_giga-d-23-00044_revision_2.pdf]

GigaScience

Evaluation of Swin Transformer and knowledge transfer for denoising of super-resolution structured illumination microscopy data

--Manuscript Draft--

**Manuscript Number:**	GIGA-D-23-00044R2
**Full Title:**	Evaluation of Swin Transformer and knowledge transfer for denoising of super-resolution structured illumination microscopy data
**Article Type:**	Research
**Funding Information:**	H2020 Marie Skłodowska-Curie Actions (642157) — Mr. Thomas Huser<br>Deutsche Forschungsgemeinschaft (415832635) — Mr. Thomas Huser<br>EFRE-NRW funding programme Forschungsinfrastrukture (34.EFRE- 0300180) — Mr. Wolfram Schenck<br>SAIL: SustAInable Lifecycle of Intelligent SocioTechnical Systems funding programme Netzwerke 2021 (NW21-059B) — Mr. Wolfram Schenck
**Abstract:**	In recent years, convolutional neural network (CNN)-based methods have shown remarkable performance in the denoising and reconstruction of super-resolved structured illumination microscopy (SR-SIM) data. Therefore, CNN-based architectures have been the main focus of existing studies. Recently, however, an alternative and highly competitive deep learning architecture, Swin Transformer, has been proposed for image restoration tasks. In this work, we present SwinT-fairSIM, a novel method for restoring SR-SIM images with low signal-to-noise ratio (SNR) based on Swin Transformer. The experimental results show that SwinT-fairSIM outperforms previous CNN-based denoising methods. Furthermore, the generalization capabilities of deep learning methods for image restoration tasks on real fluorescence microscopy data have not been fully explored yet, i.e., the extent to which trained artificial neural networks are limited to specific types of cell structures and noise. Therefore, as a second contribution, we benchmark two types of transfer learning, i.e., direct transfer and fine-tuning, in combination with SwinT-fairSIM and two CNN-based methods for denoising SR-SIM data. Direct transfer does not prove to be a viable strategy, but fine-tuning achieves results comparable to conventional training from scratch, while saving computational time and potentially reducing the amount of required training data. As a third contribution, we publish four datasets of raw SIM images and already reconstructed SR-SIM images. These datasets cover two different types of cell structures, tubulin filaments and vesicle structures. Different noise levels are available for the tubulin filaments. These datasets are structured in such a way that they can be easily used by the research community for research on denoising, super-resolution, and transfer learning strategies.
**Corresponding Author:**	Zafran Hussain Shah<br>Bielefeld University of Applied Sciences: Fachhochschule Bielefeld<br>Bielefeld, North Rhine-Westphalia GERMANY
**Corresponding Author Secondary Information:**	
**Corresponding Author's Institution:**	Bielefeld University of Applied Sciences: Fachhochschule Bielefeld
**Corresponding Author's Secondary Institution:**	
**First Author:**	Zafran Hussain Shah
**First Author Secondary Information:**	
**Order of Authors:**	Zafran Hussain Shah<br>Marcel Müller, Doctorate

Powered by Editorial Manager® and ProduXion Manager® from Aries Systems Corporation

	Wolfgang Hübner, Doctorate
	Tung-Cheng Wang, Doctorate
	Daniel Telman, Bachelor
	Thomas Huser, Doctorate
	Wolfram Schenck, Doctorate
**Order of Authors Secondary Information:**	
**Response to Reviewers:**	Title: Evaluation of Swin Transformer and knowledge transfer for denoising of super-resolution structured illumination microscopy data<br><br>Journal Name: GigaScience<br>Manuscript Number: GIGA-D-23-00044<br><br>Dear Dr. Zauner,<br><br>Thank you once again for your and the reviewers' appreciation and valuable comments on our manuscript entitled, "Evaluation of Swin Transformer and knowledge transfer for denoising of super-resolution structured illumination microscopy data". We have carefully revised the manuscript according to the suggested changes. The changes in the manuscript text are highlighted by the yellow highlighter. The point-by-point responses are included in the response box.<br><br>Regarding the main concern of reviewer #3: We agree with you and reviewer #3 that the data acquired and processed using the 3D-SIM illumination and algorithm has higher axial resolution than 2D-SIM to some extent. However, the main objective of this work was to remove the noise and reconstruction artifacts from the lateral plane of 2D and 3D SIM data. The 2D slices were obtained from the 3D-SIM data using a slice-by-slice strategy. As a result, the network solely denoises the lateral planes of both types of data.<br><br>We are also grateful for your time and effort in discussing the matter with your experts. We hope that our responses address the concerns of the reviewers. We believe that the manuscript is now suitable for publication in GigaScience.<br><br>All the results at GigaDB are updated and the new code is uploaded to the GitHub repository.<br><br>#####Reviewer # 1#####<br><br>Comment #1: In the "Response-5", the authors declared that "dataset 1 can be used to denoise raw SIM data, dataset 2 can be used to denoise SR-SIM data, and dataset 3 can be used for super-resolution tasks or joint denoising and super-resolution tasks." While, dataset 1 contains the functionality provided by datasets 2-3 because the users can utilize the fairSIM and SoftWorX for reconstructing the SR-SIM images in datasets 2-3 from the raw SIM data in dataset 1. And the authors may provide other sample structures besides tubulin filaments and vesicles in the form of dataset 1.<br><br>Response #1: Thank you again for your review and constructive feedback. We believe your last suggestion is very useful, but our current circumstances limit us to these two available sample structures (i.e., tubulin filaments and vesicles). Nevertheless, we believe that these structures can be very useful for the development, evaluation, and validation of future Deep Learning-based image recovery methods in the field of SIM microscopy. In addition, we would like to point out that datasets 2 and 3 may be very useful for readers who come from the machine learning field and do not yet have experience using fairSIM or SoftWorX.<br><br>#####Reviewer # 2#####

Powered by Editorial Manager® and ProduXion Manager® from Aries Systems Corporation

Comment #1: The authors mentioned the pixel size in their response, but I would like to see a more detailed explanation in the paper about the relationship between pixel size and the external parameters of the microscope setup. This is crucial information for the dataset.

Response #1: Thank you for the review and the appreciation in your feedback. The choice of pixel size in a microscope depends on two main factors: the camera pixel size, and the total magnification. Higher resolutions are achieved by using smaller camera pixels and stronger magnifications, but the former at the expense of sensitivity (larger pixels are more sensitive) and the latter at the cost of field of view (higher magnifications decrease the imaging area). The OMX microscope uses a scientific camera for wide-field detection. Thus, the effective (aka projected) pixel size in the sample plane is given by the physical pixel size of the camera and the magnification of the optical system, i.e. the combination of the objective lens and any intermediate optics. For the OMX, the camera pixel size is 6.5 µm and the magnification is 82x, yielding an effective pixel size of 6.5 µm/82 = 80nm. The choice of magnification and thus effective pixel size is not arbitrary for a high-resolution imaging system. The imaging system has an inherent maximum spatial resolution that can be detected (Abbe limit), for a sample in water (n=1.33) and at blue wavelength (425nm) which is 160nm. Since detection by a pixelated camera is a discrete sampling process, the Nyquist criterion is now used to determine the maximum pixel size allowed to fully sample a signal with 160nm spatial resolution, which is 80nm. Larger pixels would miss high-frequency information that the optical system still transmits. Smaller pixels would fully sample all information, but introduce additional noise, as each pixel contributes an amount of read noise, and thus the total number of pixels should be kept small. More effects, especially dynamic range, can enterinto a pixel size determination, but this is the basic process. As a commercial microscope system, the pixel size of the OMX is constrained by the manufacturer to 80 nm, but it is likely the follow the above reasoning.

A shorter version of this explanation is now included in the manuscript at the beginning of “Materials and Methods – SR-SIM microscopy and sample preparation”.

Comment #2: If the noise was not added to the image, how were the variables controlled to ensure a consistent noise level in each capture? In low-light conditions, changes in camera parameters can significantly affect the level of noise in the captured images.

Response #2: Several imaging parameters such as exposure time and illumination intensity can be adjusted in SIM microscopy to obtain samples with a high signal-to-noise ratio. Higher photon counts (N) typically result in samples with a higher signal-to-noise ratio. In SIM imaging, we typically control for two types of noise: Poisson noise, that is the variation in the number of photons being converted into photo-electrons in each pixel, and thermal and read noise, summarizing the error in counting the photo-electrons in the digitization process. The latter, the error introduced every time when reading out the camera, is very reproducible as long as two parameters are controlled for Camera temperature (influencing thermal and sometimes read noise) and exposure time (influencing mostly thermal noise). In the data sets at hand, we thus kept constant exposure time and temperature of the camera system in the OMX by cooling liquid.
In contrast, Poisson noise is a property of both illumination intensity and the specific sample. We can and do control illumination intensity, but the sample-to-sample variations (different effectiveness of the staining, variation in photobleaching) cannot be controlled. Therefore, we believe that these variations should be part of the datasets, since they are to be expected in real data and denoising networks must be able to deal with them.

Some remarks addressing the different types of noise are now included in the manuscript at the end of “Materials and Methods – SR-SIM microscopy and sample preparation”.

Powered by Editorial Manager® and ProduXion Manager® from Aries Systems Corporation

	#####Reviewer # 3#####<br><br>Comment #1: In the revised version, the manuscript's quality has seen notable improvements, particularly due to the incorporation of results obtained using the Transformer-based SR-SIM image denoising algorithm. However, it remains concerning that the authors have not adequately addressed my comments, resulting in persisting weaknesses within the manuscript. One significant concern is that, regardless of whether 9 or 15 raw SIM images are employed in 2D reconstruction, the axial resolution consistently falls short compared to that achieved in 3D reconstruction. Consequently, the statement asserting that "this rules out the different axial resolution difference" is entirely inaccurate. Due to these unresolved issues, I am unable to ascertain the model's generalization capability across the four datasets. Moreover, the 'BioSR' dataset and other publicly available data showcasing new deep learning techniques for SIM denoising and reconstruction seem to overshadow the significance of the four datasets presented in this manuscript. Given the low image quality demonstrated within the manuscript, my confidence in the datasets' usefulness for the benefit of the scientific community has significantly diminished.<br><br>Response #1: Thank you for your review. Our previous statement "this rules out the different axial resolution difference" was unfortunately misleading. We fully agree that when a 3D SIM dataset is acquired and processed by 3D SIM algorithms, a higher axial resolution can be expected than for 2D SIM reconstruction (to some extent independent of the acquisition mode), since the axial resolution in SIM data depends on the illumination and the SIM reconstruction scheme [Gustafsson 2008]. Nevertheless, the SIM illumination and reconstruction algorithm for 3D SIM produces more noise and pattern artifacts in the lateral (XY) direction than in the axial (Z) direction [Gustafsson 2008]. Thus, a slice-by-slice (XY) strategy can still be useful to remove noise and artifacts introduced laterally by the reconstruction process for 3D SIM data.<br>In our work, we focus exclusively on the lateral plane and compare the generalization ability of denoising networks of 2D and 3D SIM microscopy data in the lateral plane using individual slices. An extension into the axial direction is out of the scope of the presented work and was not intended. We improved the manuscript by making this point clear at several locations:<br>• End of subsection “Dataset preprocessing and image reconstruction”<br>• Description of dataset 4<br>• New final sentence of the discussion section<br>As for the image quality in the datasets, we tend to disagree here, with all due respect. The images are of the usual quality that can be expected as the output of a SIM acquisition and processing toolchain for the intended cell structures. In particular, the reference images with high signal-to-noise ratios clearly show this.<br><br>#####Reference#####<br><br>[Gustafsson 2008]: Gustafsson, Mats GL, et al. "Three-dimensional resolution doubling in wide-field fluorescence microscopy by structured illumination." Biophysical journal 94.12 (2008): 4957-4970.
**Additional Information:**	
**Question**	**Response**
Are you submitting this manuscript to a special series or article collection?	No
**Experimental design and statistics**	Yes

Powered by Editorial Manager® and ProduXion Manager® from Aries Systems Corporation

Full details of the experimental design and statistical methods used should be given in the Methods section, as detailed in our Minimum Standards Reporting Checklist. Information essential to interpreting the data presented should be made available in the figure legends.<br><br>Have you included all the information requested in your manuscript?	
**Resources**<br><br>A description of all resources used, including antibodies, cell lines, animals and software tools, with enough information to allow them to be uniquely identified, should be included in the Methods section. Authors are strongly encouraged to cite Research Resource Identifiers (RRIDs) for antibodies, model organisms and tools, where possible.<br><br>Have you included the information requested as detailed in our Minimum Standards Reporting Checklist?	Yes
**Availability of data and materials**<br><br>All datasets and code on which the conclusions of the paper rely must be either included in your submission or deposited in publicly available repositories (where available and ethically appropriate), referencing such data using a unique identifier in the references and in the “Availability of Data and Materials” section of your manuscript.<br><br>Have you have met the above requirement as detailed in our Minimum Standards Reporting Checklist?	Yes

Powered by Editorial Manager® and ProduXion Manager® from Aries Systems Corporation

Manuscript

Click here to access/download;Manuscript;Manuscript_GIGA-D-23-00044_Revision_2_highlighted_version.pdf

OXFORD (GIGA)^n SCIENCE

GigaScience, 2023, 1–15

doi: xx.xxxx/xxxx
Research

RESEARCH

Evaluation of Swin Transformer and knowledge transfer for denoising of super-resolution structured illumination microscopy data

Zafran Hussain Shah[1,*], Marcel Müller[2], Wolfgang Hübner[2], Tung-Cheng Wang[2,3], Daniel Telman[1], Thomas Huser[2] and Wolfram Schenck[1,*]

[1]Faculty of Engineering and Mathematics, Bielefeld University of Applied Sciences and Arts, Interaktion 1, 33619 Bielefeld, Germany and [2]Faculty of Physics, Bielefeld University, Universitätsstr. 25, 33615 Bielefeld, Germany and [3]Leica Microsystems CMS GmbH, Am Friedensplatz 3, 68165 Mannheim, Germany

[*]zafran_hussain.shah@hsbi.de; wolfram.schenck@hsbi.de

Abstract

In recent years, convolutional neural network (CNN)-based methods have shown remarkable performance in the denoising and reconstruction of super-resolved structured illumination microscopy (SR-SIM) data. Therefore, CNN-based architectures have been the main focus of existing studies. Recently, however, an alternative and highly competitive deep learning architecture, Swin Transformer, has been proposed for image restoration tasks. In this work, we present SwinT-fairSIM, a novel method for restoring SR-SIM images with low signal-to-noise ratio (SNR) based on Swin Transformer. The experimental results show that SwinT-fairSIM outperforms previous CNN-based denoising methods. Furthermore, the generalization capabilities of deep learning methods for image restoration tasks on real fluorescence microscopy data have not been fully explored yet, i.e., the extent to which trained artificial neural networks are limited to specific types of cell structures and noise. Therefore, as a second contribution, we benchmark two types of transfer learning, i.e., direct transfer and fine-tuning, in combination with SwinT-fairSIM and two CNN-based methods for denoising SR-SIM data. Direct transfer does not prove to be a viable strategy, but fine-tuning achieves results comparable to conventional training from scratch, while saving computational time and potentially reducing the amount of required training data. As a third contribution, we publish four datasets of raw SIM images and already reconstructed SR-SIM images. These datasets cover two different types of cell structures, tubulin filaments and vesicle structures. Different noise levels are available for the tubulin filaments. These datasets are structured in such a way that they can be easily used by the research community for research on denoising, super-resolution, and transfer learning strategies.

Key words: Structured illumination microscopy; Fluorescence microscopy; Deep learning; Tranformers; Swin Transformer; SwinIR; Convolutional neural networks; Denoising; Image restoration; Transfer learning; Fine-tuning

Introduction

In optical microscopy, super-resolution structured illumination microscopy (SR-SIM) plays a significant role in the field of biological and biomedical studies to analyze living cells and biological specimens with characteristic features below the resolution limit of classical microscopes (approx. 250 nm for high-end systems with oil-immersion objective lenses). Structures of interest include e.g. the internal organelles of mitochondria, cellular cytoskeleton, virus particles, or small vesicles [1, 2, 3, 4, 5]. SR-SIM is an important super-resolution approach to disentangle complex biological cellular structures with an up to twofold enhancement of spatial resolution beyond the

Compiled on: September 22, 2023.
Draft manuscript prepared by the author.

diffraction limit. During the process of super-resolution imaging, SR-SIM involves the illumination of the biological sample with spatially patterned light following a sinusoidal intensity distribution. A series of raw images with typically 3 or 5 different phase positions and 3 angles of orientation of the illumination pattern are typically acquired [6]. Subsequently, frequency-domain based image reconstruction algorithms, e.g. implemented in software packages such as fairSIM [7], OpenSIM [8], and Python-based packages [9] are applied to a set of raw SIM images to generate the final twofold super-resolved images. SR-SIM has many advantages over other super-resolution methods e.g., it does not require special sample preparation, it permits the use of conventional fluorophores in multiple colors, simultaneously, and it allows for imaging at high speed and large fields-of-view (FOV) while being compatible with live cell samples by making efficient use of low illumination intensity levels [10, 11]. The conventional SR-SIM reconstruction algorithms have some limitations whenever the signal-to-noise level of the raw images is poor due to low fluorescence emission or short exposure times [12]. In general, in fluorescence microscopy weak emission due to, e.g. low labeling densities, high light scattering or absorption, or optical aberrations is often encountered [13, 14]. In the case of several super-resolution fluorescence microscopy methodologies, this leads to low signal-to-noise ratio (SNR), and poor image reconstructions or artifacts. Nevertheless, SR-SIM imaging provides better resolution and optical sectioning abilities than confocal microscopy with a just minimally larger number of raw images to be acquired [15].

In the last decade, deep learning methods have become generally accepted in image processing. In addition, they are being used with increasing success for the restoration of SR-SIM images, e.g. for denoising [16, 17, 18, 19, 20]. Qiao et al. proposed a generative adversarial network (GAN) based deep Fourier channel attention network (DFCAN) method to reconstruct SR-SIM images under low SNR conditions [21]. Xypakis et al. introduced a custom convolutional neural network (CNN) architecture for blind-SIM: BS-CNN [22]. Liu et al. proposed a dual-domain learning strategy for the reconstruction of SIM images [23]. Authors in [24] suggested another GAN based channel attention generative adversarial network (caGAN) to improve the quality of 3D-SIM reconstruction using fewer raw samples of low SNR.

Recently, so-called Transformers have shown great success in various natural language processing tasks [25, 26, 27, 28, 29]. Since then, as an alternative to CNNs, Transformer-based architectures have been adapted to computer vision tasks as well, such as classification [30], detection [31, 32], and image restoration [33]. Vision Transformers for the image restoration typically divide each image into fixed-size patches and process each patch independently to limit computational complexity [34], which results in the introduction of border artifacts around each patch in the restored image. The Swin Transformer overcomes this shortcoming by integrating a shifted window operation along with a few CNN layers in the Transformer architecture [35]. Swin serves as the basis for the Shifted window Image Restoration (SwinIR) method [36], which has been proposed especially for various image restoration tasks. Although these latest Transformers outperform CNN-based methods in conventional image restoration to some extent, they were never explored for the restoration of high-resolution SR-SIM microscopy images. Therefore, during this work, we propose a SwinIR based Transformer architecture named "SwinT-fairSIM" to denoise SR-SIM images under low SNR conditions.

Both CNN- and Transformer-based methods require a large number of images to train the underlying models. In the field of microscopy, the sheer size and storage requirements of datasets of high-resolution reconstructed microscopic images make them difficult to produce and typically not publicly available. The few open source datasets available are mostly related to wide-field microscopy and contain a relatively small number of images. For example, Zhang et al. [37] collected three wide-field microscopy datasets purely for denoising tasks without providing high-resolution ground truth images obtained by SR-SIM technology. They used image averaging to generate ground truth data with high SNR images. Zhou et al. [38], on the other hand, published a dataset called "Widefield2SIM" using wide-field fluorescence microscopy. They captured 120 different fields of view (FOV) with 400 low SNR images for each FOV and generated high-resolution ground truth data using SR-SIM imaging technology. Qiao et al. [21] presented the "BioSR" dataset consisting of 2200 pairs of low-resolution (LR) raw data and high-resolution (HR) data. Four different biological structures (CCPs, ER, MTs, F-actin), nine signal levels (15-600 average photon count), and two upscaling-factors (linear SIM and non-linear SIM) are covered in the BioSR dataset according to Qiao et al. Similarly, Hagen et al. [39] published a variety of datasets which were collected using wide-field and confocal microscopy. They captured various fluorescently labeled structures such as actin, mitochondria, membrane, and nuclei with low and high SNR. However, their collection of datasets consists of significantly fewer images than the BioSR data collection.

Here, we present a series of datasets that are related to SR-SIM microscopy for image restoration tasks. These datasets cover two types of biological structures, tubulin filaments, and vesicles, with several fields-of-view for the denoising, super-resolution, and joint denoising and super-resolution tasks. In our datasets, noisy input and reference output images were obtained by using SR-SIM reconstruction algorithms. We believe that our datasets will be helpful for the research community to benchmark different deep-learning based denoising and super-resolution (SR) methods, not least because of the rather large number of provided samples.

Finally, we show how some of these datasets can be used to demonstrate the generalization capabilities of image restoration algorithms in the field of SIM microscopy. To this end, we apply the concept of transfer learning to three algorithms for the denoising of SR-SIM data. Two of these algorithms are based on CNNs and were proposed by us in [16]. They are called "Red-fairSIM" and "UNet-fairSIM". The third algorithm is the above-mentioned Transformer architecture "SwinT-fairSIM", which is first proposed in this paper. With respect to transfer learning, we compare two methods from this area: Direct transfer and fine-tuning. In direct transfer, a model pre-trained on one type of data is used for inference on a related but different type of data. In fine-tuning, part of the pre-trained model is retrained on the new type of data before inference. In previous work, we were already able to show that some CNN-based algorithms for the denoising of SR-SIM images are robust to different noise levels and SIM modes, e.g. varying pattern spacings at different illumination wavelengths [19]. However, due to the limited amount of SR-SIM data available at that time, we were not able to explore the techniques of transfer learning in more depth. Thus, here, we also aim to answer the following questions related to direct transfer and fine-tuning: (1) If a model is trained on a specific biological structure with a specific type of noise, will it also generalize well to denoising another, different structure with another type of noise? And: (2) Is fine-tuning of a pre-trained model (i.e., previously trained on one type of structure and noise) more effective than training the model from scratch? To answer these questions, we have conducted a series of experiments which are discussed in later sections.

In particular, the contribution of this work is threefold:

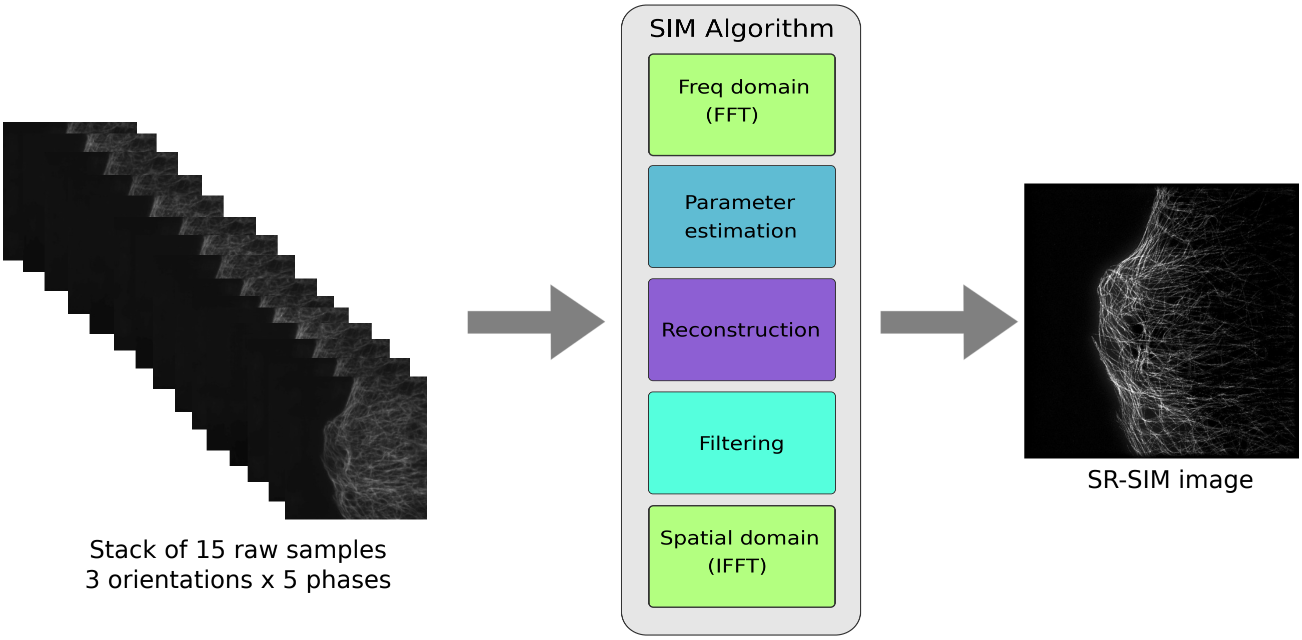

Figure 1. The architecture of the image reconstruction algorithms which are used to generate the reconstructed SR-SIM images in this work. Both reconstruction algorithms (i.e., as implemented in the fairSIM and softWoRx software) are based on the SIM algorithm. During the reconstruction of SR-SIM images, a stack of 15 raw SIM images of size 512 × 512 pixels is processed by the respective software which generates the high-resolution SR-SIM image of size 1024 × 1024 pixels as output.

First, we present high-resolution SR-SIM datasets for image denoising and super-resolution tasks. Second, we propose a method based on the SwinIR architecture for denoising SR-SIM images. Third, we evaluate the potential of direct transfer and fine-tuning for different Transformer- and CNN-based models.

Materials and Methods

SR-SIM microscopy and sample preparation

The raw SIM images for all the datasets were acquired using a DeltaVision OMX V4 (GE Healthcare, Chicago, IL, USA) 3D-SIM imaging system. The DeltaVision OMX V4 imaging system is an implementation of 3-beam 3D-SIM [15], providing both lateral and axial modulation of the excitation pattern, and thus allows to increase both the lateral and axial resolution of the imaging process (as compared to 2-beam SIM, where only the lateral resolution is increased). The system is equipped with four excitation laser lines (405nm, 488nm, 561nm and 642nm), and four sCMOS cameras to detect the emitted fluorescence light in four channels. The camera pixel size of 6.5 µm and an overall magnification of 82x yields a fixed effective pixel size of 80 nm. The magnification cannot be changed by the user, and has been set by the manufacturer to fulfill the Nyquist criterion, thus not loosing spatial information in the sampling process. While fulfilling the Nyquist sampling criterion only sets a maximum pixel size, pixel sizes much smaller than the limit are not used, as this would compromise both SNR (each pixel contributing a constant amount of read noise) and field of view (as the overall feasible pixel count is finite). To prepare the raw SIM image data (datasets 1-3) of the tubulin cytoskeleton, U2OS cells were cultured in DMEM supplemented with 10%FBS and grown on round coverslips of 170±5 µm thickness (No. 1.5H). Cells were fixed with 4% PFA for 15 min., followed by PBS washes, and permeabilization with 0.5% Triton-X100 for 3 min. Another two rounds of PBS washes were done before blocking with 3% BSA. For immunolabeling of the tubulin microfilaments, cells were stained with anti-tubulin antibody (Invitrogen Cat. No. 322500) 1:400 for 2 hr at room temperature, followed by a PBS wash and one additional hour of incubation with Alexa 488-conjugated anti-mouse IgG 1:400. Afterwards the cells were then briefly washed with PBS before Vectashield was applied to embed the coverslip onto a standard microscopy glass slide for imaging. For the preparation of raw SIM images with vesicle structures (dataset 4), U2OS cells were transfected with Lipofectamine 3000 according to the manufacturers protocol (Thermofisher Cat. No. L3000-001) together with a plasmid expressing the vesicular Lamp1 protein fused to the fluorescent protein mScarlet. After 24 hr transfection the cells were fixed with 4% PFA for 10 min., followed by PBS washes and Vectashield mounting prior to imaging. The vesicular structures represent lysosomes. During the data collection, the illumination intensity as well as the camera exposure time remained fixed. As the cameras as also actively cooled, thermal- and read-noise components should not vary during time-lapse image acquisition. Also, as typical for modern sCMOS cameras, thermal- and read-noise components are small (in the order of single electrons per read-out) compared to the Poisson noise, i.e. the stochastic distribution of detected photons generated by the quantum nature of light propagation. The dramatic drop in signal-to-noise ratio during data acquisition is due to photobleaching and phototoxicity in the sample.

Dataset preprocessing and image reconstruction

Dataset 1 is a raw dataset without any image processing or reconstruction applied to the raw SIM images. The images in the datasets 2 and 3 are reconstructed by using the open-source fairSIM reconstruction algorithm as shown in Figure 1. fairSIM implements a single-slice (2D) SR-SIM image reconstruction algorithm [7]. It works in three steps: parameter estimation, reconstruction, and filtering. The mathematical and algorithmic details of the fairSIM reconstruction method are explained in the original publication [7]. A synthetic optical transfer function, with NA = 1.4, λ = 525 nm, a = 0.31

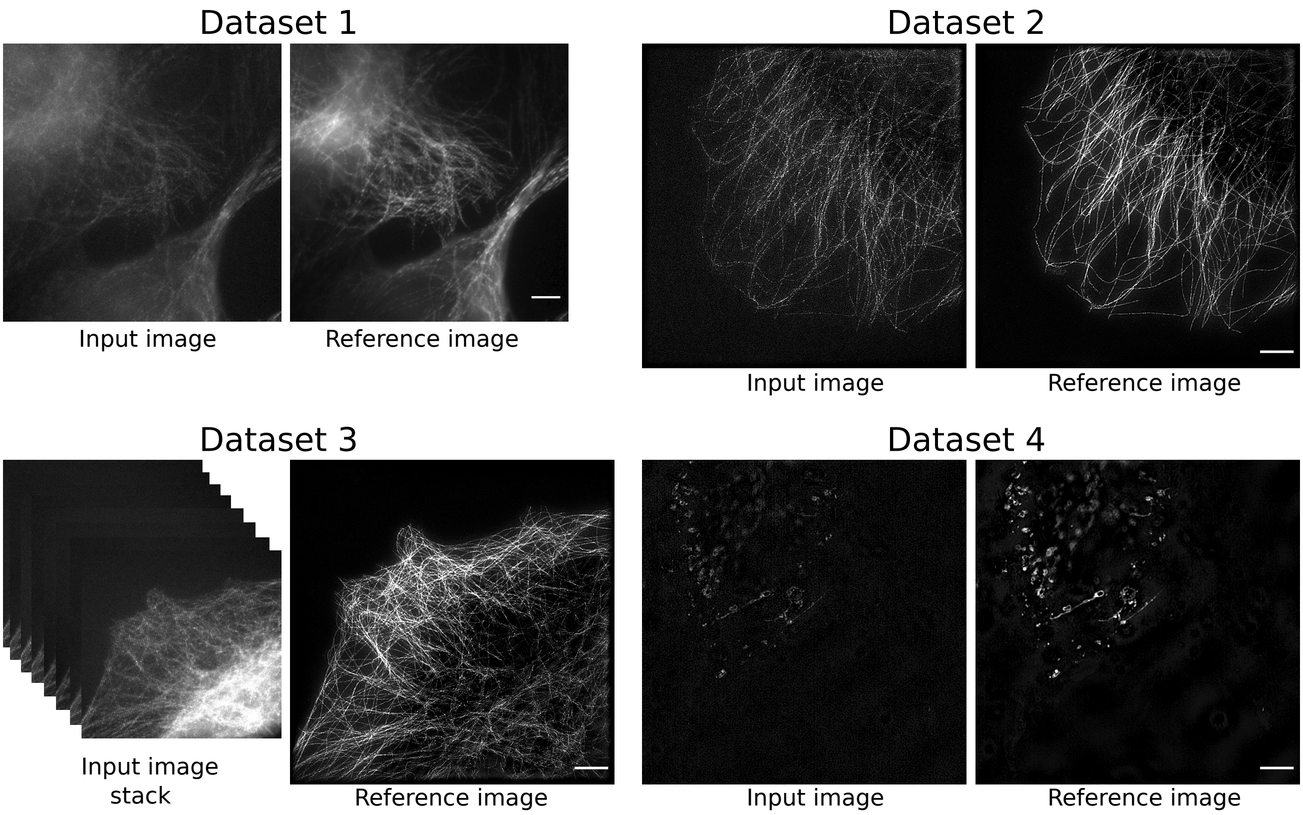

Figure 2. Dataset 1 consists of raw SIM data. The input-reference pairs represent each phase and orientation from different noise levels of tubulin filaments. The size of the input and reference images is 512 × 512 pixels in dataset 1. Scale bar: 4 µm. Dataset 2 contains the reconstructed SR-SIM images of tubulin structure, and each input-reference pair has a size of 1024 × 1024 pixels. Scale bar: 4 µm. In dataset 3, raw SIM data in the form of an image stack (i.e., 15 × 512 × 512 pixels) are used as input samples, whereas the reconstructed SR-SIM images (1024 × 1024 pixels) are used as the corresponding reference samples for joint denoising and super-resolution. Scale bar of reference image: 4 µm. Dataset 4 is based on the reconstructed SR-SIM images of size 1024 × 1024 pixels of fluorescently labeled vesicles. Scale bar: 4 µm.

(a is a compensation parameter,see [7, 40]) is used. For the tubulin samples, a background of 500 counts per pixel is subtracted during the reconstruction process. SR-SIM reconstruction parameters (pattern orientation, global phase, etc.) are automatically determined by fairSIM's standard, iterative cross-correlation approach. Filter parameters are set to a generalized Wiener filter with a strength of $w = 0.05$, apodization is set at 1.9× the resolution limit with a *bend* of 0.8. A notch-style filter implemented as *OTF attenuation* with a strength of 0.995 and a FWHM of 1.2 µm^{-1} is used. The full information about the functionality of these parameters is explained in [7], and the general guide for using SIM reconstruction parameters is discussed in [40]. The code used to generate the samples of dataset 2 is available at [41]. The samples in dataset 4 (the vLamp1-mScarlet expressing cell) were reconstructed using the commerical software 'softWoRx v7' (GE Healthcare manufacturer's software) for 3D-SIM. Here, a full 3D volume of data is acquired by the microscope and passed through the reconstruction process as a 3D stack. While both single-slice reconstruction (fairSIM) and full 3D volume reconstruction offer the same increase in lateral resolution, a full 3D volume reconstruction additionally provides an axial resolution increase (when used on 3-beam data as provided by the Delta Vision OMX and other 3-beam SIM systems). This of course comes at the expense of requiring full 3D volumes of data to be aquired, largely increasing imaging time and photo-toxicity. Thus, depending on the imaging needs, either single-slice data acquisition (and reconstruction) or full 3D volumes are chosen. For an overview of all datasets, see Table 1.

Description of datasets

Dataset 1

Dataset 1 contains around 101 fields-of-view (FOV) of tubulin filaments and each FOV further consists of 3000 images (all in one TIF file for each FOV). In each FOV, a stack of 15 raw SIM images represents the combination of 5 orientations and 3 phases, whereas this full stack is repeatedly captured for 200 timestamps. The signal-to-noise ratio decreases with every timestamp. Thus, dataset 1 contains a total of 303000 raw SIM images of size 512 × 512 (width ×height) pixels with 15 combinations of phase and orientation at each timestamp. Each timestamp in the raw images lasts approximately 25 ms. Each pixel contains a single 16-bit integer value captured by the microscope's camera, which is calibrated to provide a signal linear in photon count for each pixel. This is typical for scientific camera systems, but dissimilar to standard image processing, where often gamma mapping is applied between light intensity and pixel values. This dataset can be used mainly for image denoising tasks (from noisy input to output with higher signal-to-noise ratio). Therefore, the images from timestamp 1 are intended as output images (reference/ground truth), and the rest of the images can be categorized as input images as shown in Figure 2.

Dataset 2

We constructed dataset 2 by applying the fairSIM reconstruction algorithm to the raw SIM images from dataset 1. Dataset 2 contains therefore pairs of reconstructed high-resolution SR-SIM images, each pair consisting of the noisy input and the reference output. The process of the generation of input and

Table 1. Description of all the datasets

Dataset	Dataset 1	Dataset 2	Dataset 3	Dataset 4
Structure	tubulin filaments	tubulin filaments	tubulin filaments	vesicles
Microscope	SR-SIM microscopy	SR-SIM microscopy	SR-SIM microscopy	SR-SIM microscopy
Pixel Size, nm	80	40	80 (input) / 40 (ref.)	40
Number of timestamps	200	200	200	max: 99, min: 15
Fields-of-view	101	101	101	175
Input image size, pixels	512x512	1024x1024	15x512x512	1024x1024
Output/Reference image size, pixels	512x512	1024x1024	1024x1024	1024x1024
No of samples	303000	2525	2525	7284
Reconstruction	raw data	fairSIM	fairSIM	softWoRx

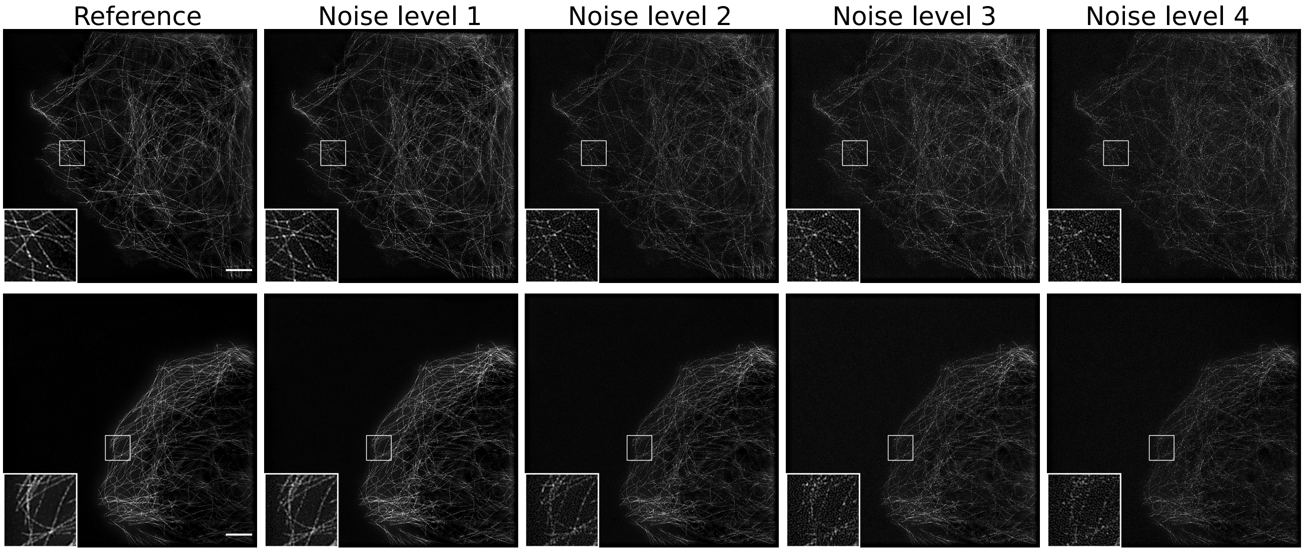

Figure 3. SR-SIM images from two different FOV, each shown at several noise levels. Each noise level corresponds to a different range of timestamps, e.g., images of noise level 4 are taken at the last timestamps and contain a high level of noise. Similarly, images of noise level 1 represent early timestamps with very low noise. The reference image is recorded at timestamp 0 and has the highest signal-to-noise ratio. Scale bar: 4 µm.

output SIM images from the raw SIM images is shown in Figure 1. The 15 raw SIM images of size 512 × 512 (width × height) pixels of different phases and orientations are propagated into the fairSIM algorithm to reconstruct the SR-SIM images of size 1024 × 1024 (width × height) pixels. During the formation of this dataset, the raw samples from timestamp 1 were used to generate the output images (i.e., reference images). We use the term 'reference images' instead of 'ground truth images' because of the SIM reconstruction artifacts in the output images of this dataset. The input samples were reconstructed by using the raw SIM images from timestamps 176 – 200. Therefore, dataset 2 is composed of 2525 reconstructed pairs of SR-SIM images with a size of 1024 × 1024 (width × height) pixels extracted from the 101 FOV. In addition to these 2525 image pairs, for the last 20 FOV we also include image pairs in the data collection where the noisy input is from timestamps 76 – 100, 126 – 150 and 176 – 200. This additional data can be used to create test sets to evaluate the robustness of denoising networks for different noise levels. In our previous work [19], we denoted data from timestamp 26 – 50 as noise level 1. Similarly, noise level 2, 3, and 4 correspond to the data from the timestamps 76 – 100, 126 – 150 and 176 – 200 (shown in Figure 3). Overall, dataset 2 is generated mainly for the denoising of SR-SIM images.

Dataset 3

Dataset 3 was created mainly for joint denoising and super-resolution tasks. The composition of this dataset is based on the stack of raw noisy input SIM images obtained from Dataset 1 of noise level 4 and the reference SR-SIM images from Dataset 2 of timestamp 0. The raw noisy SIM images in the input stack represents different illumination phases and orientations. The reference samples are the reconstructed high-resolution SR-SIM images. The size and dimension of each input sample are 15 × 512 × 512 (depth × width × height) pixels. The size of the output or reference sample is 1024 × 1024 (width × height) pixels. The input and reference samples in this dataset contain tubulin filaments as biological structures.

Dataset 4

Dataset 4 is composed of 3D-SR-SIM images showing vesicles in U2OS cells (i.e. typically round intracellular droplets) and can be used mainly for denoising tasks. Full 3D SIM raw data stacks have been passed through a 3D-enabled SIM reconstruction, extending both lateral and axial resolution of the data [15]. This data is composed of nine z-stacks, each with a varying number of depth levels (z-planes), and each captured for a different number of timestamps. In our work, we focus on the denoising along the lateral plane, therefore each z-stack is split into its single z-planes (also called "slices"). The recorded structures in the different z-planes are very different from each other, so we categorize them here as separate FOV.[1]

As result, the raw data of this dataset contains 175 FOV, and each FOV is recorded for a different number of timestamps. This yields a total of 7284 pairs of input and output

1 The file names in the dataset contain also indices for the original z-stack.

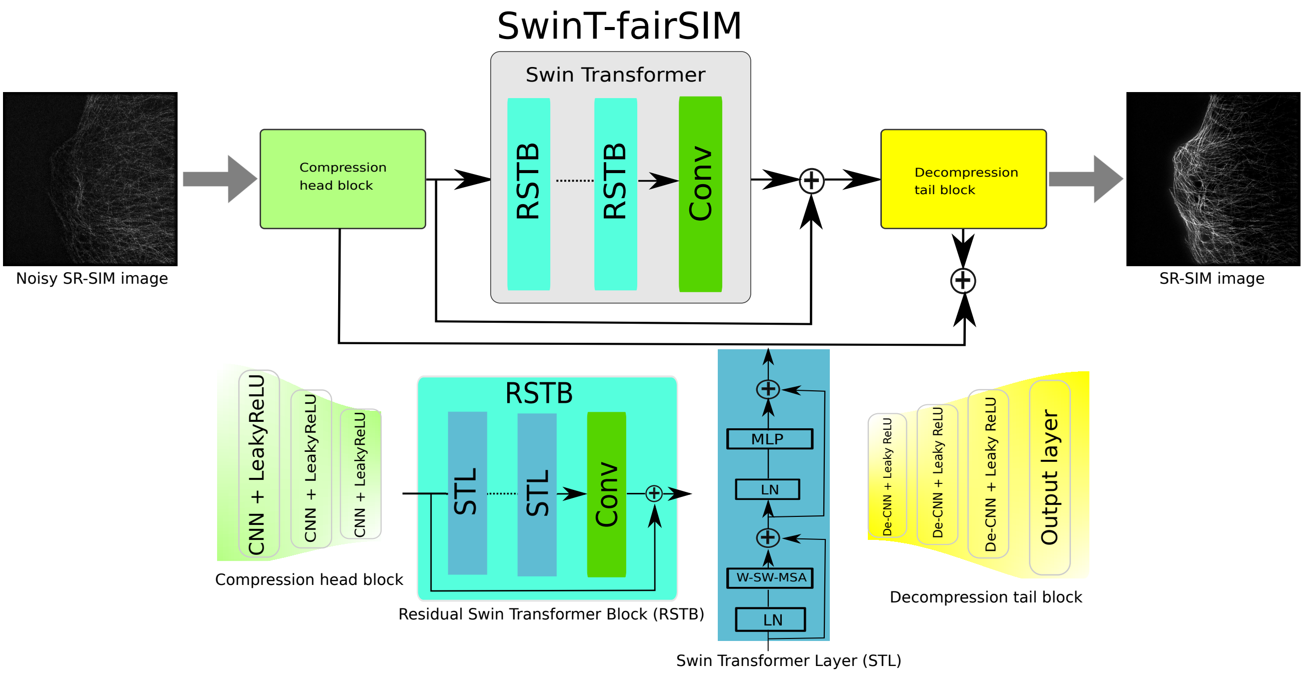

Figure 4. The architecture of the SwinT-fairSIM denoising method. A more detailed view into each block of the pipeline is shown in the lower part of the figure.

SR-SIM images. The size of the input and reference images is 1024×1024 (width × height) pixels. In each image pair, the output/reference image is taken from timestamp 1 of the respective FOV, whereas the input images are taken from the following time stamps and exhibit therefore a lower signal-to-noise ratio. The rational for splitting 3D data into separate 2D slices for denoising and artifact removal is as follows: The structure of the 3D SIM reconstruction algorithm (shifting and recombining lateral but not axial frequencies) makes it very likely that noise and reconstruction artifacts arise in the lateral directions of the acquired images, not so much in the axial component. Limiting the post-processing to 2D increases speed and reduces training effort significantly. Also, the height of a 3D stack (number of slices) varies from sample to sample, while the lateral FOV is typically constant. Of course, dataset 4 would allow full 3D implementations to be tested, by recombining the z-sliced data into 3D volumes. The reconstruction of the original raw SIM images into the SR-SIM images in this dataset was performed by using the softWoRx v7 software (GE Healthcare manufacturer's software).

Data partitioning

In our experiments, in the first three datasets, the images of the last 20 FOV were used as test samples, and the remaining 81 FOV were used for the training set. The training set of dataset 2 is therefore composed of 2025 samples (image pairs) from 81 FOV and the test set is composed of 500 samples from 20 FOV. Similarly, in dataset 4 we used 5562 samples from 121 FOV for the training set and 1380 samples from 46 FOV for the test set; the remaining 8 FOV were discarded because their reference images contain only noise without any meaningful structure. Datasets 1, 2, and 4 can only be used for denoising tasks, however, the data from dataset 3 can be used for both joint denoising and super-resolution. To reproduce the results of this work, we share the source code on the GitHub repository [42], the trained models [43] and the data on GigaDB ["link will be uploaded after the allocation of space from Giga Database"].

Shifted window Transformer for the denoising of SR-SIM images (SwinT-fairSIM)

The architecture of SwinT-fairSIM is composed of three main components: Compression head, Swin Transformer, and decompression tail block as shown in Figure 4. The architecture of SwinT-fairSIM is an extended version of the SwinIR architecture [36]. The compression head block is responsible for shallow feature extraction and the downsizing of the input images, the Swin Transformer block is based on encoder-decoder components to recover missing information and for deep feature extraction, and the decompression tail component is used for upsampling and restoring the features in the resultant images. The compression head block is composed of three convolution layers to compress the input image features to a size of 256×256 (width × height) pixels by using a stride of size 2. The encoder-decoder Swin Transformer block is made up of several Residual Swin Transformer blocks (RSTB), and each RSTB is based on few Swin Transformer layers (STL) and convolutional layers [36]. The STL is further composed of two window (W) and shifted window (SW) based multi-head self-attention (MSA) modules, followed by a multi-layer perceptron that has two fully-connected layers with GELU non-linearity [25, 35]. The shifted window partitioning strategy introduces connections between non-overlapping patches in the preceding layer and is found to be effective in a variety of computer vision tasks [36]. The Swin Transformer layer first reshapes the input (X) of size $H \times W \times C$ (height × width × channels) into $\frac{HW}{M^2} \times M^2 \times C$, where the $\frac{HW}{M^2}$ is the total number of windows or patches. Two additive based residual skip connections are also used in each STL unit. The query (Q), key (K), and value (V) matrices are then calculated for each window separately to obtain self-attention.

$$Q = XP_Q,\ K = XP_K,\ V = XP_V \qquad (1)$$

In equation (1), P_Q, P_K, and P_V are the shared projection matrices across all the windows. The attention matrix is then computed by the self-attention mechanism in a local window as

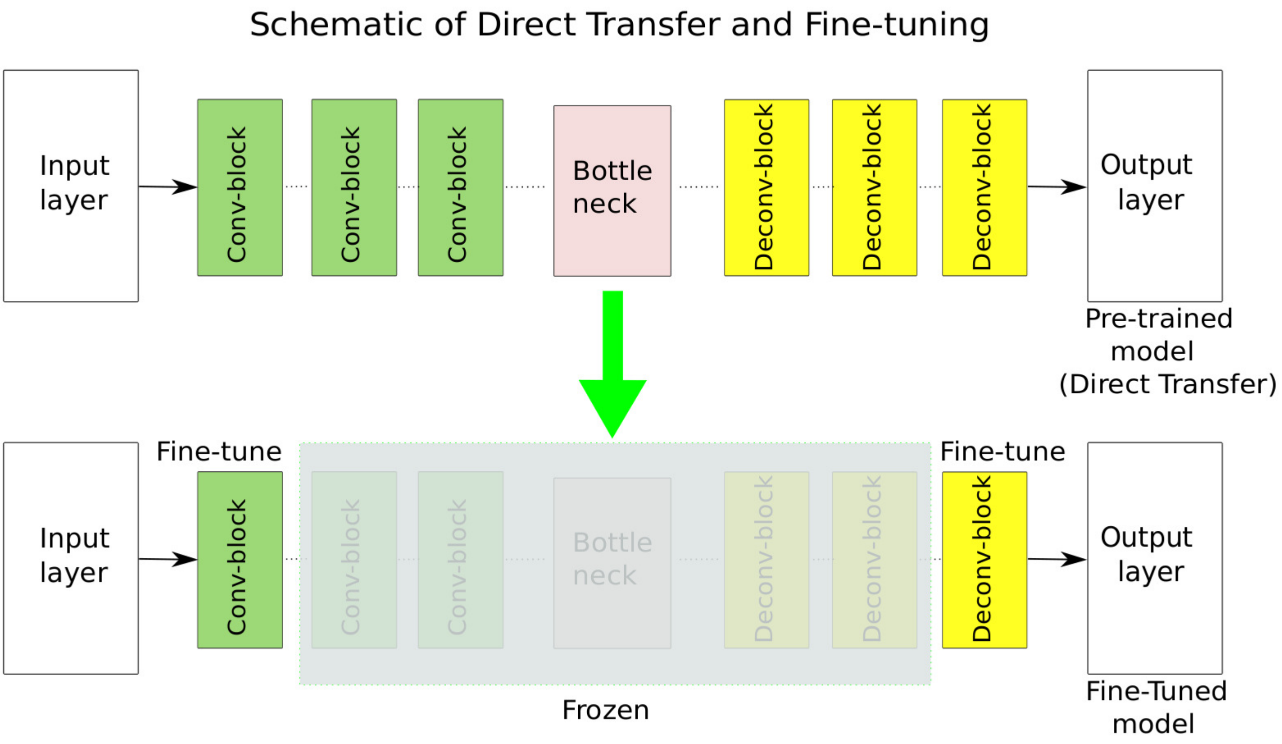

Figure 5. Schematic diagram of direct transfer and fine-tuning. The first pipeline shows the pre-trained model. The highlighted region in the second pipeline shows the frozen part; all parts of the model that are not frozen are retrained in the fine-tuning strategy.

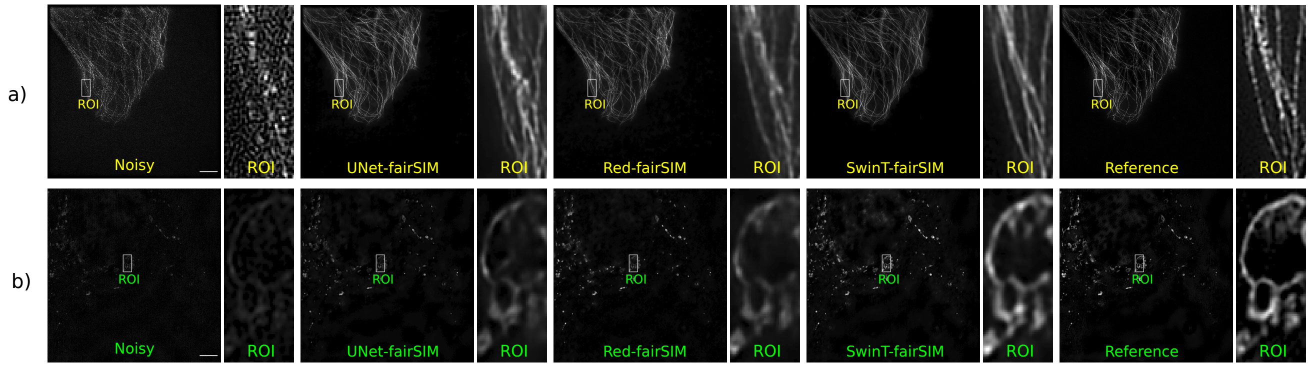

Figure 6. Results for test samples from datasets 2 and 4 are shown in this figure in the first row **(a)** and second row **(b)**. The outputs of UNet-fairSIM, Red-fairSIM, and SwinT-fairSIM are shown next to each other. The second, fourth, sixth, eighth, and tenth column depict the cropped and zoomed regions of interests (ROIs) from the full-size SR-SIM images (1024 × 1024 pixels). The cropped ROIs of size 50 × 100 pixels were upsampled to 150 × 300 pixels for demonstration purposes. Scale bar: 4 µm.

follows:

$$\text{Attention}(Q, K, V) = \text{SoftMax}(QK^T/\sqrt{d} + B)V \quad (2)$$

where B in equation (2) is the learnable relative positional encoding and d is the dimension of query and key features. The consecutive Swin Transformer layers are defined as:

$$\hat{X}^l = \text{W-MSA}(\text{LN}(X^{l-1})) + X^{l-1} \quad (3)$$
$$X^l = \text{MLP}(\text{LN}(\hat{X}^l)) + \hat{X}^l \quad (4)$$
$$\hat{X}^{l+1} = \text{SW-MSA}(\text{LN}(X^l)) + X^l \quad (5)$$
$$X^{l+1} = \text{MLP}(\text{LN}(\hat{X}^{l+1})) + \hat{X}^{l+1} \quad (6)$$

In equations (3)-(6), $\hat{X}^l$ and X^l denote the output features of the (S)W-MSA and MLP modules for layer l, where LN represents the LayerNorm operation.

Finally, the decompression tail block accommodates 3 transposed convolutional layers along with the output convolutional layer to transform and upsample the feature maps into the final resultant images. The necessity of head and tail block is to scale down and upsample the SR-SIM images with a size of 1024 × 1024 (height × width) pixels. In the SwinT-fairSIM method, we set the RSBT, STL, window size, and the attention head numbers to 5, 5, 8, and 120 respectively. The adjacent layers in the head and tail blocks are connected via additive skip connection and contain the same number of kernels. These blocks differentiate our architecture from the existing SwinIR architecture [36]. These blocks reduce the computational effort in the central Transformer part of the architecture and therefore adapt the Swin Transformer block to images with a high pixel count.

The name "SwinT-fairSIM" was chosen to be consistent with the names of our previously proposed CNN-based denoising methods for SR-SIM data, "Red-fairSIM" and "UNet-fairSIM" [16]. Red-fairSIM and UNet-fairSIM are described in detail in [16]. These algorithms originally worked only in conjunction with the fairSIM software [7] for SR-SIM reconstruction. In contrast, in this paper, we also use the softWoRx implementation of the SIM algorithm for dataset 4. However, to be consistent with the naming scheme in [16], we keep the suffix "fairSIM" in all of our deep learning based approaches.

Transfer learning and fine-tuning

In transfer learning in general, the knowledge of a trained model from a related task that has been learned is transferred to another task from the same domain [44]. The parameters of the pre-trained model, trained on specific data and for a specific task, are transferred to the different data but related task [45, 46]. The use of transfer learning in deep learning is very useful to reduce the computational demands and time complexity [47]. In addition, transfer learning is also very helpful when it comes to alleviating large dataset requirements [48].

The simplest form of transfer learning is to apply a trained model directly to another task without retraining. This direct use of a pre-trained model on new test data is called "direct transfer". Beyond this most basic approach, fine-tuning is one of the most important strategies for transferring model knowledge from one domain to another [49]. During fine-tuning, the weights of some layers of pre-trained models are preserved and the rest of the layers are retrained (i.e., "fine-tuned") [50]. The concepts of direct transfer and fine-tuning are illustrated in Figure 5. It can be seen from the schematic in Figure 5 that the weights of the intermediate layers of the pre-trained models are frozen while the rest of the layers are retrained in the fine-tuning strategy, whereas no retraining is performed in the direct transfer approach. For our work here, in the direct transfer strategy, the models are trained from scratch on dataset 2, and then the trained model is evaluated with test samples from dataset 4. Similarly, the models trained on dataset 4 are evaluated on the test images from dataset 2. In the fine-tuning strategy, models are trained from scratch on dataset 2, afterwards they are partly retrained on dataset 4 and also tested on dataset 4. Or the other way round: Complete initial training on dataset 4, afterwards fine-tuning on dataset 2 and finally evaluation on dataset 2. In this work, we performed these different training strategies with the SwinT-fairSIM, Red-fairSIM, and UNet-fairSIM algorithms [19] with datasets 2 and 4.

To perform fine-tuning on the SwinT-fairSIM algorithm, we retrained the head and tail blocks along with one adjacent RSTB block each. By doing so, about 1.9 million training parameters stayed frozen out of 4 million parameters. Regarding the CNN-based algorithms from our previous work [19], we used the following approaches to fine-tuning: Red-fairSIM is based on the residual-encoder-decoder network (RED-Net) [51]. In the fine-tuning approach for Red-fairSIM, we retrained the first and last 5 layers of the model instead of all 30 layers. By doing so, about 700K training parameters stayed frozen out of more than 1 million parameters. UNet-fairSIM is based on the UNet architecture which is based on several encoder and decoder blocks [52]. In the fine-tuning of UNet-fairSIM, we simply retrained the first and last two encoder and decoder blocks; in doing so, more than 29 million parameters out of 33 million parameters stayed frozen.

Generally, in the first step, all these algorithms were trained from scratch on datasets 2 and 4 separately for 100 epochs. The mean squared error (MSE) was used as a loss function for all training runs with the Adam optimizer. The learning rate was set to 1×10^{-4} for all training runs. In the next step, we applied direct transfer by propagating the test samples from the respective dataset not used for training the pre-trained models. In direct transfer, the test images of the dataset 2 (i.e., tubulin filaments) were propagated through the pre-trained model trained on the dataset 4 (i.e., vesicle structure) and vice versa. In the third step, the pre-trained models were fine-tuned using training data from the respective dataset not used for initial training. 30 training epochs were used for fine-tuning. The fine-tuned models were then evaluated on the test samples from the dataset used in the fine-tuning process. When fine-tuning the pre-trained models, we initially kept different numbers of layers/blocks frozen. However, we found that the best results were obtained by fine-tuning rather few trainable parameters for 30 epochs (except for SwinT-fairSIM, where a larger part of the network had to be "unfrozen"). In the final step, we compared the results of conventional training with the direct transfer and fine-tuning strategies visually, as well as in terms of peak signal-to-noise ratio (PSNR) [53] and structural similarity index measurement (SSIM) [54] values.

Results

We collected four data sets for denoising and super-resolution tasks. The overview and full characteristics of these datasets are shown in Table 1. The images in dataset 2 contain the tubulin structure along with mixed Poisson-Gaussian (MPG) noise and SR-SIM reconstruction artifacts. The images in dataset 4 contain the vesicle structure along with MPG noise and honeycomb pattern artifacts that arise when raw data carrying predominantly Poisson noise is subjected to the frequency-based SR-SIM reconstruction algorithm, which then introduces re-

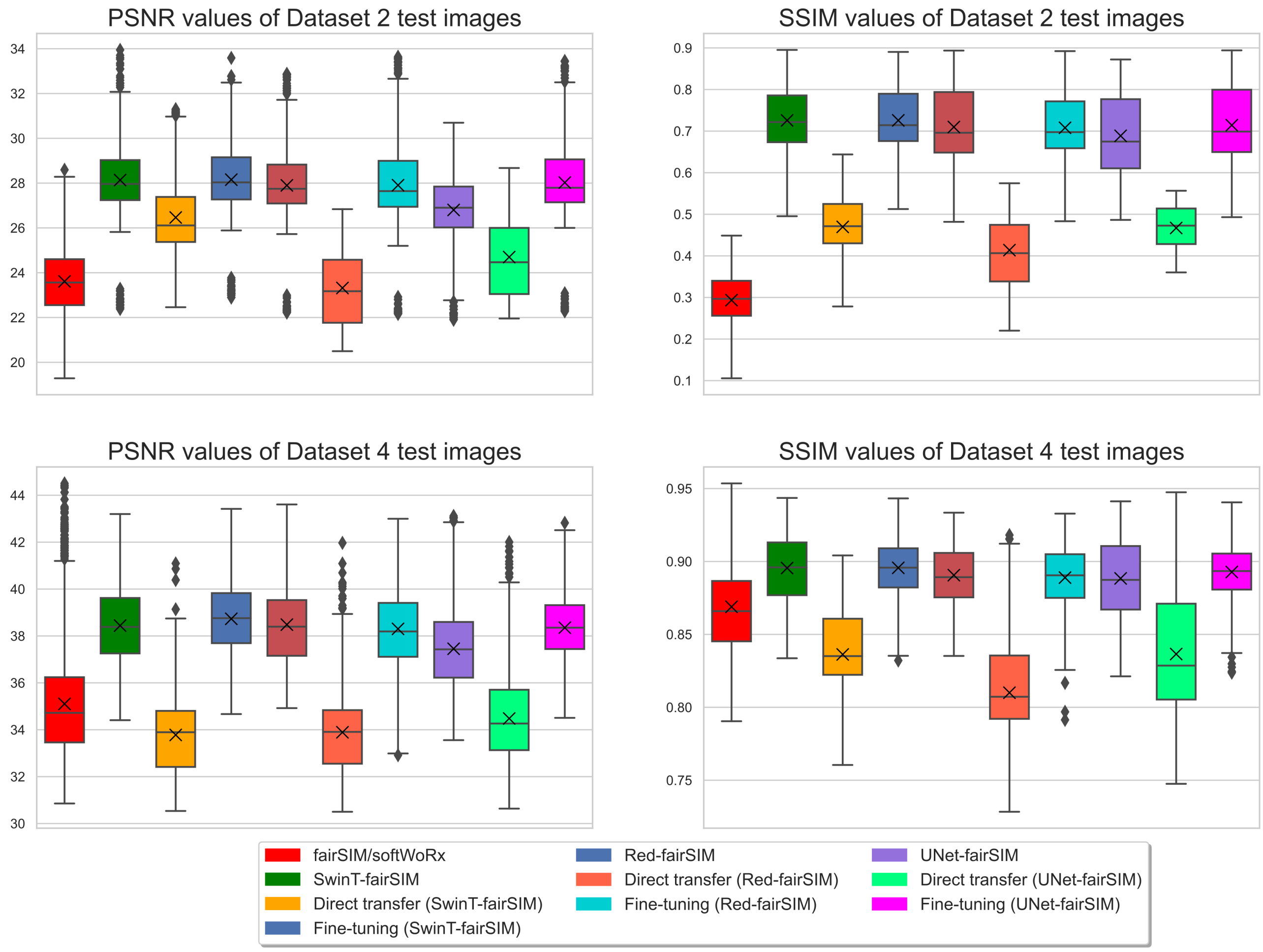

Figure 7. Boxplots of PSNR and SSIM values of test samples of dataset 2 and dataset 4 with different denoising algorithms and training strategies. The test data contains 500 images from noise level 4 for dataset 2 and 1380 images for dataset 4. The horizontal line inside each box plot represent the median value, whereas, the black cross display the mean values. The diamond-shaped black markers display the outlier observations. The color of the boxes in each subplot refers to the methods defined in the legend box.

construction artifacts [14]. In our previous studies [19], we already used the first three datasets for the denoising and super-resolution tasks. Here, we mainly focus on the denoising of SR-SIM images by SwinTransfomer-fairSIM and compare the results of this new method with the results of Red-fairSIM and UNet-fairSIM [19]. We also analyze two transfer learning strategies, direct transfer and fine-tuning of the pre-trained models of these network architectures.

To compare the performance of conventional training of these deep learning-based denoising methods with direct transfer and fine-tuning, we first train the SwinT-fairSIM, Red-fairSIM, and UNet-fairSIM networks separately with datasets 2 and 4 for 100 epochs. The results of all methods trained separately with both datasets are shown visually in Figure 6 and quantitatively in Table 2. The visual and quantitative results of SwinT-fairSIM show superiority over the Red-fairSIM and UNet-fairSIM methods. The resulting ROIs of both datasets from SwinT-fairSIM are more appealing and sharper than those of the other methods in Figure 6. The PSNR and SSIM values of SwinT-fairSIM are also slightly higher than those of Red-fairSIM and UNet-fairSIM. Overall, SwinT-fairSIM outperforms its counterparts on both datasets 2 and 4 after conventional training from scratch.

In the next step, we evaluate the direct transfer strategy by using these pre-trained models. In direct transfer, the model initially trained with dataset 2 is tested with the test samples of an alternative dataset (i.e., dataset 4), and vice versa. Similarly, in the assessment of the fine-tuning strategy, we re-trained the first and last layers of the pre-trained models with the alternative data to see the improvement in the generalization power of the pre-trained models. The resulting denoised images obtained by direct transfer of models trained on vesicle structures (dataset 4) to test images containing tubulin filaments (dataset 2) are displayed within block **(a)** of Figures 8 and 9. These images clearly show that the model trained on a specific type of noise and structure (i.e., vesicle images with honey comb pattern noise) is not able to produce a refined denoised image of another structure (i.e., tubulin filaments with MPG noise). Similarly, the models initially trained on tubulin filaments from dataset 2 are not able to properly produce the denoised images of dataset 4 with different structure and noise (see block **(b)** of Figures 8 and 9). However, it can be noticed that the pre-trained models try to replicate the filamentous structure of tubulin in the vesicle data (very prominent in block **(b)** of Figure 8). This is a clear indication that these deep learning based denoising models are not robust against different types of noise and structure. However, the outcomes of the fine-tuning approach are promising as shown in Figures 8 and 9. The ROIs 1 and 2 in column 6 of Figure 8 and also the ROIs in column 4 of Figure 9 show that the results of the fine-tuning strategy are very close to the results of training from scratch for the SwinT-fairSIM, Red-fairSIM and UNet-fairSIM

Table 2. Mean PSNR and SSIM values along with standard deviations (STD) of all experiments calculated on the noisy test images of datasets 2 and 4 (for dataset 2, noise level 4 was used). The test data contains 500 images for dataset 2 and 1380 images for dataset 4. The PSNR and SSIM values are calculated relative to the reference images, i.e. SR-SIM images reconstructed with fairSIM/softWoRx from raw SIM images with the highest signal-to-noise ratio. In the row entitled "fairSIM/softWoRx", PSNR and SSIM values are calculated for the direct fairSIM/softWoRx reconstruction of the noisy images. The rows entitled "SwinT-fairSIM","Red-fairSIM" and "UNet-fairSIM" show the results for the denoised test images after conventional training from scratch with the respective algorithms. In the direct transfer rows, the mean PSNR and SSIM values in the column "dataset 2" are calculated for models which are initially trained on dataset 4 and afterwards tested with the test samples from dataset 2. For the column "dataset 4", this is the other way round. The same principle holds for the rows for fine-tuning, only that the models are fine-tuned on the respective dataset before testing.

	Mean PSNR (STD) and SSIM (STD) values of test data			
	**dataset =2**		**dataset =4**	
	PSNR (STD)	SSIM (STD)	PSNR (STD)	SSIM (STD)
fairSIM/softWoRx	23.61 (1.54)	0.29 (0.07)	35.10 (2.71)	0.86 (0.03)
SwinT-fairSIM	28.19 (2.09)	0.72 (0.09)	38.44 (1.73)	0.89 (0.02)
Direct transfer (SwinT-fairSIM)	26.07 (1.91)	0.48 (0.07)	34.05 (2.24)	0.83 (0.03)
Fine-tuning (SwinT-fairSIM)	28.15 (1.87)	0.72 (0.08)	38.52 (1.50)	0.89 (0.02)
Red-fairSIM	27.97 (2.01)	0.71 (0.09)	38.43 (1.45)	0.89 (0.01)
Direct transfer (Red-fairSIM)	23.31 (1.68)	0.41 (0.07)	33.89 (1.93)	0.81 (0.03)
Fine-tuning (Red-fairSIM)	27.90 (2.14)	0.70 (0.09)	38.30 (1.60)	0.88 (0.02)
UNet-fairSIM	26.80 (1.65)	0.68 (0.10)	37.45 (1.79)	0.88 (0.02)
Direct transfer (UNet-fairSIM)	24.69 (1.54)	0.46 (0.05)	34.47 (2.24)	0.83 (0.04)
Fine-tuning (UNet-fairSIM)	28.02 (1.97)	0.71 (0.07)	38.35 (1.44)	0.89 (0.01)

algorithms. The overall comparison of all the approaches in Figures 8 and 9 demonstrates that the fine-tuning method is crucial for the Transformer and CNN-based denoising algorithms in the case of changes in structure or noise types. Thus, fine-tuning is inevitably required to profit from knowledge transfer.

Table 2 lists the average PSNR and SSIM values of the test images of the datasets 2 and 4 calculated for all the training and testing strategies of SwinT-fairSIM, Red-fairSIM, and UNet-fairSIM. Table 2 clearly points out the decline in the average PSNR and SSIM values on both datasets 2 and 4 after the application of direct transfer. However, the average PSNR and SSIM values show considerable improvement after fine-tuning. The average PSNR and SSIM values of the fine-tuned models in Table 2 are very close to the models that were trained from scratch (partly slightly better, partly slightly worse). In addition, Figure 7 shows boxplots of the PSNR and SSIM values of the test samples of both datasets with all different methods/strategies. The boxplots complement the mean values in Table 2 by depicting additional statistics. It can be observed that SwinT-fairSIM not only has often higher mean and median values, but also a more consistent interquartile range in all of the boxplots in Figure 7. In the SSIM boxplots of dataset 2, very few outliers are noticable overall.

Compared to conventional training, the fine-tuning strategy retrains only a subset of the entire set of model parameters. In this study, we retrained about 2.1 million parameters out of 4 million parameters of SwinT-fairSIM, while for Red-fairSIM and UNet-fairSIM, out of more than 1 million and 33 million training parameters, only 295K and 3.7 million parameters were retrained, respectively. We were able to achieve results comparable to those of conventional training after only 30 epochs as opposed to 100 epochs. Training times for dataset 2 on two Nvida V100 GPUs (32 GB) were (training from scratch vs. fine-tuning):

SwinT-fairSIM: 26 h vs. 3.25 h
Red-fairSIM: 24 h vs. 3 h
UNet-fairSIM: 18 h vs. 1.5 h

Therefore, in terms of computational requirements or memory consumption, it is advantageous to use fine-tuning instead of training from scratch. Fine-tuning is beneficial when the computational resources are limited.

Discussion

In summary, the contribution of this work is threefold: First, we publish novel datasets related to SIM microscopy, then we explore a Transformer-based algorithm for the restoration of SR-SIM images. Finally, we investigate the potential of the direct transfer and fine-tuning strategies for various deep learning-based denoising algorithms. Regarding datasets, we provide four novel datasets for testing denoising and super-resolution image reconstruction strategies with tubulin filaments and vesicle structures. These datasets contain a large number of raw SIM images and reconstructed twofold super-resolved SIM images and cover different noise levels, a wide range of fields of view, and varying degrees of structural complexity. High-quality data from the real world of microscopy are highly relevant for benchmarking and evaluating current and upcoming denoising and super-resolution methods.

Regarding our work with Transformers, we suggested the SwinT-fairSIM architecture to produce high-quality SR-SIM images from low SNR inputs. Importantly, we visually and quantitatively showed that the Transformer-based method can achieve mostly better results than the CNN-based Red-fairSIM and UNet-fairSIM methods (i.e., for the quantitative results, PSNR and SSIM values, see Table 2 and the boxplots in Figure 7). We demonstrated that SwinT-fairSIM can retrieve more well-preserved cell structures and texture information than the CNN-based methods, especially in the ROIs of Figure 6. This is an important finding, since most existing SR-SIM restoration

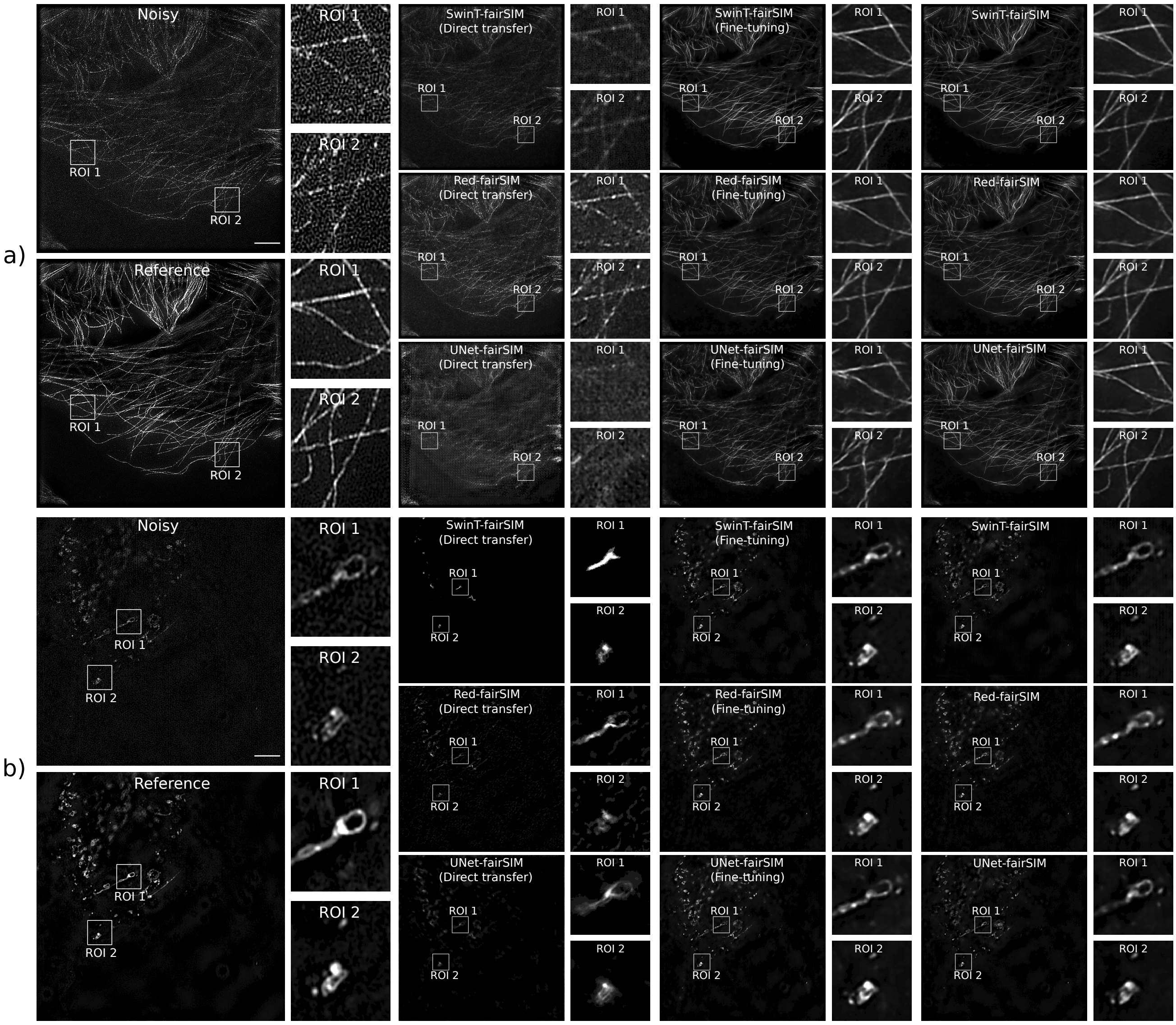

Figure 8. Blocks (**a**) and (**b**) show the results for test samples from dataset 2 (block **a**) and dataset 4 (block **b**) for different algorithms and learning strategies. The first and second columns of blocks **(a)** and **(b)** show the noisy and reference SR-SIM images, respectively, along with extracted and magnified regions of interest (ROIs). The noisy images are the test input for all algorithms. Columns 3 to 6 show the results of direct transfer and fine-tuning with SwinT-fairSIM, Red-fairSIM, and UNet-fairSIM. In direct transfer, the model is trained with dataset 2 and tested with the test sample of dataset 4 and vice versa. Similarly, in fine-tuning, the model is first pre-trained on dataset 2 and then fine-tuned and tested on dataset 4 and vice versa. Columns 7 and 8 show the results of all algorithms when trained from scratch on the respective dataset, which is also used for testing. The fourth, sixth, and eighth columns show the cropped and enlarged ROIs from the full-size SR-SIM images to the left of each ROI. The ROIs of size 100 pixels $\times$ 100 pixels have been upsampled to 300 pixels $\times$ 300 pixels for illustration purposes. Scale bar: 4 µm.

methods are based on CNNs [17, 18, 19, 21, 22, 23, 38, 39]. An explanation for the better performance of SwinT-fairSIM may be the size of the receptive fields of the different network architectures. In CNNs, the size of their receptive fields is limited by the size and stride of the filter kernels and the depth of the network. In contrast, Swin Transformers may obtain a larger receptive field by the shifted windows operation [55, 56].

In the last section of this work, we showed the limitations of Transformer- and CNN-based deep learning methods in the area of knowledge transfer when it comes to different types of noise and structure. In the direct transfer strategy, we simply evaluated the pre-trained networks with new test data. The direct transfer strategy exhibits very downgraded visual (in Figures 8 and 9) and quantitative (in Table 2 and Figure 7) results. However, the approach of fine-tuning enables these deep learning models to generalize well to other noise types and structures after retraining few layers. This holds for all learning algorithms and datasets in a similar way as shown in Figures 7, 8, 9 and in Table 2. All of the deep neural network architectures tested here are, at their core, encoder-decoder architectures (for SwinT-fairSIM, this applies at least to the compression head block and the decompression tail block). In these architectures, the initial and final layers are responsible for handling low-level features. Noise patterns can be interpreted as low-level features that are separated from the valuable input during the encoding process so that they can be selectively suppressed at a later stage during decoding. Accordingly, we retrained only the initial and final layers of all three types of neural networks to achieve results on par with training from scratch. It is worth mentioning that we also attempted to retrain only the first, middle, or last layers during first exploratory research, and also varied the number of retrained layers, but the results obtained in this way were not competitive. Since fine-tuning often saves computation time and often requires fewer learning samples, we conclude that fine-tuning of pre-trained models has advantages over conventional train-

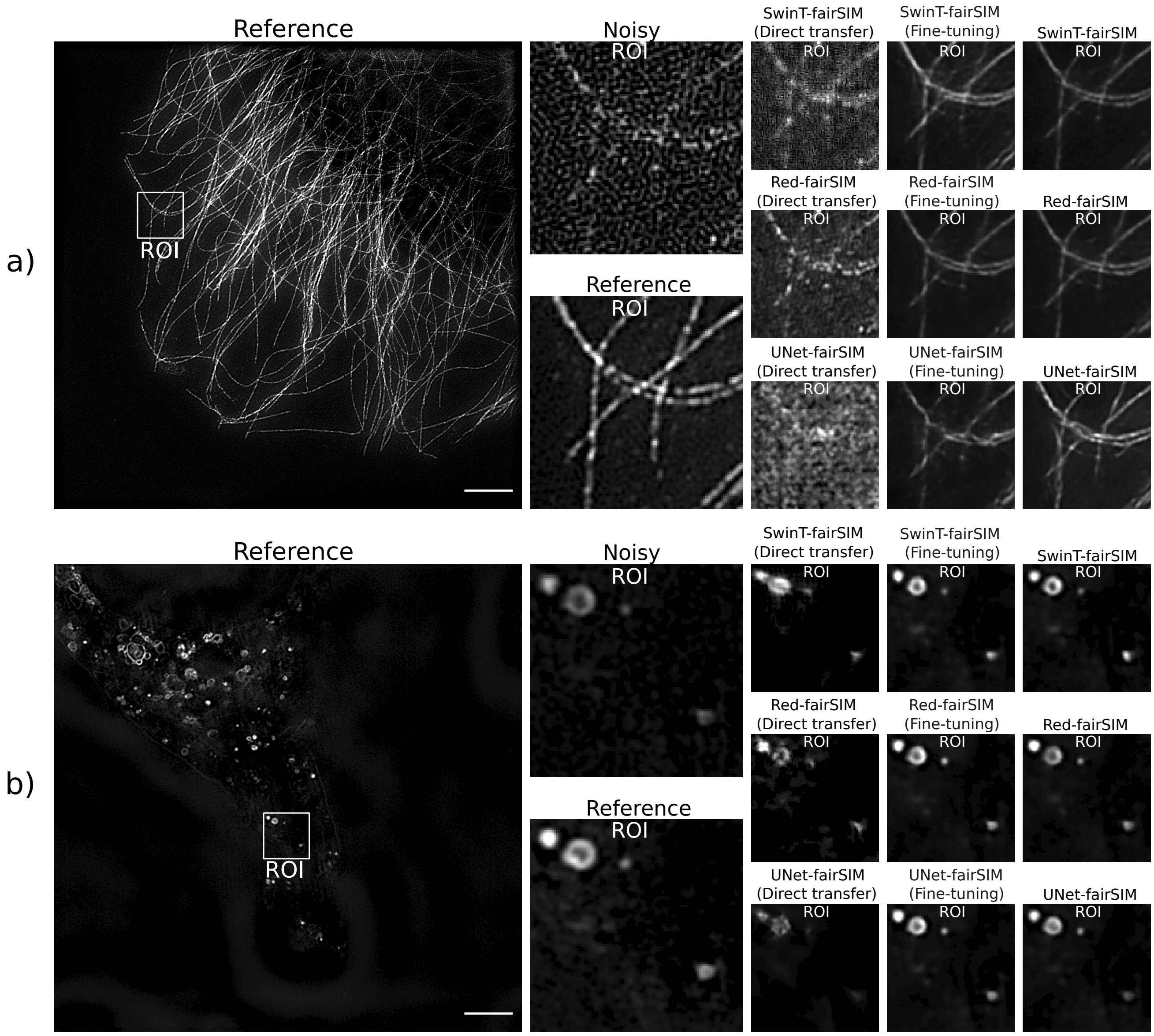

Figure 9. Two more test samples from datasets 2 and 4 are shown in this figure. Both blocks (**a**) and (**b**) contain the reference SR-SIM images along with the resultant denoised ROIs which are extracted from the full size denoised images of all the methods used in this work. The extracted ROIs are upsampled from 100 pixels $\times$ 100 pixels to 300 pixels $\times$ 300 pixels for illustration purposes. Scale bar: 4 µm.

ing from scratch, at least when the difference between the task domains is not huge. In contrast, direct transfer obviously fails because the learned detectors for low-level features (noise patterns and typical simple visual features of the main cell structures) are too much tuned to the original training data.

There are still some limitations of this work. First, while our datasets are useful for various image restoration tasks and contain more samples than many of the datasets previously published in the field, they are limited to two structures, tubulin filaments and vesicle structures. Second, our results indicate the superiority of SwinT-fairSIM over Red-fairSIM and UNet-fairSIM. However, this improvement leads to higher computational costs. Third, fine-tuning strategies provide similar results to training from scratch at lower cost, but it is often difficult to find task-specific pre-trained networks. Finally, slice-by-slice denoising of 3D SIM images focuses exclusively on the lateral plane; despite this limitation, it was shown that the presented approach can still be very useful for denoising 3D SIM structures.

Conclusion

In this work, we first presented a series of datasets for SIM image restoration tasks such as denoising and super-resolution. Additionally, we proposed a new algorithm based on the Swin Transformer, “SwinT-fairSIM”, for the denoising of SR-SIM images. We showed that this Transformer-based algorithm outperforms CNN-based denoising algorithms both visually and quantitatively. This suggests that Transformer-based denoising algorithms can play an important role in the field of SR-SIM microscopy. Similarly, we evaluated different knowledge transfer strategies such as direct transfer and fine-tuning for Transformer- and CNN-based denoising algorithms. For the direct transfer strategy, we noticed a decline in the performance of these algorithms. However, we were able to recover the declined performance by the fine-tuning strategy. This clearly indicates that the Transformer- and CNN-based denoising methods require retraining of some initial and final layers of the pre-trained models when applied to new biological structures and noise types. This retraining requires fewer epochs than the conventional training from scratch. This holds

also for the novel SwinT-fairSIM denoising algorithm which outperforms all CNN-based algorithm also in the fine-tuning regime.

Potential implications

The implementation of different deep learning methods requires a large quantity of images in order to train the underlying models. We believe that our published datasets will help the research community to develop new deep learning based methods and evaluate the existing methods by either training from scratch or by applying fine-tuning. These datasets can be especially used for image denoising and super-resolution tasks.

Data and code Availability

All the raw and reconstructed datasets which are discussed in this article are available on GigaDB ["link will be uploaded after the allocation of space from Giga Database"]. All the datasets are distributed under the Creative Commons CCO waiver, with a request for attribution. The CCO permits the unrestricted reuse, distribution and reproduction, provided the original work is properly cited. Similarly, all the codes which are used during this work are publicly available [42].

Declarations

Abbreviations

SIM: structured illumination microscopy; SR-SIM: super-resolution structured illumination microscopy; NA: numerical aperture; PSNR: peak-signal-to-noise ratio; SSIM: structural similarity index measurement; MSE: mean square error; SNR: signal-to-noise ratio; W2S: wide-field2SIM; fairSIM: free analysis and interactive reconstruction for structured illumination microscopy.

Competing Interests

The authors declare no competing interests.

Funding

This work was supported by the EFRE-NRW funding programme ”Forschungsinfrastrukturen” (grant no. 34.EFRE-0300180) and partly conducted within the framework of the project “SAIL: SustAInable Lifecycle of Intelligent SocioTechnical Systems” (grant no. NW21-059B). SAIL is receiving funding from the programme “Netzwerke 2021”, an initiative of the Ministry of Culture and Science of the State of Northrhine Westphalia. T.-C.W. and T.H. were supported by funding from the European Union’s Horizon 2020 research and innovation program under the Marie Sklodowska-Curie Grant Agreements No. 642157, project “TOLLerant”, and No. 766181. project "DeLIVER". T.H. also acknowledges funding by the Deutsche Forschungsgemeinschaft (DFG, German Science Foundation)—project number 415832635.

Author’s Contributions

Z.H.S. carried out the preprocessing of datasets for the machine learning work, created the figures, and wrote the manuscript. D.T. conducted experiments to find the optimal hyperparameters for the CNN-based approaches. T.-C.W. recorded the raw SIM images of dataset 1. M.M. supported Z.H.S. in the reconstruction of data for datasets 2 and 3 with fairSIM. W.H. recorded and reconstructed the images of dataset 4. W.S. and T.H. supervised the research. W.S., T.H., M.M., and W.H. contributed to the editing of the manuscript. In addition, they discussed the experimental results together with Z.H.S. All authors discussed and agreed on the final manuscript.

Acknowledgements

The authors would like to thank Dr. Matthias Fricke from the Center for Applied Data Science (CfADS) at Bielefeld University of Applied Sciencesn and Arts for providing access to their GPU compute cluster. We would also like to thank Dr. Olaf Kaczmarek and Markus Klappenback for providing access to the GPU compute cluster at Bielefeld University.

References

1. Hirvonen LM, Wicker K, Mandula O, Heintzmann R. Structured illumination microscopy of a living cell. European Biophysics Journal 2009;38(6):807–812.
2. Hell SW, Sahl SJ, Bates M, Zhuang X, Heintzmann R, Booth MJ, et al. The 2015 super-resolution microscopy roadmap. Journal of Physics D: Applied Physics 2015;48(44):443001.
3. Heintzmann R, Huser T. Super-resolution structured illumination microscopy. Chemical reviews 2017;117(23):13890–13908.
4. Demmerle J, Innocent C, North AJ, Ball G, Müller M, Miron E, et al. Strategic and practical guidelines for successful structured illumination microscopy. Nature protocols 2017;12(5):988–1010.
5. Schermelleh L, Ferrand A, Huser T, Eggeling C, Sauer M, Biehlmaier O, et al. Super-resolution microscopy demystified. Nature cell biology 2019;21(1):72–84.
6. Gustafsson MG. Surpassing the lateral resolution limit by a factor of two using structured illumination microscopy. Journal of microscopy 2000;198(2):82–87.
7. Müller M, Mönkemöller V, Hennig S, Hübner W, Huser T. Open-source image reconstruction of super-resolution structured illumination microscopy data in ImageJ. Nature communications 2016;7(1):1–6.
8. Lal A, Shan C, Xi P. Structured illumination microscopy image reconstruction algorithm. IEEE Journal of Selected Topics in Quantum Electronics 2016;22(4):50–63.
9. Brown PT, Kruithoff R, Seedorf GJ, Shepherd DP. Multicolor structured illumination microscopy and quantitative control of polychromatic light with a digital micromirror device. Biomedical Optics Express 2021;12(6):3700–3716.
10. Ströhl F, Kaminski CF. Frontiers in structured illumination microscopy. Optica 2016;3(6):667–677.
11. Zheng X, Zhou J, Wang L, Wang M, Wu W, Chen J, et al. Current challenges and solutions of super-resolution structured illumination microscopy. APL Photonics 2021;6(2):020901.
12. Huang X, Fan J, Li L, Liu H, Wu R, Wu Y, et al. Fast, long-term, super-resolution imaging with Hessian structured illumination microscopy. Nature biotechnology 2018;36(5):451–459.
13. Hoffman DP, Betzig E. Tiled reconstruction improves structured illumination microscopy. BioRxiv 2020;.
14. Smith CS, Slotman JA, Schermelleh L, Chakrova N, Hari S, Vos Y, et al. Structured illumination microscopy with noise-controlled image reconstructions. Nature methods

2021;18(7):821–828.

15. Gustafsson MG, Shao L, Carlton PM, Wang CR, Golubovskaya IN, Cande WZ, et al. Three-dimensional resolution doubling in wide-field fluorescence microscopy by structured illumination. Biophysical journal 2008;94(12):4957–4970.
16. Shah ZH, Müller M, Hammer B, Huser T, Schenck W. Impact of different loss functions on denoising of microscopic images. In: 2022 International Joint Conference on Neural Networks (IJCNN) IEEE; 2022. p. 1–10.
17. Jin L, Liu B, Zhao F, Hahn S, Dong B, Song R, et al. Deep learning enables structured illumination microscopy with low light levels and enhanced speed. Nature communications 2020;11(1):1–7.
18. Chen X, Li B, Jiang S, Zhang T, Zhang X, Qin P, et al. Accelerated Phase Shifting for Structured Illumination Microscopy based on Deep Learning. IEEE Transactions on Computational Imaging 2021;7:700–712.
19. Shah ZH, Müller M, Wang TC, Scheidig PM, Schneider A, Schüttpelz M, et al. Deep-learning based denoising and reconstruction of super-resolution structured illumination microscopy images. Photonics Research 2021;9(5):B168–B181.
20. Belthangady C, Royer LA. Applications, promises, and pitfalls of deep learning for fluorescence image reconstruction. Nature methods 2019;16(12):1215–1225.
21. Qiao C, Li D, Guo Y, Liu C, Jiang T, Dai Q, et al. Evaluation and development of deep neural networks for image super-resolution in optical microscopy. Nature Methods 2021;18(2):194–202.
22. Xypakis E, Gosti G, Giordani T, Santagati R, Ruocco G, Leonetti M. Deep learning for blind structured illumination microscopy. Scientific Reports 2022;12(1):8623.
23. Liu T, Liu J, Li D, Tan S. Improving Reconstruction of Structured Illumination Microscopy Images Via Dual-Domain Learning. IEEE Journal of Selected Topics in Quantum Electronics 2023;.
24. Qiao C, Chen X, Zhang S, Li D, Guo Y, Dai Q, et al. 3D structured illumination microscopy via channel attention generative adversarial network. IEEE Journal of Selected Topics in Quantum Electronics 2021;27(4):1–11.
25. Vaswani A, Shazeer N, Parmar N, Uszkoreit J, Jones L, Gomez AN, et al. Attention is all you need. Advances in neural information processing systems 2017;30.
26. Sutskever I, Vinyals O, Le QV. Sequence to sequence learning with neural networks. Advances in neural information processing systems 2014;27.
27. Dehghani M, Gouws S, Vinyals O, Uszkoreit J, Kaiser L. Universal Transformers. In: International Conference on Learning Representations;. .
28. Vyas A, Katharopoulos A, Fleuret F. Fast transformers with clustered attention. Advances in Neural Information Processing Systems 2020;33:21665–21674.
29. Wang B, Shang L, Lioma C, Jiang X, Yang H, Liu Q, et al. On position embeddings in bert. In: International Conference on Learning Representations; 2021. .
30. Cheng X, Lin H, Wu X, Shen D, Yang F, Liu H, et al. Mltr: Multi-label classification with transformer. In: 2022 IEEE International Conference on Multimedia and Expo (ICME) IEEE; 2022. p. 1–6.
31. Carion N, Massa F, Synnaeve G, Usunier N, Kirillov A, Zagoruyko S. End-to-end object detection with transformers. In: Computer Vision–ECCV 2020: 16th European Conference, Glasgow, UK, August 23–28, 2020, Proceedings, Part I 16 Springer; 2020. p. 213–229.
32. Sun Z, Cao S, Yang Y, Kitani KM. Rethinking transformer-based set prediction for object detection. In: Proceedings of the IEEE/CVF international conference on computer vision; 2021. p. 3611–3620.
33. Chen H, Wang Y, Guo T, Xu C, Deng Y, Liu Z, et al. Pre-trained image processing transformer. In: Proceedings of the IEEE/CVF Conference on Computer Vision and Pattern Recognition; 2021. p. 12299–12310.
34. Ali AM, Benjdira B, Koubaa A, El-Shafai W, Khan Z, Boulila W. Vision transformers in image restoration: A survey. Sensors 2023;23(5):2385.
35. Liu Z, Lin Y, Cao Y, Hu H, Wei Y, Zhang Z, et al. Swin transformer: Hierarchical vision transformer using shifted windows. In: Proceedings of the IEEE/CVF international conference on computer vision; 2021. p. 10012–10022.
36. Liang J, Cao J, Sun G, Zhang K, Van Gool L, Timofte R. Swinir: Image restoration using swin transformer. In: Proceedings of the IEEE/CVF international conference on computer vision; 2021. p. 1833–1844.
37. Zhang Y, Zhu Y, Nichols E, Wang Q, Zhang S, Smith C, et al. A poisson-gaussian denoising dataset with real fluorescence microscopy images. In: Proceedings of the IEEE/CVF Conference on Computer Vision and Pattern Recognition; 2019. p. 11710–11718.
38. Zhou R, Helou ME, Sage D, Laroche T, Seitz A, Süsstrunk S. W2S: microscopy data with joint denoising and super-resolution for widefield to SIM mapping. In: European Conference on Computer Vision Springer; 2020. p. 474–491.
39. Hagen GM, Bendesky J, Machado R, Nguyen TA, Kumar T, Ventura J. Fluorescence microscopy datasets for training deep neural networks. GigaScience 2021;10(5):giab032.
40. Karras C, Smedh M, Förster R, Deschout H, Fernandez-Rodriguez J, Heintzmann R. Successful optimization of reconstruction parameters in structured illumination microscopy–a practical guide. Optics Communications 2019;436:69–75.
41. Mueller M, Free Analysis and Interactive Reconstruction for Structured Illumination Microscopy. GitHub; 2016. https://github.com/fairSIM/fairSIM.git.
42. Shah ZH, Evaluation of Swin Transformer and knowledge transfer for denoising of super-resolution structured illumination microscopy data. GitHub; 2023. https://github.com/ZafranShah/Denoising_and_fine_tuning_of_SR-SIM_data.
43. Shah ZH, Evaluation of Swin Transformer and knowledge transfer for denoising of super-resolution structured illumination microscopy data. zenodo; 2023. https://doi.org/10.5281/zenodo.7626173.
44. Ching JY, Wong AKC, Chan KCC. Class-dependent discretization for inductive learning from continuous and mixed-mode data. IEEE Transactions on Pattern Analysis and Machine Intelligence 1995;17(7):641–651.
45. Hussain M, Bird JJ, Faria DR. A study on cnn transfer learning for image classification. In: UK Workshop on computational Intelligence Springer; 2018. p. 191–202.
46. Tan C, Sun F, Kong T, Zhang W, Yang C, Liu C. A survey on deep transfer learning. In: International conference on artificial neural networks Springer; 2018. p. 270–279.
47. Bengio Y. Deep learning of representations for unsupervised and transfer learning. In: Proceedings of ICML workshop on unsupervised and transfer learning JMLR Workshop and Conference Proceedings; 2012. p. 17–36.
48. Ng HW, Nguyen VD, Vonikakis V, Winkler S. Deep learning for emotion recognition on small datasets using transfer learning. In: Proceedings of the 2015 ACM on international conference on multimodal interaction; 2015. p. 443–449.
49. Nogueira K, Penatti OA, Dos Santos JA. Towards better exploiting convolutional neural networks for remote sensing scene classification. Pattern Recognition 2017;61:539–556.
50. Yosinski J, Clune J, Bengio Y, Lipson H. How transferable

are features in deep neural networks? Advances in neural information processing systems 2014;27.

51. Mao X, Shen C, Yang YB. Image restoration using very deep convolutional encoder-decoder networks with symmetric skip connections. Advances in neural information processing systems 2016;29.
52. Ronneberger O, Fischer P, Brox T. U-net: Convolutional networks for biomedical image segmentation. In: International Conference on Medical image computing and computer-assisted intervention Springer; 2015. p. 234–241.
53. Hore A, Ziou D. Image quality metrics: PSNR vs. SSIM. In: 2010 20th international conference on pattern recognition IEEE; 2010. p. 2366–2369.
54. Setiadi DRIM. PSNR vs SSIM: imperceptibility quality assessment for image steganography. Multimedia Tools and Applications 2021;80(6):8423–8444.
55. Parmar N, Vaswani A, Uszkoreit J, Kaiser L, Shazeer N, Ku A, et al. Image transformer. In: International conference on machine learning PMLR; 2018. p. 4055–4064.
56. Ranftl R, Bochkovskiy A, Koltun V. Vision transformers for dense prediction. In: Proceedings of the IEEE/CVF International Conference on Computer Vision; 2021. p. 12179–12188.

Manuscript
Click here to access/download;Manuscript;Manuscript_GIGA-D-23-00044_Revision_2.pdf

OXFORD (GIGA)^n SCIENCE

GigaScience, 2023, 1–15

doi: xx.xxxx/xxxx
Research

RESEARCH

Evaluation of Swin Transformer and knowledge transfer for denoising of super-resolution structured illumination microscopy data

Zafran Hussain Shah[1,*], Marcel Müller[2], Wolfgang Hübner[2], Tung-Cheng Wang[2,3], Daniel Telman[1], Thomas Huser[2] and Wolfram Schenck[1,*]

[1]Faculty of Engineering and Mathematics, Bielefeld University of Applied Sciences and Arts, Interaktion 1, 33619 Bielefeld, Germany and [2]Faculty of Physics, Bielefeld University, Universitätsstr. 25, 33615 Bielefeld, Germany and [3]Leica Microsystems CMS GmbH, Am Friedensplatz 3, 68165 Mannheim, Germany

[*]zafran_hussain.shah@hsbi.de; wolfram.schenck@hsbi.de

Abstract

In recent years, convolutional neural network (CNN)-based methods have shown remarkable performance in the denoising and reconstruction of super-resolved structured illumination microscopy (SR-SIM) data. Therefore, CNN-based architectures have been the main focus of existing studies. Recently, however, an alternative and highly competitive deep learning architecture, Swin Transformer, has been proposed for image restoration tasks. In this work, we present SwinT-fairSIM, a novel method for restoring SR-SIM images with low signal-to-noise ratio (SNR) based on Swin Transformer. The experimental results show that SwinT-fairSIM outperforms previous CNN-based denoising methods. Furthermore, the generalization capabilities of deep learning methods for image restoration tasks on real fluorescence microscopy data have not been fully explored yet, i.e., the extent to which trained artificial neural networks are limited to specific types of cell structures and noise. Therefore, as a second contribution, we benchmark two types of transfer learning, i.e., direct transfer and fine-tuning, in combination with SwinT-fairSIM and two CNN-based methods for denoising SR-SIM data. Direct transfer does not prove to be a viable strategy, but fine-tuning achieves results comparable to conventional training from scratch, while saving computational time and potentially reducing the amount of required training data. As a third contribution, we publish four datasets of raw SIM images and already reconstructed SR-SIM images. These datasets cover two different types of cell structures, tubulin filaments and vesicle structures. Different noise levels are available for the tubulin filaments. These datasets are structured in such a way that they can be easily used by the research community for research on denoising, super-resolution, and transfer learning strategies.

Key words: Structured illumination microscopy; Fluorescence microscopy; Deep learning; Tranformers; Swin Transformer; SwinIR; Convolutional neural networks; Denoising; Image restoration; Transfer learning; Fine-tuning

Introduction

In optical microscopy, super-resolution structured illumination microscopy (SR-SIM) plays a significant role in the field of biological and biomedical studies to analyze living cells and biological specimens with characteristic features below the resolution limit of classical microscopes (approx. 250 nm for high-end systems with oil-immersion objective lenses). Structures of interest include e.g. the internal organelles of mitochondria, cellular cytoskeleton, virus particles, or small vesicles [1, 2, 3, 4, 5]. SR-SIM is an important super-resolution approach to disentangle complex biological cellular structures with an up to twofold enhancement of spatial resolution beyond the

Compiled on: September 22, 2023.
Draft manuscript prepared by the author.

diffraction limit. During the process of super-resolution imaging, SR-SIM involves the illumination of the biological sample with spatially patterned light following a sinusoidal intensity distribution. A series of raw images with typically 3 or 5 different phase positions and 3 angles of orientation of the illumination pattern are typically acquired [6]. Subsequently, frequency-domain based image reconstruction algorithms, e.g. implemented in software packages such as fairSIM [7], OpenSIM [8], and Python-based packages [9] are applied to a set of raw SIM images to generate the final twofold super-resolved images. SR-SIM has many advantages over other super-resolution methods e.g., it does not require special sample preparation, it permits the use of conventional fluorophores in multiple colors, simultaneously, and it allows for imaging at high speed and large fields-of-view (FOV) while being compatible with live cell samples by making efficient use of low illumination intensity levels [10, 11]. The conventional SR-SIM reconstruction algorithms have some limitations whenever the signal-to-noise level of the raw images is poor due to low fluorescence emission or short exposure times [12]. In general, in fluorescence microscopy weak emission due to, e.g. low labeling densities, high light scattering or absorption, or optical aberrations is often encountered [13, 14]. In the case of several super-resolution fluorescence microscopy methodologies, this leads to low signal-to-noise ratio (SNR), and poor image reconstructions or artifacts. Nevertheless, SR-SIM imaging provides better resolution and optical sectioning abilities than confocal microscopy with a just minimally larger number of raw images to be acquired [15].

In the last decade, deep learning methods have become generally accepted in image processing. In addition, they are being used with increasing success for the restoration of SR-SIM images, e.g. for denoising [16, 17, 18, 19, 20]. Qiao et al. proposed a generative adversarial network (GAN) based deep Fourier channel attention network (DFCAN) method to reconstruct SR-SIM images under low SNR conditions [21]. Xypakis et al. introduced a custom convolutional neural network (CNN) architecture for blind-SIM: BS-CNN [22]. Liu et al. proposed a dual-domain learning strategy for the reconstruction of SIM images [23]. Authors in [24] suggested another GAN based channel attention generative adversarial network (caGAN) to improve the quality of 3D-SIM reconstruction using fewer raw samples of low SNR.

Recently, so-called Transformers have shown great success in various natural language processing tasks [25, 26, 27, 28, 29]. Since then, as an alternative to CNNs, Transformer-based architectures have been adapted to computer vision tasks as well, such as classification [30], detection [31, 32], and image restoration [33]. Vision Transformers for the image restoration typically divide each image into fixed-size patches and process each patch independently to limit computational complexity [34], which results in the introduction of border artifacts around each patch in the restored image. The Swin Transformer overcomes this shortcoming by integrating a shifted window operation along with a few CNN layers in the Transformer architecture [35]. Swin serves as the basis for the Shifted window Image Restoration (SwinIR) method [36], which has been proposed especially for various image restoration tasks. Although these latest Transformers outperform CNN-based methods in conventional image restoration to some extent, they were never explored for the restoration of high-resolution SR-SIM microscopy images. Therefore, during this work, we propose a SwinIR based Transformer architecture named "SwinT-fairSIM" to denoise SR-SIM images under low SNR conditions.

Both CNN- and Transformer-based methods require a large number of images to train the underlying models. In the field of microscopy, the sheer size and storage requirements of datasets of high-resolution reconstructed microscopic images make them difficult to produce and typically not publicly available. The few open source datasets available are mostly related to wide-field microscopy and contain a relatively small number of images. For example, Zhang et al. [37] collected three wide-field microscopy datasets purely for denoising tasks without providing high-resolution ground truth images obtained by SR-SIM technology. They used image averaging to generate ground truth data with high SNR images. Zhou et al. [38], on the other hand, published a dataset called "Widefield2SIM" using wide-field fluorescence microscopy. They captured 120 different fields of view (FOV) with 400 low SNR images for each FOV and generated high-resolution ground truth data using SR-SIM imaging technology. Qiao et al. [21] presented the "BioSR" dataset consisting of 2200 pairs of low-resolution (LR) raw data and high-resolution (HR) data. Four different biological structures (CCPs, ER, MTs, F-actin), nine signal levels (15-600 average photon count), and two upscaling-factors (linear SIM and non-linear SIM) are covered in the BioSR dataset according to Qiao et al. Similarly, Hagen et al. [39] published a variety of datasets which were collected using wide-field and confocal microscopy. They captured various fluorescently labeled structures such as actin, mitochondria, membrane, and nuclei with low and high SNR. However, their collection of datasets consists of significantly fewer images than the BioSR data collection.

Here, we present a series of datasets that are related to SR-SIM microscopy for image restoration tasks. These datasets cover two types of biological structures, tubulin filaments, and vesicles, with several fields-of-view for the denoising, super-resolution, and joint denoising and super-resolution tasks. In our datasets, noisy input and reference output images were obtained by using SR-SIM reconstruction algorithms. We believe that our datasets will be helpful for the research community to benchmark different deep-learning based denoising and super-resolution (SR) methods, not least because of the rather large number of provided samples.

Finally, we show how some of these datasets can be used to demonstrate the generalization capabilities of image restoration algorithms in the field of SIM microscopy. To this end, we apply the concept of transfer learning to three algorithms for the denoising of SR-SIM data. Two of these algorithms are based on CNNs and were proposed by us in [16]. They are called "Red-fairSIM" and "UNet-fairSIM". The third algorithm is the above-mentioned Transformer architecture "SwinT-fairSIM", which is first proposed in this paper. With respect to transfer learning, we compare two methods from this area: Direct transfer and fine-tuning. In direct transfer, a model pre-trained on one type of data is used for inference on a related but different type of data. In fine-tuning, part of the pre-trained model is retrained on the new type of data before inference. In previous work, we were already able to show that some CNN-based algorithms for the denoising of SR-SIM images are robust to different noise levels and SIM modes, e.g. varying pattern spacings at different illumination wavelengths [19]. However, due to the limited amount of SR-SIM data available at that time, we were not able to explore the techniques of transfer learning in more depth. Thus, here, we also aim to answer the following questions related to direct transfer and fine-tuning: (1) If a model is trained on a specific biological structure with a specific type of noise, will it also generalize well to denoising another, different structure with another type of noise? And: (2) Is fine-tuning of a pre-trained model (i.e., previously trained on one type of structure and noise) more effective than training the model from scratch? To answer these questions, we have conducted a series of experiments which are discussed in later sections.

In particular, the contribution of this work is threefold:

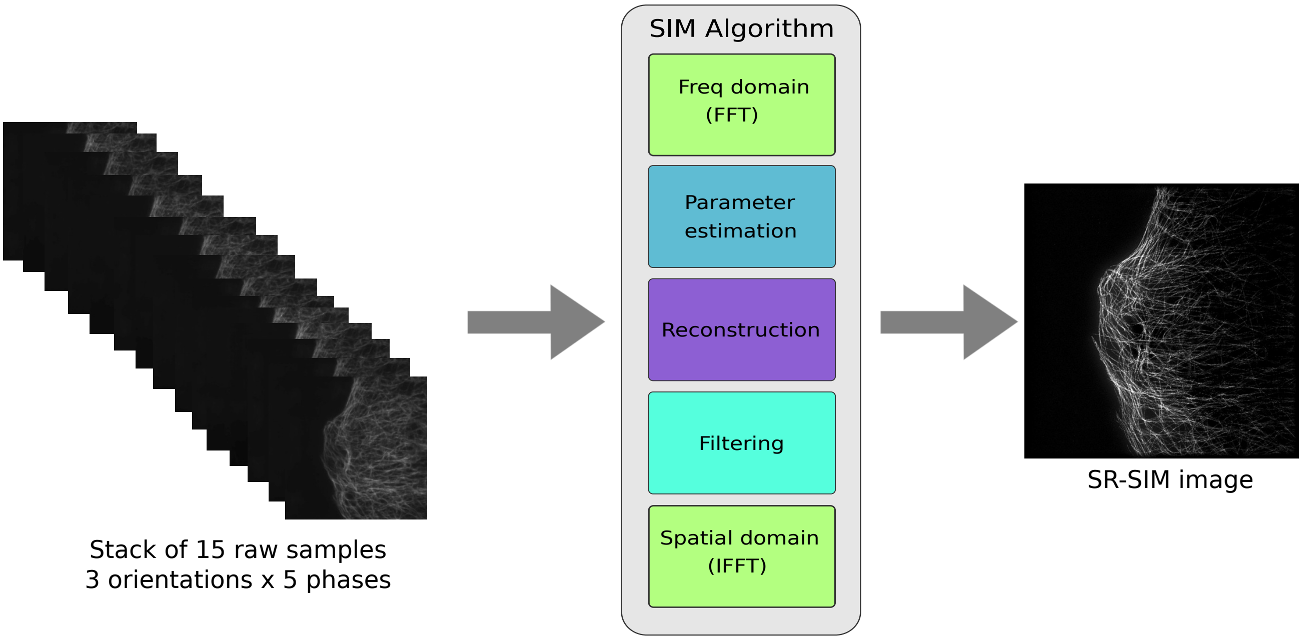

Figure 1. The architecture of the image reconstruction algorithms which are used to generate the reconstructed SR-SIM images in this work. Both reconstruction algorithms (i.e., as implemented in the fairSIM and softWoRx software) are based on the SIM algorithm. During the reconstruction of SR-SIM images, a stack of 15 raw SIM images of size 512 × 512 pixels is processed by the respective software which generates the high-resolution SR-SIM image of size 1024 × 1024 pixels as output.

First, we present high-resolution SR-SIM datasets for image denoising and super-resolution tasks. Second, we propose a method based on the SwinIR architecture for denoising SR-SIM images. Third, we evaluate the potential of direct transfer and fine-tuning for different Transformer- and CNN-based models.

Materials and Methods

SR-SIM microscopy and sample preparation

The raw SIM images for all the datasets were acquired using a DeltaVision OMX V4 (GE Healthcare, Chicago, IL, USA) 3D-SIM imaging system. The DeltaVision OMX V4 imaging system is an implementation of 3-beam 3D-SIM [15], providing both lateral and axial modulation of the excitation pattern, and thus allows to increase both the lateral and axial resolution of the imaging process (as compared to 2-beam SIM, where only the lateral resolution is increased). The system is equipped with four excitation laser lines (405nm, 488nm, 561nm and 642nm), and four sCMOS cameras to detect the emitted fluorescence light in four channels. The camera pixel size of 6.5 µm and an overall magnification of 82x yields a fixed effective pixel size of 80 nm. The magnification cannot be changed by the user, and has been set by the manufacturer to fulfill the Nyquist criterion, thus not loosing spatial information in the sampling process. While fulfilling the Nyquist sampling criterion only sets a maximum pixel size, pixel sizes much smaller than the limit are not used, as this would compromise both SNR (each pixel contributing a constant amount of read noise) and field of view (as the overall feasible pixel count is finite). To prepare the raw SIM image data (datasets 1-3) of the tubulin cytoskeleton, U2OS cells were cultured in DMEM supplemented with 10%FBS and grown on round coverslips of 170±5 µm thickness (No. 1.5H). Cells were fixed with 4% PFA for 15 min., followed by PBS washes, and permeabilization with 0.5% Triton-X100 for 3 min. Another two rounds of PBS washes were done before blocking with 3% BSA. For immunolabeling of the tubulin microfilaments, cells were stained with anti-tubulin antibody (Invitrogen Cat. No. 322500) 1:400 for 2 hr at room temperature, followed by a PBS wash and one additional hour of incubation with Alexa 488-conjugated anti-mouse IgG 1:400. Afterwards the cells were then briefly washed with PBS before Vectashield was applied to embed the coverslip onto a standard microscopy glass slide for imaging. For the preparation of raw SIM images with vesicle structures (dataset 4), U2OS cells were transfected with Lipofectamine 3000 according to the manufacturers protocol (Thermofisher Cat. No. L3000-001) together with a plasmid expressing the vesicular Lamp1 protein fused to the fluorescent protein mScarlet. After 24 hr transfection the cells were fixed with 4% PFA for 10 min., followed by PBS washes and Vectashield mounting prior to imaging. The vesicular structures represent lysosomes. During the data collection, the illumination intensity as well as the camera exposure time remained fixed. As the cameras as also actively cooled, thermal- and read-noise components should not vary during time-lapse image acquisition. Also, as typical for modern sCMOS cameras, thermal- and read-noise components are small (in the order of single electrons per read-out) compared to the Poisson noise, i.e. the stochastic distribution of detected photons generated by the quantum nature of light propagation. The dramatic drop in signal-to-noise ratio during data acquisition is due to photobleaching and phototoxicity in the sample.

Dataset preprocessing and image reconstruction

Dataset 1 is a raw dataset without any image processing or reconstruction applied to the raw SIM images. The images in the datasets 2 and 3 are reconstructed by using the open-source fairSIM reconstruction algorithm as shown in Figure 1. fairSIM implements a single-slice (2D) SR-SIM image reconstruction algorithm [7]. It works in three steps: parameter estimation, reconstruction, and filtering. The mathematical and algorithmic details of the fairSIM reconstruction method are explained in the original publication [7]. A synthetic optical transfer function, with NA = 1.4, λ = 525 nm, a = 0.31

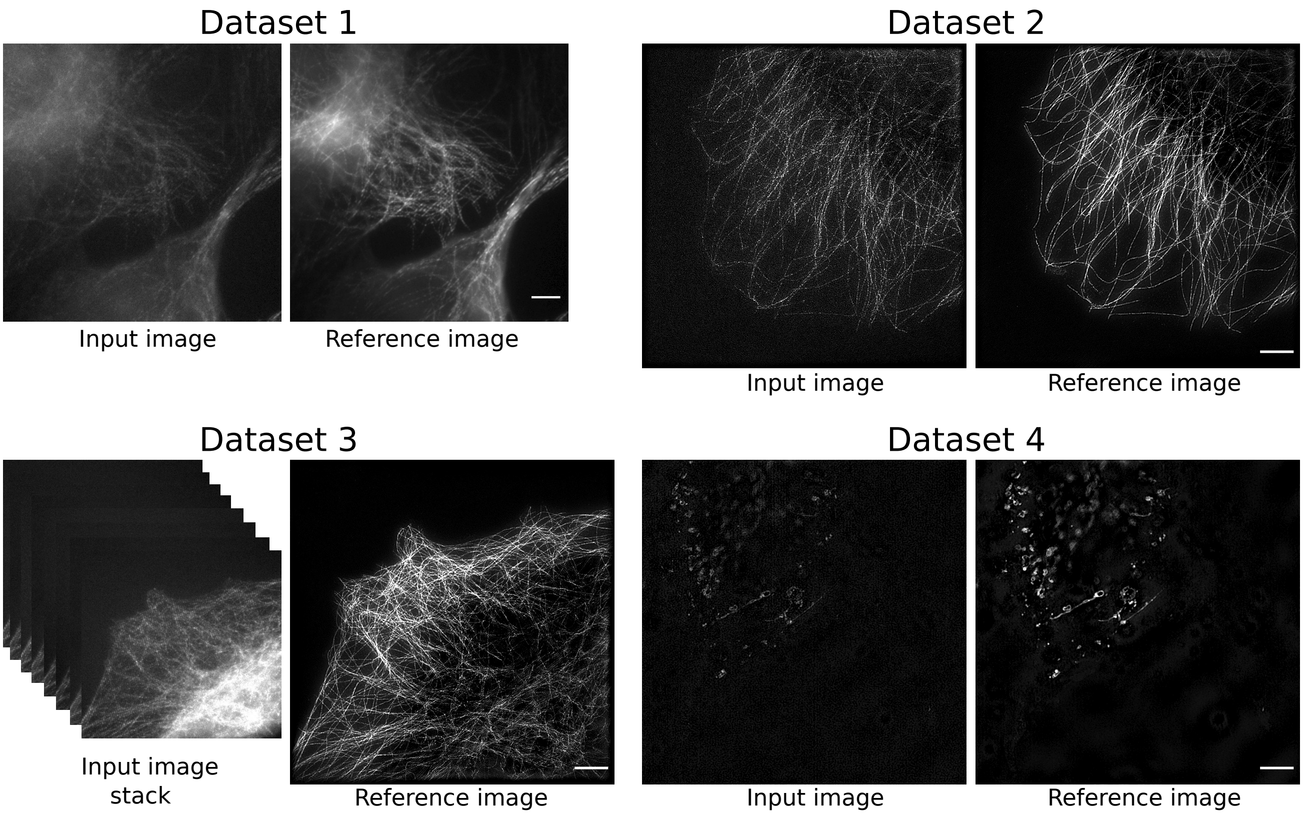

Figure 2. Dataset 1 consists of raw SIM data. The input-reference pairs represent each phase and orientation from different noise levels of tubulin filaments. The size of the input and reference images is 512 × 512 pixels in dataset 1. Scale bar: 4 µm. Dataset 2 contains the reconstructed SR-SIM images of tubulin structure, and each input-reference pair has a size of 1024 × 1024 pixels. Scale bar: 4 µm. In dataset 3, raw SIM data in the form of an image stack (i.e., 15 × 512 × 512 pixels) are used as input samples, whereas the reconstructed SR-SIM images (1024 × 1024 pixels) are used as the corresponding reference samples for joint denoising and super-resolution. Scale bar of reference image: 4 µm. Dataset 4 is based on the reconstructed SR-SIM images of size 1024 × 1024 pixels of fluorescently labeled vesicles. Scale bar: 4 µm.

(a is a compensation parameter,see [7, 40]) is used. For the tubulin samples, a background of 500 counts per pixel is subtracted during the reconstruction process. SR-SIM reconstruction parameters (pattern orientation, global phase, etc.) are automatically determined by fairSIM's standard, iterative cross-correlation approach. Filter parameters are set to a generalized Wiener filter with a strength of $w = 0.05$, apodization is set at 1.9× the resolution limit with a *bend* of 0.8. A notch-style filter implemented as *OTF attenuation* with a strength of 0.995 and a FWHM of 1.2 µm^{-1} is used. The full information about the functionality of these parameters is explained in [7], and the general guide for using SIM reconstruction parameters is discussed in [40]. The code used to generate the samples of dataset 2 is available at [41]. The samples in dataset 4 (the vLamp1-mScarlet expressing cell) were reconstructed using the commerical software 'softWoRx v7' (GE Healthcare manufacturer's software) for 3D-SIM. Here, a full 3D volume of data is acquired by the microscope and passed through the reconstruction process as a 3D stack. While both single-slice reconstruction (fairSIM) and full 3D volume reconstruction offer the same increase in lateral resolution, a full 3D volume reconstruction additionally provides an axial resolution increase (when used on 3-beam data as provided by the Delta Vision OMX and other 3-beam SIM systems). This of course comes at the expense of requiring full 3D volumes of data to be aquired, largely increasing imaging time and photo-toxicity. Thus, depending on the imaging needs, either single-slice data acquisition (and reconstruction) or full 3D volumes are chosen. For an overview of all datasets, see Table 1.

Description of datasets

Dataset 1

Dataset 1 contains around 101 fields-of-view (FOV) of tubulin filaments and each FOV further consists of 3000 images (all in one TIF file for each FOV). In each FOV, a stack of 15 raw SIM images represents the combination of 5 orientations and 3 phases, whereas this full stack is repeatedly captured for 200 timestamps. The signal-to-noise ratio decreases with every timestamp. Thus, dataset 1 contains a total of 303000 raw SIM images of size 512 × 512 (width ×height) pixels with 15 combinations of phase and orientation at each timestamp. Each timestamp in the raw images lasts approximately 25 ms. Each pixel contains a single 16-bit integer value captured by the microscope's camera, which is calibrated to provide a signal linear in photon count for each pixel. This is typical for scientific camera systems, but dissimilar to standard image processing, where often gamma mapping is applied between light intensity and pixel values. This dataset can be used mainly for image denoising tasks (from noisy input to output with higher signal-to-noise ratio). Therefore, the images from timestamp 1 are intended as output images (reference/ground truth), and the rest of the images can be categorized as input images as shown in Figure 2.

Dataset 2

We constructed dataset 2 by applying the fairSIM reconstruction algorithm to the raw SIM images from dataset 1. Dataset 2 contains therefore pairs of reconstructed high-resolution SR-SIM images, each pair consisting of the noisy input and the reference output. The process of the generation of input and

Table 1. Description of all the datasets

Dataset	Dataset 1	Dataset 2	Dataset 3	Dataset 4
Structure	tubulin filaments	tubulin filaments	tubulin filaments	vesicles
Microscope	SR-SIM microscopy	SR-SIM microscopy	SR-SIM microscopy	SR-SIM microscopy
Pixel Size, nm	80	40	80 (input) / 40 (ref.)	40
Number of timestamps	200	200	200	max: 99, min: 15
Fields-of-view	101	101	101	175
Input image size, pixels	512x512	1024x1024	15x512x512	1024x1024
Output/Reference image size, pixels	512x512	1024x1024	1024x1024	1024x1024
No of samples	303000	2525	2525	7284
Reconstruction	raw data	fairSIM	fairSIM	softWoRx

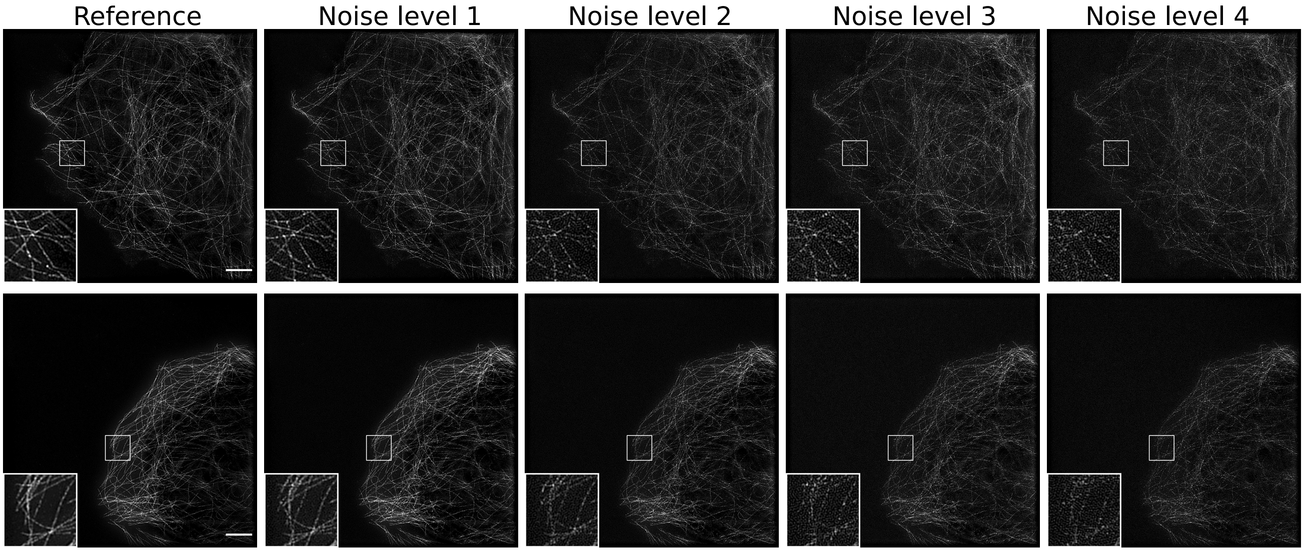

Figure 3. SR-SIM images from two different FOV, each shown at several noise levels. Each noise level corresponds to a different range of timestamps, e.g., images of noise level 4 are taken at the last timestamps and contain a high level of noise. Similarly, images of noise level 1 represent early timestamps with very low noise. The reference image is recorded at timestamp 0 and has the highest signal-to-noise ratio. Scale bar: 4 µm.

output SIM images from the raw SIM images is shown in Figure 1. The 15 raw SIM images of size 512 × 512 (width × height) pixels of different phases and orientations are propagated into the fairSIM algorithm to reconstruct the SR-SIM images of size 1024 × 1024 (width × height) pixels. During the formation of this dataset, the raw samples from timestamp 1 were used to generate the output images (i.e., reference images). We use the term 'reference images' instead of 'ground truth images' because of the SIM reconstruction artifacts in the output images of this dataset. The input samples were reconstructed by using the raw SIM images from timestamps 176 – 200. Therefore, dataset 2 is composed of 2525 reconstructed pairs of SR-SIM images with a size of 1024 × 1024 (width × height) pixels extracted from the 101 FOV. In addition to these 2525 image pairs, for the last 20 FOV we also include image pairs in the data collection where the noisy input is from timestamps 76 – 100, 126 – 150 and 176 – 200. This additional data can be used to create test sets to evaluate the robustness of denoising networks for different noise levels. In our previous work [19], we denoted data from timestamp 26 – 50 as noise level 1. Similarly, noise level 2, 3, and 4 correspond to the data from the timestamps 76 – 100, 126 – 150 and 176 – 200 (shown in Figure 3). Overall, dataset 2 is generated mainly for the denoising of SR-SIM images.

Dataset 3

Dataset 3 was created mainly for joint denoising and super-resolution tasks. The composition of this dataset is based on the stack of raw noisy input SIM images obtained from Dataset 1 of noise level 4 and the reference SR-SIM images from Dataset 2 of timestamp 0. The raw noisy SIM images in the input stack represents different illumination phases and orientations. The reference samples are the reconstructed high-resolution SR-SIM images. The size and dimension of each input sample are 15 × 512 × 512 (depth × width × height) pixels. The size of the output or reference sample is 1024 × 1024 (width × height) pixels. The input and reference samples in this dataset contain tubulin filaments as biological structures.

Dataset 4

Dataset 4 is composed of 3D-SR-SIM images showing vesicles in U2OS cells (i.e. typically round intracellular droplets) and can be used mainly for denoising tasks. Full 3D SIM raw data stacks have been passed through a 3D-enabled SIM reconstruction, extending both lateral and axial resolution of the data [15]. This data is composed of nine z-stacks, each with a varying number of depth levels (z-planes), and each captured for a different number of timestamps. In our work, we focus on the denoising along the lateral plane, therefore each z-stack is split into its single z-planes (also called "slices"). The recorded structures in the different z-planes are very different from each other, so we categorize them here as separate FOV.[1]

As result, the raw data of this dataset contains 175 FOV, and each FOV is recorded for a different number of timestamps. This yields a total of 7284 pairs of input and output

1 The file names in the dataset contain also indices for the original z-stack.

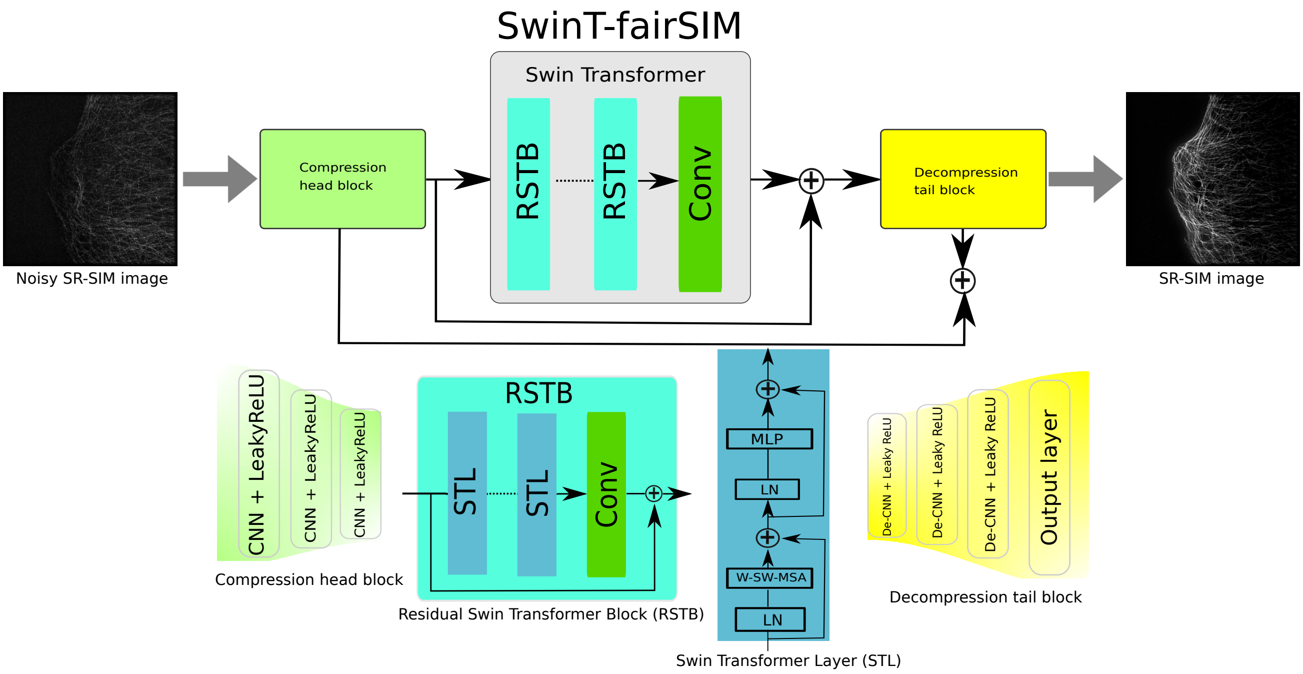

Figure 4. The architecture of the SwinT-fairSIM denoising method. A more detailed view into each block of the pipeline is shown in the lower part of the figure.

SR-SIM images. The size of the input and reference images is 1024×1024 (width × height) pixels. In each image pair, the output/reference image is taken from timestamp 1 of the respective FOV, whereas the input images are taken from the following time stamps and exhibit therefore a lower signal-to-noise ratio. The rational for splitting 3D data into separate 2D slices for denoising and artifact removal is as follows: The structure of the 3D SIM reconstruction algorithm (shifting and recombining lateral but not axial frequencies) makes it very likely that noise and reconstruction artifacts arise in the lateral directions of the acquired images, not so much in the axial component. Limiting the post-processing to 2D increases speed and reduces training effort significantly. Also, the height of a 3D stack (number of slices) varies from sample to sample, while the lateral FOV is typically constant. Of course, dataset 4 would allow full 3D implementations to be tested, by recombining the z-sliced data into 3D volumes. The reconstruction of the original raw SIM images into the SR-SIM images in this dataset was performed by using the softWoRx v7 software (GE Healthcare manufacturer's software).

Data partitioning

In our experiments, in the first three datasets, the images of the last 20 FOV were used as test samples, and the remaining 81 FOV were used for the training set. The training set of dataset 2 is therefore composed of 2025 samples (image pairs) from 81 FOV and the test set is composed of 500 samples from 20 FOV. Similarly, in dataset 4 we used 5562 samples from 121 FOV for the training set and 1380 samples from 46 FOV for the test set; the remaining 8 FOV were discarded because their reference images contain only noise without any meaningful structure. Datasets 1, 2, and 4 can only be used for denoising tasks, however, the data from dataset 3 can be used for both joint denoising and super-resolution. To reproduce the results of this work, we share the source code on the GitHub repository [42], the trained models [43] and the data on GigaDB ["link will be uploaded after the allocation of space from Giga Database"].

Shifted window Transformer for the denoising of SR-SIM images (SwinT-fairSIM)

The architecture of SwinT-fairSIM is composed of three main components: Compression head, Swin Transformer, and decompression tail block as shown in Figure 4. The architecture of SwinT-fairSIM is an extended version of the SwinIR architecture [36]. The compression head block is responsible for shallow feature extraction and the downsizing of the input images, the Swin Transformer block is based on encoder-decoder components to recover missing information and for deep feature extraction, and the decompression tail component is used for upsampling and restoring the features in the resultant images. The compression head block is composed of three convolution layers to compress the input image features to a size of 256×256 (width × height) pixels by using a stride of size 2. The encoder-decoder Swin Transformer block is made up of several Residual Swin Transformer blocks (RSTB), and each RSTB is based on few Swin Transformer layers (STL) and convolutional layers [36]. The STL is further composed of two window (W) and shifted window (SW) based multi-head self-attention (MSA) modules, followed by a multi-layer perceptron that has two fully-connected layers with GELU non-linearity [25, 35]. The shifted window partitioning strategy introduces connections between non-overlapping patches in the preceding layer and is found to be effective in a variety of computer vision tasks [36]. The Swin Transformer layer first reshapes the input (X) of size $H \times W \times C$ (height × width × channels) into $\frac{HW}{M^2} \times M^2 \times C$, where the $\frac{HW}{M^2}$ is the total number of windows or patches. Two additive based residual skip connections are also used in each STL unit. The query (Q), key (K), and value (V) matrices are then calculated for each window separately to obtain self-attention.

$$Q = XP_Q,\ K = XP_K,\ V = XP_V \tag{1}$$

In equation (1), P_Q, P_K, and P_V are the shared projection matrices across all the windows. The attention matrix is then computed by the self-attention mechanism in a local window as

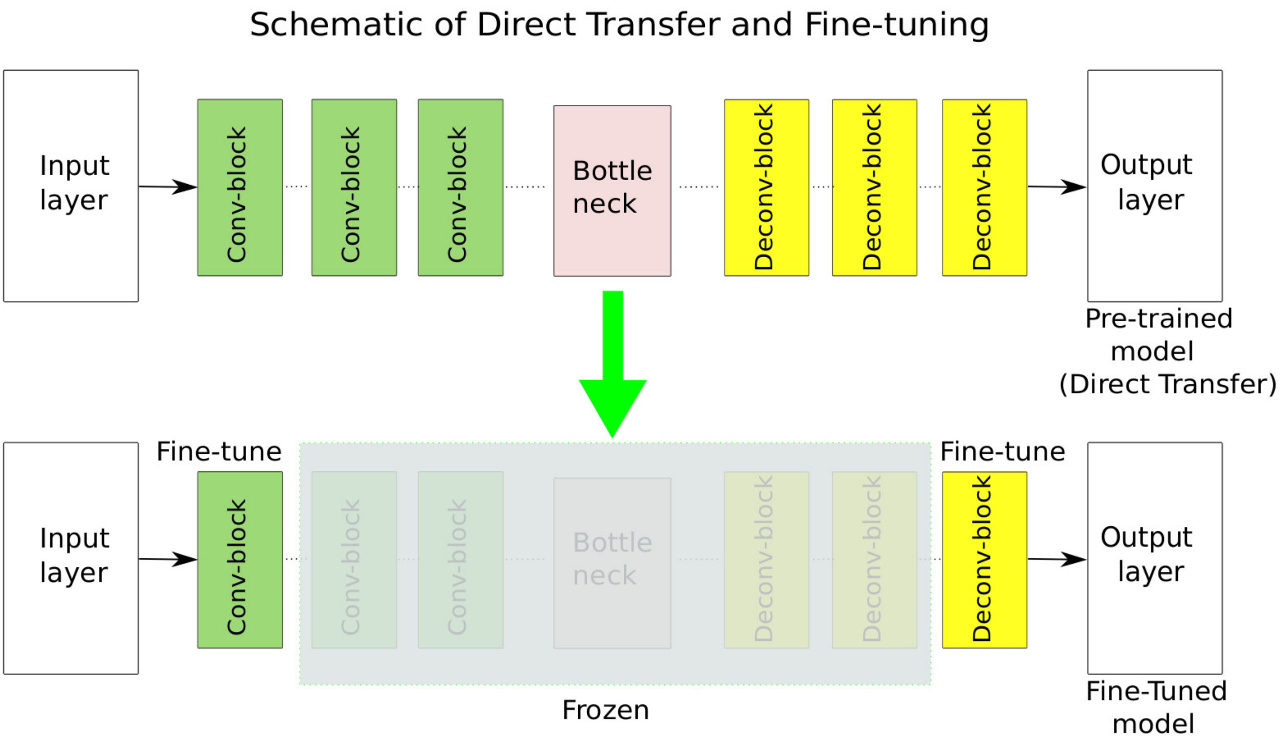

Figure 5. Schematic diagram of direct transfer and fine-tuning. The first pipeline shows the pre-trained model. The highlighted region in the second pipeline shows the frozen part; all parts of the model that are not frozen are retrained in the fine-tuning strategy.

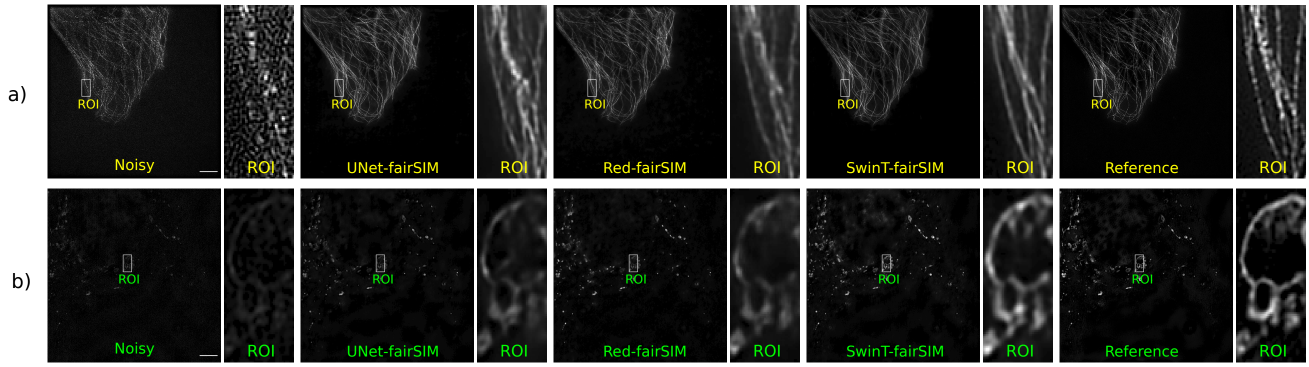

Figure 6. Results for test samples from datasets 2 and 4 are shown in this figure in the first row **(a)** and second row **(b)**. The outputs of UNet-fairSIM, Red-fairSIM, and SwinT-fairSIM are shown next to each other. The second, fourth, sixth, eighth, and tenth column depict the cropped and zoomed regions of interests (ROIs) from the full-size SR-SIM images (1024 $\times$ 1024 pixels). The cropped ROIs of size 50 $\times$ 100 pixels were upsampled to 150 $\times$ 300 pixels for demonstration purposes. Scale bar: 4 µm.

follows:

$$\text{Attention}(Q, K, V) = \text{SoftMax}(QK^T/\sqrt{d} + B)V \quad (2)$$

where B in equation (2) is the learnable relative positional encoding and d is the dimension of query and key features. The consecutive Swin Transformer layers are defined as:

$$\hat{X}^l = \text{W-MSA}(\text{LN}(X^{l-1})) + X^{l-1} \quad (3)$$
$$X^l = \text{MLP}(\text{LN}(\hat{X}^l)) + \hat{X}^l \quad (4)$$
$$\hat{X}^{l+1} = \text{SW-MSA}(\text{LN}(X^l)) + X^l \quad (5)$$
$$X^{l+1} = \text{MLP}(\text{LN}(\hat{X}^{l+1})) + \hat{X}^{l+1} \quad (6)$$

In equations (3)-(6), $\hat{X}^l$ and X^l denote the output features of the (S)W-MSA and MLP modules for layer l, where LN represents the LayerNorm operation.

Finally, the decompression tail block accommodates 3 transposed convolutional layers along with the output convolutional layer to transform and upsample the feature maps into the final resultant images. The necessity of head and tail block is to scale down and upsample the SR-SIM images with a size of 1024 × 1024 (height × width) pixels. In the SwinT-fairSIM method, we set the RSBT, STL, window size, and the attention head numbers to 5, 5, 8, and 120 respectively. The adjacent layers in the head and tail blocks are connected via additive skip connection and contain the same number of kernels. These blocks differentiate our architecture from the existing SwinIR architecture [36]. These blocks reduce the computational effort in the central Transformer part of the architecture and therefore adapt the Swin Transformer block to images with a high pixel count.

The name "SwinT-fairSIM" was chosen to be consistent with the names of our previously proposed CNN-based denoising methods for SR-SIM data, "Red-fairSIM" and "UNet-fairSIM" [16]. Red-fairSIM and UNet-fairSIM are described in detail in [16]. These algorithms originally worked only in conjunction with the fairSIM software [7] for SR-SIM reconstruction. In contrast, in this paper, we also use the softWoRx implementation of the SIM algorithm for dataset 4. However, to be consistent with the naming scheme in [16], we keep the suffix "fairSIM" in all of our deep learning based approaches.

Transfer learning and fine-tuning

In transfer learning in general, the knowledge of a trained model from a related task that has been learned is transferred to another task from the same domain [44]. The parameters of the pre-trained model, trained on specific data and for a specific task, are transferred to the different data but related task [45, 46]. The use of transfer learning in deep learning is very useful to reduce the computational demands and time complexity [47]. In addition, transfer learning is also very helpful when it comes to alleviating large dataset requirements [48].

The simplest form of transfer learning is to apply a trained model directly to another task without retraining. This direct use of a pre-trained model on new test data is called "direct transfer". Beyond this most basic approach, fine-tuning is one of the most important strategies for transferring model knowledge from one domain to another [49]. During fine-tuning, the weights of some layers of pre-trained models are preserved and the rest of the layers are retrained (i.e., "fine-tuned") [50]. The concepts of direct transfer and fine-tuning are illustrated in Figure 5. It can be seen from the schematic in Figure 5 that the weights of the intermediate layers of the pre-trained models are frozen while the rest of the layers are retrained in the fine-tuning strategy, whereas no retraining is performed in the direct transfer approach. For our work here, in the direct transfer strategy, the models are trained from scratch on dataset 2, and then the trained model is evaluated with test samples from dataset 4. Similarly, the models trained on dataset 4 are evaluated on the test images from dataset 2. In the fine-tuning strategy, models are trained from scratch on dataset 2, afterwards they are partly retrained on dataset 4 and also tested on dataset 4. Or the other way round: Complete initial training on dataset 4, afterwards fine-tuning on dataset 2 and finally evaluation on dataset 2. In this work, we performed these different training strategies with the SwinT-fairSIM, Red-fairSIM, and UNet-fairSIM algorithms [19] with datasets 2 and 4.

To perform fine-tuning on the SwinT-fairSIM algorithm, we retrained the head and tail blocks along with one adjacent RSTB block each. By doing so, about 1.9 million training parameters stayed frozen out of 4 million parameters. Regarding the CNN-based algorithms from our previous work [19], we used the following approaches to fine-tuning: Red-fairSIM is based on the residual-encoder-decoder network (RED-Net) [51]. In the fine-tuning approach for Red-fairSIM, we retrained the first and last 5 layers of the model instead of all 30 layers. By doing so, about 700K training parameters stayed frozen out of more than 1 million parameters. UNet-fairSIM is based on the UNet architecture which is based on several encoder and decoder blocks [52]. In the fine-tuning of UNet-fairSIM, we simply retrained the first and last two encoder and decoder blocks; in doing so, more than 29 million parameters out of 33 million parameters stayed frozen.

Generally, in the first step, all these algorithms were trained from scratch on datasets 2 and 4 separately for 100 epochs. The mean squared error (MSE) was used as a loss function for all training runs with the Adam optimizer. The learning rate was set to 1×10^{-4} for all training runs. In the next step, we applied direct transfer by propagating the test samples from the respective dataset not used for training the pre-trained models. In direct transfer, the test images of the dataset 2 (i.e., tubulin filaments) were propagated through the pre-trained model trained on the dataset 4 (i.e., vesicle structure) and vice versa. In the third step, the pre-trained models were fine-tuned using training data from the respective dataset not used for initial training. 30 training epochs were used for fine-tuning. The fine-tuned models were then evaluated on the test samples from the dataset used in the fine-tuning process. When fine-tuning the pre-trained models, we initially kept different numbers of layers/blocks frozen. However, we found that the best results were obtained by fine-tuning rather few trainable parameters for 30 epochs (except for SwinT-fairSIM, where a larger part of the network had to be "unfrozen"). In the final step, we compared the results of conventional training with the direct transfer and fine-tuning strategies visually, as well as in terms of peak signal-to-noise ratio (PSNR) [53] and structural similarity index measurement (SSIM) [54] values.

Results

We collected four data sets for denoising and super-resolution tasks. The overview and full characteristics of these datasets are shown in Table 1. The images in dataset 2 contain the tubulin structure along with mixed Poisson-Gaussian (MPG) noise and SR-SIM reconstruction artifacts. The images in dataset 4 contain the vesicle structure along with MPG noise and honeycomb pattern artifacts that arise when raw data carrying predominantly Poisson noise is subjected to the frequency-based SR-SIM reconstruction algorithm, which then introduces re-

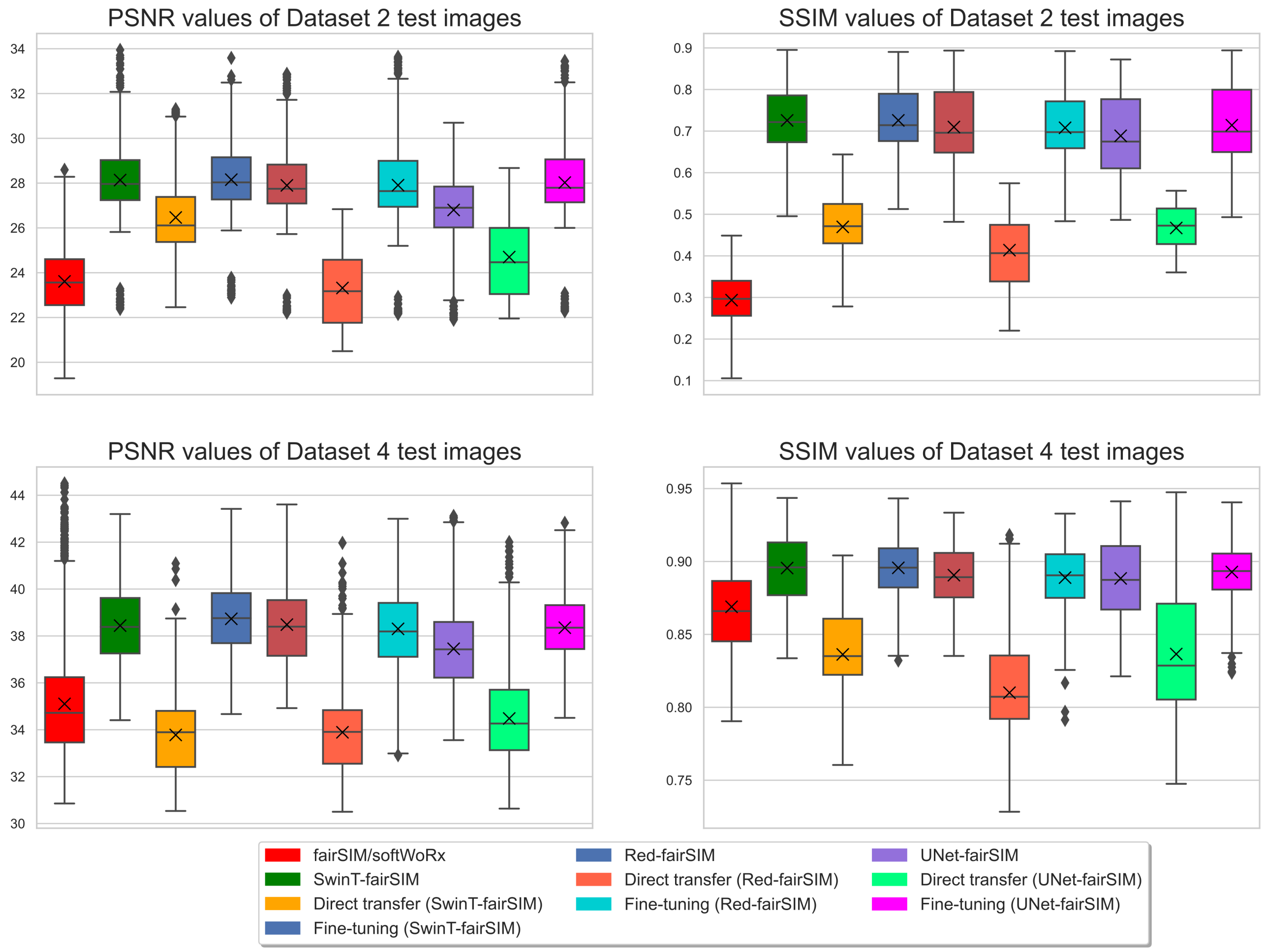

Figure 7. Boxplots of PSNR and SSIM values of test samples of dataset 2 and dataset 4 with different denoising algorithms and training strategies. The test data contains 500 images from noise level 4 for dataset 2 and 1380 images for dataset 4. The horizontal line inside each box plot represent the median value, whereas, the black cross display the mean values. The diamond-shaped black markers display the outlier observations. The color of the boxes in each subplot refers to the methods defined in the legend box.

construction artifacts [14]. In our previous studies [19], we already used the first three datasets for the denoising and super-resolution tasks. Here, we mainly focus on the denoising of SR-SIM images by SwinTransfomer-fairSIM and compare the results of this new method with the results of Red-fairSIM and UNet-fairSIM [19]. We also analyze two transfer learning strategies, direct transfer and fine-tuning of the pre-trained models of these network architectures.

To compare the performance of conventional training of these deep learning-based denoising methods with direct transfer and fine-tuning, we first train the SwinT-fairSIM, Red-fairSIM, and UNet-fairSIM networks separately with datasets 2 and 4 for 100 epochs. The results of all methods trained separately with both datasets are shown visually in Figure 6 and quantitatively in Table 2. The visual and quantitative results of SwinT-fairSIM show superiority over the Red-fairSIM and UNet-fairSIM methods. The resulting ROIs of both datasets from SwinT-fairSIM are more appealing and sharper than those of the other methods in Figure 6. The PSNR and SSIM values of SwinT-fairSIM are also slightly higher than those of Red-fairSIM and UNet-fairSIM. Overall, SwinT-fairSIM outperforms its counterparts on both datasets 2 and 4 after conventional training from scratch.

In the next step, we evaluate the direct transfer strategy by using these pre-trained models. In direct transfer, the model initially trained with dataset 2 is tested with the test samples of an alternative dataset (i.e., dataset 4), and vice versa. Similarly, in the assessment of the fine-tuning strategy, we re-trained the first and last layers of the pre-trained models with the alternative data to see the improvement in the generalization power of the pre-trained models. The resulting denoised images obtained by direct transfer of models trained on vesicle structures (dataset 4) to test images containing tubulin filaments (dataset 2) are displayed within block **(a)** of Figures 8 and 9. These images clearly show that the model trained on a specific type of noise and structure (i.e., vesicle images with honey comb pattern noise) is not able to produce a refined denoised image of another structure (i.e., tubulin filaments with MPG noise). Similarly, the models initially trained on tubulin filaments from dataset 2 are not able to properly produce the denoised images of dataset 4 with different structure and noise (see block **(b)** of Figures 8 and 9). However, it can be noticed that the pre-trained models try to replicate the filamentous structure of tubulin in the vesicle data (very prominent in block **(b)** of Figure 8). This is a clear indication that these deep learning based denoising models are not robust against different types of noise and structure. However, the outcomes of the fine-tuning approach are promising as shown in Figures 8 and 9. The ROIs 1 and 2 in column 6 of Figure 8 and also the ROIs in column 4 of Figure 9 show that the results of the fine-tuning strategy are very close to the results of training from scratch for the SwinT-fairSIM, Red-fairSIM and UNet-fairSIM

Table 2. Mean PSNR and SSIM values along with standard deviations (STD) of all experiments calculated on the noisy test images of datasets 2 and 4 (for dataset 2, noise level 4 was used). The test data contains 500 images for dataset 2 and 1380 images for dataset 4. The PSNR and SSIM values are calculated relative to the reference images, i.e. SR-SIM images reconstructed with fairSIM/softWoRx from raw SIM images with the highest signal-to-noise ratio. In the row entitled “fairSIM/softWoRx”, PSNR and SSIM values are calculated for the direct fairSIM/softWoRx reconstruction of the noisy images. The rows entitled “SwinT-fairSIM”,“Red-fairSIM” and “UNet-fairSIM” show the results for the denoised test images after conventional training from scratch with the respective algorithms. In the direct transfer rows, the mean PSNR and SSIM values in the column “dataset 2” are calculated for models which are initially trained on dataset 4 and afterwards tested with the test samples from dataset 2. For the column “dataset 4”, this is the other way round. The same principle holds for the rows for fine-tuning, only that the models are fine-tuned on the respective dataset before testing.

	Mean PSNR (STD) and SSIM (STD) values of test data			
	**dataset =2**		**dataset =4**	
	PSNR (STD)	SSIM (STD)	PSNR (STD)	SSIM (STD)
fairSIM/softWoRx	23.61 (1.54)	0.29 (0.07)	35.10 (2.71)	0.86 (0.03)
SwinT-fairSIM	28.19 (2.09)	0.72 (0.09)	38.44 (1.73)	0.89 (0.02)
Direct transfer (SwinT-fairSIM)	26.07 (1.91)	0.48 (0.07)	34.05 (2.24)	0.83 (0.03)
Fine-tuning (SwinT-fairSIM)	28.15 (1.87)	0.72 (0.08)	38.52 (1.50)	0.89 (0.02)
Red-fairSIM	27.97 (2.01)	0.71 (0.09)	38.43 (1.45)	0.89 (0.01)
Direct transfer (Red-fairSIM)	23.31 (1.68)	0.41 (0.07)	33.89 (1.93)	0.81 (0.03)
Fine-tuning (Red-fairSIM)	27.90 (2.14)	0.70 (0.09)	38.30 (1.60)	0.88 (0.02)
UNet-fairSIM	26.80 (1.65)	0.68 (0.10)	37.45 (1.79)	0.88 (0.02)
Direct transfer (UNet-fairSIM)	24.69 (1.54)	0.46 (0.05)	34.47 (2.24)	0.83 (0.04)
Fine-tuning (UNet-fairSIM)	28.02 (1.97)	0.71 (0.07)	38.35 (1.44)	0.89 (0.01)

algorithms. The overall comparison of all the approaches in Figures 8 and 9 demonstrates that the fine-tuning method is crucial for the Transformer and CNN-based denoising algorithms in the case of changes in structure or noise types. Thus, fine-tuning is inevitably required to profit from knowledge transfer.

Table 2 lists the average PSNR and SSIM values of the test images of the datasets 2 and 4 calculated for all the training and testing strategies of SwinT-fairSIM, Red-fairSIM, and UNet-fairSIM. Table 2 clearly points out the decline in the average PSNR and SSIM values on both datasets 2 and 4 after the application of direct transfer. However, the average PSNR and SSIM values show considerable improvement after fine-tuning. The average PSNR and SSIM values of the fine-tuned models in Table 2 are very close to the models that were trained from scratch (partly slightly better, partly slightly worse). In addition, Figure 7 shows boxplots of the PSNR and SSIM values of the test samples of both datasets with all different methods/strategies. The boxplots complement the mean values in Table 2 by depicting additional statistics. It can be observed that SwinT-fairSIM not only has often higher mean and median values, but also a more consistent interquartile range in all of the boxplots in Figure 7. In the SSIM boxplots of dataset 2, very few outliers are noticable overall.

Compared to conventional training, the fine-tuning strategy retrains only a subset of the entire set of model parameters. In this study, we retrained about 2.1 million parameters out of 4 million parameters of SwinT-fairSIM, while for Red-fairSIM and UNet-fairSIM, out of more than 1 million and 33 million training parameters, only 295K and 3.7 million parameters were retrained, respectively. We were able to achieve results comparable to those of conventional training after only 30 epochs as opposed to 100 epochs. Training times for dataset 2 on two Nvida V100 GPUs (32 GB) were (training from scratch vs. fine-tuning):

SwinT-fairSIM: 26 h vs. 3.25 h
Red-fairSIM: 24 h vs. 3 h
UNet-fairSIM: 18 h vs. 1.5 h

Therefore, in terms of computational requirements or memory consumption, it is advantageous to use fine-tuning instead of training from scratch. Fine-tuning is beneficial when the computational resources are limited.

Discussion

In summary, the contribution of this work is threefold: First, we publish novel datasets related to SIM microscopy, then we explore a Transformer-based algorithm for the restoration of SR-SIM images. Finally, we investigate the potential of the direct transfer and fine-tuning strategies for various deep learning-based denoising algorithms. Regarding datasets, we provide four novel datasets for testing denoising and super-resolution image reconstruction strategies with tubulin filaments and vesicle structures. These datasets contain a large number of raw SIM images and reconstructed twofold super-resolved SIM images and cover different noise levels, a wide range of fields of view, and varying degrees of structural complexity. High-quality data from the real world of microscopy are highly relevant for benchmarking and evaluating current and upcoming denoising and super-resolution methods.

Regarding our work with Transformers, we suggested the SwinT-fairSIM architecture to produce high-quality SR-SIM images from low SNR inputs. Importantly, we visually and quantitatively showed that the Transformer-based method can achieve mostly better results than the CNN-based Red-fairSIM and UNet-fairSIM methods (i.e., for the quantitative results, PSNR and SSIM values, see Table 2 and the boxplots in Figure 7). We demonstrated that SwinT-fairSIM can retrieve more well-preserved cell structures and texture information than the CNN-based methods, especially in the ROIs of Figure 6. This is an important finding, since most existing SR-SIM restoration

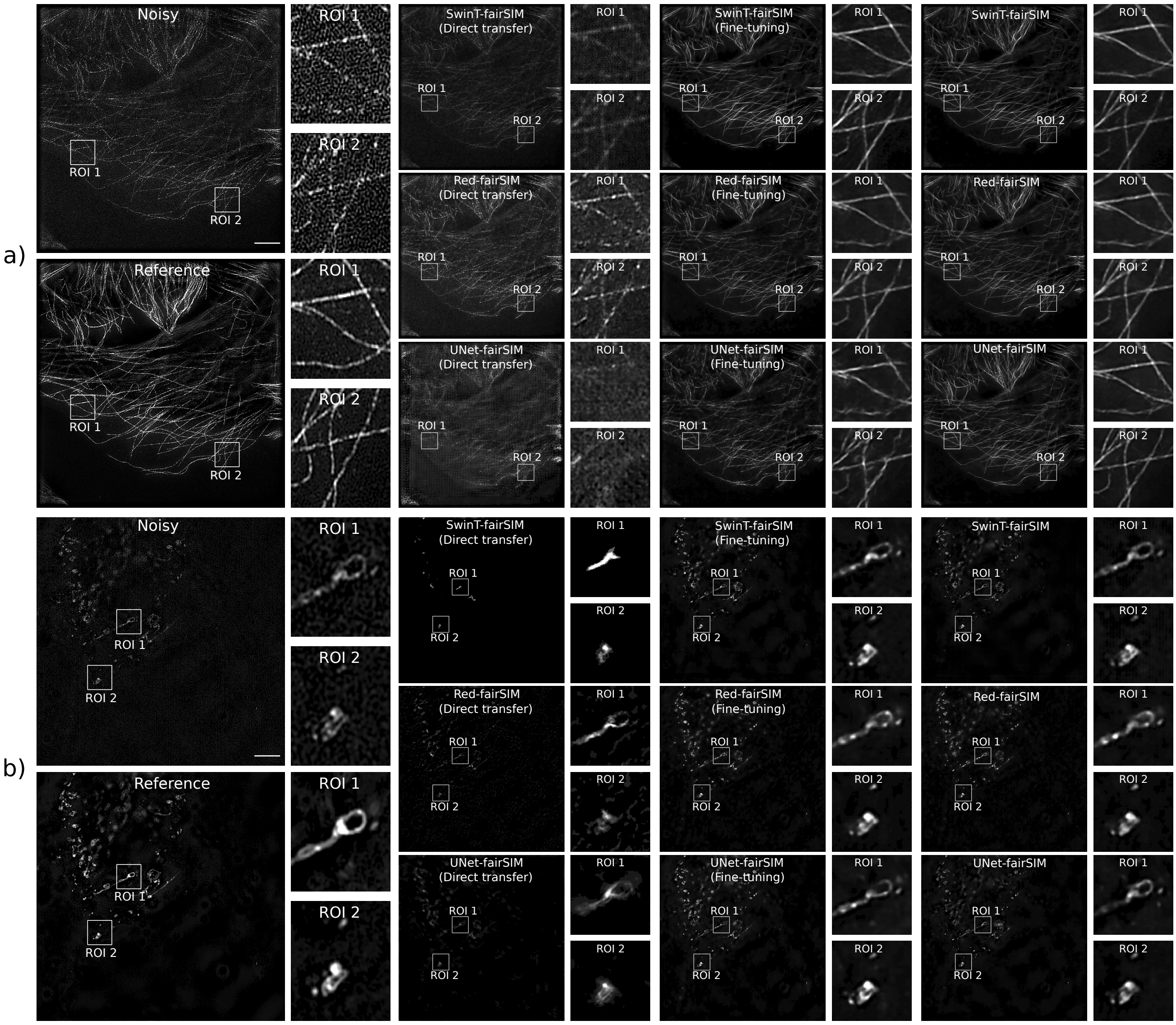

Figure 8. Blocks (**a**) and (**b**) show the results for test samples from dataset 2 (block **a**) and dataset 4 (block **b**) for different algorithms and learning strategies. The first and second columns of blocks **(a)** and **(b)** show the noisy and reference SR-SIM images, respectively, along with extracted and magnified regions of interest (ROIs). The noisy images are the test input for all algorithms. Columns 3 to 6 show the results of direct transfer and fine-tuning with SwinT-fairSIM, Red-fairSIM, and UNet-fairSIM. In direct transfer, the model is trained with dataset 2 and tested with the test sample of dataset 4 and vice versa. Similarly, in fine-tuning, the model is first pre-trained on dataset 2 and then fine-tuned and tested on dataset 4 and vice versa. Columns 7 and 8 show the results of all algorithms when trained from scratch on the respective dataset, which is also used for testing. The fourth, sixth, and eighth columns show the cropped and enlarged ROIs from the full-size SR-SIM images to the left of each ROI. The ROIs of size 100 pixels $\times$ 100 pixels have been upsampled to 300 pixels $\times$ 300 pixels for illustration purposes. Scale bar: 4 µm.

methods are based on CNNs [17, 18, 19, 21, 22, 23, 38, 39]. An explanation for the better performance of SwinT-fairSIM may be the size of the receptive fields of the different network architectures. In CNNs, the size of their receptive fields is limited by the size and stride of the filter kernels and the depth of the network. In contrast, Swin Transformers may obtain a larger receptive field by the shifted windows operation [55, 56].

In the last section of this work, we showed the limitations of Transformer- and CNN-based deep learning methods in the area of knowledge transfer when it comes to different types of noise and structure. In the direct transfer strategy, we simply evaluated the pre-trained networks with new test data. The direct transfer strategy exhibits very downgraded visual (in Figures 8 and 9) and quantitative (in Table 2 and Figure 7) results. However, the approach of fine-tuning enables these deep learning models to generalize well to other noise types and structures after retraining few layers. This holds for all learning algorithms and datasets in a similar way as shown in Figures 7, 8, 9 and in Table 2. All of the deep neural network architectures tested here are, at their core, encoder-decoder architectures (for SwinT-fairSIM, this applies at least to the compression head block and the decompression tail block). In these architectures, the initial and final layers are responsible for handling low-level features. Noise patterns can be interpreted as low-level features that are separated from the valuable input during the encoding process so that they can be selectively suppressed at a later stage during decoding. Accordingly, we retrained only the initial and final layers of all three types of neural networks to achieve results on par with training from scratch. It is worth mentioning that we also attempted to retrain only the first, middle, or last layers during first exploratory research, and also varied the number of retrained layers, but the results obtained in this way were not competitive. Since fine-tuning often saves computation time and often requires fewer learning samples, we conclude that fine-tuning of pre-trained models has advantages over conventional train-

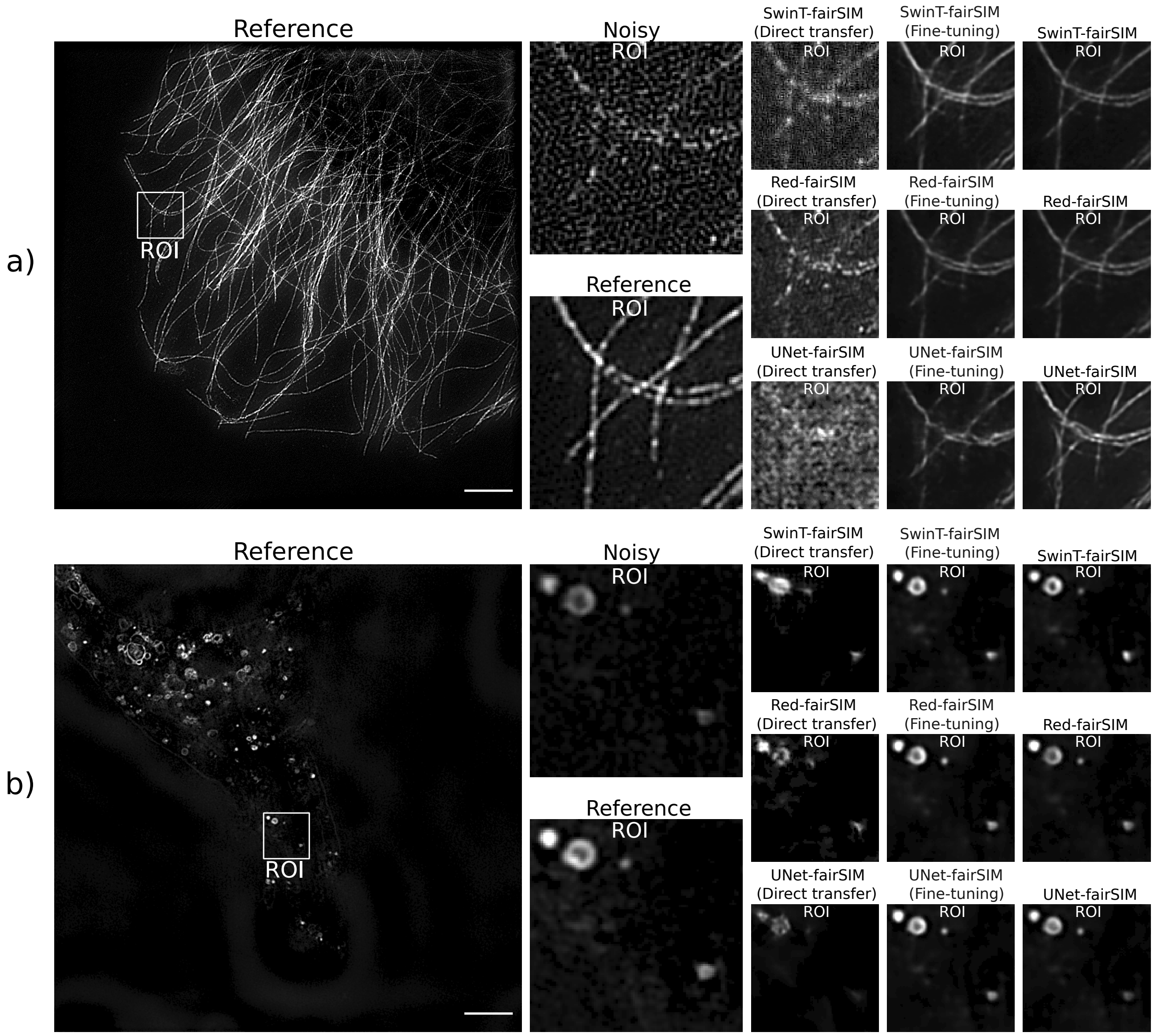

Figure 9. Two more test samples from datasets 2 and 4 are shown in this figure. Both blocks (**a**) and (**b**) contain the reference SR-SIM images along with the resultant denoised ROIs which are extracted from the full size denoised images of all the methods used in this work. The extracted ROIs are upsampled from 100 pixels $\times$ 100 pixels to 300 pixels $\times$ 300 pixels for illustration purposes. Scale bar: 4 µm.

ing from scratch, at least when the difference between the task domains is not huge. In contrast, direct transfer obviously fails because the learned detectors for low-level features (noise patterns and typical simple visual features of the main cell structures) are too much tuned to the original training data.

There are still some limitations of this work. First, while our datasets are useful for various image restoration tasks and contain more samples than many of the datasets previously published in the field, they are limited to two structures, tubulin filaments and vesicle structures. Second, our results indicate the superiority of SwinT-fairSIM over Red-fairSIM and UNet-fairSIM. However, this improvement leads to higher computational costs. Third, fine-tuning strategies provide similar results to training from scratch at lower cost, but it is often difficult to find task-specific pre-trained networks. Finally, slice-by-slice denoising of 3D SIM images focuses exclusively on the lateral plane; despite this limitation, it was shown that the presented approach can still be very useful for denoising 3D SIM structures.

Conclusion

In this work, we first presented a series of datasets for SIM image restoration tasks such as denoising and super-resolution. Additionally, we proposed a new algorithm based on the Swin Transformer, "SwinT-fairSIM", for the denoising of SR-SIM images. We showed that this Transformer-based algorithm outperforms CNN-based denoising algorithms both visually and quantitatively. This suggests that Transformer-based denoising algorithms can play an important role in the field of SR-SIM microscopy. Similarly, we evaluated different knowledge transfer strategies such as direct transfer and fine-tuning for Transformer- and CNN-based denoising algorithms. For the direct transfer strategy, we noticed a decline in the performance of these algorithms. However, we were able to recover the declined performance by the fine-tuning strategy. This clearly indicates that the Transformer- and CNN-based denoising methods require retraining of some initial and final layers of the pre-trained models when applied to new biological structures and noise types. This retraining requires fewer epochs than the conventional training from scratch. This holds

also for the novel SwinT-fairSIM denoising algorithm which outperforms all CNN-based algorithm also in the fine-tuning regime.

Potential implications

The implementation of different deep learning methods requires a large quantity of images in order to train the underlying models. We believe that our published datasets will help the research community to develop new deep learning based methods and evaluate the existing methods by either training from scratch or by applying fine-tuning. These datasets can be especially used for image denoising and super-resolution tasks.

Data and code Availability

All the raw and reconstructed datasets which are discussed in this article are available on GigaDB ["link will be uploaded after the allocation of space from Giga Database"]. All the datasets are distributed under the Creative Commons CCO waiver, with a request for attribution. The CCO permits the unrestricted reuse, distribution and reproduction, provided the original work is properly cited. Similarly, all the codes which are used during this work are publicly available [42].

Declarations

Abbreviations

SIM: structured illumination microscopy; SR-SIM: super-resolution structured illumination microscopy; NA: numerical aperture; PSNR: peak-signal-to-noise ratio; SSIM: structural similarity index measurement; MSE: mean square error; SNR: signal-to-noise ratio; W2S: wide-field2SIM; fairSIM: free analysis and interactive reconstruction for structured illumination microscopy.

Competing Interests

The authors declare no competing interests.

Funding

This work was supported by the EFRE-NRW funding programme "Forschungsinfrastrukturen" (grant no. 34.EFRE-0300180) and partly conducted within the framework of the project “SAIL: SustAInable Lifecycle of Intelligent SocioTechnical Systems” (grant no. NW21-059B). SAIL is receiving funding from the programme “Netzwerke 2021”, an initiative of the Ministry of Culture and Science of the State of Northrhine Westphalia. T.-C.W. and T.H. were supported by funding from the European Union’s Horizon 2020 research and innovation program under the Marie Sklodowska-Curie Grant Agreements No. 642157, project “TOLLerant”, and No. 766181. project "DeLIVER". T.H. also acknowledges funding by the Deutsche Forschungsgemeinschaft (DFG, German Science Foundation)—project number 415832635.

Author’s Contributions

Z.H.S. carried out the preprocessing of datasets for the machine learning work, created the figures, and wrote the manuscript. D.T. conducted experiments to find the optimal hyperparameters for the CNN-based approaches. T.-C.W. recorded the raw SIM images of dataset 1. M.M. supported Z.H.S. in the reconstruction of data for datasets 2 and 3 with fairSIM. W.H. recorded and reconstructed the images of dataset 4. W.S. and T.H. supervised the research. W.S., T.H., M.M., and W.H. contributed to the editing of the manuscript. In addition, they discussed the experimental results together with Z.H.S. All authors discussed and agreed on the final manuscript.

Acknowledgements

The authors would like to thank Dr. Matthias Fricke from the Center for Applied Data Science (CfADS) at Bielefeld University of Applied Sciencesn and Arts for providing access to their GPU compute cluster. We would also like to thank Dr. Olaf Kaczmarek and Markus Klappenback for providing access to the GPU compute cluster at Bielefeld University.

References

1. Hirvonen LM, Wicker K, Mandula O, Heintzmann R. Structured illumination microscopy of a living cell. European Biophysics Journal 2009;38(6):807–812.
2. Hell SW, Sahl SJ, Bates M, Zhuang X, Heintzmann R, Booth MJ, et al. The 2015 super-resolution microscopy roadmap. Journal of Physics D: Applied Physics 2015;48(44):443001.
3. Heintzmann R, Huser T. Super-resolution structured illumination microscopy. Chemical reviews 2017;117(23):13890–13908.
4. Demmerle J, Innocent C, North AJ, Ball G, Müller M, Miron E, et al. Strategic and practical guidelines for successful structured illumination microscopy. Nature protocols 2017;12(5):988–1010.
5. Schermelleh L, Ferrand A, Huser T, Eggeling C, Sauer M, Biehlmaier O, et al. Super-resolution microscopy demystified. Nature cell biology 2019;21(1):72–84.
6. Gustafsson MG. Surpassing the lateral resolution limit by a factor of two using structured illumination microscopy. Journal of microscopy 2000;198(2):82–87.
7. Müller M, Mönkemöller V, Hennig S, Hübner W, Huser T. Open-source image reconstruction of super-resolution structured illumination microscopy data in ImageJ. Nature communications 2016;7(1):1–6.
8. Lal A, Shan C, Xi P. Structured illumination microscopy image reconstruction algorithm. IEEE Journal of Selected Topics in Quantum Electronics 2016;22(4):50–63.
9. Brown PT, Kruithoff R, Seedorf GJ, Shepherd DP. Multicolor structured illumination microscopy and quantitative control of polychromatic light with a digital micromirror device. Biomedical Optics Express 2021;12(6):3700–3716.
10. Ströhl F, Kaminski CF. Frontiers in structured illumination microscopy. Optica 2016;3(6):667–677.
11. Zheng X, Zhou J, Wang L, Wang M, Wu W, Chen J, et al. Current challenges and solutions of super-resolution structured illumination microscopy. APL Photonics 2021;6(2):020901.
12. Huang X, Fan J, Li L, Liu H, Wu R, Wu Y, et al. Fast, long-term, super-resolution imaging with Hessian structured illumination microscopy. Nature biotechnology 2018;36(5):451–459.
13. Hoffman DP, Betzig E. Tiled reconstruction improves structured illumination microscopy. BioRxiv 2020;.
14. Smith CS, Slotman JA, Schermelleh L, Chakrova N, Hari S, Vos Y, et al. Structured illumination microscopy with noise-controlled image reconstructions. Nature methods

2021;18(7):821–828.
15. Gustafsson MG, Shao L, Carlton PM, Wang CR, Golubovskaya IN, Cande WZ, et al. Three-dimensional resolution doubling in wide-field fluorescence microscopy by structured illumination. Biophysical journal 2008;94(12):4957–4970.
16. Shah ZH, Müller M, Hammer B, Huser T, Schenck W. Impact of different loss functions on denoising of microscopic images. In: 2022 International Joint Conference on Neural Networks (IJCNN) IEEE; 2022. p. 1–10.
17. Jin L, Liu B, Zhao F, Hahn S, Dong B, Song R, et al. Deep learning enables structured illumination microscopy with low light levels and enhanced speed. Nature communications 2020;11(1):1–7.
18. Chen X, Li B, Jiang S, Zhang T, Zhang X, Qin P, et al. Accelerated Phase Shifting for Structured Illumination Microscopy based on Deep Learning. IEEE Transactions on Computational Imaging 2021;7:700–712.
19. Shah ZH, Müller M, Wang TC, Scheidig PM, Schneider A, Schüttpelz M, et al. Deep-learning based denoising and reconstruction of super-resolution structured illumination microscopy images. Photonics Research 2021;9(5):B168–B181.
20. Belthangady C, Royer LA. Applications, promises, and pitfalls of deep learning for fluorescence image reconstruction. Nature methods 2019;16(12):1215–1225.
21. Qiao C, Li D, Guo Y, Liu C, Jiang T, Dai Q, et al. Evaluation and development of deep neural networks for image super-resolution in optical microscopy. Nature Methods 2021;18(2):194–202.
22. Xypakis E, Gosti G, Giordani T, Santagati R, Ruocco G, Leonetti M. Deep learning for blind structured illumination microscopy. Scientific Reports 2022;12(1):8623.
23. Liu T, Liu J, Li D, Tan S. Improving Reconstruction of Structured Illumination Microscopy Images Via Dual-Domain Learning. IEEE Journal of Selected Topics in Quantum Electronics 2023;.
24. Qiao C, Chen X, Zhang S, Li D, Guo Y, Dai Q, et al. 3D structured illumination microscopy via channel attention generative adversarial network. IEEE Journal of Selected Topics in Quantum Electronics 2021;27(4):1–11.
25. Vaswani A, Shazeer N, Parmar N, Uszkoreit J, Jones L, Gomez AN, et al. Attention is all you need. Advances in neural information processing systems 2017;30.
26. Sutskever I, Vinyals O, Le QV. Sequence to sequence learning with neural networks. Advances in neural information processing systems 2014;27.
27. Dehghani M, Gouws S, Vinyals O, Uszkoreit J, Kaiser L. Universal Transformers. In: International Conference on Learning Representations;. .
28. Vyas A, Katharopoulos A, Fleuret F. Fast transformers with clustered attention. Advances in Neural Information Processing Systems 2020;33:21665–21674.
29. Wang B, Shang L, Lioma C, Jiang X, Yang H, Liu Q, et al. On position embeddings in bert. In: International Conference on Learning Representations; 2021. .
30. Cheng X, Lin H, Wu X, Shen D, Yang F, Liu H, et al. Mltr: Multi-label classification with transformer. In: 2022 IEEE International Conference on Multimedia and Expo (ICME) IEEE; 2022. p. 1–6.
31. Carion N, Massa F, Synnaeve G, Usunier N, Kirillov A, Zagoruyko S. End-to-end object detection with transformers. In: Computer Vision–ECCV 2020: 16th European Conference, Glasgow, UK, August 23–28, 2020, Proceedings, Part I 16 Springer; 2020. p. 213–229.
32. Sun Z, Cao S, Yang Y, Kitani KM. Rethinking transformer-based set prediction for object detection. In: Proceedings of the IEEE/CVF international conference on computer vision; 2021. p. 3611–3620.
33. Chen H, Wang Y, Guo T, Xu C, Deng Y, Liu Z, et al. Pre-trained image processing transformer. In: Proceedings of the IEEE/CVF Conference on Computer Vision and Pattern Recognition; 2021. p. 12299–12310.
34. Ali AM, Benjdira B, Koubaa A, El-Shafai W, Khan Z, Boulila W. Vision transformers in image restoration: A survey. Sensors 2023;23(5):2385.
35. Liu Z, Lin Y, Cao Y, Hu H, Wei Y, Zhang Z, et al. Swin transformer: Hierarchical vision transformer using shifted windows. In: Proceedings of the IEEE/CVF international conference on computer vision; 2021. p. 10012–10022.
36. Liang J, Cao J, Sun G, Zhang K, Van Gool L, Timofte R. Swinir: Image restoration using swin transformer. In: Proceedings of the IEEE/CVF international conference on computer vision; 2021. p. 1833–1844.
37. Zhang Y, Zhu Y, Nichols E, Wang Q, Zhang S, Smith C, et al. A poisson-gaussian denoising dataset with real fluorescence microscopy images. In: Proceedings of the IEEE/CVF Conference on Computer Vision and Pattern Recognition; 2019. p. 11710–11718.
38. Zhou R, Helou ME, Sage D, Laroche T, Seitz A, Süsstrunk S. W2S: microscopy data with joint denoising and super-resolution for widefield to SIM mapping. In: European Conference on Computer Vision Springer; 2020. p. 474–491.
39. Hagen GM, Bendesky J, Machado R, Nguyen TA, Kumar T, Ventura J. Fluorescence microscopy datasets for training deep neural networks. GigaScience 2021;10(5):giab032.
40. Karras C, Smedh M, Förster R, Deschout H, Fernandez-Rodriguez J, Heintzmann R. Successful optimization of reconstruction parameters in structured illumination microscopy–a practical guide. Optics Communications 2019;436:69–75.
41. Mueller M, Free Analysis and Interactive Reconstruction for Structured Illumination Microscopy. GitHub; 2016. https://github.com/fairSIM/fairSIM.git.
42. Shah ZH, Evaluation of Swin Transformer and knowledge transfer for denoising of super-resolution structured illumination microscopy data. GitHub; 2023. https://github.com/ZafranShah/Denoising_and_fine_tuning_of_SR-SIM_data.
43. Shah ZH, Evaluation of Swin Transformer and knowledge transfer for denoising of super-resolution structured illumination microscopy data. zenodo; 2023. https://doi.org/10.5281/zenodo.7626173.
44. Ching JY, Wong AKC, Chan KCC. Class-dependent discretization for inductive learning from continuous and mixed-mode data. IEEE Transactions on Pattern Analysis and Machine Intelligence 1995;17(7):641–651.
45. Hussain M, Bird JJ, Faria DR. A study on cnn transfer learning for image classification. In: UK Workshop on computational Intelligence Springer; 2018. p. 191–202.
46. Tan C, Sun F, Kong T, Zhang W, Yang C, Liu C. A survey on deep transfer learning. In: International conference on artificial neural networks Springer; 2018. p. 270–279.
47. Bengio Y. Deep learning of representations for unsupervised and transfer learning. In: Proceedings of ICML workshop on unsupervised and transfer learning JMLR Workshop and Conference Proceedings; 2012. p. 17–36.
48. Ng HW, Nguyen VD, Vonikakis V, Winkler S. Deep learning for emotion recognition on small datasets using transfer learning. In: Proceedings of the 2015 ACM on international conference on multimodal interaction; 2015. p. 443–449.
49. Nogueira K, Penatti OA, Dos Santos JA. Towards better exploiting convolutional neural networks for remote sensing scene classification. Pattern Recognition 2017;61:539–556.
50. Yosinski J, Clune J, Bengio Y, Lipson H. How transferable

are features in deep neural networks? Advances in neural information processing systems 2014;27.

51. Mao X, Shen C, Yang YB. Image restoration using very deep convolutional encoder-decoder networks with symmetric skip connections. Advances in neural information processing systems 2016;29.
52. Ronneberger O, Fischer P, Brox T. U-net: Convolutional networks for biomedical image segmentation. In: International Conference on Medical image computing and computer-assisted intervention Springer; 2015. p. 234–241.
53. Hore A, Ziou D. Image quality metrics: PSNR vs. SSIM. In: 2010 20th international conference on pattern recognition IEEE; 2010. p. 2366–2369.
54. Setiadi DRIM. PSNR vs SSIM: imperceptibility quality assessment for image steganography. Multimedia Tools and Applications 2021;80(6):8423–8444.
55. Parmar N, Vaswani A, Uszkoreit J, Kaiser L, Shazeer N, Ku A, et al. Image transformer. In: International conference on machine learning PMLR; 2018. p. 4055–4064.
56. Ranftl R, Bochkovskiy A, Koltun V. Vision transformers for dense prediction. In: Proceedings of the IEEE/CVF International Conference on Computer Vision; 2021. p. 12179–12188.

Personal Cover Click here to access/download;Personal Cover;GIGA_D_23_00044_Cover_Response_Revision_2.pdf

Title: Evaluation of Swin Transformer and knowledge transfer for denoising of super-resolution structured illumination microscopy data

Journal Name: GigaScience
Manuscript Number: GIGA-D-23-00044

Dear Dr. Zauner,

Thank you once again for your and the reviewers' appreciation and valuable comments on our manuscript entitled, **"Evaluation of Swin Transformer and knowledge transfer for denoising of super-resolution structured illumination microscopy data"**. We have carefully revised the manuscript according to the suggested changes. The changes in the manuscript text are highlighted by the yellow highlighter. The point-by-point responses are included in the response box.

Regarding the main concern of reviewer #3: We agree with you and reviewer #3 that the data acquired and processed using the 3D-SIM illumination and algorithm has higher axial resolution than 2D-SIM to some extent. However, the main objective of this work was to remove the noise and reconstruction artifacts from the lateral plane of 2D and 3D SIM data. The 2D slices were obtained from the 3D-SIM data using a slice-by-slice strategy. As a result, the network solely denoises the lateral planes of both types of data.

We are also grateful for your time and effort in discussing the matter with your experts. We hope that our responses address the concerns of the reviewers. We believe that the manuscript is now suitable for publication in GigaScience.

All the results at GigaDB are updated and the new code is uploaded to the GitHub repository.

Reviewer # 1

Comment #1: In the "Response-5", the authors declared that "dataset 1 can be used to denoise raw SIM data, dataset 2 can be used to denoise SR-SIM data, and dataset 3 can be used for super-resolution tasks or joint denoising and super-resolution tasks." While, dataset 1 contains the functionality provided by datasets 2-3 because the users can utilize the fairSIM and SoftWorX for reconstructing the SR-SIM images in datasets 2-3 from the raw SIM data in dataset 1. And the authors may provide other sample structures besides tubulin filaments and vesicles in the form of dataset 1.

Response #1: Thank you again for your review and constructive feedback. We believe your last suggestion is very useful, but our current circumstances limit us to these two available sample structures (i.e., tubulin filaments and vesicles). Nevertheless, we believe that these structures can be very useful for the development, evaluation, and validation of future Deep Learning-based image recovery methods in the field of SIM microscopy. In addition, we would like to point out that datasets 2 and 3 may be very useful for readers who come from the machine learning field and do not yet have experience using fairSIM or SoftWorX.

Reviewer # 2:

Comment #1: The authors mentioned the pixel size in their response, but I would like to see a more detailed explanation in the paper about the relationship between pixel size and the external parameters of the microscope setup. This is crucial information for the dataset.

Response #1: Thank you for the review and the appreciation in your feedback. The choice of pixel size in a microscope depends on two main factors: the camera pixel size, and the total magnification. Higher resolutions are achieved by using smaller camera pixels and stronger magnifications, but the former at the expense of sensitivity (larger pixels are more sensitive) and the latter at the cost of field of view (higher magnifications decrease the imaging area). The OMX microscope uses a scientific camera for wide-field detection. Thus, the effective (aka projected) pixel size in the sample plane is given by the physical pixel size of the camera and the magnification of the optical system, i.e. the combination of the objective lens and any intermediate optics. For the OMX, the camera pixel size is 6.5 µm and the magnification is 82x, yielding an effective pixel size of 6.5 µm/82 = 80nm. The choice of magnification and thus effective pixel size is not arbitrary for a high-resolution imaging system. The imaging system has an inherent maximum spatial resolution that can be detected (Abbe limit), for a sample in water (n=1.33) and at blue wavelength (425nm) which is 160nm. Since detection by a pixelated camera is a discrete sampling process, the Nyquist criterion is now used to determine the maximum pixel size allowed to fully sample a signal with 160nm spatial resolution, which is 80nm. Larger pixels would miss high-frequency information that the optical system still transmits. Smaller pixels would fully sample all information, but introduce additional noise, as each pixel contributes an amount of read noise, and thus the total number of pixels should be kept small. More effects, especially dynamic range, can enter

into a pixel size determination, but this is the basic process. As a commercial microscope system, the pixel size of the OMX is constrained by the manufacturer to 80 nm, but it is likely the follow the above reasoning.
A shorter version of this explanation is now included in the manuscript at the beginning of "Materials and Methods – SR-SIM microscopy and sample preparation".

Comment #2: If the noise was not added to the image, how were the variables controlled to ensure a consistent noise level in each capture? In low-light conditions, changes in camera parameters can significantly affect the level of noise in the captured images.

Response #2: Several imaging parameters such as exposure time and illumination intensity can be adjusted in SIM microscopy to obtain samples with a high signal-to-noise ratio. Higher photon counts (N) typically result in samples with a higher signal-to-noise ratio. In SIM imaging, we typically control for two types of noise: Poisson noise, that is the variation in the number of photons being converted into photo-electrons in each pixel, and thermal and read noise, summarizing the error in counting the photo-electrons in the digitization process. The latter, the error introduced every time when reading out the camera, is very reproducible as long as two parameters are controlled for Camera temperature (influencing thermal and sometimes read noise) and exposure time (influencing mostly thermal noise). In the data sets at hand, we thus kept constant exposure time and temperature of the camera system in the OMX by cooling liquid.
In contrast, Poisson noise is a property of both illumination intensity and the specific sample. We can and do control illumination intensity, but the sample-to-sample variations (different effectiveness of the staining, variation in photo-bleaching) cannot be controlled. Therefore, we believe that these variations should be part of the datasets, since they are to be expected in real data and denoising networks must be able to deal with them.
Some remarks addressing the different types of noise are now included in the manuscript at the end of "Materials and Methods – SR-SIM microscopy and sample preparation".

Reviewer # 3:

Comment #1: In the revised version, the manuscript's quality has seen notable improvements, particularly due to the incorporation of results obtained using the Transformer-based SR-SIM image denoising algorithm. However, it remains concerning that the authors have not adequately addressed my comments, resulting in persisting weaknesses within the manuscript. One significant concern is that, regardless of whether 9 or 15 raw SIM images are employed in 2D reconstruction, the axial resolution consistently falls short compared to that achieved in 3D reconstruction. Consequently, the statement asserting that "this rules out the different axial resolution difference" is entirely inaccurate. Due to these unresolved issues, I am unable to ascertain the model's generalization capability across the four datasets. Moreover, the 'BioSR' dataset and other publicly available data showcasing new deep learning techniques for SIM denoising and reconstruction seem to overshadow the significance of the four datasets presented in this manuscript. Given the low image quality demonstrated within the manuscript, my confidence in the datasets' usefulness for the benefit of the scientific community has significantly diminished.

Response #1: Thank you for your review. Our previous statement "this rules out the different axial resolution difference" was unfortunately misleading. We fully agree that when a 3D SIM dataset is acquired and processed by 3D SIM algorithms, a higher axial resolution can be expected than for 2D SIM reconstruction (to some extent independent of the acquisition mode), since the axial resolution in SIM data depends on the illumination and the SIM reconstruction scheme [Gustafsson 2008]. Nevertheless, the SIM illumination and reconstruction algorithm for 3D SIM produces more noise and pattern artifacts in the lateral (XY) direction than in the axial (Z) direction [Gustafsson 2008]. Thus, a slice-by-slice (XY) strategy can still be useful to remove noise and artifacts introduced laterally by the reconstruction process for 3D SIM data.

In our work, we focus exclusively on the lateral plane and compare the generalization ability of denoising networks of 2D and 3D SIM microscopy data in the lateral plane using individual slices. An extension into the axial direction is out of the scope of the presented work and was not intended. We improved the manuscript by making this point clear at several locations:

- End of subsection “Dataset preprocessing and image reconstruction”
- Description of dataset 4
- New final sentence of the discussion section

As for the image quality in the datasets, we tend to disagree here, with all due respect. The images are of the usual quality that can be expected as the output of a SIM acquisition and processing toolchain for the intended cell structures. In particular, the reference images with high signal-to-noise ratios clearly show this.

Reference:

[Gustafsson 2008]: Gustafsson, Mats GL, et al. "Three-dimensional resolution doubling in wide-field fluorescence microscopy by structured illumination." Biophysical journal 94.12 (2008): 4957-4970.
