# Supplementary material for: Evaluation of Swin Transformer and knowledge transfer for denoising of super-resolution structured illumination microscopy data
Source: Gigascience. 2024 Jan 13;13:giad109. doi: 10.1093/gigascience/giad109 (PMC10787368; doi:10.1093/gigascience/giad109)
Supplement: giad109_GIGA-D-23-00044_Revision_3 [file giad109_giga-d-23-00044_revision_3.pdf]

GigaScience

Evaluation of Swin Transformer and knowledge transfer for denoising of super-resolution structured illumination microscopy data

--Manuscript Draft--

**Manuscript Number:**	GIGA-D-23-00044R3
**Full Title:**	Evaluation of Swin Transformer and knowledge transfer for denoising of super-resolution structured illumination microscopy data
**Article Type:**	Research
**Funding Information:**	H2020 Marie Skłodowska-Curie Actions (642157) — Mr. Thomas Huser<br>Deutsche Forschungsgemeinschaft (415832635) — Mr. Thomas Huser<br>EFRE-NRW funding programme Forschungsinfrastrukture (34.EFRE- 0300180) — Mr. Wolfram Schenck<br>SAIL: SustAInable Lifecycle of Intelligent SocioTechnical Systems funding programme Netzwerke 2021 (NW21-059B) — Mr. Wolfram Schenck
**Abstract:**	Background:<br>Convolutional Neural Network (CNN)-based methods have shown excellent performance in denoising and reconstruction of super-resolved structured illumination microscopy (SR-SIM) data. Therefore, CNN-based architectures have been the focus of existing studies. However, Swin Transformer, an alternative and recently proposed deep learning-based image restoration architecture, has not been fully investigated for denoising SR-SIM images. Furthermore, it has not been fully explored how well transfer learning strategies work for denoising SR-SIM images with different noise characteristics and recorded cell structures for these different types of deep learning-based methods. Currently, the scarcity of publicly available SR-SIM datasets limits the exploration of the performance and generalization capabilities of deep learning methods.<br>Results:<br>In this work, we present SwinT-fairSIM, a novel method based on the Swin Transformer for restoring SR-SIM images with low signal-to-noise ratio (SNR). The experimental results show that SwinT-fairSIM outperforms previous CNN-based denoising methods. Furthermore, as a second contribution, two types of transfer learning, namely direct transfer and fine-tuning, were benchmarked in combination with SwinT-fairSIM and two CNN-based methods for denoising SR-SIM data. Direct transfer did not prove to be a viable strategy, but fine-tuning produced results comparable to conventional training from scratch, while saving computational time and potentially reducing the amount of training data required. As a third contribution, we publish four datasets of raw SIM images and already reconstructed SR-SIM images. These datasets cover two different types of cell structures, tubulin filaments and vesicle structures. Different noise levels are available for the tubulin filaments.<br>Conclusions:<br>In conclusion, the SwinT-fairSIM method is well suited for denoising SR-SIM images. By fine-tuning, already trained models can be easily adapted to different noise characteristics and cell structures. Furthermore, the provided datasets are structured in a way that the research community can readily use them for research on denoising, super-resolution, and transfer learning strategies.
**Corresponding Author:**	Zafran Hussain Shah<br>Bielefeld University of Applied Sciences: Hochschule Bielefeld<br>Bielefeld, North Rhine-Westphalia GERMANY
**Corresponding Author Secondary Information:**	
**Corresponding Author's Institution:**	Bielefeld University of Applied Sciences: Hochschule Bielefeld
**Corresponding Author's Secondary**	

Powered by Editorial Manager® and ProduXion Manager® from Aries Systems Corporation

**Institution:**	
**First Author:**	Zafran Hussain Shah
**First Author Secondary Information:**	
**Order of Authors:**	Zafran Hussain Shah
	Marcel Müller, Doctorate
	Wolfgang Hübner, Doctorate
	Tung-Cheng Wang, Doctorate
	Daniel Telman, Bachelor
	Thomas Huser, Doctorate
	Wolfram Schenck, Doctorate
**Order of Authors Secondary Information:**	
**Response to Reviewers:**	Title: Evaluation of Swin Transformer and knowledge transfer for denoising of super-resolution structured illumination microscopy data<br>Journal Name: GigaScience<br>Manuscript Number: GIGA-D-23-00044<br><br>Dear Dr. Zauner,<br><br>Thank you once again for your efforts. Similarly, a special thanks to the GigaDB team for their cooperation and efforts in data publication and release. We carefully revised the manuscript to reflect the necessary changes. The editorial points are addressed below:<br><br>1) Please structure your abstract (Background - Results - Conclusions)<br><br>- We have restructured the abstract into these three parts.<br><br>2) Please add ORCIds to the title page (don't worry about the format for now - just a list in plain text is fine). We have these on our files: ORCID iDs: Zafran Hussain Shah [0000-0001-8721-3882]; Marcel Müller [0000-0002-2264-3643]; Wolfgang Hübner [0000-0002-1128-6468]; Tung-Cheng Wang; Daniel Telman; Thomas Huser [0000-0003-2348-7416]; Wolfram Schenck [0000-0003-3300-2048];<br><br>- We have added the ORCIds to the title page along with the author names. Please see the pdf of the manuscript 'Manuscript_GIGA-D-23-00044_Revisions'. The style file (i.e.,"orcidlink.sty") is also attached for your convenience.<br><br>3) Please add the GigaDB citation (see below) to the bibliography and cite it by number from the data availability section (I see you already have a "placeholder" where the GigaDB set will be cited).<br><br>- We filled all the placeholders with the GigaDB citation number.<br><br>4) Please also mention the Zenodo repo (reference [43]) again in the availability section, if it's not redundant with the GigaDb set.<br><br>- We added the reference in the zenodo repo (reference [44]) to the availability section.<br><br>5) For the code (reference [42]), please add a section "Availability of supporting source code and requirements" at the end.<br><br>- We have included a new section called "Availability of supporting source code and

	requirements" which provides information about the project and its requirements. The computational workflows are also registered in workflowhub.eu and DOIs is cited in this section. Similarly, we received the RRID from the Scicrunch.org database after registering our tool, which is also cited in this section of the manuscript. Regarding bio.tools, unfortunately we were not able to create an account on the bio.tools website (i.e. the link is down). I hope the registrations at scicrunch and workflowhub are sufficient for reproducibility and reuse of your tool.<br><br>6) In the data availability section, you write "All the datasets are distributed under the Creative Commons CCO waiver, with a request for attribution. The CCO permits the unrestricted reuse, distribution and reproduction, provided the original work is properly cited."<br><br>- We removed the second sentence to make it simpler and more convenient.<br><br>We believe that the necessary points have been addressed, and the manuscript is now ready for publication.
Additional Information:	
Question	Response
Are you submitting this manuscript to a special series or article collection?	No
**Experimental design and statistics**<br><br>Full details of the experimental design and statistical methods used should be given in the Methods section, as detailed in our Minimum Standards Reporting Checklist. Information essential to interpreting the data presented should be made available in the figure legends.<br><br>Have you included all the information requested in your manuscript?	Yes
**Resources**<br><br>A description of all resources used, including antibodies, cell lines, animals and software tools, with enough information to allow them to be uniquely identified, should be included in the Methods section. Authors are strongly encouraged to cite Research Resource Identifiers (RRIDs) for antibodies, model organisms and tools, where possible.<br><br>Have you included the information requested as detailed in our Minimum	Yes

Standards Reporting Checklist?	
**Availability of data and materials**<br><br>All datasets and code on which the conclusions of the paper rely must be either included in your submission or deposited in publicly available repositories (where available and ethically appropriate), referencing such data using a unique identifier in the references and in the "Availability of Data and Materials" section of your manuscript.<br><br>Have you have met the above requirement as detailed in our Minimum Standards Reporting Checklist?	Yes

LaTeX - Main Document (TeX file)

Click here to access/download;LaTeX - Main Document (TeX file);main_manuscript.tex

```
This is pdfTeX, Version 3.141592653-2.6-1.40.24 (TeX Live 2022)
(preloaded format=pdflatex 2023.3.8)  30 NOV 2023 09:25
entering extended mode
 restricted \write18 enabled.
 %&-line parsing enabled.
**main_manuscript.tex
(./main_manuscript.tex
LaTeX2e <2022-11-01> patch level 1
L3 programming layer <2023-02-22> (./oup-contemporary.cls
Document Class: oup-contemporary 2023/06/12, v1.2
(c:/TeXLive/2022/texmf-dist/tex/latex/base/article.cls
Document Class: article 2022/07/02 v1.4n Standard LaTeX document class
(c:/TeXLive/2022/texmf-dist/tex/latex/base/size10.clo
File: size10.clo 2022/07/02 v1.4n Standard LaTeX file (size option)
)
\c@part=\count185
\c@section=\count186
\c@subsection=\count187
\c@subsubsection=\count188
\c@paragraph=\count189
\c@subparagraph=\count190
\c@figure=\count191
\c@table=\count192
\abovecaptionskip=\skip48
\belowcaptionskip=\skip49
\bibindent=\dimen140
) (c:/TeXLive/2022/texmf-dist/tex/latex/base/inputenc.sty
Package: inputenc 2021/02/14 v1.3d Input encoding file
\inpenc@prehook=\toks16
\inpenc@posthook=\toks17
) (c:/TeXLive/2022/texmf-dist/tex/latex/base/fontenc.sty
Package: fontenc 2021/04/29 v2.0v Standard LaTeX package
) (c:/TeXLive/2022/texmf-dist/tex/generic/iftex/ifpdf.sty
Package: ifpdf 2019/10/25 v3.4 ifpdf legacy package. Use iftex instead.
(c:/TeXLive/2022/texmf-dist/tex/generic/iftex/iftex.sty
Package: iftex 2022/02/03 v1.0f TeX engine tests
)) (c:/TeXLive/2022/texmf-dist/tex/latex/microtype/microtype.sty
Package: microtype 2023/03/13 v3.1a Micro-typographical refinements (RS)
(c:/TeXLive/2022/texmf-dist/tex/latex/graphics/keyval.sty
Package: keyval 2022/05/29 v1.15 key=value parser (DPC)
\KV@toks@=\toks18
) (c:/TeXLive/2022/texmf-dist/tex/latex/etoolbox/etoolbox.sty
Package: etoolbox 2020/10/05 v2.5k e-TeX tools for LaTeX (JAW)
\etb@tempcnta=\count193
)
\MT@toks=\toks19
\MT@tempbox=\box51
\MT@count=\count194
LaTeX Info: Redefining \noprotrusionifhmode on input line 1059.
LaTeX Info: Redefining \leftprotrusion on input line 1060.
\MT@prot@toks=\toks20
LaTeX Info: Redefining \rightprotrusion on input line 1078.
LaTeX Info: Redefining \textls on input line 1368.
\MT@outer@kern=\dimen141
```

```
LaTeX Info: Redefining \textmicrotypecontext on input line 1988.
\MT@listname@count=\count195
(c:/TeXLive/2022/texmf-dist/tex/latex/microtype/microtype-pdftex.def
File: microtype-pdftex.def 2023/03/13 v3.1a Definitions specific to
pdftex (RS)

LaTeX Info: Redefining \lsstyle on input line 902.
LaTeX Info: Redefining \lslig on input line 902.
\MT@outer@space=\skip50
)
Package microtype Info: Loading configuration file microtype.cfg.
(c:/TeXLive/2022/texmf-dist/tex/latex/microtype/microtype.cfg
File: microtype.cfg 2023/03/13 v3.1a microtype main configuration file
(RS)
)) (c:/TeXLive/2022/texmf-dist/tex/latex/euler/euler.sty
Package: euler 1995/03/05 v2.5
Package: `euler' v2.5 <1995/03/05> (FJ and FMi)
LaTeX Font Info:    Redeclaring symbol font `letters' on input line 35.
LaTeX Font Info:    Encoding `OML' has changed to `U' for symbol font
(Font)              `letters' in the math version `normal' on input line
35.
LaTeX Font Info:    Overwriting symbol font `letters' in version `normal'
(Font)                  OML/cmm/m/it --> U/eur/m/n on input line 35.
LaTeX Font Info:    Encoding `OML' has changed to `U' for symbol font
(Font)              `letters' in the math version `bold' on input line
35.
LaTeX Font Info:    Overwriting symbol font `letters' in version `bold'
(Font)                  OML/cmm/b/it --> U/eur/m/n on input line 35.
LaTeX Font Info:    Overwriting symbol font `letters' in version `bold'
(Font)                  U/eur/m/n --> U/eur/b/n on input line 36.
LaTeX Font Info:    Redeclaring math symbol \Gamma on input line 47.
LaTeX Font Info:    Redeclaring math symbol \Delta on input line 48.
LaTeX Font Info:    Redeclaring math symbol \Theta on input line 49.
LaTeX Font Info:    Redeclaring math symbol \Lambda on input line 50.
LaTeX Font Info:    Redeclaring math symbol \Xi on input line 51.
LaTeX Font Info:    Redeclaring math symbol \Pi on input line 52.
LaTeX Font Info:    Redeclaring math symbol \Sigma on input line 53.
LaTeX Font Info:    Redeclaring math symbol \Upsilon on input line 54.
LaTeX Font Info:    Redeclaring math symbol \Phi on input line 55.
LaTeX Font Info:    Redeclaring math symbol \Psi on input line 56.
LaTeX Font Info:    Redeclaring math symbol \Omega on input line 57.
\symEulerFraktur=\mathgroup4
LaTeX Font Info:    Overwriting symbol font `EulerFraktur' in version
`bold'
(Font)                  U/euf/m/n --> U/euf/b/n on input line 63.
LaTeX Info: Redefining \oldstylenums on input line 85.
\symEulerScript=\mathgroup5
LaTeX Font Info:    Overwriting symbol font `EulerScript' in version
`bold'
(Font)                  U/eus/m/n --> U/eus/b/n on input line 93.
LaTeX Font Info:    Redeclaring math symbol \aleph on input line 97.
LaTeX Font Info:    Redeclaring math symbol \Re on input line 98.
LaTeX Font Info:    Redeclaring math symbol \Im on input line 99.
LaTeX Font Info:    Redeclaring math delimiter \vert on input line 101.
```

```
LaTeX Font Info:    Redeclaring math delimiter \backslash on input line
103.
LaTeX Font Info:    Redeclaring math symbol \neg on input line 106.
LaTeX Font Info:    Redeclaring math symbol \wedge on input line 108.
LaTeX Font Info:    Redeclaring math symbol \vee on input line 110.
LaTeX Font Info:    Redeclaring math symbol \setminus on input line 112.
LaTeX Font Info:    Redeclaring math symbol \sim on input line 113.
LaTeX Font Info:    Redeclaring math symbol \mid on input line 114.
LaTeX Font Info:    Redeclaring math delimiter \arrowvert on input line
116.
LaTeX Font Info:    Redeclaring math symbol \mathsection on input line
117.
\symEulerExtension=\mathgroup6
LaTeX Font Info:    Redeclaring math symbol \coprod on input line 125.
LaTeX Font Info:    Redeclaring math symbol \prod on input line 125.
LaTeX Font Info:    Redeclaring math symbol \sum on input line 125.
LaTeX Font Info:    Redeclaring math symbol \intop on input line 130.
LaTeX Font Info:    Redeclaring math symbol \ointop on input line 131.
LaTeX Font Info:    Redeclaring math symbol \braceld on input line 132.
LaTeX Font Info:    Redeclaring math symbol \bracerd on input line 133.
LaTeX Font Info:    Redeclaring math symbol \bracelu on input line 134.
LaTeX Font Info:    Redeclaring math symbol \braceru on input line 135.
LaTeX Font Info:    Redeclaring math symbol \infty on input line 136.
LaTeX Font Info:    Redeclaring math symbol \nearrow on input line 153.
LaTeX Font Info:    Redeclaring math symbol \searrow on input line 154.
LaTeX Font Info:    Redeclaring math symbol \nwarrow on input line 155.
LaTeX Font Info:    Redeclaring math symbol \swarrow on input line 156.
LaTeX Font Info:    Redeclaring math symbol \Leftrightarrow on input line
157.
LaTeX Font Info:    Redeclaring math symbol \Leftarrow on input line 158.
LaTeX Font Info:    Redeclaring math symbol \Rightarrow on input line
159.
LaTeX Font Info:    Redeclaring math symbol \leftrightarrow on input line
160.
LaTeX Font Info:    Redeclaring math symbol \leftarrow on input line 161.
LaTeX Font Info:    Redeclaring math symbol \rightarrow on input line
163.
LaTeX Font Info:    Redeclaring math delimiter \uparrow on input line
166.
LaTeX Font Info:    Redeclaring math delimiter \downarrow on input line
168.
LaTeX Font Info:    Redeclaring math delimiter \updownarrow on input line
170.
LaTeX Font Info:    Redeclaring math delimiter \Uparrow on input line
172.
LaTeX Font Info:    Redeclaring math delimiter \Downarrow on input line
174.
LaTeX Font Info:    Redeclaring math delimiter \Updownarrow on input line
176.
LaTeX Font Info:    Redeclaring math symbol \leftharpoonup on input line
177.
LaTeX Font Info:    Redeclaring math symbol \leftharpoondown on input
line 178.
```

```
LaTeX Font Info:    Redeclaring math symbol \rightharpoonup on input line
179.
LaTeX Font Info:    Redeclaring math symbol \rightharpoondown on input
line 180
.
LaTeX Font Info:    Redeclaring math delimiter \lbrace on input line 182.
LaTeX Font Info:    Redeclaring math delimiter \rbrace on input line 184.
\symcmmigroup=\mathgroup7
LaTeX Font Info:    Overwriting symbol font `cmmigroup' in version `bold'
(Font)                  OML/cmm/m/it --> OML/cmm/b/it on input line 200.
LaTeX Font Info:    Redeclaring math accent \vec on input line 201.
LaTeX Font Info:    Redeclaring math symbol \triangleleft on input line
202.
LaTeX Font Info:    Redeclaring math symbol \triangleright on input line
203.
LaTeX Font Info:    Redeclaring math symbol \star on input line 204.
LaTeX Font Info:    Redeclaring math symbol \lhook on input line 205.
LaTeX Font Info:    Redeclaring math symbol \rhook on input line 206.
LaTeX Font Info:    Redeclaring math symbol \flat on input line 207.
LaTeX Font Info:    Redeclaring math symbol \natural on input line 208.
LaTeX Font Info:    Redeclaring math symbol \sharp on input line 209.
LaTeX Font Info:    Redeclaring math symbol \smile on input line 210.
LaTeX Font Info:    Redeclaring math symbol \frown on input line 211.
LaTeX Font Info:    Redeclaring math accent \grave on input line 245.
LaTeX Font Info:    Redeclaring math accent \acute on input line 246.
LaTeX Font Info:    Redeclaring math accent \tilde on input line 247.
LaTeX Font Info:    Redeclaring math accent \ddot on input line 248.
LaTeX Font Info:    Redeclaring math accent \check on input line 249.
LaTeX Font Info:    Redeclaring math accent \breve on input line 250.
LaTeX Font Info:    Redeclaring math accent \bar on input line 251.
LaTeX Font Info:    Redeclaring math accent \dot on input line 252.
LaTeX Font Info:    Redeclaring math accent \hat on input line 254.
) (c:/TeXLive/2022/texmf-dist/tex/latex/merriweather/merriweather.sty
Package: merriweather 2022/09/20 (Bob Tennent) Supports
Merriweather(Sans) font
s for all LaTeX engines.
(c:/TeXLive/2022/texmf-dist/tex/generic/iftex/ifxetex.sty
Package: ifxetex 2019/10/25 v0.7 ifxetex legacy package. Use iftex
instead.
) (c:/TeXLive/2022/texmf-dist/tex/generic/iftex/ifluatex.sty
Package: ifluatex 2019/10/25 v1.5 ifluatex legacy package. Use iftex
instead.
) (c:/TeXLive/2022/texmf-dist/tex/latex/base/textcomp.sty
Package: textcomp 2020/02/02 v2.0n Standard LaTeX package
) (c:/TeXLive/2022/texmf-dist/tex/latex/xkeyval/xkeyval.sty
Package: xkeyval 2022/06/16 v2.9 package option processing (HA)
(c:/TeXLive/2022/texmf-dist/tex/generic/xkeyval/xkeyval.tex
(c:/TeXLive/2022/te
xmf-dist/tex/generic/xkeyval/xkvutils.tex
\XKV@toks=\toks21
\XKV@tempa@toks=\toks22
)
\XKV@depth=\count196
File: xkeyval.tex 2014/12/03 v2.7a key=value parser (HA)
```

```
)) (c:/TeXLive/2022/texmf-dist/tex/latex/base/fontenc.sty
Package: fontenc 2021/04/29 v2.0v Standard LaTeX package
) (c:/TeXLive/2022/texmf-dist/tex/latex/fontaxes/fontaxes.sty
Package: fontaxes 2020/07/21 v1.0e Font selection axes
LaTeX Info: Redefining \upshape on input line 29.
LaTeX Info: Redefining \itshape on input line 31.
LaTeX Info: Redefining \slshape on input line 33.
LaTeX Info: Redefining \swshape on input line 35.
LaTeX Info: Redefining \scshape on input line 37.
LaTeX Info: Redefining \sscshape on input line 39.
LaTeX Info: Redefining \ulcshape on input line 41.
LaTeX Info: Redefining \textsw on input line 47.
LaTeX Info: Redefining \textssc on input line 48.
LaTeX Info: Redefining \textulc on input line 49.
)) (c:/TeXLive/2022/texmf-dist/tex/latex/mathastext/mathastext.sty
Package: mathastext 2022/11/04 v1.3y Use the text font in math mode (JFB)
\mst@exists@muskip=\muskip16
\mst@forall@muskip=\muskip17
\mst@prime@muskip=\muskip18
\mst@do@nonletters=\toks23
\mst@do@easynonletters=\toks24
\mst@do@az=\toks25
\mst@do@AZ=\toks26
\symmtoperatorfont=\mathgroup8
\symmtletterfont=\mathgroup9
** ! and ?
** punctuation: , . : ; and \colon
LaTeX Info: Redefining \relbar on input line 844.
LaTeX Info: Redefining \rightarrowfill on input line 847.
LaTeX Info: Redefining \leftarrowfill on input line 852.
** + and =
LaTeX Info: Redefining \Relbar on input line 943.
** adding = ; and + to \nfss@catcodes
** parentheses ( ) [ ] and slash /
** alldelims: < > \backslash \setminus | \vert \mid \{ and \}
LaTeX Font Info:    Redeclaring math delimiter \backslash on input line
989.
LaTeX Font Info:    Redeclaring math symbol \setminus on input line 1001.
LaTeX Info: Redefining \models on input line 1010.
** \# \mathdollar \% \&
** \imath and \jmath
LaTeX Font Info:    Overwriting math alphabet `\mathnormalbold' in
version `nor
mal'
(Font)                  T1/Merriwthr-OsF/b/it --> T1/Merriwthr-OsF/b/it
on inpu
t line 2370.
LaTeX Font Info:    Overwriting math alphabet `\mathnormalbold' in
version `bol
d'
(Font)                  T1/Merriwthr-OsF/b/it --> T1/Merriwthr-OsF/b/it
on inpu
t line 2370.
```

```
LaTeX Font Info:    Overwriting symbol font `mtletterfont' in version
`normal'
(Font)                  T1/Merriwthr-OsF/m/it --> T1/Merriwthr-OsF/m/it
on inpu
t line 2370.
LaTeX Font Info:    Overwriting symbol font `mtletterfont' in version
`bold'
(Font)                  T1/Merriwthr-OsF/m/it --> T1/Merriwthr-OsF/b/it
on inpu
t line 2370.
LaTeX Font Info:    Overwriting symbol font `mtoperatorfont' in version
`normal
'
(Font)                  T1/Merriwthr-OsF/m/n --> T1/Merriwthr-OsF/m/n on
input
line 2370.
LaTeX Font Info:    Overwriting symbol font `mtoperatorfont' in version
`bold'
(Font)                  T1/Merriwthr-OsF/m/n --> T1/Merriwthr-OsF/b/n on
input
line 2370.
LaTeX Font Info:    Overwriting math alphabet `\Mathbf' in version
`normal'
(Font)                  T1/Merriwthr-OsF/b/n --> T1/Merriwthr-OsF/b/n on
input
line 2370.
LaTeX Font Info:    Overwriting math alphabet `\Mathbf' in version `bold'
(Font)                  T1/Merriwthr-OsF/b/n --> T1/Merriwthr-OsF/b/n on
input
line 2370.
LaTeX Font Info:    Overwriting math alphabet `\Mathit' in version
`normal'
(Font)                  T1/Merriwthr-OsF/m/it --> T1/Merriwthr-OsF/m/it
on inpu
t line 2370.
LaTeX Font Info:    Overwriting math alphabet `\Mathit' in version `bold'
(Font)                  T1/Merriwthr-OsF/m/it --> T1/Merriwthr-OsF/b/it
on inpu
t line 2370.
LaTeX Font Info:    Overwriting math alphabet `\Mathsf' in version
`normal'
(Font)                  T1/MerriwthrSans-OsF/m/n --> T1/MerriwthrSans-
OsF/m/n o
n input line 2370.
LaTeX Font Info:    Overwriting math alphabet `\Mathsf' in version `bold'
(Font)                  T1/MerriwthrSans-OsF/m/n --> T1/MerriwthrSans-
OsF/b/n o
n input line 2370.
LaTeX Font Info:    Overwriting math alphabet `\Mathtt' in version
`normal'
(Font)                  T1/lmtt/m/n --> T1/lmtt/m/n on input line 2370.
LaTeX Font Info:    Overwriting math alphabet `\Mathtt' in version `bold'
(Font)                  T1/lmtt/m/n --> T1/lmtt/b/n on input line 2370.
** Latin letters in the `normal' (resp. `bold') math versions are now
```

```
** set up to use the fonts T1/Merriwthr-OsF/m(b)/it
** Other characters (digits, ...) and \log-like names will be
** typeset with the n shape.
** \hbar
** minus as endash
** \HUGE has been (re)-defined.
** mathastext has declared larger sizes for subscripts.
** To keep LaTeX defaults, use option `defaultmathsizes'.
) (c:/TeXLive/2022/texmf-dist/tex/latex/relsize/relsize.sty
Package: relsize 2013/03/29 ver 4.1
) (c:/TeXLive/2022/texmf-dist/tex/latex/ragged2e/ragged2e.sty
Package: ragged2e 2023/02/25 v3.4 ragged2e Package
\CenteringLeftskip=\skip51
\RaggedLeftLeftskip=\skip52
\RaggedRightLeftskip=\skip53
\CenteringRightskip=\skip54
\RaggedLeftRightskip=\skip55
\RaggedRightRightskip=\skip56
\CenteringParfillskip=\skip57
\RaggedLeftParfillskip=\skip58
\RaggedRightParfillskip=\skip59
\JustifyingParfillskip=\skip60
\CenteringParindent=\skip61
\RaggedLeftParindent=\skip62
\RaggedRightParindent=\skip63
\JustifyingParindent=\skip64
) (c:/TeXLive/2022/texmf-dist/tex/latex/xcolor/xcolor.sty
Package: xcolor 2022/06/12 v2.14 LaTeX color extensions (UK)
(c:/TeXLive/2022/texmf-dist/tex/latex/graphics-cfg/color.cfg
File: color.cfg 2016/01/02 v1.6 sample color configuration
)
Package xcolor Info: Driver file: pdftex.def on input line 227.
(c:/TeXLive/2022/texmf-dist/tex/latex/graphics-def/pdftex.def
File: pdftex.def 2022/09/22 v1.2b Graphics/color driver for pdftex
) (c:/TeXLive/2022/texmf-dist/tex/latex/graphics/mathcolor.ltx)
Package xcolor Info: Model `cmy' substituted by `cmy0' on input line
1353.
Package xcolor Info: Model `hsb' substituted by `rgb' on input line 1357.
Package xcolor Info: Model `RGB' extended on input line 1369.
Package xcolor Info: Model `HTML' substituted by `rgb' on input line
1371.
Package xcolor Info: Model `Hsb' substituted by `hsb' on input line 1372.
Package xcolor Info: Model `tHsb' substituted by `hsb' on input line
1373.
Package xcolor Info: Model `HSB' substituted by `hsb' on input line 1374.
Package xcolor Info: Model `Gray' substituted by `gray' on input line
1375.
Package xcolor Info: Model `wave' substituted by `hsb' on input line
1376.
) (c:/TeXLive/2022/texmf-dist/tex/latex/colortbl/colortbl.sty
Package: colortbl 2022/06/20 v1.0f Color table columns (DPC)
(c:/TeXLive/2022/texmf-dist/tex/latex/tools/array.sty
Package: array 2022/09/04 v2.5g Tabular extension package (FMi)
\col@sep=\dimen142
```

```
\ar@mcellbox=\box52
\extrarowheight=\dimen143
\NC@list=\toks27
\extratabsurround=\skip65
\backup@length=\skip66
\ar@cellbox=\box53
)
\everycr=\toks28
\minrowclearance=\skip67
\rownum=\count197
) (c:/TeXLive/2022/texmf-dist/tex/latex/graphics/graphicx.sty
Package: graphicx 2021/09/16 v1.2d Enhanced LaTeX Graphics (DPC,SPQR)
(c:/TeXLive/2022/texmf-dist/tex/latex/graphics/graphics.sty
Package: graphics 2022/03/10 v1.4e Standard LaTeX Graphics (DPC,SPQR)
(c:/TeXLive/2022/texmf-dist/tex/latex/graphics/trig.sty
Package: trig 2021/08/11 v1.11 sin cos tan (DPC)
) (c:/TeXLive/2022/texmf-dist/tex/latex/graphics-cfg/graphics.cfg
File: graphics.cfg 2016/06/04 v1.11 sample graphics configuration
)
Package graphics Info: Driver file: pdftex.def on input line 107.
)
\Gin@req@height=\dimen144
\Gin@req@width=\dimen145
) (c:/TeXLive/2022/texmf-dist/tex/latex/xpatch/xpatch.sty
(c:/TeXLive/2022/texm
f-dist/tex/latex/l3kernel/expl3.sty
Package: expl3 2023-02-22 L3 programming layer (loader)
(c:/TeXLive/2022/texmf-dist/tex/latex/l3backend/l3backend-pdftex.def
File: l3backend-pdftex.def 2023-01-16 L3 backend support: PDF output
(pdfTeX)
\l__color_backend_stack_int=\count198
\l__pdf_internal_box=\box54
))
Package: xpatch 2020/03/25 v0.3a Extending etoolbox patching commands
(c:/TeXLive/2022/texmf-dist/tex/latex/l3packages/xparse/xparse.sty
Package: xparse 2023-02-02 L3 Experimental document command parser
)) (c:/TeXLive/2022/texmf-dist/tex/latex/environ/environ.sty
Package: environ 2014/05/04 v0.3 A new way to define environments
(c:/TeXLive/2022/texmf-dist/tex/latex/trimspaces/trimspaces.sty
Package: trimspaces 2009/09/17 v1.1 Trim spaces around a token list
)
\@envbody=\toks29
) (c:/TeXLive/2022/texmf-dist/tex/latex/lastpage/lastpage.sty
Package: lastpage 2023/03/07 v2.0a lastpage: 2.09 or 2e? (HMM)
(c:/TeXLive/2022/texmf-dist/tex/latex/lastpage/lastpage2e.sty
Package: lastpage2e 2023/03/07 v2.0a Decide which 2e lastpage version to
use (H
MM)
(c:/TeXLive/2022/texmf-dist/tex/latex/lastpage/lastpagemodern.sty
Package: lastpagemodern 2023-03-07 v2.0a Refers to last page's name (HMM;
JPG)
)
)) (c:/TeXLive/2022/texmf-dist/tex/latex/graphics/rotating.sty
Package: rotating 2016/08/11 v2.16d rotated objects in LaTeX
```

```
(c:/TeXLive/2022/texmf-dist/tex/latex/base/ifthen.sty
Package: ifthen 2022/04/13 v1.1d Standard LaTeX ifthen package (DPC)
)
\c@r@tfl@t=\count199
\rotFPtop=\skip68
\rotFPbot=\skip69
\rot@float@box=\box55
\rot@mess@toks=\toks30
) (c:/TeXLive/2022/texmf-dist/tex/latex/graphics/lscape.sty
Package: lscape 2020/05/28 v3.02 Landscape Pages (DPC)
) (c:/TeXLive/2022/texmf-dist/tex/latex/tools/afterpage.sty
Package: afterpage 2014/10/28 v1.08 After-Page Package (DPC)
\AP@output=\toks31
\AP@partial=\box56
\AP@footins=\box57
) (c:/TeXLive/2022/texmf-dist/tex/latex/textpos/textpos.sty
Package: textpos 2022/07/23 v1.10.1
Package textpos Info: choosing support for LaTeX3 on input line 60.
\TP@textbox=\box58
\TP@holdbox=\box59
\TPHorizModule=\dimen146
\TPVertModule=\dimen147
\TP@margin=\dimen148
\TP@absmargin=\dimen149
Grid set 16 x 16 = 37.34424pt x 52.81541pt
\TPboxrulesize=\dimen150
\TP@ox=\dimen151
\TP@oy=\dimen152
\TP@tbargs=\toks32
TextBlockOrigin set to 0pt x 0pt
) (c:/TeXLive/2022/texmf-dist/tex/latex/url/url.sty
\Urlmuskip=\muskip19
Package: url 2013/09/16  ver 3.4  Verb mode for urls, etc.
) (c:/TeXLive/2022/texmf-dist/tex/latex/newfloat/newfloat.sty
Package: newfloat 2019/09/02 v1.1l Defining new floating environments
(AR)
Package newfloat Info: `rotating' package detected.
) (c:/TeXLive/2022/texmf-dist/tex/latex/mdframed/mdframed.sty
Package: mdframed 2013/07/01 1.9b: mdframed
(c:/TeXLive/2022/texmf-dist/tex/latex/kvoptions/kvoptions.sty
Package: kvoptions 2022-06-15 v3.15 Key value format for package options
(HO)
(c:/TeXLive/2022/texmf-dist/tex/generic/ltxcmds/ltxcmds.sty
Package: ltxcmds 2020-05-10 v1.25 LaTeX kernel commands for general use
(HO)
) (c:/TeXLive/2022/texmf-dist/tex/latex/kvsetkeys/kvsetkeys.sty
Package: kvsetkeys 2022-10-05 v1.19 Key value parser (HO)
)) (c:/TeXLive/2022/texmf-dist/tex/latex/zref/zref-abspage.sty
Package: zref-abspage 2022-04-07 v2.34 Module abspage for zref (HO)
(c:/TeXLive/2022/texmf-dist/tex/latex/zref/zref-base.sty
Package: zref-base 2022-04-07 v2.34 Module base for zref (HO)
(c:/TeXLive/2022/texmf-dist/tex/generic/infwarerr/infwarerr.sty
Package: infwarerr 2019/12/03 v1.5 Providing info/warning/error messages
(HO)
```

```
) (c:/TeXLive/2022/texmf-dist/tex/generic/kvdefinekeys/kvdefinekeys.sty
Package: kvdefinekeys 2019-12-19 v1.6 Define keys (HO)
) (c:/TeXLive/2022/texmf-dist/tex/generic/pdftexcmds/pdftexcmds.sty
Package: pdftexcmds 2020-06-27 v0.33 Utility functions of pdfTeX for
LuaTeX (HO
)
Package pdftexcmds Info: \pdf@primitive is available.
Package pdftexcmds Info: \pdf@ifprimitive is available.
Package pdftexcmds Info: \pdfdraftmode found.
) (c:/TeXLive/2022/texmf-dist/tex/generic/etexcmds/etexcmds.sty
Package: etexcmds 2019/12/15 v1.7 Avoid name clashes with e-TeX commands
(HO)
) (c:/TeXLive/2022/texmf-dist/tex/latex/auxhook/auxhook.sty
Package: auxhook 2019-12-17 v1.6 Hooks for auxiliary files (HO)
)
Package zref Info: New property list: main on input line 767.
Package zref Info: New property: default on input line 768.
Package zref Info: New property: page on input line 769.
) (c:/TeXLive/2022/texmf-dist/tex/latex/base/atbegshi-ltx.sty
Package: atbegshi-ltx 2021/01/10 v1.0c Emulation of the original atbegshi
package with kernel methods
)
\c@abspage=\count266
Package zref Info: New property: abspage on input line 65.
) (c:/TeXLive/2022/texmf-dist/tex/latex/needspace/needspace.sty
Package: needspace 2010/09/12 v1.3d reserve vertical space
)
\mdf@templength=\skip70
\c@mdf@globalstyle@cnt=\count267
\mdf@skipabove@length=\skip71
\mdf@skipbelow@length=\skip72
\mdf@leftmargin@length=\skip73
\mdf@rightmargin@length=\skip74
\mdf@innerleftmargin@length=\skip75
\mdf@innerrightmargin@length=\skip76
\mdf@innertopmargin@length=\skip77
\mdf@innerbottommargin@length=\skip78
\mdf@splittopskip@length=\skip79
\mdf@splitbottomskip@length=\skip80
\mdf@outermargin@length=\skip81
\mdf@innermargin@length=\skip82
\mdf@linewidth@length=\skip83
\mdf@innerlinewidth@length=\skip84
\mdf@middlelinewidth@length=\skip85
\mdf@outerlinewidth@length=\skip86
\mdf@roundcorner@length=\skip87
\mdf@footenotedistance@length=\skip88
\mdf@userdefinedwidth@length=\skip89
\mdf@needspace@length=\skip90
\mdf@frametitleaboveskip@length=\skip91
\mdf@frametitlebelowskip@length=\skip92
\mdf@frametitlerulewidth@length=\skip93
\mdf@frametitleleftmargin@length=\skip94
\mdf@frametitlerightmargin@length=\skip95
```

```
\mdf@shadowsize@length=\skip96
\mdf@extratopheight@length=\skip97
\mdf@subtitleabovelinewidth@length=\skip98
\mdf@subtitlebelowlinewidth@length=\skip99
\mdf@subtitleaboveskip@length=\skip100
\mdf@subtitlebelowskip@length=\skip101
\mdf@subtitleinneraboveskip@length=\skip102
\mdf@subtitleinnerbelowskip@length=\skip103
\mdf@subsubtitleabovelinewidth@length=\skip104
\mdf@subsubtitlebelowlinewidth@length=\skip105
\mdf@subsubtitleaboveskip@length=\skip106
\mdf@subsubtitlebelowskip@length=\skip107
\mdf@subsubtitleinneraboveskip@length=\skip108
\mdf@subsubtitleinnerbelowskip@length=\skip109
(c:/TeXLive/2022/texmf-dist/tex/latex/mdframed/md-frame-0.mdf
File: md-frame-0.mdf 2013/07/01\ 1.9b: md-frame-0
)
\mdf@frametitlebox=\box60
\mdf@footnotebox=\box61
\mdf@splitbox@one=\box62
\mdf@splitbox@two=\box63
\mdf@splitbox@save=\box64
\mdfsplitboxwidth=\skip110
\mdfsplitboxtotalwidth=\skip111
\mdfsplitboxheight=\skip112
\mdfsplitboxdepth=\skip113
\mdfsplitboxtotalheight=\skip114
\mdfframetitleboxwidth=\skip115
\mdfframetitleboxtotalwidth=\skip116
\mdfframetitleboxheight=\skip117
\mdfframetitleboxdepth=\skip118
\mdfframetitleboxtotalheight=\skip119
\mdffootnoteboxwidth=\skip120
\mdffootnoteboxtotalwidth=\skip121
\mdffootnoteboxheight=\skip122
\mdffootnoteboxdepth=\skip123
\mdffootnoteboxtotalheight=\skip124
\mdftotallinewidth=\skip125
\mdfboundingboxwidth=\skip126
\mdfboundingboxtotalwidth=\skip127
\mdfboundingboxheight=\skip128
\mdfboundingboxdepth=\skip129
\mdfboundingboxtotalheight=\skip130
\mdf@freevspace@length=\skip131
\mdf@horizontalwidthofbox@length=\skip132
\mdf@verticalmarginwhole@length=\skip133
\mdf@horizontalspaceofbox=\skip134
\mdfsubtitleheight=\skip135
\mdfsubsubtitleheight=\skip136
\c@mdfcountframes=\count268

****** mdframed patching \endmdf@trivlist

****** -- success******
```

```
\mdf@envdepth=\count269
\c@mdf@env@i=\count270
\c@mdf@env@ii=\count271
\c@mdf@zref@counter=\count272
Package zref Info: New property: mdf@pagevalue on input line 895.
) (c:/TeXLive/2022/texmf-dist/tex/latex/titlesec/titlesec.sty
Package: titlesec 2021/07/05 v2.14 Sectioning titles
\ttl@box=\box65
\beforetitleunit=\skip137
\aftertitleunit=\skip138
\ttl@plus=\dimen153
\ttl@minus=\dimen154
\ttl@toksa=\toks33
\titlewidth=\dimen155
\titlewidthlast=\dimen156
\titlewidthfirst=\dimen157
) (c:/TeXLive/2022/texmf-dist/tex/latex/koma-script/scrextend.sty
Package: scrextend 2022/10/12 v3.38 KOMA-Script package (extend other
classes w
ith features of KOMA-Script classes)
(c:/TeXLive/2022/texmf-dist/tex/latex/koma-script/scrkbase.sty
Package: scrkbase 2022/10/12 v3.38 KOMA-Script package (KOMA-Script-
dependent b
asics and keyval usage)
(c:/TeXLive/2022/texmf-dist/tex/latex/koma-script/scrbase.sty
Package: scrbase 2022/10/12 v3.38 KOMA-Script package (KOMA-Script-
independent
basics and keyval usage)
(c:/TeXLive/2022/texmf-dist/tex/latex/koma-script/scrlfile.sty
Package: scrlfile 2022/10/12 v3.38 KOMA-Script package (file load hooks)
(c:/TeXLive/2022/texmf-dist/tex/latex/koma-script/scrlfile-hook.sty
Package: scrlfile-hook 2022/10/12 v3.38 KOMA-Script package (using LaTeX
hooks)

(c:/TeXLive/2022/texmf-dist/tex/latex/koma-script/scrlogo.sty
Package: scrlogo 2022/10/12 v3.38 KOMA-Script package (logo)
)))
Applying: [2021/05/01] Usage of raw or classic option list on input line
252.
Already applied: [0000/00/00] Usage of raw or classic option list on
input line
 368.
))
Package scrextend Info: unexpected definition of `\@makefnmark'.
(scrextend)             Trying to patch it on input line 1709.
Package scrextend Info: patch seems to be successfull on input line 1709.
)

LaTeX Font Warning: Font shape `T1/cmr/m/n' in size <7.5> not available
(Font)              size <7> substituted on input line 66.

(c:/TeXLive/2022/texmf-dist/tex/latex/tools/calc.sty
Package: calc 2017/05/25 v4.3 Infix arithmetic (KKT,FJ)
```

```
\calc@Acount=\count273
\calc@Bcount=\count274
\calc@Adimen=\dimen158
\calc@Bdimen=\dimen159
\calc@Askip=\skip139
\calc@Bskip=\skip140
LaTeX Info: Redefining \setlength on input line 80.
LaTeX Info: Redefining \addtolength on input line 81.
\calc@Ccount=\count275
\calc@Cskip=\skip141
) (c:/TeXLive/2022/texmf-dist/tex/latex/geometry/geometry.sty
Package: geometry 2020/01/02 v5.9 Page Geometry
(c:/TeXLive/2022/texmf-dist/tex/generic/iftex/ifvtex.sty
Package: ifvtex 2019/10/25 v1.7 ifvtex legacy package. Use iftex instead.
)
\Gm@cnth=\count276
\Gm@cntv=\count277
\c@Gm@tempcnt=\count278
\Gm@bindingoffset=\dimen160
\Gm@wd@mp=\dimen161
\Gm@odd@mp=\dimen162
\Gm@even@mp=\dimen163
\Gm@layoutwidth=\dimen164
\Gm@layoutheight=\dimen165
\Gm@layouthoffset=\dimen166
\Gm@layoutvoffset=\dimen167
\Gm@dimlist=\toks34
) (c:/TeXLive/2022/texmf-dist/tex/latex/hyperref/hyperref.sty
Package: hyperref 2023-02-07 v7.00v Hypertext links for LaTeX
(c:/TeXLive/2022/texmf-dist/tex/generic/pdfescape/pdfescape.sty
Package: pdfescape 2019/12/09 v1.15 Implements pdfTeX's escape features
(HO)
) (c:/TeXLive/2022/texmf-dist/tex/latex/hycolor/hycolor.sty
Package: hycolor 2020-01-27 v1.10 Color options for hyperref/bookmark
(HO)
) (c:/TeXLive/2022/texmf-dist/tex/latex/letltxmacro/letltxmacro.sty
Package: letltxmacro 2019/12/03 v1.6 Let assignment for LaTeX macros (HO)
) (c:/TeXLive/2022/texmf-dist/tex/latex/hyperref/nameref.sty
Package: nameref 2022-05-17 v2.50 Cross-referencing by name of section
(c:/TeXLive/2022/texmf-dist/tex/latex/refcount/refcount.sty
Package: refcount 2019/12/15 v3.6 Data extraction from label references
(HO)
) (c:/TeXLive/2022/texmf-
dist/tex/generic/gettitlestring/gettitlestring.sty
Package: gettitlestring 2019/12/15 v1.6 Cleanup title references (HO)
)
\c@section@level=\count279
)
\@linkdim=\dimen168
\Hy@linkcounter=\count280
\Hy@pagecounter=\count281
(c:/TeXLive/2022/texmf-dist/tex/latex/hyperref/pd1enc.def
File: pd1enc.def 2023-02-07 v7.00v Hyperref: PDFDocEncoding definition
(HO)
```

```
Now handling font encoding PD1 ...
... no UTF-8 mapping file for font encoding PD1
) (c:/TeXLive/2022/texmf-dist/tex/generic/intcalc/intcalc.sty
Package: intcalc 2019/12/15 v1.3 Expandable calculations with integers
(HO)
)
\Hy@SavedSpaceFactor=\count282
(c:/TeXLive/2022/texmf-dist/tex/latex/hyperref/puenc.def
File: puenc.def 2023-02-07 v7.00v Hyperref: PDF Unicode definition (HO)
Now handling font encoding PU ...
... no UTF-8 mapping file for font encoding PU
)
Package hyperref Info: Option `colorlinks' set `true' on input line 4060.
Package hyperref Info: Hyper figures OFF on input line 4177.
Package hyperref Info: Link nesting OFF on input line 4182.
Package hyperref Info: Hyper index ON on input line 4185.
Package hyperref Info: Plain pages OFF on input line 4192.
Package hyperref Info: Backreferencing OFF on input line 4197.
Package hyperref Info: Implicit mode ON; LaTeX internals redefined.
Package hyperref Info: Bookmarks ON on input line 4425.
\c@Hy@tempcnt=\count283
LaTeX Info: Redefining \url on input line 4763.
\XeTeXLinkMargin=\dimen169
(c:/TeXLive/2022/texmf-dist/tex/generic/bitset/bitset.sty
Package: bitset 2019/12/09 v1.3 Handle bit-vector datatype (HO)
(c:/TeXLive/2022/texmf-dist/tex/generic/bigintcalc/bigintcalc.sty
Package: bigintcalc 2019/12/15 v1.5 Expandable calculations on big
integers (HO
)
))
\Fld@menulength=\count284
\Field@Width=\dimen170
\Fld@charsize=\dimen171
Package hyperref Info: Hyper figures OFF on input line 6042.
Package hyperref Info: Link nesting OFF on input line 6047.
Package hyperref Info: Hyper index ON on input line 6050.
Package hyperref Info: backreferencing OFF on input line 6057.
Package hyperref Info: Link coloring ON on input line 6060.
Package hyperref Info: Link coloring with OCG OFF on input line 6067.
Package hyperref Info: PDF/A mode OFF on input line 6072.
\Hy@abspage=\count285
\c@Item=\count286
\c@Hfootnote=\count287
)
Package hyperref Info: Driver (autodetected): hpdftex.
(c:/TeXLive/2022/texmf-dist/tex/latex/hyperref/hpdftex.def
File: hpdftex.def 2023-02-07 v7.00v Hyperref driver for pdfTeX
(c:/TeXLive/2022/texmf-dist/tex/latex/base/atveryend-ltx.sty
Package: atveryend-ltx 2020/08/19 v1.0a Emulation of the original
atveryend pac
kage
with kernel methods
)
\HyAnn@Count=\count288
```

```
\Fld@listcount=\count289
\c@bookmark@seq@number=\count290
(c:/TeXLive/2022/texmf-dist/tex/latex/rerunfilecheck/rerunfilecheck.sty
Package: rerunfilecheck 2022-07-10 v1.10 Rerun checks for auxiliary files
(HO)
(c:/TeXLive/2022/texmf-dist/tex/generic/uniquecounter/uniquecounter.sty
Package: uniquecounter 2019/12/15 v1.4 Provide unlimited unique counter
(HO)
)
Package uniquecounter Info: New unique counter `rerunfilecheck' on input
line 2
85.
)
\Hy@SectionHShift=\skip142
) (c:/TeXLive/2022/texmf-dist/tex/latex/preprint/authblk.sty
Package: authblk 2001/02/27 1.3 (PWD)
\affilsep=\skip143
\@affilsep=\skip144
\c@Maxaffil=\count291
\c@authors=\count292
\c@affil=\count293
) (c:/TeXLive/2022/texmf-dist/tex/latex/footmisc/footmisc.sty
Package: footmisc 2022/03/08 v6.0d a miscellany of footnote facilities
\FN@temptoken=\toks35
\footnotemargin=\dimen172
\@outputbox@depth=\dimen173
Package footmisc Info: Declaring symbol style bringhurst on input line
695.
Package footmisc Info: Declaring symbol style chicago on input line 703.
Package footmisc Info: Declaring symbol style wiley on input line 712.
Package footmisc Info: Declaring symbol style lamport-robust on input
line 723.

Package footmisc Info: Declaring symbol style lamport* on input line 743.
Package footmisc Info: Declaring symbol style lamport*-robust on input
line 764
.
) (c:/TeXLive/2022/texmf-dist/tex/latex/fancyhdr/fancyhdr.sty
Package: fancyhdr 2022/11/09 v4.1 Extensive control of page headers and
footers

\f@nch@headwidth=\skip145
\f@nch@O@elh=\skip146
\f@nch@O@erh=\skip147
\f@nch@O@olh=\skip148
\f@nch@O@orh=\skip149
\f@nch@O@elf=\skip150
\f@nch@O@erf=\skip151
\f@nch@O@olf=\skip152
\f@nch@O@orf=\skip153
) (c:/TeXLive/2022/texmf-dist/tex/generic/alphalph/alphalph.sty
Package: alphalph 2019/12/09 v2.6 Convert numbers to letters (HO)
)
\c@authorfn=\count294
```

```
(c:/TeXLive/2022/texmf-dist/tex/latex/abstract/abstract.sty
Package: abstract 2009/06/08 v1.2a configurable abstracts
\abstitleskip=\skip154
\absleftindent=\skip155
\absrightindent=\skip156
\absparindent=\skip157
\absparsep=\skip158
)
Package newfloat Info: New float `keypoints' with options
`placement=t!,name=kp
t' on input line 287.
\c@keypoints=\count295
\newfloat@ftype=\count296
Package newfloat Info: float type `keypoints'=8 on input line 287.
(c:/TeXLive/2022/texmf-dist/tex/latex/enumitem/enumitem.sty
Package: enumitem 2019/06/20 v3.9 Customized lists
\labelindent=\skip159
\enit@outerparindent=\dimen174
\enit@toks=\toks36
\enit@inbox=\box66
\enit@count@id=\count297
\enitdp@description=\count298
) (c:/TeXLive/2022/texmf-dist/tex/latex/quoting/quoting.sty
Package: quoting 2014/01/28 v0.1c Consolidated environment for displayed
text
\quo@toppartop=\skip160
) (c:/TeXLive/2022/texmf-dist/tex/latex/sttools/stfloats.sty
Package: stfloats 2017/03/27 v3.3 Improve float mechanism and
baselineskip sett
ings
\@dblbotnum=\count299
\c@dblbotnumber=\count300
) (c:/TeXLive/2022/texmf-dist/tex/latex/booktabs/booktabs.sty
Package: booktabs 2020/01/12 v1.61803398 Publication quality tables
\heavyrulewidth=\dimen175
\lightrulewidth=\dimen176
\cmidrulewidth=\dimen177
\belowrulesep=\dimen178
\belowbottomsep=\dimen179
\aboverulesep=\dimen180
\abovetopsep=\dimen181
\cmidrulesep=\dimen182
\cmidrulekern=\dimen183
\defaultaddspace=\dimen184
\@cmidla=\count301
\@cmidlb=\count302
\@aboverulesep=\dimen185
\@belowrulesep=\dimen186
\@thisruleclass=\count303
\@lastruleclass=\count304
\@thisrulewidth=\dimen187
) (c:/TeXLive/2022/texmf-dist/tex/latex/tools/tabularx.sty
Package: tabularx 2020/01/15 v2.11c `tabularx' package (DPC)
\TX@col@width=\dimen188
```

```
\TX@old@table=\dimen189
\TX@old@col=\dimen190
\TX@target=\dimen191
\TX@delta=\dimen192
\TX@cols=\count305
\TX@ftn=\toks37
)
\enitdp@tablenotes=\count306
(c:/TeXLive/2022/texmf-dist/tex/latex/caption/caption.sty
Package: caption 2022/03/01 v3.6b Customizing captions (AR)
(c:/TeXLive/2022/texmf-dist/tex/latex/caption/caption3.sty
Package: caption3 2022/03/17 v2.3b caption3 kernel (AR)
\caption@tempdima=\dimen193
\captionmargin=\dimen194
\caption@leftmargin=\dimen195
\caption@rightmargin=\dimen196
\caption@width=\dimen197
\caption@indent=\dimen198
\caption@parindent=\dimen199
\caption@hangindent=\dimen256
Package caption Info: Standard document class detected.
)
\c@caption@flags=\count307
\c@continuedfloat=\count308
Package caption Info: hyperref package is loaded.
Package caption Info: rotating package is loaded.
) (c:/TeXLive/2022/texmf-dist/tex/latex/natbib/natbib.sty
Package: natbib 2010/09/13 8.31b (PWD, AO)
\bibhang=\skip161
\bibsep=\skip162
LaTeX Info: Redefining \cite on input line 694.
\c@NAT@ctr=\count309
)) (c:/TeXLive/2022/texmf-dist/tex/latex/siunitx/siunitx.sty
Package: siunitx 2023-03-04 v3.2.2 A comprehensive (SI) units package
\l__siunitx_angle_tmp_dim=\dimen257
\l__siunitx_angle_marker_box=\box67
\l__siunitx_angle_unit_box=\box68
\l__siunitx_compound_count_int=\count310
(c:/TeXLive/2022/texmf-dist/tex/latex/translations/translations.sty
Package: translations 2022/02/05 v1.12 internationalization of LaTeX2e
packages
 (CN)
)
\l__siunitx_number_exponent_fixed_int=\count311
\l__siunitx_number_min_decimal_int=\count312
\l__siunitx_number_min_integer_int=\count313
\l__siunitx_number_round_precision_int=\count314
\l__siunitx_number_lower_threshold_int=\count315
\l__siunitx_number_upper_threshold_int=\count316
\l__siunitx_number_group_first_int=\count317
\l__siunitx_number_group_size_int=\count318
\l__siunitx_number_group_minimum_int=\count319
(c:/TeXLive/2022/texmf-dist/tex/latex/amsmath/amstext.sty
Package: amstext 2021/08/26 v2.01 AMS text
```

```
(c:/TeXLive/2022/texmf-dist/tex/latex/amsmath/amsgen.sty
File: amsgen.sty 1999/11/30 v2.0 generic functions
\@emptytoks=\toks38
\ex@=\dimen258
))
\l__siunitx_table_tmp_box=\box69
\l__siunitx_table_tmp_dim=\dimen259
\l__siunitx_table_column_width_dim=\dimen260
\l__siunitx_table_integer_box=\box70
\l__siunitx_table_decimal_box=\box71
\l__siunitx_table_uncert_box=\box72
\l__siunitx_table_before_box=\box73
\l__siunitx_table_after_box=\box74
\l__siunitx_table_before_dim=\dimen261
\l__siunitx_table_carry_dim=\dimen262
\l__siunitx_unit_tmp_int=\count320
\l__siunitx_unit_position_int=\count321
\l__siunitx_unit_total_int=\count322
) (c:/TeXLive/2022/texmf-dist/tex/latex/comment/comment.sty
\CommentStream=\write3
Excluding comment 'comment') (c:/TeXLive/2022/texmf-
dist/tex/latex/amsmath/amsm
ath.sty
Package: amsmath 2022/04/08 v2.17n AMS math features
\@mathmargin=\skip163
For additional information on amsmath, use the `?' option.
(c:/TeXLive/2022/texmf-dist/tex/latex/amsmath/amsbsy.sty
Package: amsbsy 1999/11/29 v1.2d Bold Symbols
\pmbraise@=\dimen263
) (c:/TeXLive/2022/texmf-dist/tex/latex/amsmath/amsopn.sty
Package: amsopn 2022/04/08 v2.04 operator names
)
\inf@bad=\count323
LaTeX Info: Redefining \frac on input line 234.
\uproot@=\count324
\leftroot@=\count325
LaTeX Info: Redefining \overline on input line 399.
LaTeX Info: Redefining \colon on input line 410.
\classnum@=\count326
\DOTSCASE@=\count327
LaTeX Info: Redefining \ldots on input line 496.
LaTeX Info: Redefining \dots on input line 499.
LaTeX Info: Redefining \cdots on input line 620.
\Mathstrutbox@=\box75
\strutbox@=\box76
LaTeX Info: Redefining \big on input line 722.
LaTeX Info: Redefining \Big on input line 723.
LaTeX Info: Redefining \bigg on input line 724.
LaTeX Info: Redefining \Bigg on input line 725.
\big@size=\dimen264
LaTeX Font Info:    Redeclaring font encoding OML on input line 743.
LaTeX Font Info:    Redeclaring font encoding OMS on input line 744.
\macc@depth=\count328
LaTeX Info: Redefining \bmod on input line 905.
```

```
LaTeX Info: Redefining \pmod on input line 910.
LaTeX Info: Redefining \smash on input line 940.
LaTeX Info: Redefining \relbar on input line 970.
LaTeX Info: Redefining \Relbar on input line 971.
\c@MaxMatrixCols=\count329
\dotsspace@=\muskip20
\c@parentequation=\count330
\dspbrk@lvl=\count331
\tag@help=\toks39
\row@=\count332
\column@=\count333
\maxfields@=\count334
\andhelp@=\toks40
\eqnshift@=\dimen265
\alignsep@=\dimen266
\tagshift@=\dimen267
\tagwidth@=\dimen268
\totwidth@=\dimen269
\lineht@=\dimen270
\@envbody=\toks41
\multlinegap=\skip164
\multlinetaggap=\skip165
\mathdisplay@stack=\toks42
LaTeX Info: Redefining \[ on input line 2953.
LaTeX Info: Redefining \] on input line 2954.
) (c:/TeXLive/2022/texmf-dist/tex/generic/soul/soul.sty
Package: soul 2023-02-18 v3.0 Permit use of UTF-8 characters in soul (HO)
(c:/TeXLive/2022/texmf-dist/tex/generic/soul/soul-ori.sty
Package: soul-ori 2023-02-18 v3.0 letterspacing/underlining (mf)
\SOUL@word=\toks43
\SOUL@lasttoken=\toks44
\SOUL@syllable=\toks45
\SOUL@cmds=\toks46
\SOUL@buffer=\toks47
\SOUL@token=\toks48
\SOUL@syllgoal=\dimen271
\SOUL@syllwidth=\dimen272
\SOUL@charkern=\dimen273
\SOUL@hyphkern=\dimen274
\SOUL@dimen=\dimen275
\SOUL@dimeni=\dimen276
\SOUL@minus=\count335
\SOUL@comma=\count336
\SOUL@apo=\count337
\SOUL@grave=\count338
\SOUL@spaceskip=\skip166
\SOUL@ttwidth=\dimen277
\SOUL@uldp=\dimen278
\SOUL@ulht=\dimen279
)) (c:/TeXLive/2022/texmf-dist/tex/latex/academicons/academicons.sty
Package: academicons 2021/11/26 v1.9.1 Academicons Icons
) (./orcidlink.sty
Package: orcidlink 2021/06/11 v1.0.4 Linked ORCiD logo macro package
```

```
(c:/TeXLive/2022/texmf-dist/tex/latex/pgf/frontendlayer/tikz.sty
(c:/TeXLive/20
22/texmf-dist/tex/latex/pgf/basiclayer/pgf.sty (c:/TeXLive/2022/texmf-
dist/tex/
latex/pgf/utilities/pgfrcs.sty (c:/TeXLive/2022/texmf-
dist/tex/generic/pgf/util
ities/pgfutil-common.tex
\pgfutil@everybye=\toks49
\pgfutil@tempdima=\dimen280
\pgfutil@tempdimb=\dimen281
) (c:/TeXLive/2022/texmf-dist/tex/generic/pgf/utilities/pgfutil-latex.def
\pgfutil@abb=\box77
) (c:/TeXLive/2022/texmf-dist/tex/generic/pgf/utilities/pgfrcs.code.tex
(c:/TeX
Live/2022/texmf-dist/tex/generic/pgf/pgf.revision.tex)
Package: pgfrcs 2023-01-15 v3.1.10 (3.1.10)
))
Package: pgf 2023-01-15 v3.1.10 (3.1.10)
(c:/TeXLive/2022/texmf-dist/tex/latex/pgf/basiclayer/pgfcore.sty
(c:/TeXLive/20
22/texmf-dist/tex/latex/pgf/systemlayer/pgfsys.sty
(c:/TeXLive/2022/texmf-dist/
tex/generic/pgf/systemlayer/pgfsys.code.tex
Package: pgfsys 2023-01-15 v3.1.10 (3.1.10)
(c:/TeXLive/2022/texmf-dist/tex/generic/pgf/utilities/pgfkeys.code.tex
\pgfkeys@pathtoks=\toks50
\pgfkeys@temptoks=\toks51

(c:/TeXLive/2022/texmf-
dist/tex/generic/pgf/utilities/pgfkeyslibraryfiltered.co
de.tex
\pgfkeys@tmptoks=\toks52
))
\pgf@x=\dimen282
\pgf@y=\dimen283
\pgf@xa=\dimen284
\pgf@ya=\dimen285
\pgf@xb=\dimen286
\pgf@yb=\dimen287
\pgf@xc=\dimen288
\pgf@yc=\dimen289
\pgf@xd=\dimen290
\pgf@yd=\dimen291
\w@pgf@writea=\write4
\r@pgf@reada=\read2
\c@pgf@counta=\count339
\c@pgf@countb=\count340
\c@pgf@countc=\count341
\c@pgf@countd=\count342
\t@pgf@toka=\toks53
\t@pgf@tokb=\toks54
\t@pgf@tokc=\toks55
\pgf@sys@id@count=\count343
(c:/TeXLive/2022/texmf-dist/tex/generic/pgf/systemlayer/pgf.cfg
```

```
File: pgf.cfg 2023-01-15 v3.1.10 (3.1.10)
)
Driver file for pgf: pgfsys-pdftex.def
(c:/TeXLive/2022/texmf-dist/tex/generic/pgf/systemlayer/pgfsys-pdftex.def
File: pgfsys-pdftex.def 2023-01-15 v3.1.10 (3.1.10)
(c:/TeXLive/2022/texmf-dist/tex/generic/pgf/systemlayer/pgfsys-common-
pdf.def
File: pgfsys-common-pdf.def 2023-01-15 v3.1.10 (3.1.10)
)))
(c:/TeXLive/2022/texmf-
dist/tex/generic/pgf/systemlayer/pgfsyssoftpath.code.tex
File: pgfsyssoftpath.code.tex 2023-01-15 v3.1.10 (3.1.10)
\pgfsyssoftpath@smallbuffer@items=\count344
\pgfsyssoftpath@bigbuffer@items=\count345
)
(c:/TeXLive/2022/texmf-
dist/tex/generic/pgf/systemlayer/pgfsysprotocol.code.tex
File: pgfsysprotocol.code.tex 2023-01-15 v3.1.10 (3.1.10)
)) (c:/TeXLive/2022/texmf-
dist/tex/generic/pgf/basiclayer/pgfcore.code.tex
Package: pgfcore 2023-01-15 v3.1.10 (3.1.10)
(c:/TeXLive/2022/texmf-dist/tex/generic/pgf/math/pgfmath.code.tex
(c:/TeXLive/2
022/texmf-dist/tex/generic/pgf/math/pgfmathutil.code.tex)
(c:/TeXLive/2022/texm
f-dist/tex/generic/pgf/math/pgfmathparser.code.tex
\pgfmath@dimen=\dimen292
\pgfmath@count=\count346
\pgfmath@box=\box78
\pgfmath@toks=\toks56
\pgfmath@stack@operand=\toks57
\pgfmath@stack@operation=\toks58
) (c:/TeXLive/2022/texmf-
dist/tex/generic/pgf/math/pgfmathfunctions.code.tex)
(c:/TeXLive/2022/texmf-
dist/tex/generic/pgf/math/pgfmathfunctions.basic.code.te
x)
(c:/TeXLive/2022/texmf-
dist/tex/generic/pgf/math/pgfmathfunctions.trigonometric
.code.tex)
(c:/TeXLive/2022/texmf-
dist/tex/generic/pgf/math/pgfmathfunctions.random.code.t
ex)
(c:/TeXLive/2022/texmf-
dist/tex/generic/pgf/math/pgfmathfunctions.comparison.co
de.tex)
(c:/TeXLive/2022/texmf-
dist/tex/generic/pgf/math/pgfmathfunctions.base.code.tex
)
(c:/TeXLive/2022/texmf-
dist/tex/generic/pgf/math/pgfmathfunctions.round.code.te
x)
(c:/TeXLive/2022/texmf-
dist/tex/generic/pgf/math/pgfmathfunctions.misc.code.tex
```

```
)
(c:/TeXLive/2022/texmf-
dist/tex/generic/pgf/math/pgfmathfunctions.integerarithm
etics.code.tex) (c:/TeXLive/2022/texmf-
dist/tex/generic/pgf/math/pgfmathcalc.co
de.tex) (c:/TeXLive/2022/texmf-
dist/tex/generic/pgf/math/pgfmathfloat.code.tex
\c@pgfmathroundto@lastzeros=\count347
)) (c:/TeXLive/2022/texmf-dist/tex/generic/pgf/math/pgfint.code.tex)
(c:/TeXLiv
e/2022/texmf-dist/tex/generic/pgf/basiclayer/pgfcorepoints.code.tex
File: pgfcorepoints.code.tex 2023-01-15 v3.1.10 (3.1.10)
\pgf@picminx=\dimen293
\pgf@picmaxx=\dimen294
\pgf@picminy=\dimen295
\pgf@picmaxy=\dimen296
\pgf@pathminx=\dimen297
\pgf@pathmaxx=\dimen298
\pgf@pathminy=\dimen299
\pgf@pathmaxy=\dimen300
\pgf@xx=\dimen301
\pgf@xy=\dimen302
\pgf@yx=\dimen303
\pgf@yy=\dimen304
\pgf@zx=\dimen305
\pgf@zy=\dimen306
)
(c:/TeXLive/2022/texmf-
dist/tex/generic/pgf/basiclayer/pgfcorepathconstruct.cod
e.tex
File: pgfcorepathconstruct.code.tex 2023-01-15 v3.1.10 (3.1.10)
\pgf@path@lastx=\dimen307
\pgf@path@lasty=\dimen308
)
(c:/TeXLive/2022/texmf-
dist/tex/generic/pgf/basiclayer/pgfcorepathusage.code.te
x
File: pgfcorepathusage.code.tex 2023-01-15 v3.1.10 (3.1.10)
\pgf@shorten@end@additional=\dimen309
\pgf@shorten@start@additional=\dimen310
) (c:/TeXLive/2022/texmf-
dist/tex/generic/pgf/basiclayer/pgfcorescopes.code.tex
File: pgfcorescopes.code.tex 2023-01-15 v3.1.10 (3.1.10)
\pgfpic=\box79
\pgf@hbox=\box80
\pgf@layerbox@main=\box81
\pgf@picture@serial@count=\count348
)
(c:/TeXLive/2022/texmf-
dist/tex/generic/pgf/basiclayer/pgfcoregraphicstate.code
.tex
File: pgfcoregraphicstate.code.tex 2023-01-15 v3.1.10 (3.1.10)
\pgflinewidth=\dimen311
)
```

```
(c:/TeXLive/2022/texmf-
dist/tex/generic/pgf/basiclayer/pgfcoretransformations.c
ode.tex
File: pgfcoretransformations.code.tex 2023-01-15 v3.1.10 (3.1.10)
\pgf@pt@x=\dimen312
\pgf@pt@y=\dimen313
\pgf@pt@temp=\dimen314
) (c:/TeXLive/2022/texmf-
dist/tex/generic/pgf/basiclayer/pgfcorequick.code.tex
File: pgfcorequick.code.tex 2023-01-15 v3.1.10 (3.1.10)
) (c:/TeXLive/2022/texmf-
dist/tex/generic/pgf/basiclayer/pgfcoreobjects.code.te
x
File: pgfcoreobjects.code.tex 2023-01-15 v3.1.10 (3.1.10)
)
(c:/TeXLive/2022/texmf-
dist/tex/generic/pgf/basiclayer/pgfcorepathprocessing.co
de.tex
File: pgfcorepathprocessing.code.tex 2023-01-15 v3.1.10 (3.1.10)
) (c:/TeXLive/2022/texmf-
dist/tex/generic/pgf/basiclayer/pgfcorearrows.code.tex
File: pgfcorearrows.code.tex 2023-01-15 v3.1.10 (3.1.10)
\pgfarrowsep=\dimen315
) (c:/TeXLive/2022/texmf-
dist/tex/generic/pgf/basiclayer/pgfcoreshade.code.tex
File: pgfcoreshade.code.tex 2023-01-15 v3.1.10 (3.1.10)
\pgf@max=\dimen316
\pgf@sys@shading@range@num=\count349
\pgf@shadingcount=\count350
) (c:/TeXLive/2022/texmf-
dist/tex/generic/pgf/basiclayer/pgfcoreimage.code.tex
File: pgfcoreimage.code.tex 2023-01-15 v3.1.10 (3.1.10)
)
(c:/TeXLive/2022/texmf-
dist/tex/generic/pgf/basiclayer/pgfcoreexternal.code.tex
File: pgfcoreexternal.code.tex 2023-01-15 v3.1.10 (3.1.10)
\pgfexternal@startupbox=\box82
) (c:/TeXLive/2022/texmf-
dist/tex/generic/pgf/basiclayer/pgfcorelayers.code.tex
File: pgfcorelayers.code.tex 2023-01-15 v3.1.10 (3.1.10)
)
(c:/TeXLive/2022/texmf-
dist/tex/generic/pgf/basiclayer/pgfcoretransparency.code
.tex
File: pgfcoretransparency.code.tex 2023-01-15 v3.1.10 (3.1.10)
)
(c:/TeXLive/2022/texmf-
dist/tex/generic/pgf/basiclayer/pgfcorepatterns.code.tex
File: pgfcorepatterns.code.tex 2023-01-15 v3.1.10 (3.1.10)
) (c:/TeXLive/2022/texmf-
dist/tex/generic/pgf/basiclayer/pgfcorerdf.code.tex
File: pgfcorerdf.code.tex 2023-01-15 v3.1.10 (3.1.10)
))) (c:/TeXLive/2022/texmf-
dist/tex/generic/pgf/modules/pgfmoduleshapes.code.te
```

```
x
File: pgfmoduleshapes.code.tex 2023-01-15 v3.1.10 (3.1.10)
\pgfnodeparttextbox=\box83
) (c:/TeXLive/2022/texmf-
dist/tex/generic/pgf/modules/pgfmoduleplot.code.tex
File: pgfmoduleplot.code.tex 2023-01-15 v3.1.10 (3.1.10)
)
(c:/TeXLive/2022/texmf-dist/tex/latex/pgf/compatibility/pgfcomp-version-
0-65.st
y
Package: pgfcomp-version-0-65 2023-01-15 v3.1.10 (3.1.10)
\pgf@nodesepstart=\dimen317
\pgf@nodesepend=\dimen318
)
(c:/TeXLive/2022/texmf-dist/tex/latex/pgf/compatibility/pgfcomp-version-
1-18.st
y
Package: pgfcomp-version-1-18 2023-01-15 v3.1.10 (3.1.10)
)) (c:/TeXLive/2022/texmf-dist/tex/latex/pgf/utilities/pgffor.sty
(c:/TeXLive/2
022/texmf-dist/tex/latex/pgf/utilities/pgfkeys.sty
(c:/TeXLive/2022/texmf-dist/
tex/generic/pgf/utilities/pgfkeys.code.tex)) (c:/TeXLive/2022/texmf-
dist/tex/la
tex/pgf/math/pgfmath.sty (c:/TeXLive/2022/texmf-
dist/tex/generic/pgf/math/pgfma
th.code.tex)) (c:/TeXLive/2022/texmf-
dist/tex/generic/pgf/utilities/pgffor.code
.tex
Package: pgffor 2023-01-15 v3.1.10 (3.1.10)
\pgffor@iter=\dimen319
\pgffor@skip=\dimen320
\pgffor@stack=\toks59
\pgffor@toks=\toks60
)) (c:/TeXLive/2022/texmf-
dist/tex/generic/pgf/frontendlayer/tikz/tikz.code.tex
Package: tikz 2023-01-15 v3.1.10 (3.1.10)

(c:/TeXLive/2022/texmf-
dist/tex/generic/pgf/libraries/pgflibraryplothandlers.co
de.tex
File: pgflibraryplothandlers.code.tex 2023-01-15 v3.1.10 (3.1.10)
\pgf@plot@mark@count=\count351
\pgfplotmarksize=\dimen321
)
\tikz@lastx=\dimen322
\tikz@lasty=\dimen323
\tikz@lastxsaved=\dimen324
\tikz@lastysaved=\dimen325
\tikz@lastmovetox=\dimen326
\tikz@lastmovetoy=\dimen327
\tikzleveldistance=\dimen328
\tikzsiblingdistance=\dimen329
\tikz@figbox=\box84
```

```
\tikz@figbox@bg=\box85
\tikz@tempbox=\box86
\tikz@tempbox@bg=\box87
\tikztreelevel=\count352
\tikznumberofchildren=\count353
\tikznumberofcurrentchild=\count354
\tikz@fig@count=\count355
(c:/TeXLive/2022/texmf-
dist/tex/generic/pgf/modules/pgfmodulematrix.code.tex
File: pgfmodulematrix.code.tex 2023-01-15 v3.1.10 (3.1.10)
\pgfmatrixcurrentrow=\count356
\pgfmatrixcurrentcolumn=\count357
\pgf@matrix@numberofcolumns=\count358
)
\tikz@expandcount=\count359

(c:/TeXLive/2022/texmf-
dist/tex/generic/pgf/frontendlayer/tikz/libraries/tikzli
brarytopaths.code.tex
File: tikzlibrarytopaths.code.tex 2023-01-15 v3.1.10 (3.1.10)
)))
(c:/TeXLive/2022/texmf-
dist/tex/generic/pgf/frontendlayer/tikz/libraries/tikzli
brarysvg.path.code.tex
File: tikzlibrarysvg.path.code.tex 2023-01-15 v3.1.10 (3.1.10)

(c:/TeXLive/2022/texmf-
dist/tex/generic/pgf/libraries/pgflibrarysvg.path.code.t
ex
File: pgflibrarysvg.path.code.tex 2023-01-15 v3.1.10 (3.1.10)
(c:/TeXLive/2022/texmf-
dist/tex/generic/pgf/modules/pgfmoduleparser.code.tex
File: pgfmoduleparser.code.tex 2023-01-15 v3.1.10 (3.1.10)
\pgfparserdef@arg@count=\count360
)
\pgf@lib@svg@last@x=\dimen330
\pgf@lib@svg@last@y=\dimen331
\pgf@lib@svg@last@c@x=\dimen332
\pgf@lib@svg@last@c@y=\dimen333
\pgf@lib@svg@count=\count361
\pgf@lib@svg@max@num=\count362
))
\@curXheight=\skip167
)
Package translations Info: No language package found. I am going to use
`englis
h' as default language. on input line 68.
LaTeX Font Info:    Trying to load font information for T1+Merriwthr-OsF
on inp
ut line 68.
(c:/TeXLive/2022/texmf-dist/tex/latex/merriweather/T1Merriwthr-OsF.fd
File: T1Merriwthr-OsF.fd 2020/08/30 (autoinst) Font definitions for
T1/Merriwth
r-OsF.
```

```
)
LaTeX Font Info:    Font shape `T1/Merriwthr-OsF/m/n' will be
(Font)              scaled to size 7.5pt on input line 68.
(./main_manuscript.aux)
\openout1 = `main_manuscript.aux'.

LaTeX Font Info:    Checking defaults for OML/cmm/m/it on input line 68.
LaTeX Font Info:    ... okay on input line 68.
LaTeX Font Info:    Checking defaults for OMS/cmsy/m/n on input line 68.
LaTeX Font Info:    ... okay on input line 68.
LaTeX Font Info:    Checking defaults for OT1/cmr/m/n on input line 68.
LaTeX Font Info:    ... okay on input line 68.
LaTeX Font Info:    Checking defaults for T1/cmr/m/n on input line 68.
LaTeX Font Info:    ... okay on input line 68.
LaTeX Font Info:    Checking defaults for TS1/cmr/m/n on input line 68.
LaTeX Font Info:    ... okay on input line 68.
LaTeX Font Info:    Checking defaults for OMX/cmex/m/n on input line 68.
LaTeX Font Info:    ... okay on input line 68.
LaTeX Font Info:    Checking defaults for U/cmr/m/n on input line 68.
LaTeX Font Info:    ... okay on input line 68.
LaTeX Font Info:    Checking defaults for PD1/pdf/m/n on input line 68.
LaTeX Font Info:    ... okay on input line 68.
LaTeX Font Info:    Checking defaults for PU/pdf/m/n on input line 68.
LaTeX Font Info:    ... okay on input line 68.
LaTeX Info: Redefining \microtypecontext on input line 68.
Package microtype Info: Applying patch `item' on input line 68.
Package microtype Info: Applying patch `toc' on input line 68.
Package microtype Info: Applying patch `eqnum' on input line 68.

Package microtype Warning: Unable to apply patch `footnote' on input line
68.

Package microtype Info: Applying patch `verbatim' on input line 68.
Package microtype Info: Generating PDF output.
Package microtype Info: Character protrusion enabled (level 2).
Package microtype Info: Using default protrusion set `alltext'.
Package microtype Info: Automatic font expansion enabled (level 2),
(microtype)             stretch: 20, shrink: 20, step: 1, non-selected.
Package microtype Info: Using default expansion set `alltext-nott'.
LaTeX Info: Redefining \showhyphens on input line 68.
Package microtype Info: No adjustment of tracking.
Package microtype Info: No adjustment of interword spacing.
Package microtype Info: No adjustment of character kerning.
Package microtype Info: Loading generic protrusion settings for font
family
(microtype)             `Merriwthr-OsF' (encoding: T1).
(microtype)             For optimal results, create family-specific
settings.
(microtype)             See the microtype manual for details.
LaTeX Font Info:    Redeclaring symbol font `operators' on input line 68.
LaTeX Font Info:    Encoding `OT1' has changed to `T1' for symbol font
(Font)              `operators' in the math version `normal' on input
line 68.
```

```
LaTeX Font Info:    Overwriting symbol font `operators' in version
`normal'
(Font)                  OT1/cmr/m/n --> T1/Merriwthr-OsF/m/up on input
line 68.

LaTeX Font Info:    Encoding `OT1' has changed to `T1' for symbol font
(Font)              `operators' in the math version `bold' on input line
68.
LaTeX Font Info:    Overwriting symbol font `operators' in version `bold'
(Font)                  OT1/cmr/bx/n --> T1/Merriwthr-OsF/m/up on input
line 68
.
LaTeX Font Info:    Overwriting symbol font `operators' in version `bold'
(Font)                  T1/Merriwthr-OsF/m/up --> T1/Merriwthr-OsF/b/up
on inpu
t line 68.
LaTeX Font Info:    Redeclaring math alphabet \mathbf on input line 68.
LaTeX Font Info:    Overwriting math alphabet `\mathbf' in version
`normal'
(Font)                  OT1/cmr/bx/n --> T1/Merriwthr-OsF/b/up on input
line 68
.
LaTeX Font Info:    Overwriting math alphabet `\mathbf' in version `bold'
(Font)                  OT1/cmr/bx/n --> T1/Merriwthr-OsF/b/up on input
line 68
.
LaTeX Font Info:    Redeclaring math alphabet \mathsf on input line 68.
LaTeX Font Info:    Overwriting math alphabet `\mathsf' in version
`normal'
(Font)                  OT1/cmss/m/n --> T1/MerriwthrSans-OsF/m/up on
input lin
e 68.
LaTeX Font Info:    Overwriting math alphabet `\mathsf' in version `bold'
(Font)                  OT1/cmss/bx/n --> T1/MerriwthrSans-OsF/m/up on
input li
ne 68.
LaTeX Font Info:    Redeclaring math alphabet \mathit on input line 68.
LaTeX Font Info:    Overwriting math alphabet `\mathit' in version
`normal'
(Font)                  OT1/cmr/m/it --> T1/Merriwthr-OsF/m/it on input
line 68
.
LaTeX Font Info:    Overwriting math alphabet `\mathit' in version `bold'
(Font)                  OT1/cmr/bx/it --> T1/Merriwthr-OsF/m/it on input
line 6
8.
LaTeX Font Info:    Redeclaring math alphabet \mathtt on input line 68.
LaTeX Font Info:    Overwriting math alphabet `\mathtt' in version
`normal'
(Font)                  OT1/cmtt/m/n --> T1/lmtt/m/up on input line 68.
LaTeX Font Info:    Overwriting math alphabet `\mathtt' in version `bold'
(Font)                  OT1/cmtt/m/n --> T1/lmtt/m/up on input line 68.
LaTeX Font Info:    Overwriting math alphabet `\mathsf' in version `bold'
```

```
(Font)                  T1/MerriwthrSans-OsF/m/up --> T1/MerriwthrSans-
OsF/b/up
 on input line 68.
LaTeX Font Info:    Overwriting math alphabet `\mathit' in version `bold'
(Font)                  T1/Merriwthr-OsF/m/it --> T1/Merriwthr-OsF/b/it
on inpu
t line 68.
\c@mv@tabular=\count363
\c@mv@boldtabular=\count364
Package mathastext Info: current meaning of amsmath \resetMathstrut@
saved on i
nput line 68.
(c:/TeXLive/2022/texmf-dist/tex/context/base/mkii/supp-pdf.mkii
[Loading MPS to PDF converter (version 2006.09.02).]
\scratchcounter=\count365
\scratchdimen=\dimen334
\scratchbox=\box88
\nofMPsegments=\count366
\nofMParguments=\count367
\everyMPshowfont=\toks61
\MPscratchCnt=\count368
\MPscratchDim=\dimen335
\MPnumerator=\count369
\makeMPintoPDFobject=\count370
\everyMPtoPDFconversion=\toks62
) (c:/TeXLive/2022/texmf-dist/tex/latex/epstopdf-pkg/epstopdf-base.sty
Package: epstopdf-base 2020-01-24 v2.11 Base part for package epstopdf
Package epstopdf-base Info: Redefining graphics rule for `.eps' on input
line 4
85.
(c:/TeXLive/2022/texmf-dist/tex/latex/latexconfig/epstopdf-sys.cfg
File: epstopdf-sys.cfg 2010/07/13 v1.3 Configuration of (r)epstopdf for
TeX Liv
e
))
*geometry* driver: auto-detecting
*geometry* detected driver: pdftex
*geometry* verbose mode - [ preamble ] result:
* driver: pdftex
* paper: a4paper
* layout: <same size as paper>
* layoutoffset:(h,v)=(0.0pt,0.0pt)
* modes: includefoot twoside
* h-part:(L,W,R)=(54.64pt, 488.22787pt, 54.64pt)
* v-part:(T,H,B)=(66.0pt, 745.04684pt, 34.0pt)
* \paperwidth=597.50787pt
* \paperheight=845.04684pt
* \textwidth=488.22787pt
* \textheight=715.04684pt
* \oddsidemargin=-17.62999pt
* \evensidemargin=-17.62999pt
* \topmargin=-47.76999pt
* \headheight=17.5pt
* \headsep=24.0pt
```

```
* \topskip=10.0pt
* \footskip=30.0pt
* \marginparwidth=48.0pt
* \marginparsep=10.0pt
* \columnsep=18.0pt
* \skip\footins=22.0pt plus 2.0pt
* \hoffset=0.0pt
* \voffset=0.0pt
* \mag=1000
* \@twocolumntrue
* \@twosidetrue
* \@mparswitchtrue
* \@reversemarginfalse
* (1in=72.27pt=25.4mm, 1cm=28.453pt)

Package hyperref Info: Link coloring ON on input line 68.
(./main_manuscript.out) (./main_manuscript.out)
\@outlinefile=\write5
\openout5 = `main_manuscript.out'.

\@gscitedetails=\box89
\@gscitedetailsheight=\skip168
\@gsheadbox=\box90
\@gsheadboxheight=\skip169
LaTeX Font Info:    Font shape `T1/Merriwthr-OsF/b/n' will be
(Font)              scaled to size 6.5pt on input line 68.
LaTeX Font Info:    Calculating math sizes for size <7.5> on input line
68.

LaTeX Font Warning: Font shape `T1/Merriwthr-OsF/m/up' undefined
(Font)              using `T1/Merriwthr-OsF/m/n' instead on input line
68.

LaTeX Font Info:    Font shape `T1/Merriwthr-OsF/m/up' will be
(Font)              scaled to size 6.24973pt on input line 68.
LaTeX Font Info:    Font shape `T1/Merriwthr-OsF/m/up' will be
(Font)              scaled to size 5.24997pt on input line 68.
LaTeX Font Info:    Trying to load font information for U+eur on input
line 68.

(c:/TeXLive/2022/texmf-dist/tex/latex/amsfonts/ueur.fd
File: ueur.fd 2013/01/14 v3.01 Euler Roman
) (c:/TeXLive/2022/texmf-dist/tex/latex/microtype/mt-eur.cfg
File: mt-eur.cfg 2006/07/31 v1.1 microtype config. file: AMS Euler Roman
(RS)
)

LaTeX Font Warning: Font shape `OMS/cmsy/m/n' in size <7.5> not available
(Font)              size <7> substituted on input line 68.

LaTeX Font Info:    Trying to load font information for U+euf on input
line 68.

(c:/TeXLive/2022/texmf-dist/tex/latex/amsfonts/ueuf.fd
```

```
File: ueuf.fd 2013/01/14 v3.01 Euler Fraktur
) (c:/TeXLive/2022/texmf-dist/tex/latex/microtype/mt-euf.cfg
File: mt-euf.cfg 2006/07/03 v1.1 microtype config. file: AMS Euler
Fraktur (RS)

)
LaTeX Font Info:    Trying to load font information for U+eus on input
line 68.

(c:/TeXLive/2022/texmf-dist/tex/latex/amsfonts/ueus.fd
File: ueus.fd 2013/01/14 v3.01 Euler Script
) (c:/TeXLive/2022/texmf-dist/tex/latex/microtype/mt-eus.cfg
File: mt-eus.cfg 2006/07/28 v1.2 microtype config. file: AMS Euler Script
(RS)
)
LaTeX Font Info:    Trying to load font information for U+euex on input
line 68
.
(c:/TeXLive/2022/texmf-dist/tex/latex/amsfonts/ueuex.fd
File: ueuex.fd 2013/01/14 v3.01 Euler extra symbols
)

LaTeX Font Warning: Font shape `OML/cmm/m/it' in size <7.5> not available
(Font)              size <7> substituted on input line 68.

LaTeX Font Info:    Font shape `T1/Merriwthr-OsF/m/n' will be
(Font)              scaled to size 6.24973pt on input line 68.
LaTeX Font Info:    Font shape `T1/Merriwthr-OsF/m/n' will be
(Font)              scaled to size 5.24997pt on input line 68.
LaTeX Font Info:    Font shape `T1/Merriwthr-OsF/m/it' will be
(Font)              scaled to size 7.5pt on input line 68.
LaTeX Font Info:    Font shape `T1/Merriwthr-OsF/m/it' will be
(Font)              scaled to size 6.24973pt on input line 68.
LaTeX Font Info:    Font shape `T1/Merriwthr-OsF/m/it' will be
(Font)              scaled to size 5.24997pt on input line 68.
LaTeX Font Info:    Font shape `T1/Merriwthr-OsF/m/n' will be
(Font)              scaled to size 8.0pt on input line 68.
LaTeX Font Info:    Font shape `T1/Merriwthr-OsF/m/it' will be
(Font)              scaled to size 8.0pt on input line 68.
LaTeX Font Info:    Font shape `T1/Merriwthr-OsF/b/it' will be
(Font)              scaled to size 8.0pt on input line 68.
Package caption Info: Begin \AtBeginDocument code.
Package caption Info: End \AtBeginDocument code.

(c:/TeXLive/2022/texmf-dist/tex/latex/translations/translations-basic-
dictionar
y-english.trsl
File: translations-basic-dictionary-english.trsl (english translation
file `tra
nslations-basic-dictionary')
)
Package translations Info: loading dictionary `translations-basic-
dictionary' f
or `english'. on input line 68.
```

```
TextBlockOrigin set to 4pc+6.64pt x 4pc+6pt
<oup.pdf, id=116, 49.18375pt x 48.18pt>
File: oup.pdf Graphic file (type pdf)
<use oup.pdf>
Package pdftex.def Info: oup.pdf  used on input line 86.
(pdftex.def)             Requested size: 39.90764pt x 39.09323pt.
<gigascience-logo.pdf, id=117, 99.37125pt x 33.12375pt>
File: gigascience-logo.pdf Graphic file (type pdf)
<use gigascience-logo.pdf>
Package pdftex.def Info: gigascience-logo.pdf  used on input line 86.
(pdftex.def)             Requested size: 69.16667pt x 23.05524pt.

Overfull \hbox (54.64pt too wide) in paragraph at lines 86--86
[][]
 []

LaTeX Font Info:    Font shape `T1/Merriwthr-OsF/m/n' will be
(Font)              scaled to size 14.0pt on input line 86.
LaTeX Font Info:    Font shape `T1/Merriwthr-OsF/m/n' will be
(Font)              scaled to size 8.99997pt on input line 86.
LaTeX Font Info:    Calculating math sizes for size <14> on input line
86.
LaTeX Font Info:    Font shape `T1/Merriwthr-OsF/m/up' will be
(Font)              scaled to size 14.0pt on input line 86.
LaTeX Font Info:    Font shape `T1/Merriwthr-OsF/m/up' will be
(Font)              scaled to size 11.66617pt on input line 86.
LaTeX Font Info:    Font shape `T1/Merriwthr-OsF/m/up' will be
(Font)              scaled to size 9.79996pt on input line 86.
LaTeX Font Info:    Font shape `T1/Merriwthr-OsF/m/n' will be
(Font)              scaled to size 11.66617pt on input line 86.
LaTeX Font Info:    Font shape `T1/Merriwthr-OsF/m/n' will be
(Font)              scaled to size 9.79996pt on input line 86.
LaTeX Font Info:    Font shape `T1/Merriwthr-OsF/m/it' will be
(Font)              scaled to size 14.0pt on input line 86.
LaTeX Font Info:    Font shape `T1/Merriwthr-OsF/m/it' will be
(Font)              scaled to size 11.66617pt on input line 86.
LaTeX Font Info:    Font shape `T1/Merriwthr-OsF/m/it' will be
(Font)              scaled to size 9.79996pt on input line 86.
LaTeX Font Info:    Font shape `T1/Merriwthr-OsF/b/n' will be
(Font)              scaled to size 18.0pt on input line 86.
LaTeX Font Info:    Font shape `T1/Merriwthr-OsF/m/n' will be
(Font)              scaled to size 13.0pt on input line 86.
LaTeX Font Info:    Calculating math sizes for size <13> on input line
86.
LaTeX Font Info:    Font shape `T1/Merriwthr-OsF/m/up' will be
(Font)              scaled to size 13.0pt on input line 86.
LaTeX Font Info:    Font shape `T1/Merriwthr-OsF/m/up' will be
(Font)              scaled to size 10.83287pt on input line 86.
LaTeX Font Info:    Font shape `T1/Merriwthr-OsF/m/up' will be
(Font)              scaled to size 9.09996pt on input line 86.

LaTeX Font Warning: Font shape `OMS/cmsy/m/n' in size <13> not available
(Font)              size <12> substituted on input line 86.
```

```
LaTeX Font Warning: Font shape `OMX/cmex/m/n' in size <13> not available
(Font)              size <12> substituted on input line 86.


LaTeX Font Warning: Font shape `OML/cmm/m/it' in size <13> not available
(Font)              size <12> substituted on input line 86.

LaTeX Font Info:    Font shape `T1/Merriwthr-OsF/m/n' will be
(Font)              scaled to size 10.83287pt on input line 86.
LaTeX Font Info:    Font shape `T1/Merriwthr-OsF/m/n' will be
(Font)              scaled to size 9.09996pt on input line 86.
LaTeX Font Info:    Font shape `T1/Merriwthr-OsF/m/it' will be
(Font)              scaled to size 13.0pt on input line 86.
LaTeX Font Info:    Font shape `T1/Merriwthr-OsF/m/it' will be
(Font)              scaled to size 10.83287pt on input line 86.
LaTeX Font Info:    Font shape `T1/Merriwthr-OsF/m/it' will be
(Font)              scaled to size 9.09996pt on input line 86.
LaTeX Font Info:    Trying to load font information for TS1+Merriwthr-OsF
on in
put line 86.
(c:/TeXLive/2022/texmf-dist/tex/latex/merriweather/TS1Merriwthr-OsF.fd
File: TS1Merriwthr-OsF.fd 2020/08/30 (autoinst) Font definitions for
TS1/Merriw
thr-OsF.
)
LaTeX Font Info:    Font shape `TS1/Merriwthr-OsF/m/n' will be
(Font)              scaled to size 10.83287pt on input line 86.
Package microtype Info: Loading generic protrusion settings for font
family
(microtype)             `Merriwthr-OsF' (encoding: TS1).
(microtype)             For optimal results, create family-specific
settings.
(microtype)             See the microtype manual for details.
LaTeX Font Info:    Font shape `T1/Merriwthr-OsF/m/n' will be
(Font)              scaled to size 9.0pt on input line 86.
LaTeX Font Info:    Font shape `T1/Merriwthr-OsF/m/up' will be
(Font)              scaled to size 9.0pt on input line 86.
LaTeX Font Info:    Font shape `T1/Merriwthr-OsF/m/up' will be
(Font)              scaled to size 7.0pt on input line 86.
LaTeX Font Info:    Font shape `T1/Merriwthr-OsF/m/up' will be
(Font)              scaled to size 5.0pt on input line 86.
LaTeX Font Info:    Font shape `T1/Merriwthr-OsF/m/n' will be
(Font)              scaled to size 7.0pt on input line 86.
LaTeX Font Info:    Font shape `T1/Merriwthr-OsF/m/n' will be
(Font)              scaled to size 5.0pt on input line 86.
LaTeX Font Info:    Font shape `T1/Merriwthr-OsF/m/it' will be
(Font)              scaled to size 9.0pt on input line 86.
LaTeX Font Info:    Font shape `T1/Merriwthr-OsF/m/it' will be
(Font)              scaled to size 7.0pt on input line 86.
LaTeX Font Info:    Font shape `T1/Merriwthr-OsF/m/it' will be
(Font)              scaled to size 5.0pt on input line 86.
LaTeX Font Info:    Font shape `T1/Merriwthr-OsF/m/n' will be
(Font)              scaled to size 6.5pt on input line 86.
```

```
LaTeX Font Info:    Calculating math sizes for size <6.5> on input line
86.
LaTeX Font Info:    Font shape `T1/Merriwthr-OsF/m/up' will be
(Font)              scaled to size 6.5pt on input line 86.
LaTeX Font Info:    Font shape `T1/Merriwthr-OsF/m/up' will be
(Font)              scaled to size 5.41643pt on input line 86.
LaTeX Font Info:    Font shape `T1/Merriwthr-OsF/m/up' will be
(Font)              scaled to size 4.54997pt on input line 86.

LaTeX Font Warning: Font shape `OMS/cmsy/m/n' in size <6.5> not available
(Font)              size <6> substituted on input line 86.


LaTeX Font Warning: Font shape `OMS/cmsy/m/n' in size <5.41643> not
available
(Font)              size <5> substituted on input line 86.


LaTeX Font Warning: Font shape `OMS/cmsy/m/n' in size <4.54997> not
available
(Font)              size <5> substituted on input line 86.


LaTeX Font Warning: Font shape `OML/cmm/m/it' in size <6.5> not available
(Font)              size <6> substituted on input line 86.


LaTeX Font Warning: Font shape `OML/cmm/m/it' in size <5.41643> not
available
(Font)              size <5> substituted on input line 86.


LaTeX Font Warning: Font shape `OML/cmm/m/it' in size <4.54997> not
available
(Font)              size <5> substituted on input line 86.

LaTeX Font Info:    Font shape `T1/Merriwthr-OsF/m/n' will be
(Font)              scaled to size 5.41643pt on input line 86.
LaTeX Font Info:    Font shape `T1/Merriwthr-OsF/m/n' will be
(Font)              scaled to size 4.54997pt on input line 86.
LaTeX Font Info:    Font shape `T1/Merriwthr-OsF/m/it' will be
(Font)              scaled to size 6.5pt on input line 86.
LaTeX Font Info:    Font shape `T1/Merriwthr-OsF/m/it' will be
(Font)              scaled to size 5.41643pt on input line 86.
LaTeX Font Info:    Font shape `T1/Merriwthr-OsF/m/it' will be
(Font)              scaled to size 4.54997pt on input line 86.
LaTeX Font Info:    Font shape `TS1/Merriwthr-OsF/m/n' will be
(Font)              scaled to size 5.41643pt on input line 86.

Overfull \hbox (54.64pt too wide) in paragraph at lines 86--86
[][][]
 []

LaTeX Font Info:    Font shape `T1/Merriwthr-OsF/b/n' will be
```

```
(Font)              scaled to size 10.0pt on input line 86.
LaTeX Font Info:    Font shape `T1/Merriwthr-OsF/b/n' will be
(Font)              scaled to size 7.5pt on input line 86.
LaTeX Font Info:    Font shape `T1/Merriwthr-OsF/b/sl' in size <7.5> not
availa
ble
(Font)              Font shape `T1/Merriwthr-OsF/b/it' tried instead on
input l
ine 86.
LaTeX Font Info:    Font shape `T1/Merriwthr-OsF/b/it' will be
(Font)              scaled to size 7.5pt on input line 86.
LaTeX Font Info:    Font shape `T1/Merriwthr-OsF/b/n' will be
(Font)              scaled to size 8.0pt on input line 86.

Overfull \hbox (54.64pt too wide) in paragraph at lines 86--86
[][][]
 []


LaTeX Warning: Optional argument of \twocolumn too tall on page 1.


Underfull \vbox (badness 10000) has occurred while \output is active []


Underfull \vbox (badness 10000) has occurred while \output is active []

LaTeX Font Info:    Font shape `T1/Merriwthr-OsF/m/n' will be
(Font)              scaled to size 7.8pt on input line 86.
LaTeX Font Info:    Font shape `T1/Merriwthr-OsF/b/n' will be
(Font)              scaled to size 7.8pt on input line 86.
[1{c:/TeXLive/2022/texmf-var/fonts/map/pdftex/updmap/pdftex.map}


 <./oup.pdf> <./gigascience-logo.pdf>]

! Package siunitx Error: Found prefix part with no unit.

For immediate help type H <return>.
 ...

l.92 ...al microscopes (approx.~250{\rm\, \si\nano
                                                  m} for high-end
systems w...


Each prefix part must be associated with a unit: a prefix part was found
but
no following unit was given.


LaTeX Font Info:    Font shape `T1/Merriwthr-OsF/m/up' will be
(Font)              scaled to size 7.5pt on input line 92.
```

```
Package natbib Warning: Citation `hirvonen2009structured' on page 2
undefined o
n input line 93.


Package natbib Warning: Citation `hell20152015' on page 2 undefined on
input li
ne 93.


Package natbib Warning: Citation `heintzmann2017super' on page 2
undefined on i
nput line 93.


Package natbib Warning: Citation `demmerle2017strategic' on page 2
undefined on
 input line 93.


Package natbib Warning: Citation `schermelleh2019super' on page 2
undefined on
input line 93.


Package natbib Warning: Citation `gustafsson2000surpassing' on page 2
undefined
 on input line 93.


Package natbib Warning: Citation `muller2016open' on page 2 undefined on
input
line 93.


Package natbib Warning: Citation `lal2016structured' on page 2 undefined
on inp
ut line 93.


Package natbib Warning: Citation `brown2021multicolor' on page 2
undefined on i
nput line 93.


Package natbib Warning: Citation `strohl2016frontiers' on page 2
undefined on i
nput line 94.


Package natbib Warning: Citation `zheng2021current' on page 2 undefined
on inpu
t line 94.
```

```
Package natbib Warning: Citation `huang2018fast' on page 2 undefined on input l
ine 94.


Package natbib Warning: Citation `hoffman2020tiled' on page 2 undefined on inpu
t line 96.


Package natbib Warning: Citation `smith2021structured' on page 2 undefined on i
nput line 96.


Package natbib Warning: Citation `gustafsson2008three' on page 2 undefined on i
nput line 97.


Package natbib Warning: Citation `shah2022impact' on page 2 undefined on input
line 99.


Package natbib Warning: Citation `jin2020deep' on page 2 undefined on input lin
e 99.


Package natbib Warning: Citation `chen2021accelerated' on page 2 undefined on i
nput line 99.


Package natbib Warning: Citation `shah2021deep' on page 2 undefined on input li
ne 99.


Package natbib Warning: Citation `belthangady2019applications' on page 2 undefi
ned on input line 99.


Package natbib Warning: Citation `qiao2021evaluation' on page 2 undefined on in
put line 99.


Package natbib Warning: Citation `xypakis2022deep' on page 2 undefined on input
 line 99.
```

Package natbib Warning: Citation `liu2023improving' on page 2 undefined on inpu
t line 99.

Package natbib Warning: Citation `qiao20213d' on page 2 undefined on input line
 99.

Package natbib Warning: Citation `vaswani2017attention' on page 2 undefined on
input line 101.

Package natbib Warning: Citation `sutskever2014sequence' on page 2 undefined on
 input line 101.

Package natbib Warning: Citation `dehghaniuniversal' on page 2 undefined on inp
ut line 101.

Package natbib Warning: Citation `vyas2020fast' on page 2 undefined on input li
ne 101.

Package natbib Warning: Citation `wang2021position' on page 2 undefined on inpu
t line 101.

Package natbib Warning: Citation `cheng2022mltr' on page 2 undefined on input l
ine 101.

Package natbib Warning: Citation `carion2020end' on page 2 undefined on input l
ine 101.

Package natbib Warning: Citation `sun2021rethinking' on page 2 undefined on inp
ut line 101.

Package natbib Warning: Citation `chen2021pre' on page 2 undefined on input lin

```
e 101.


Package natbib Warning: Citation `ali2023vision' on page 2 undefined on
input l
ine 101.


Package natbib Warning: Citation `liu2021swin' on page 2 undefined on
input lin
e 101.


Package natbib Warning: Citation `liang2021swinir' on page 2 undefined on
input
 line 101.


Package natbib Warning: Citation `zhang2019poisson' on page 2 undefined
on inpu
t line 103.


Package natbib Warning: Citation `zhou2020w2s' on page 2 undefined on
input lin
e 103.


Package natbib Warning: Citation `qiao2021evaluation' on page 2 undefined
on in
put line 103.


Package natbib Warning: Citation `hagen2021fluorescence' on page 2
undefined on
 input line 105.


Underfull \hbox (badness 1014) in paragraph at lines 103--106
\T1/Merriwthr-OsF/m/up/7.5 (+20) purely for de-nois-ing tasks with-out
pro-vid-
ing high-resolution
 []


LaTeX Warning: File `Figures/Figure1.eps' not found on input line 109.

Package epstopdf Info: Source file: <Figures/Figure1.eps>
(epstopdf)             Output file: <Figures/Figure1-eps-converted-
to.pdf>
(epstopdf)             Command: <repstopdf --outfile=Figures/Figure1-eps-
conver
ted-to.pdf Figures/Figure1.eps>
(epstopdf)             \includegraphics on input line 109.
```

```
Package epstopdf Info: Output file is already uptodate.

! Package pdftex.def Error: File `Figures/Figure1-eps-converted-to.pdf'
not fou
nd: using draft setting.

See the pdftex.def package documentation for explanation.
Type  H <return>  for immediate help.
 ...

l.109 ...width=1.0\linewidth]{Figures/Figure1.eps}

Try typing  <return>  to proceed.
If that doesn't work, type  X <return>  to quit.

LaTeX Font Info:    Trying to load font information for T1+lmtt on input
line 1
09.
(c:/TeXLive/2022/texmf-dist/tex/latex/lm/t1lmtt.fd
File: t1lmtt.fd 2015/05/01 v1.6.1 Font defs for Latin Modern
)
Package microtype Info: Loading generic protrusion settings for font
family
(microtype)             `lmtt' (encoding: T1).
(microtype)             For optimal results, create family-specific
settings.
(microtype)             See the microtype manual for details.
LaTeX Font Info:    Font shape `T1/Merriwthr-OsF/m/n' will be
(Font)              scaled to size 6.0pt on input line 110.
LaTeX Font Info:    Font shape `T1/Merriwthr-OsF/b/n' will be
(Font)              scaled to size 6.0pt on input line 110.
LaTeX Font Info:    Font shape `T1/Merriwthr-OsF/m/up' will be
(Font)              scaled to size 6.0pt on input line 110.
LaTeX Font Info:    Font shape `T1/Merriwthr-OsF/m/it' will be
(Font)              scaled to size 6.0pt on input line 110.

LaTeX Warning: `!h' float specifier changed to `!ht'.


Package natbib Warning: Citation `shah2022impact' on page 2 undefined on
input
line 117.


Package natbib Warning: Citation `shah2021deep' on page 2 undefined on
input li
ne 119.

LaTeX Font Info:    Font shape `T1/Merriwthr-OsF/m/it' will be
(Font)              scaled to size 7.8pt on input line 120.
[2]
Underfull \vbox (badness 10000) has occurred while \output is active []
```

```
LaTeX Warning: File `Figures/Figure2.eps' not found on input line 130.

Package epstopdf Info: Source file: <Figures/Figure2.eps>
(epstopdf)             Output file: <Figures/Figure2-eps-converted-
to.pdf>
(epstopdf)             Command: <repstopdf --outfile=Figures/Figure2-eps-
conver
ted-to.pdf Figures/Figure2.eps>
(epstopdf)             \includegraphics on input line 130.
Package epstopdf Info: Output file is already uptodate.

! Package pdftex.def Error: File `Figures/Figure2-eps-converted-to.pdf'
not fou
nd: using draft setting.

See the pdftex.def package documentation for explanation.
Type  H <return>  for immediate help.
 ...

l.130 ...width=1.0\linewidth]{Figures/Figure2.eps}

Try typing  <return>  to proceed.
If that doesn't work, type  X <return>  to quit.


! Package siunitx Error: Found prefix part with no unit.

For immediate help type H <return>.
 ...

l.131 ...les. Scale bar: $4 {\rm\, \si\micro m}$.}


Each prefix part must be associated with a unit: a prefix part was found
but
no following unit was given.


Package textcomp Info: Symbol \textmu not provided by
(textcomp)             font family Merriwthr-OsF in TS1 encoding.
(textcomp)             Default family used instead on input line 131.
(c:/TeXLive/2022/texmf-dist/tex/latex/microtype/mt-cmr.cfg
File: mt-cmr.cfg 2013/05/19 v2.2 microtype config. file: Computer Modern
Roman
(RS)
)

! Package siunitx Error: Found prefix part with no unit.

For immediate help type H <return>.
 ...

l.131 ...les. Scale bar: $4 {\rm\, \si\micro m}$.}
```

```
Each prefix part must be associated with a unit: a prefix part was found
but
no following unit was given.


Package textcomp Info: Symbol \textmu not provided by
(textcomp)             font family Merriwthr-OsF in TS1 encoding.
(textcomp)             Default family used instead on input line 131.

! Package siunitx Error: Found prefix part with no unit.

For immediate help type H <return>.
 ...

l.131 ...les. Scale bar: $4 {\rm\, \si\micro m}$.}


Each prefix part must be associated with a unit: a prefix part was found
but
no following unit was given.


Package textcomp Info: Symbol \textmu not provided by
(textcomp)             font family Merriwthr-OsF in TS1 encoding.
(textcomp)             Default family used instead on input line 131.

! Package siunitx Error: Found prefix part with no unit.

For immediate help type H <return>.
 ...

l.131 ...les. Scale bar: $4 {\rm\, \si\micro m}$.}


Each prefix part must be associated with a unit: a prefix part was found
but
no following unit was given.


Package textcomp Info: Symbol \textmu not provided by
(textcomp)             font family Merriwthr-OsF in TS1 encoding.
(textcomp)             Default family used instead on input line 131.

LaTeX Warning: `!h' float specifier changed to `!ht'.

LaTeX Font Info:    Font shape `T1/Merriwthr-OsF/b/n' will be
(Font)              scaled to size 8.5pt on input line 136.

Package natbib Warning: Citation `gustafsson2008three' on page 3
undefined on i
nput line 140.

Package textcomp Info: Symbol \textmu not provided by
```

```
(textcomp)             font family Merriwthr-OsF in TS1 encoding.
(textcomp)             Default family used instead on input line 141.

LaTeX Font Warning: Font shape `TS1/cmr/m/n' in size <7.5> not available
(Font)              size <7> substituted on input line 141.

Package textcomp Info: Symbol \textmu not provided by
(textcomp)             font family Merriwthr-OsF in TS1 encoding.
(textcomp)             Default family used instead on input line 142.

Underfull \vbox (badness 2538) has occurred while \output is active []

[3]
Underfull \vbox (badness 10000) has occurred while \output is active []


Underfull \vbox (badness 10000) has occurred while \output is active []

[4]

Package natbib Warning: Citation `muller2016open' on page 5 undefined on
input
line 149.


Package natbib Warning: Citation `muller2016open' on page 5 undefined on
input
line 149.


Package natbib Warning: Citation `muller2016open' on page 5 undefined on
input
line 149.


Package natbib Warning: Citation `karras2019successful' on page 5
undefined on
input line 149.

Package textcomp Info: Symbol \textmu not provided by
(textcomp)             font family Merriwthr-OsF in TS1 encoding.
(textcomp)             Default family used instead on input line 149.

Package natbib Warning: Citation `muller2016open' on page 5 undefined on
input
line 149.


Package natbib Warning: Citation `karras2019successful' on page 5
undefined on
input line 149.
```

```
Package natbib Warning: Citation `Mueller-physics' on page 5 undefined on
input
 line 149.

LaTeX Font Info:    Font shape `T1/Merriwthr-OsF/b/n' will be
(Font)              scaled to size 7.0pt on input line 163.

LaTeX Warning: `!h' float specifier changed to `!ht'.


Package natbib Warning: Citation `shah2021deep' on page 5 undefined on
input li
ne 186.


LaTeX Warning: File `Figures/Figure3.eps' not found on input line 190.

Package epstopdf Info: Source file: <Figures/Figure3.eps>
(epstopdf)             Output file: <Figures/Figure3-eps-converted-
to.pdf>
(epstopdf)             Command: <repstopdf --outfile=Figures/Figure3-eps-
conver
ted-to.pdf Figures/Figure3.eps>
(epstopdf)             \includegraphics on input line 190.
Package epstopdf Info: Output file is already uptodate.

! Package pdftex.def Error: File `Figures/Figure3-eps-converted-to.pdf'
not fou
nd: using draft setting.

See the pdftex.def package documentation for explanation.
Type  H <return>  for immediate help.
 ...

l.190 ...width=1.0\linewidth]{Figures/Figure3.eps}

Try typing  <return>  to proceed.
If that doesn't work, type  X <return>  to quit.


! Package siunitx Error: Found prefix part with no unit.

For immediate help type H <return>.
 ...

l.191 ...tio. Scale bar: $4 {\rm\, \si\micro m}$.}


Each prefix part must be associated with a unit: a prefix part was found
but
no following unit was given.


Package textcomp Info: Symbol \textmu not provided by
```

```
(textcomp)             font family Merriwthr-OsF in TS1 encoding.
(textcomp)             Default family used instead on input line 191.

Underfull \hbox (badness 1769) in paragraph at lines 197--198
\T1/Merriwthr-OsF/m/up/7.5 (+20) Dataset $3$ was cre-ated mainly for
joint de-n
ois-ing and super-
 []


Package natbib Warning: Citation `gustafsson2008three' on page 5
undefined on i
nput line 202.

LaTeX Font Info:    Font shape `T1/Merriwthr-OsF/m/n' will be
(Font)              scaled to size 6.25008pt on input line 204.
[5] [6]

LaTeX Warning: File `Figures/Figure4.eps' not found on input line 212.

Package epstopdf Info: Source file: <Figures/Figure4.eps>
(epstopdf)             Output file: <Figures/Figure4-eps-converted-
to.pdf>
(epstopdf)             Command: <repstopdf --outfile=Figures/Figure4-eps-
conver
ted-to.pdf Figures/Figure4.eps>
(epstopdf)             \includegraphics on input line 212.
Package epstopdf Info: Output file is already uptodate.

! Package pdftex.def Error: File `Figures/Figure4-eps-converted-to.pdf'
not fou
nd: using draft setting.

See the pdftex.def package documentation for explanation.
Type  H <return>  for immediate help.
 ...

l.212 ...width=1.0\linewidth]{Figures/Figure4.eps}

Try typing  <return>  to proceed.
If that doesn't work, type  X <return>  to quit.


LaTeX Warning: `!h' float specifier changed to `!ht'.


Package natbib Warning: Citation `zafranshahGit' on page 7 undefined on
input l
ine 222.


Package natbib Warning: Citation `workflowhub_Eu' on page 7 undefined on
input
line 222.
```

```
Package natbib Warning: Citation `zafranshah' on page 7 undefined on
input line
 222.


Package natbib Warning: Citation `dataset_GigaDB' on page 7 undefined on
input
line 222.


Package natbib Warning: Citation `liang2021swinir' on page 7 undefined on
input
 line 229.


Package natbib Warning: Citation `liang2021swinir' on page 7 undefined on
input
 line 229.


Package natbib Warning: Citation `vaswani2017attention' on page 7
undefined on
input line 229.


Package natbib Warning: Citation `liu2021swin' on page 7 undefined on
input lin
e 229.


Package natbib Warning: Citation `liang2021swinir' on page 7 undefined on
input
 line 229.


Package natbib Warning: Citation `liang2021swinir' on page 7 undefined on
input
 line 250.


Package natbib Warning: Citation `shah2022impact' on page 7 undefined on
input
line 253.


Package natbib Warning: Citation `shah2022impact' on page 7 undefined on
input
line 253.


Package natbib Warning: Citation `muller2016open' on page 7 undefined on
input
```

```
line 253.


Package natbib Warning: Citation `shah2022impact' on page 7 undefined on
input
line 253.


Underfull \vbox (badness 1789) has occurred while \output is active []

[7]

LaTeX Warning: File `Figures/Figure5.eps' not found on input line 258.

Package epstopdf Info: Source file: <Figures/Figure5.eps>
(epstopdf)             Output file: <Figures/Figure5-eps-converted-
to.pdf>
(epstopdf)             Command: <repstopdf --outfile=Figures/Figure5-eps-
conver
ted-to.pdf Figures/Figure5.eps>
(epstopdf)             \includegraphics on input line 258.
Package epstopdf Info: Output file is already uptodate.

! Package pdftex.def Error: File `Figures/Figure5-eps-converted-to.pdf'
not fou
nd: using draft setting.

See the pdftex.def package documentation for explanation.
Type  H <return>  for immediate help.
 ...

l.258 ...width=1.0\linewidth]{Figures/Figure5.eps}

Try typing  <return>  to proceed.
If that doesn't work, type  X <return>  to quit.


LaTeX Warning: `!h' float specifier changed to `!ht'.


LaTeX Warning: File `Figures/Figure6.eps' not found on input line 268.

Package epstopdf Info: Source file: <Figures/Figure6.eps>
(epstopdf)             Output file: <Figures/Figure6-eps-converted-
to.pdf>
(epstopdf)             Command: <repstopdf --outfile=Figures/Figure6-eps-
conver
ted-to.pdf Figures/Figure6.eps>
(epstopdf)             \includegraphics on input line 268.
Package epstopdf Info: Output file is already uptodate.

! Package pdftex.def Error: File `Figures/Figure6-eps-converted-to.pdf'
not fou
nd: using draft setting.
```

```
See the pdftex.def package documentation for explanation.
Type  H <return>  for immediate help.
 ...

l.268 ...width=1.0\linewidth]{Figures/Figure6.eps}

Try typing  <return>  to proceed.
If that doesn't work, type  X <return>  to quit.


! Package siunitx Error: Found prefix part with no unit.

For immediate help type H <return>.
 ...

l.270   }


Each prefix part must be associated with a unit: a prefix part was found
but
no following unit was given.


Package textcomp Info: Symbol \textmu not provided by
(textcomp)             font family Merriwthr-OsF in TS1 encoding.
(textcomp)             Default family used instead on input line 270.

LaTeX Warning: File `Figures/Figure7.eps' not found on input line 279.

Package epstopdf Info: Source file: <Figures/Figure7.eps>
(epstopdf)             Output file: <Figures/Figure7-eps-converted-
to.pdf>
(epstopdf)             Command: <repstopdf --outfile=Figures/Figure7-eps-
conver
ted-to.pdf Figures/Figure7.eps>
(epstopdf)             \includegraphics on input line 279.
Package epstopdf Info: Output file is already uptodate.

! Package pdftex.def Error: File `Figures/Figure7-eps-converted-to.pdf'
not fou
nd: using draft setting.

See the pdftex.def package documentation for explanation.
Type  H <return>  for immediate help.
 ...

l.279 ...width=1.0\linewidth]{Figures/Figure7.eps}

Try typing  <return>  to proceed.
If that doesn't work, type  X <return>  to quit.


LaTeX Warning: `!h' float specifier changed to `!ht'.
```

```
Overfull \hbox (13.05385pt too wide) in paragraph at lines 296--318
 []
 []


Package natbib Warning: Citation `ching1995class' on page 8 undefined on input
line 324.


Package natbib Warning: Citation `hussain2018study' on page 8 undefined on inpu
t line 324.


Package natbib Warning: Citation `tan2018survey' on page 8 undefined on input l
ine 324.


Package natbib Warning: Citation `bengio2012deep' on page 8 undefined on input
line 324.


Package natbib Warning: Citation `ng2015deep' on page 8 undefined on input line
 324.


Underfull \vbox (badness 3724) has occurred while \output is active []


Package natbib Warning: Citation `nogueira2017towards' on page 8 undefined on i
nput line 326.


Package natbib Warning: Citation `yosinski2014transferable' on page 8 undefined
 on input line 326.


Package natbib Warning: Citation `shah2021deep' on page 8 undefined on input li
ne 326.

[8]

Package natbib Warning: Citation `shah2021deep' on page 9 undefined on input li
ne 328.
```

```
Package natbib Warning: Citation `mao2016image' on page 9 undefined on input li
ne 328.


Package natbib Warning: Citation `ronneberger2015u' on page 9 undefined on inpu
t line 328.


Underfull \vbox (badness 3219) has occurred while \output is active []


Package natbib Warning: Citation `hore2010image' on page 9 undefined on input l
ine 330.


Package natbib Warning: Citation `setiadi2021psnr' on page 9 undefined on input
 line 330.


Underfull \vbox (badness 3219) has occurred while \output is active []

[9] [10]

Package natbib Warning: Citation `smith2021structured' on page 11 undefined on
input line 336.


Underfull \vbox (badness 10000) has occurred while \output is active []


LaTeX Warning: File `Figures/Figure8.eps' not found on input line 342.

Package epstopdf Info: Source file: <Figures/Figure8.eps>
(epstopdf)             Output file: <Figures/Figure8-eps-converted-to.pdf>
(epstopdf)             Command: <repstopdf --outfile=Figures/Figure8-eps-conver
ted-to.pdf Figures/Figure8.eps>
(epstopdf)             \includegraphics on input line 342.
Package epstopdf Info: Output file is already uptodate.

! Package pdftex.def Error: File `Figures/Figure8-eps-converted-to.pdf' not fou
nd: using draft setting.

See the pdftex.def package documentation for explanation.
Type  H <return>  for immediate help.
```

```
 ...

l.342 ...width=1.0\linewidth]{Figures/Figure8.eps}

Try typing  <return>  to proceed.
If that doesn't work, type  X <return>  to quit.


! Package siunitx Error: Found prefix part with no unit.

For immediate help type H <return>.
 ...

l.344   }


Each prefix part must be associated with a unit: a prefix part was found
but
no following unit was given.


Package textcomp Info: Symbol \textmu not provided by
(textcomp)             font family Merriwthr-OsF in TS1 encoding.
(textcomp)             Default family used instead on input line 344.

LaTeX Warning: `!h' float specifier changed to `!ht'.


Package natbib Warning: Citation `shah2021deep' on page 11 undefined on
input l
ine 349.


Package natbib Warning: Citation `shah2021deep' on page 11 undefined on
input l
ine 349.


Underfull \hbox (badness 2644) in paragraph at lines 349--350
[]\T1/Merriwthr-OsF/m/up/7.5 (+20) In our pre-vi-ous stud-ies
[\T1/Merriwthr-Os
F/b/n/7.5 (+20) ? \T1/Merriwthr-OsF/m/up/7.5 (+20) ], we al-ready used
the firs
t
 []


Underfull \hbox (badness 2846) in paragraph at lines 349--350
\T1/Merriwthr-OsF/m/up/7.5 (+20) three datasets for the de-nois-ing and
super-r
esolution tasks.
 []
```

```
Underfull \vbox (badness 3724) has occurred while \output is active []

[11]
Underfull \vbox (badness 10000) has occurred while \output is active []


LaTeX Warning: File `Figures/Figure9.eps' not found on input line 362.

Package epstopdf Info: Source file: <Figures/Figure9.eps>
(epstopdf)             Output file: <Figures/Figure9-eps-converted-
to.pdf>
(epstopdf)             Command: <repstopdf --outfile=Figures/Figure9-eps-
conver
ted-to.pdf Figures/Figure9.eps>
(epstopdf)             \includegraphics on input line 362.
Package epstopdf Info: Output file is already uptodate.

! Package pdftex.def Error: File `Figures/Figure9-eps-converted-to.pdf'
not fou
nd: using draft setting.

See the pdftex.def package documentation for explanation.
Type  H <return>  for immediate help.
 ...

l.362 ...width=1.0\linewidth]{Figures/Figure9.eps}

Try typing  <return>  to proceed.
If that doesn't work, type  X <return>  to quit.


! Package siunitx Error: Found prefix part with no unit.

For immediate help type H <return>.
 ...

l.364   }


Each prefix part must be associated with a unit: a prefix part was found
but
no following unit was given.


Package textcomp Info: Symbol \textmu not provided by
(textcomp)             font family Merriwthr-OsF in TS1 encoding.
(textcomp)             Default family used instead on input line 364.

LaTeX Warning: `!h' float specifier changed to `!ht'.


Underfull \vbox (badness 10000) has occurred while \output is active []

[12]
```

```
Underfull \vbox (badness 1038) has occurred while \output is active []

[13]

Package natbib Warning: Citation `jin2020deep' on page 14 undefined on
input li
ne 382.


Package natbib Warning: Citation `chen2021accelerated' on page 14
undefined on
input line 382.


Package natbib Warning: Citation `shah2021deep' on page 14 undefined on
input l
ine 382.


Package natbib Warning: Citation `qiao2021evaluation' on page 14
undefined on i
nput line 382.


Package natbib Warning: Citation `xypakis2022deep' on page 14 undefined
on inpu
t line 382.


Package natbib Warning: Citation `liu2023improving' on page 14 undefined
on inp
ut line 382.


Package natbib Warning: Citation `zhou2020w2s' on page 14 undefined on
input li
ne 382.


Package natbib Warning: Citation `hagen2021fluorescence' on page 14
undefined o
n input line 382.


Package natbib Warning: Citation `parmar2018image' on page 14 undefined
on inpu
t line 382.


Package natbib Warning: Citation `ranftl2021vision' on page 14 undefined
on inp
ut line 382.
```

```
Underfull \vbox (badness 10000) has occurred while \output is active []


Underfull \vbox (badness 10000) has occurred while \output is active []

[14]

Package natbib Warning: Citation `dataset_GigaDB' on page 15 undefined on
input
 line 409.


Package natbib Warning: Citation `zafranshah' on page 15 undefined on
input lin
e 409.

LaTeX Font Info:    Font shape `TS1/Merriwthr-OsF/m/n' will be
(Font)              scaled to size 7.5pt on input line 415.

Package natbib Warning: Citation `zafranshahGit' on page 15 undefined on
input
line 415.

LaTeX Font Info:    Calculating math sizes for size <6.25008> on input
line 416
.
LaTeX Font Info:    Font shape `T1/Merriwthr-OsF/m/up' will be
(Font)              scaled to size 6.25008pt on input line 416.
LaTeX Font Info:    Font shape `T1/Merriwthr-OsF/m/up' will be
(Font)              scaled to size 5.20816pt on input line 416.
LaTeX Font Info:    Font shape `T1/Merriwthr-OsF/m/up' will be
(Font)              scaled to size 4.37503pt on input line 416.

LaTeX Font Warning: Font shape `OMS/cmsy/m/n' in size <4.37503> not
available
(Font)              size <5> substituted on input line 416.


LaTeX Font Warning: Font shape `OML/cmm/m/it' in size <4.37503> not
available
(Font)              size <5> substituted on input line 416.

LaTeX Font Info:    Font shape `T1/Merriwthr-OsF/m/n' will be
(Font)              scaled to size 5.20816pt on input line 416.
LaTeX Font Info:    Font shape `T1/Merriwthr-OsF/m/n' will be
(Font)              scaled to size 4.37503pt on input line 416.
LaTeX Font Info:    Font shape `T1/Merriwthr-OsF/m/it' will be
(Font)              scaled to size 6.25008pt on input line 416.
LaTeX Font Info:    Font shape `T1/Merriwthr-OsF/m/it' will be
(Font)              scaled to size 5.20816pt on input line 416.
LaTeX Font Info:    Font shape `T1/Merriwthr-OsF/m/it' will be
(Font)              scaled to size 4.37503pt on input line 416.

Underfull \hbox (badness 10000) in paragraph at lines 430--431
```

```
\T1/Merriwthr-OsF/m/up/7.5 (+20) SIM: struc-tured il-lu-mi-na-tion mi-
croscopy;
 SR-SIM: super-
 []


Underfull \hbox (badness 1259) in paragraph at lines 430--431
\T1/Merriwthr-OsF/m/up/7.5 (+20) aper-ture; PSNR: peak-signal-to-noise
ra-tio;
SSIM: struc-tural
 []


Underfull \hbox (badness 10000) in paragraph at lines 430--431
\T1/Merriwthr-OsF/m/up/7.5 (+20) and in-ter-ac-tive re-con-struc-tion for
struc
-tured il-lu-mi-na-tion
 []

[15]
No file main_manuscript.bbl.

Package natbib Warning: There were undefined citations.

[16

]
enddocument/afterlastpage: lastpage setting LastPage.
(./main_manuscript.aux)

LaTeX Font Warning: Size substitutions with differences
(Font)              up to 1.0pt have occurred.


LaTeX Font Warning: Some font shapes were not available, defaults
substituted.

Package rerunfilecheck Info: File `main_manuscript.out' has not changed.
(rerunfilecheck)             Checksum:
FB524A8FC701B0640AF59E828D72E88C;4319.
 )
Here is how much of TeX's memory you used:
 36023 strings out of 476024
 729419 string characters out of 5794017
 1907382 words of memory out of 5000000
 55082 multiletter control sequences out of 15000+600000
 1975334 words of font info for 622 fonts, out of 8000000 for 9000
 1141 hyphenation exceptions out of 8191
 123i,12n,131p,2036b,1235s stack positions out of
10000i,1000n,20000p,200000b,200000s

pdfTeX warning (dest): name{Hfootnote.1} has been referenced but does not
exist
, replaced by a fixed one
```

```
{c:/TeXLive/2022/texmf-dist/fonts/enc/dvips/cm-super/cm-super-
ts1.enc}{c:/TeXLi
ve/2022/texmf-dist/fonts/enc/dvips/lm/lm-ec.enc}{c:/TeXLive/2022/texmf-
dist/fon
ts/enc/dvips/merriweather/merriwthr_posqbl.enc}{c:/TeXLive/2022/texmf-
dist/font
s/enc/dvips/merriweather/merriwthr_owzwzj.enc}<c:/TeXLive/2022/texmf-
dist/fonts
/type1/sorkin/merriweather/Merriwthr-Bold.pfb><c:/TeXLive/2022/texmf-
dist/fonts
/type1/sorkin/merriweather/Merriwthr-
BoldItalic.pfb><c:/TeXLive/2022/texmf-dist
/fonts/type1/sorkin/merriweather/Merriwthr-
Italic.pfb><c:/TeXLive/2022/texmf-di
st/fonts/type1/sorkin/merriweather/Merriwthr-
Regular.pfb><c:/TeXLive/2022/texmf
-
dist/fonts/type1/public/amsfonts/cmextra/cmex7.pfb><c:/TeXLive/2022/texmf
-dist
/fonts/type1/public/amsfonts/cm/cmsy6.pfb><c:/TeXLive/2022/texmf-
dist/fonts/typ
e1/public/amsfonts/cm/cmsy7.pfb><c:/TeXLive/2022/texmf-
dist/fonts/type1/public/
amsfonts/euler/eufm7.pfb><c:/TeXLive/2022/texmf-
dist/fonts/type1/public/amsfont
s/euler/eurm7.pfb><c:/TeXLive/2022/texmf-
dist/fonts/type1/public/lm/lmtt8.pfb><
c:/TeXLive/2022/texmf-dist/fonts/type1/public/cm-
super/sfrm0600.pfb><c:/TeXLive
/2022/texmf-dist/fonts/type1/public/cm-super/sfrm0700.pfb>
Output written on main_manuscript.pdf (16 pages, 425466 bytes).
PDF statistics:
 349 PDF objects out of 1000 (max. 8388607)
 299 compressed objects within 3 object streams
 60 named destinations out of 1000 (max. 500000)
 225519 words of extra memory for PDF output out of 266212 (max.
10000000)
```

Figure 1

Click here to access/download;LaTeX - Figure (eps, ps, etc.);Figure1.eps

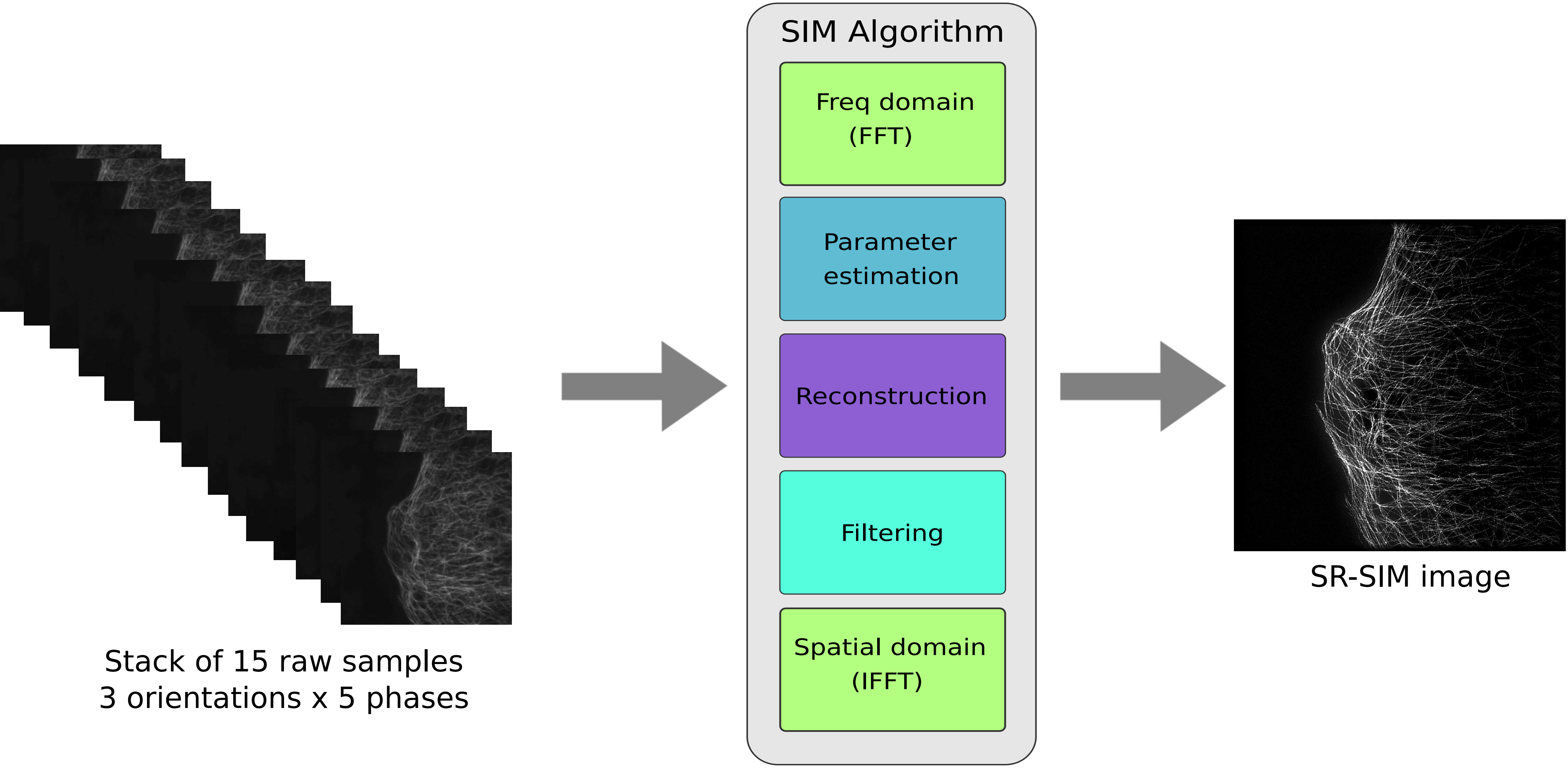

Figure 2

Click here to access/download;LaTeX - Figure (eps, ps, etc.);Figure2.eps

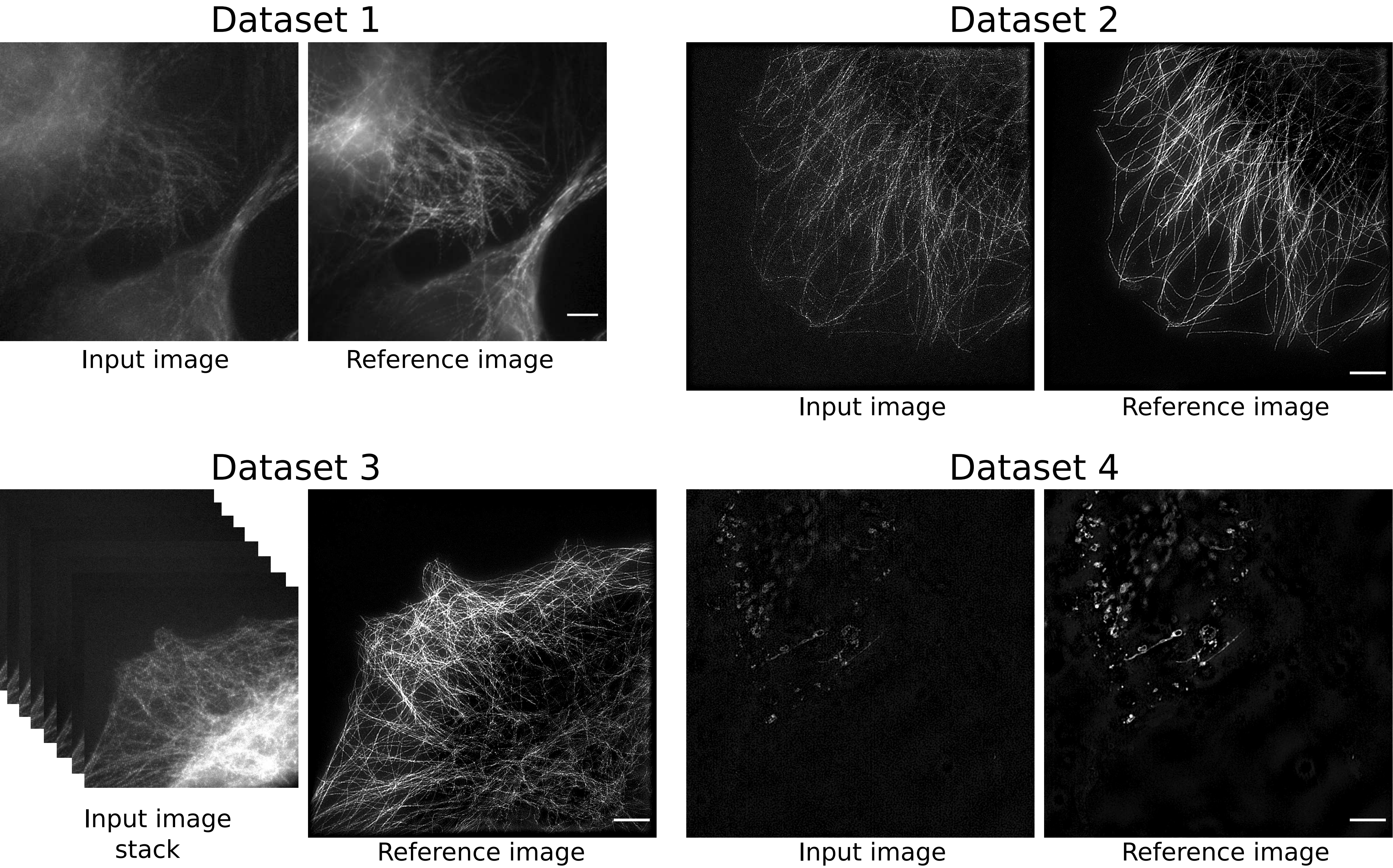

Figure 3

Click here to access/download;LaTeX - Figure (eps, ps, etc.);Figure3.eps

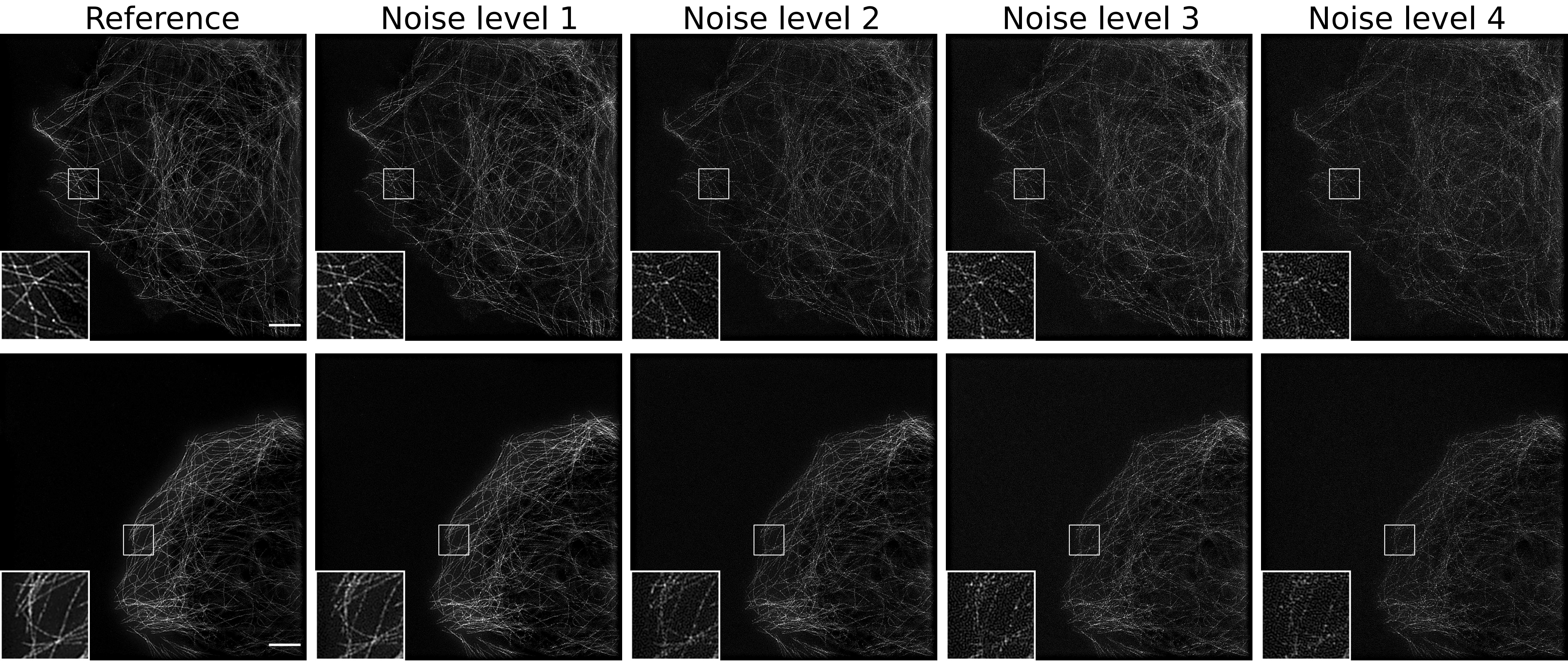

Figure 4

Click here to access/download;LaTeX - Figure (eps, ps, etc.);Figure4.eps

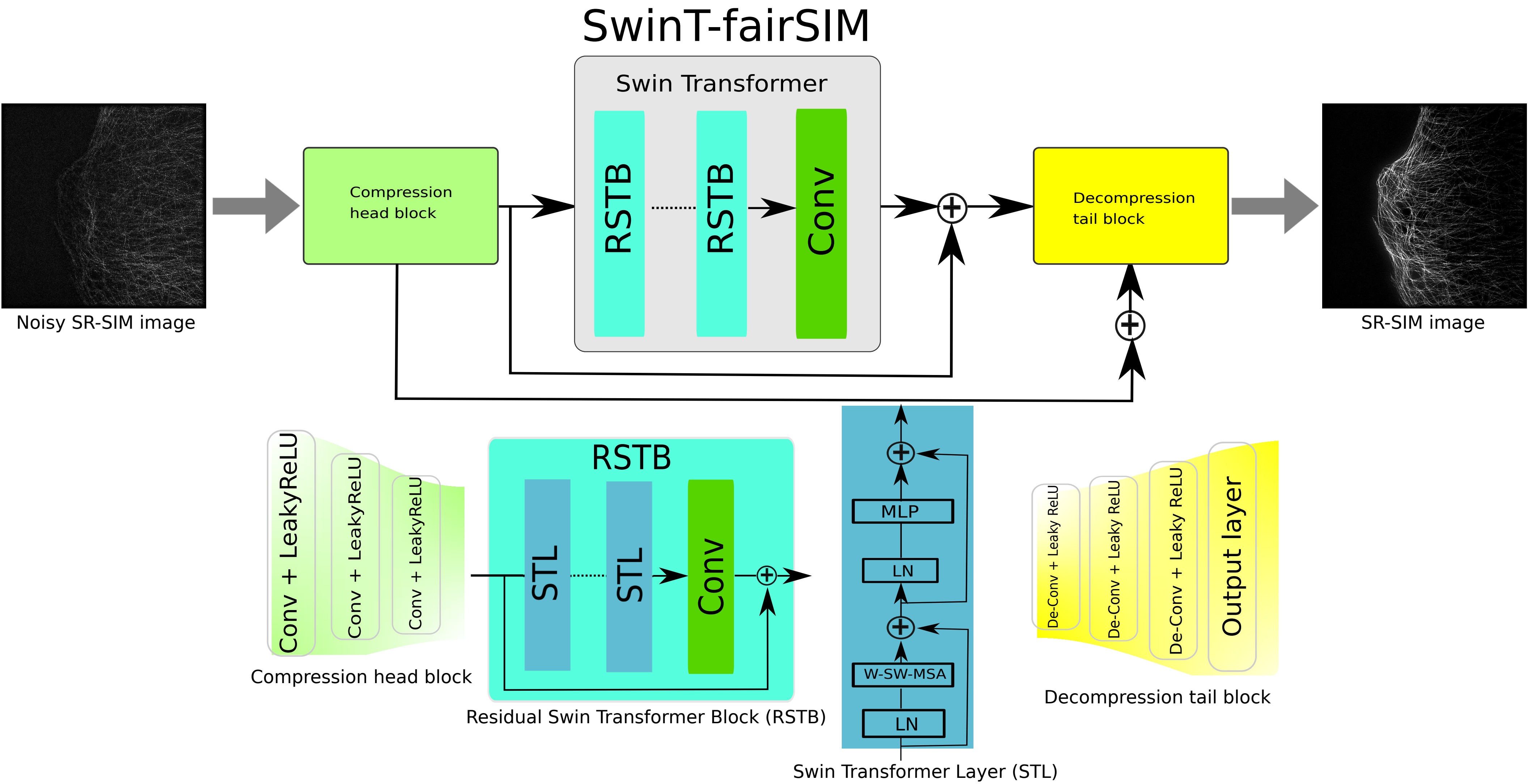

Figure 5

Click here to access/download;LaTeX - Figure (eps, ps, etc.);Figure5.eps

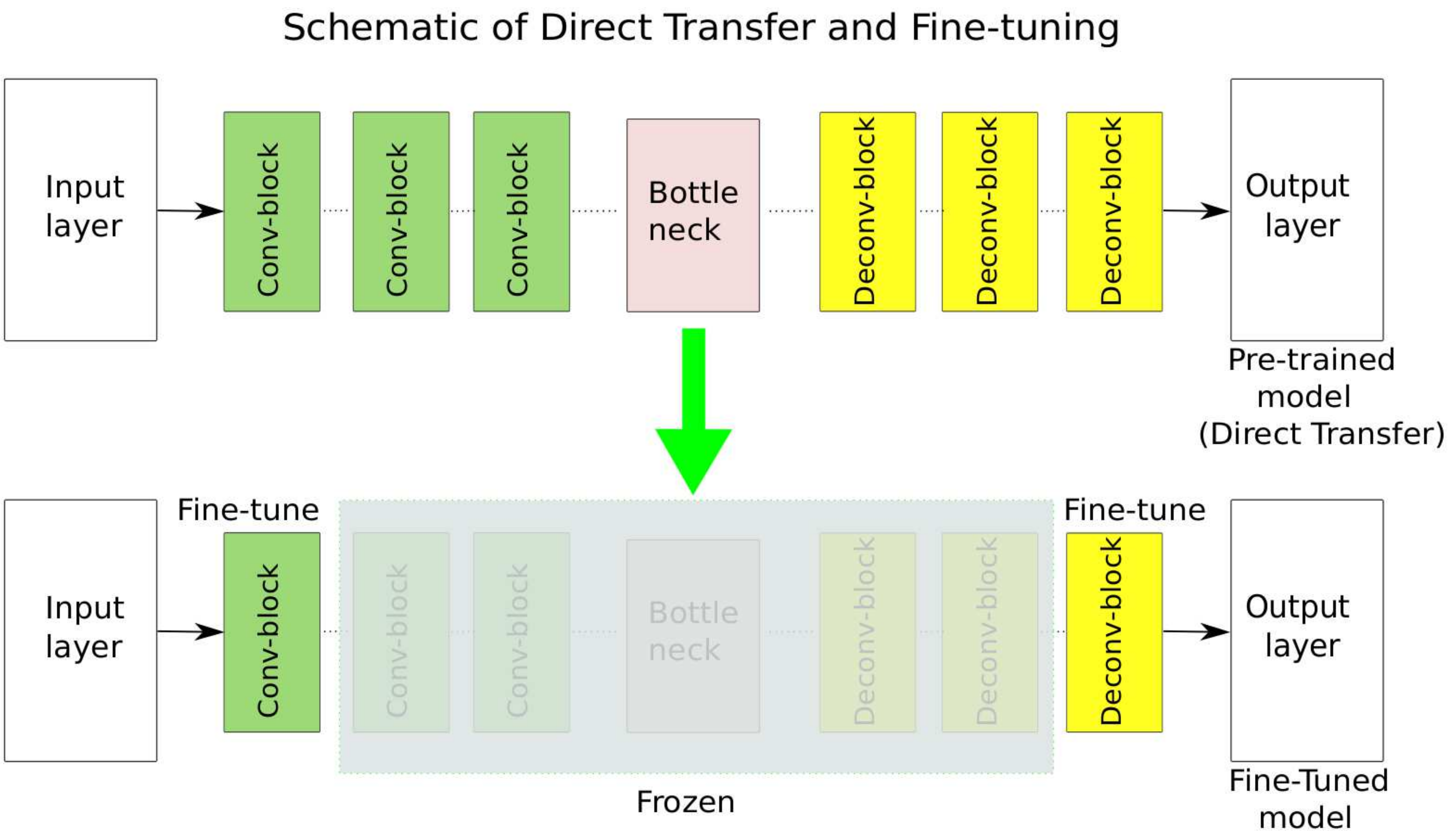

Figure 6

Click here to access/download;LaTeX - Figure (eps, ps, etc.);Figure6.eps

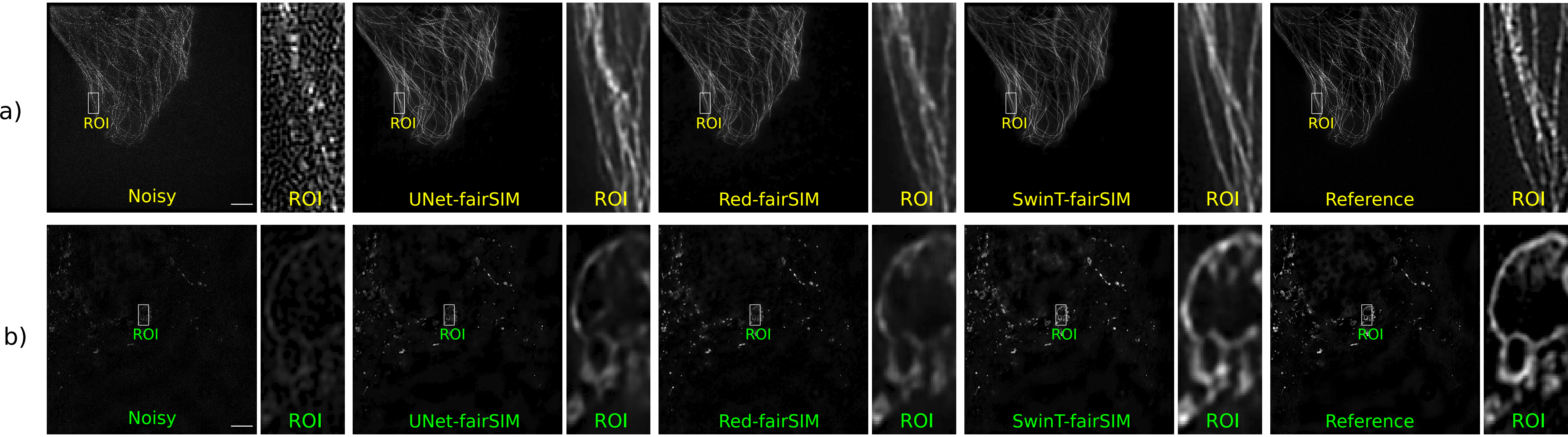

Figure 7

Click here to access/download;LaTeX - Figure (eps, ps, etc.);Figure7.eps

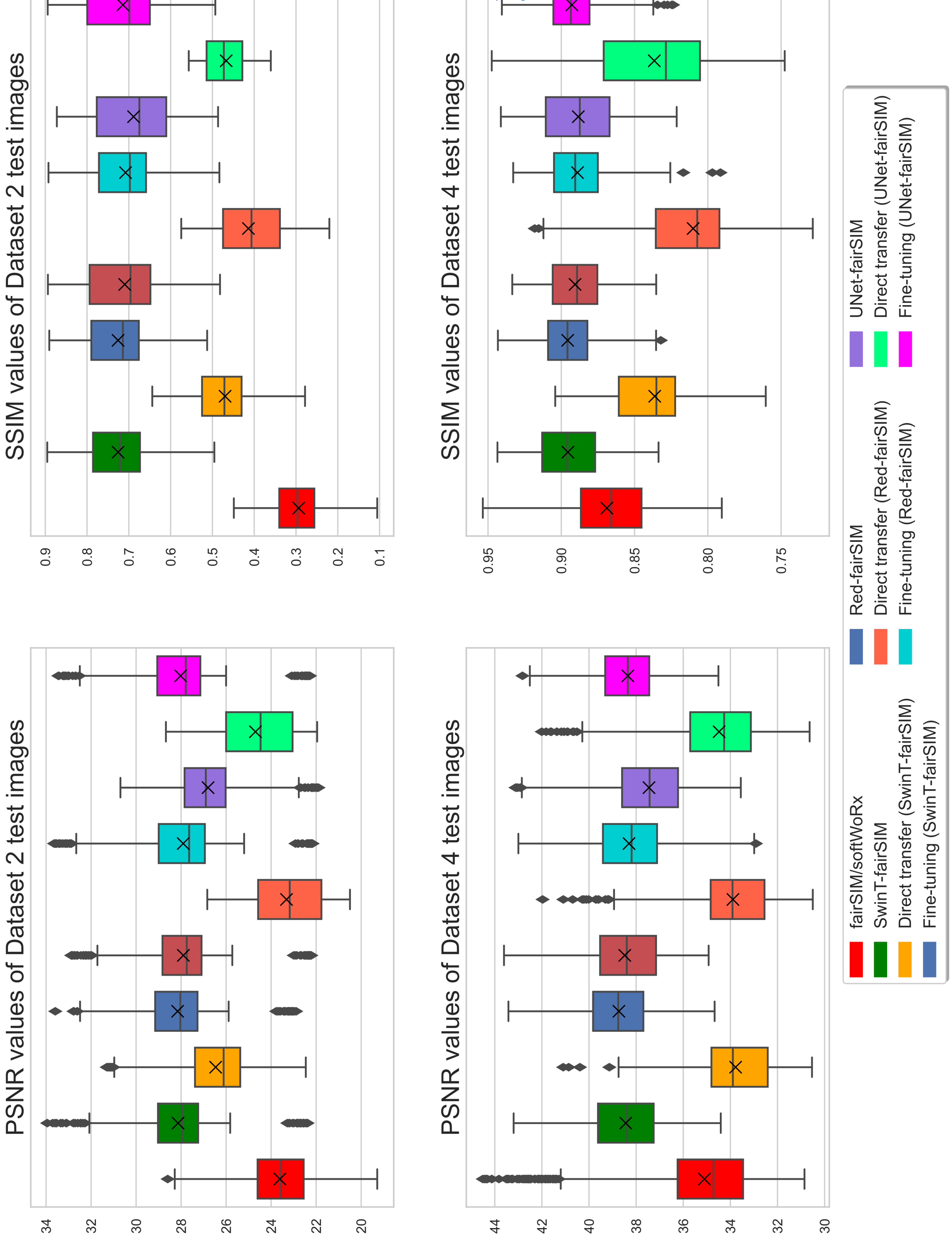

Figure 8

Click here to access/download;LaTeX - Figure (eps, ps, etc.);Figure8.eps

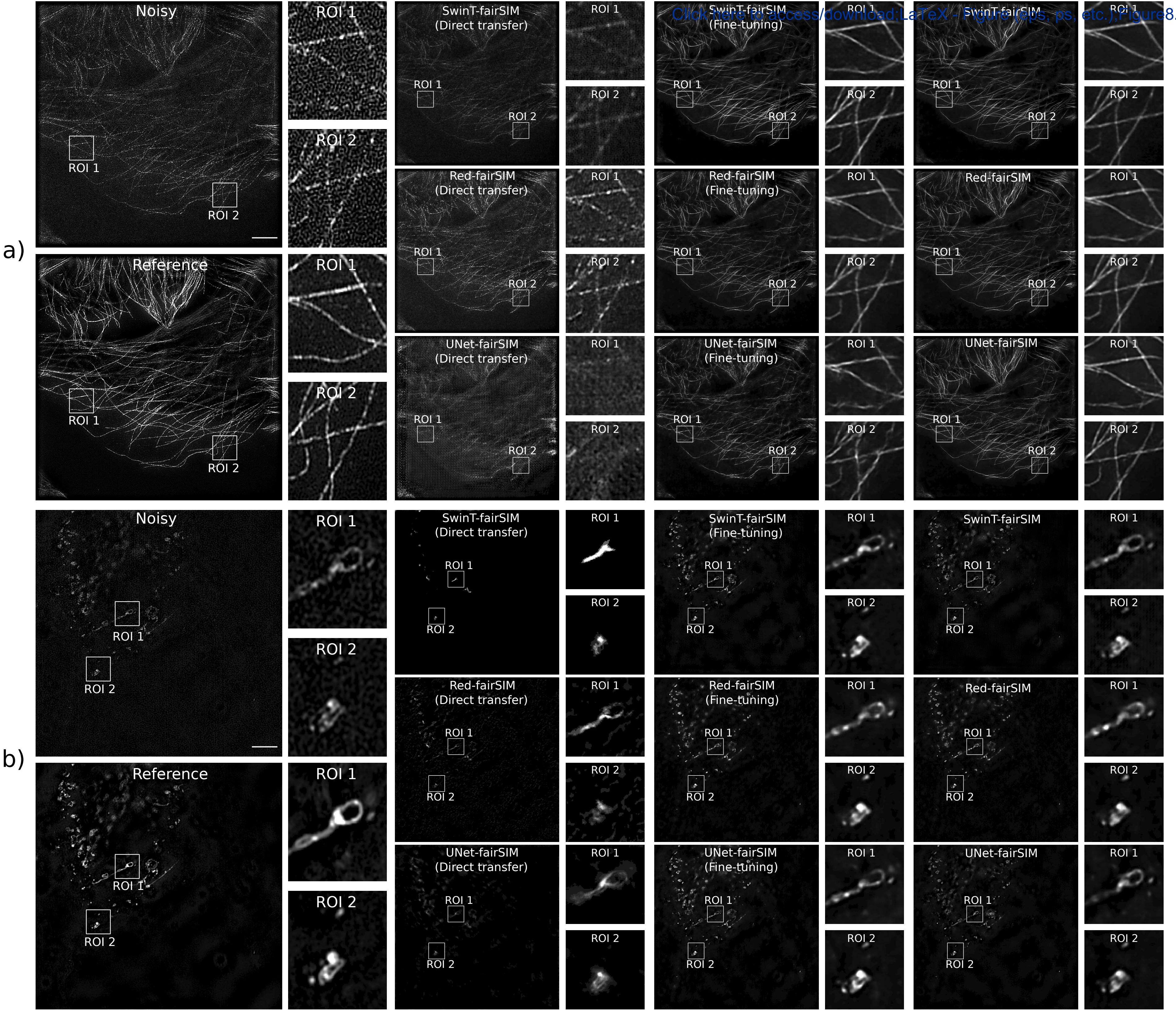

Figure 9

Click here to access/download;LaTeX - Figure (eps, ps, etc.);Figure9.eps

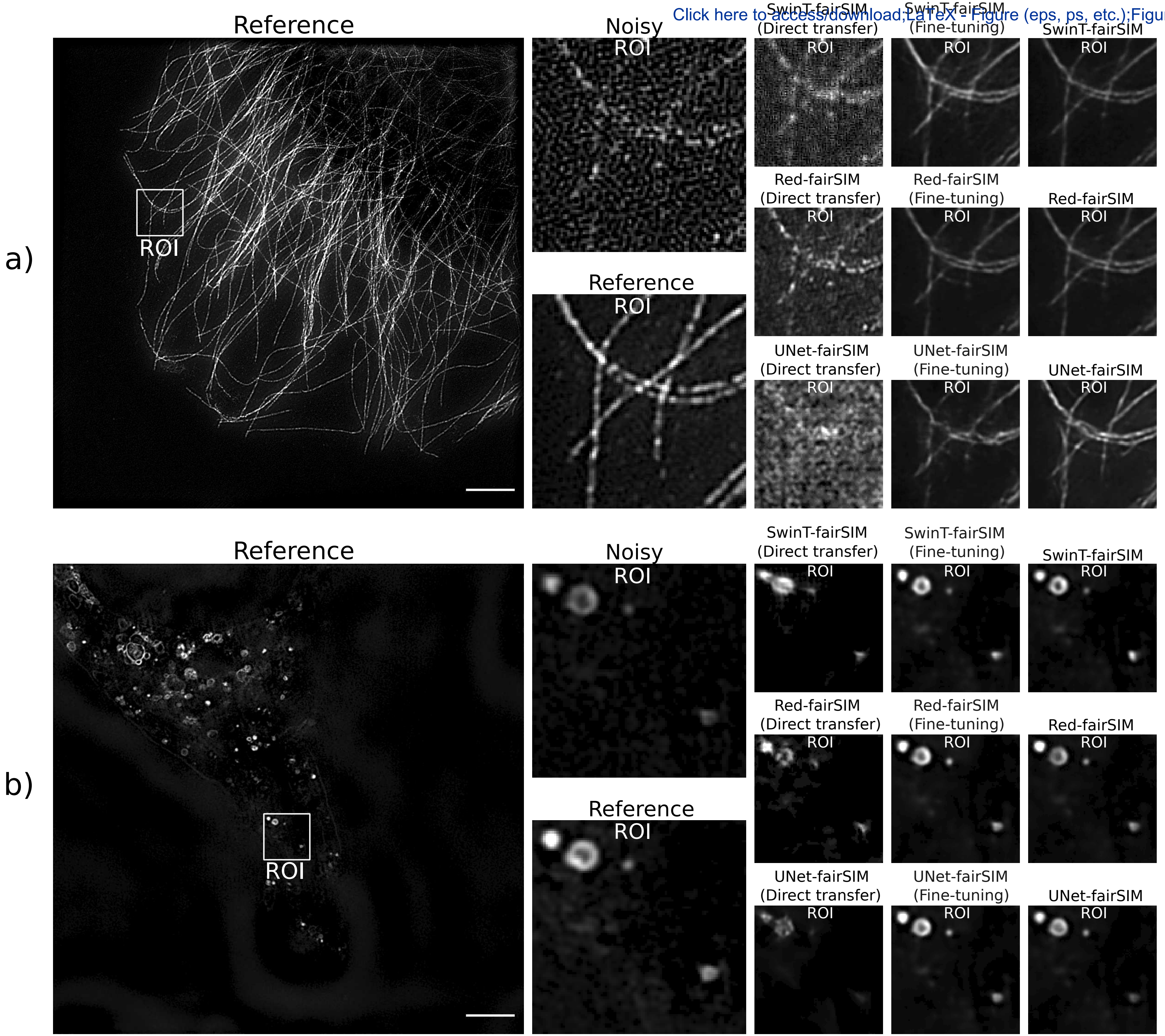

Manuscript

Click here to access/download;Manuscript;Manuscript_GIGA-D-23-00044_Revisions.pdf

OXFORD (GIGA)^n SCIENCE

GigaScience, 2023, 1–15

doi: xx.xxxx/xxxx
Research

RESEARCH

Evaluation of Swin Transformer and knowledge transfer for denoising of super-resolution structured illumination microscopy data

Zafran Hussain Shah[1,*], Marcel Müller[2], Wolfgang Hübner[2], Tung-Cheng Wang[2,3], Daniel Telman[1], Thomas Huser[2] and Wolfram Schenck[1,*]

[1]Faculty of Engineering and Mathematics, Bielefeld University of Applied Sciences and Arts, Interaktion 1, 33619 Bielefeld, Germany and [2]Faculty of Physics, Bielefeld University, Universitätsstr. 25, 33615 Bielefeld, Germany and [3]Leica Microsystems CMS GmbH, Am Friedensplatz 3, 68165 Mannheim, Germany

[*]zafran_hussain.shah@hsbi.de; wolfram.schenck@hsbi.de

Abstract

Background:
Convolutional Neural Network (CNN)-based methods have shown excellent performance in denoising and reconstruction of super-resolved structured illumination microscopy (SR-SIM) data. Therefore, CNN-based architectures have been the focus of existing studies. However, Swin Transformer, an alternative and recently proposed deep learning-based image restoration architecture, has not been fully investigated for denoising SR-SIM images. Furthermore, it has not been fully explored how well transfer learning strategies work for denoising SR-SIM images with different noise characteristics and recorded cell structures for these different types of deep learning-based methods. Currently, the scarcity of publicly available SR-SIM datasets limits the exploration of the performance and generalization capabilities of deep learning methods.

Results:
In this work, we present SwinT-fairSIM, a novel method based on the Swin Transformer for restoring SR-SIM images with low signal-to-noise ratio (SNR). The experimental results show that SwinT-fairSIM outperforms previous CNN-based denoising methods. Furthermore, as a second contribution, two types of transfer learning, namely direct transfer and fine-tuning, were benchmarked in combination with SwinT-fairSIM and two CNN-based methods for denoising SR-SIM data. Direct transfer did not prove to be a viable strategy, but fine-tuning produced results comparable to conventional training from scratch, while saving computational time and potentially reducing the amount of training data required. As a third contribution, we publish four datasets of raw SIM images and already reconstructed SR-SIM images. These datasets cover two different types of cell structures, tubulin filaments and vesicle structures. Different noise levels are available for the tubulin filaments.

Conclusions:
In conclusion, the SwinT-fairSIM method is well suited for denoising SR-SIM images. By fine-tuning, already trained models can be easily adapted to different noise characteristics and cell structures. Furthermore, the provided datasets are structured in a way that the research community can readily use them for research on denoising, super-resolution, and transfer learning strategies.

Key words: Structured illumination microscopy; Fluorescence microscopy; Deep learning; Transformer; Swin Transformer; SwinIR; Convolutional neural networks; Denoising; Image restoration; Transfer learning; Fine-tuning

Introduction

In optical microscopy, super-resolution structured illumination microscopy (SR-SIM) plays a significant role in the field of biological and biomedical studies to analyze living cells and biological specimens with characteristic features below the resolution limit of classical microscopes (approx. 250 nm for high-end systems with oil-immersion objective lenses). Structures of interest include e.g. the internal organelles of mitochondria, cellular cytoskeleton, virus particles, or small vesicles [1, 2, 3, 4, 5]. SR-SIM is an important super-resolution approach to disentangle complex biological cellular structures with an up to twofold enhancement of spatial resolution beyond the diffraction limit. During the process of super-resolution imaging, SR-SIM involves the illumination of the biological sample with spatially patterned light following a sinusoidal intensity distribution. A series of raw images with typically 3 or 5 different phase positions and 3 angles of orientation of the illumination pattern are normally acquired [6]. Subsequently, frequency-domain based image reconstruction algorithms, e.g. implemented in software packages such as fairSIM [7], OpenSIM [8], and Python-based packages [9] are applied to a set of raw SIM images to generate the final twofold super-resolved images. SR-SIM has many advantages over other super-resolution methods e.g., it does not require special sample preparation, it permits the use of conventional fluorophores in multiple colors and simultaneously, it allows imaging at high speed for large fields-of-view (FOVs) while being compatible with live cell samples by making efficient use of low illumination intensity levels [10, 11]. The conventional SR-SIM reconstruction algorithms have some limitations whenever the signal-to-noise level of the raw images is poor due to low fluorescence emission or short exposure times [12].

In general, in fluorescence microscopy weak emission due to, e.g. low labeling densities, high light scattering or absorption, or optical aberrations is often encountered [13, 14]. In the case of several super-resolution fluorescence microscopy methodologies, this leads to low signal-to-noise ratio (SNR), and poor image reconstructions or artifacts. Nevertheless, SR-SIM imaging provides better resolution and optical sectioning abilities than confocal microscopy with a just minimally larger number of raw images to be acquired [15].

In the last decade, deep learning methods have become widely accepted in image processing. In addition, they are being used with increasing success for the restoration of SR-SIM images, e.g. for denoising [16, 17, 18, 19, 20]. Qiao et al. proposed a generative adversarial network (GAN) based deep Fourier channel attention network (DFCAN) method to reconstruct SR-SIM images under low SNR conditions [21]. Xypakis et al. introduced a custom convolutional neural network (CNN) architecture for blind-SIM: BS-CNN [22]. Liu et al. proposed a dual-domain learning strategy for the reconstruction of SIM images [23]. Authors in [24] suggested another GAN based channel attention generative adversarial network (caGAN) to improve the quality of 3D-SIM reconstruction using fewer raw samples of low SNR.

Transformers have recently shown great success in various natural language processing tasks [25, 26, 27, 28, 29]. Since then, as an alternative to CNNs, Transformer-based architectures have also been adapted to computer vision tasks as well, such as classification [30], detection [31, 32], and image restoration [33]. Vision Transformers for the image restoration typically divide each image into fixed-size patches and process each patch independently to limit computational complexity [34], resulting in the introduction of border artifacts around each patch in the restored image. The Swin Transformer overcomes this shortcoming by integrating a shifted window based multi-head self attention (MSA) in the Transformer architecture [35]. Swin serves as the basis for the Shifted window Image Restoration (SwinIR) method [36], which has been proposed especially for various image restoration tasks. Although these latest Transformers outperform CNN-based methods in conventional image restoration to some extent, they were never explored for the restoration of high-resolution SR-SIM microscopy images. Therefore, during this work, we propose a SwinIR based Transformer architecture named "SwinT-fairSIM" to denoise SR-SIM images under low SNR conditions.

Both CNN- and Transformer-based methods require a large number of images to train the underlying models. In the field of microscopy, the sheer size and storage requirements of datasets of high-resolution reconstructed microscopic images make them difficult to produce and typically not publicly available. The few open source datasets available are mostly related to wide-field microscopy and contain a relatively small number of images. For example, Zhang et al. [37] collected three wide-field microscopy datasets purely for denoising tasks without providing high-resolution ground truth images obtained by SR-SIM technology. They used image averaging to generate ground truth data with high SNR images. Zhou et al. [38], on the other hand, published a dataset called "Widefield2SIM" using wide-field fluorescence microscopy. They captured 120 different fields of view (FOVs) with 400 low SNR images for each FOV and generated high-resolution ground truth data using SR-SIM imaging technology. Qiao et al. [21] presented the "BioSR" dataset consisting of 2200 pairs of low-resolution (LR) raw data and high-resolution (HR) data. According to Qiao et al., the BioSR dataset covers four different biological structures (CCPs, ER, MTs, F-actin), nine signal levels (15-600 average photon count), and two upscaling-factors (linear SIM and non-linear SIM). Similarly, Hagen et al. [39] published a variety of datasets which were collected using wide-field and confocal microscopy. They captured various fluorescently labeled structures such as actin, mitochondria, membrane, and nuclei with low and high SNR. However, their collection of datasets consists of significantly fewer images than the BioSR data collection.

Here, we present a series of datasets that are related to SR-SIM microscopy for image restoration tasks. These datasets cover two types of biological structures, tubulin filaments, and vesicles, with multiple fields-of-view for the denoising, super-resolution, and joint denoising and super-resolution tasks. In our datasets, noisy input and reference output images were obtained by using SR-SIM reconstruction algorithms. We believe that our datasets will be helpful to the research community in benchmarking different deep learning-based denoising and super-resolution (SR) methods, not least because of the rather large number of provided samples.

Finally, we show how some of these datasets can be used to demonstrate the generalization capabilities of image restoration algorithms in the field of SIM microscopy. To this end, we apply the concept of transfer learning to three algorithms for the denoising of SR-SIM data. Two of these algorithms are based on CNNs and were proposed by us in [16]. They are called "Red-fairSIM" and "UNet-fairSIM". The third algorithm is the above-mentioned Transformer architecture "SwinT-fairSIM", which is first proposed in this paper. With respect to transfer learning, we compare two methods from this area: Direct transfer and fine-tuning. In direct transfer, a model pre-trained on

Compiled on: November 30, 2023.
Draft manuscript prepared by the author.

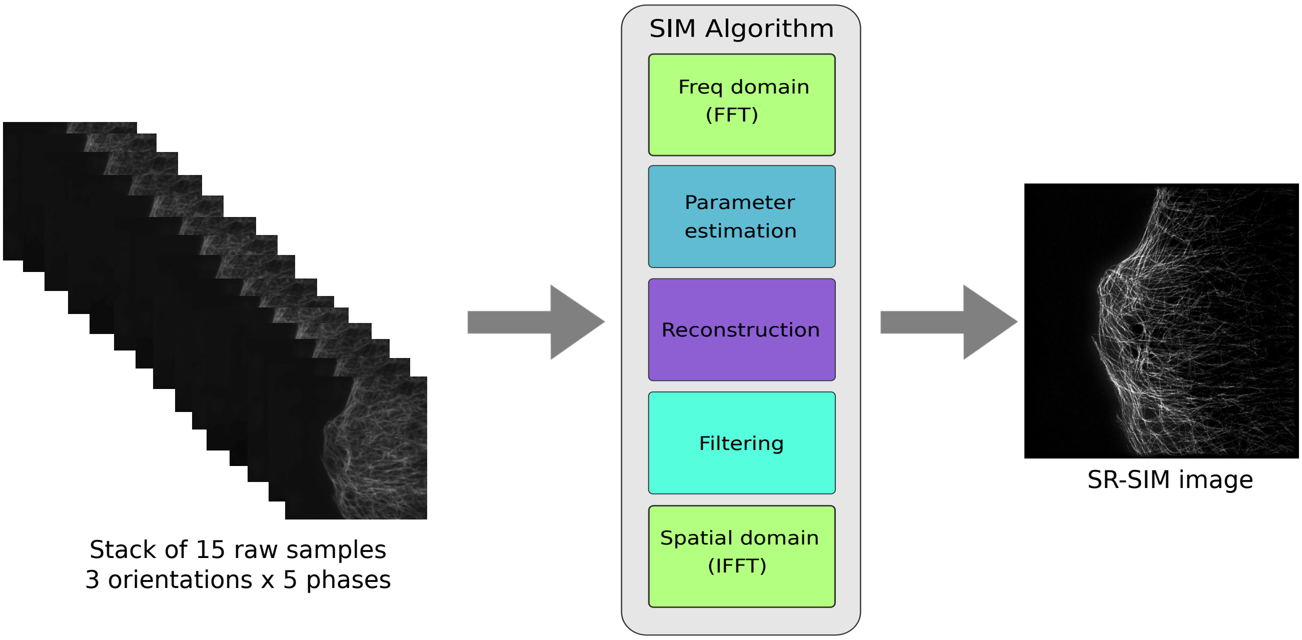

Figure 1. The architecture of the image reconstruction algorithms which are used to generate the reconstructed SR-SIM images in this work. Both reconstruction algorithms (i.e., as implemented in the fairSIM and softWoRx software) are based on the SIM algorithm. During the reconstruction of SR-SIM images, a stack of 15 raw SIM images of size 512 $\times$ 512 pixels is processed by the respective software, which generates the high-resolution SR-SIM image of size 1024 $\times$ 1024 pixels as output.

one type of data is used for inference on a related but different type of data. In fine-tuning, part of the pre-trained model is retrained on the new type of data before inference. In previous work, we were already able to show that some CNN-based algorithms for the denoising of SR-SIM images are robust to different noise levels and SIM modes, e.g. varying pattern spacings at different illumination wavelengths [19]. However, due to the limited amount of SR-SIM data available at that time, we were not able to explore the techniques of transfer learning in more depth. Thus, here, we also aim to answer the following questions related to direct transfer and fine-tuning: (1) If a model is trained on a specific biological structure with a specific type of noise, will it also generalize well to denoising another, different structure with another type of noise? And: (2) Is fine-tuning of a pre-trained model (i.e., previously trained on one type of structure and noise) more effective than training the model from scratch? To answer these questions, we have conducted a series of experiments which are discussed in later sections.

In particular, the contribution of this work is threefold: First, we present high-resolution SR-SIM datasets for image denoising and super-resolution tasks. Second, we propose a method based on the SwinIR architecture for denoising SR-SIM images. Third, we evaluate the potential of direct transfer and fine-tuning for different Transformer- and CNN-based models.

Materials and Methods

SR-SIM microscopy and sample preparation

The raw SIM images for all the datasets were acquired using a DeltaVision OMX V4 (GE Healthcare, Chicago, IL, USA) 3D-SIM imaging system. The DeltaVision OMX V4 imaging system is an implementation of 3-beam 3D-SIM [15], providing both lateral and axial modulation of the excitation pattern, and thus allows to increase both the lateral and axial resolution of the imaging process (as compared to 2-beam SIM, where only the lateral resolution is increased). The system is equipped with four excitation laser lines (405nm, 488nm, 561nm and 642nm), and four sCMOS cameras to detect the emitted fluorescence light in four channels. The camera pixel size of 6.5 µm and an overall magnification of 82x yields a fixed effective pixel size of 80 nm. The magnification cannot be changed by the user, and has been set by the manufacturer to fulfill the Nyquist criterion, thus not loosing spatial information in the sampling process. While fulfilling the Nyquist sampling criterion only sets a maximum pixel size, pixel sizes much smaller than the limit are not used, as this would compromise both SNR (each pixel contributing a constant amount of read noise) and field of view (as the overall feasible pixel count is finite). To prepare the raw SIM image data (datasets 1-3) of the tubulin cytoskeleton, U2OS cells were cultured in DMEM supplemented with 10%FBS and grown on round coverslips of 170$\pm$5 µm thickness (No. 1.5H). Cells were fixed with 4% PFA for 15 min., followed by PBS washes, and permeabilization with 0.5% Triton-X100 for 3 min. Another two rounds of PBS washes were done before blocking with 3% BSA. For immunolabeling of the tubulin microfilaments, cells were stained with anti-tubulin antibody (Invitrogen Cat. No. 322500) 1:400 for 2 hr at room temperature, followed by a PBS wash and one additional hour of incubation with Alexa 488-conjugated anti-mouse IgG 1:400. Afterwards the cells were then briefly washed with PBS before Vectashield was applied to embed the coverslip onto a standard microscopy glass slide for imaging. For the preparation of raw SIM images with vesicle structures (dataset 4), U2OS cells were transfected with Lipofectamine 3000 according to the manufacturers protocol (Thermofisher Cat. No. L3000-001) together with a plasmid expressing the vesicular Lamp1 protein fused to the fluorescent protein mScarlet. After 24 hr transfection the cells were fixed with 4% PFA for 10 min., followed by PBS washes and Vectashield mounting prior to imaging. The vesicular structures represent lysosomes. During the data collection, the illumination intensity as well as the camera exposure time remained fixed. As the cameras are also actively cooled, thermal- and read-noise components should not vary during time-lapse image acquisition. Also, as typical for modern sCMOS cameras,

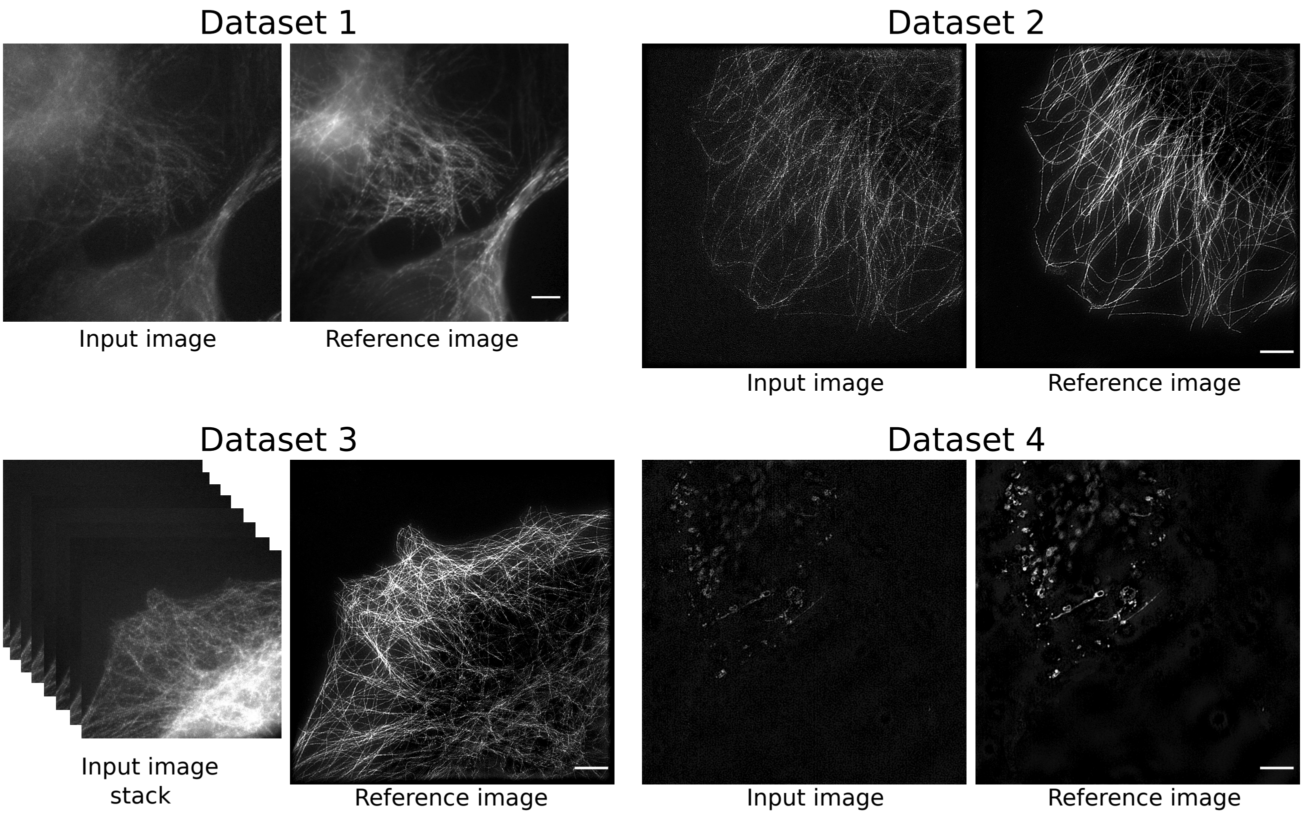

Figure 2. Dataset 1 consists of raw SIM data. The input-reference pairs represent each phase and orientation from different noise levels of the tubulin filaments. The size of the input and reference images are 512 × 512 pixels in dataset 1. Scale bar: 4 µm. Dataset 2 contains the reconstructed SR-SIM images of the tubulin structure, and each input-reference pair has a size of 1024 × 1024 pixels. Scale bar: 4 µm. In dataset 3, raw SIM data in the form of an image stack (i.e., 15 × 512 × 512 pixels) are used as input samples, while the reconstructed SR-SIM images (i.e., 1024 × 1024 pixels) are used as the corresponding reference samples for joint denoising and super-resolution. Scale bar of the reference image: 4 µm. Dataset 4 is based on the reconstructed SR-SIM images of size 1024 × 1024 pixels of fluorescently labeled vesicles. Scale bar: 4 µm.

thermal- and read-noise components are small (in the order of single electrons per read-out) compared to the Poisson noise, i.e. the stochastic distribution of detected photons generated by the quantum nature of light propagation. The dramatic drop in signal-to-noise ratio during data acquisition is due to photobleaching and phototoxicity in the sample.

Dataset preprocessing and image reconstruction

Dataset 1 is a raw dataset without any image processing or reconstruction applied to the raw SIM images. The images in the datasets 2 and 3 are reconstructed by using the open-source fairSIM reconstruction algorithm as shown in Figure 1. fairSIM implements a single-slice (2D) SR-SIM image reconstruction algorithm [7]. It works in three steps: parameter estimation, reconstruction, and filtering. The mathematical and algorithmic details of the fairSIM reconstruction method are explained in the original publication [7]. A synthetic optical transfer function, with NA = 1.4, λ = 525 nm, a = 0.31 (a is a compensation parameter,see [7, 40]) is used. For the tubulin samples, a background of 500 counts per pixel is subtracted during the reconstruction process. SR-SIM reconstruction parameters (pattern orientation, global phase, etc.) are automatically determined by fairSIM's standard, iterative cross-correlation approach. Filter parameters are set to a generalized Wiener filter with a strength of w = 0.05, apodization is set at 1.9× the resolution limit with a *bend* of 0.8. A notch-style filter implemented as *OTF attenuation* with a strength of 0.995 and a FWHM of 1.2 μm^{-1} is used. The full information about the functionality of these parameters is explained in [7], and the general guide for using SIM reconstruction parameters is discussed in [40]. The code used to generate the samples of dataset 2 is available at [41]. The samples in dataset 4 (the vLamp1-mScarlet expressing cell) were reconstructed using the commerical software 'softWoRx v7' (GE Healthcare manufacturer's software) for 3D-SIM. Here, a full 3D volume of data is acquired by the microscope and passed through the reconstruction process as a 3D stack. While both single-slice reconstruction (fairSIM) and full 3D volume reconstruction offer the same increase in lateral resolution, a full 3D volume reconstruction additionally provides an axial resolution increase (when used on 3-beam data as provided by the Delta Vision OMX and other 3-beam SIM systems). This of course comes at the expense of requiring full 3D volumes of data to be acquired, largely increasing imaging time and phototoxicity. Thus, depending on the imaging needs, either single-slice data acquisition (and reconstruction) or full 3D volumes are chosen. For an overview of all datasets, see Table 1.

Description of datasets

Dataset 1

Dataset 1 contains around 101 fields-of-view (FOVs) of tubulin filaments and each FOV further consists of 3000 images (all in one TIF file). In each FOV, a stack of 15 raw SIM images represents the combination of 5 orientations and 3 phases, whereas this full stack is repeatedly captured for 200 timestamps. The signal-to-noise ratio decreases with every timestamp. Thus, dataset 1 contains a total of 303000 raw SIM images of size 512 × 512 (width × height) pixels with 15 combinations of phase and orientation at each timestamp. Each timestamp in the

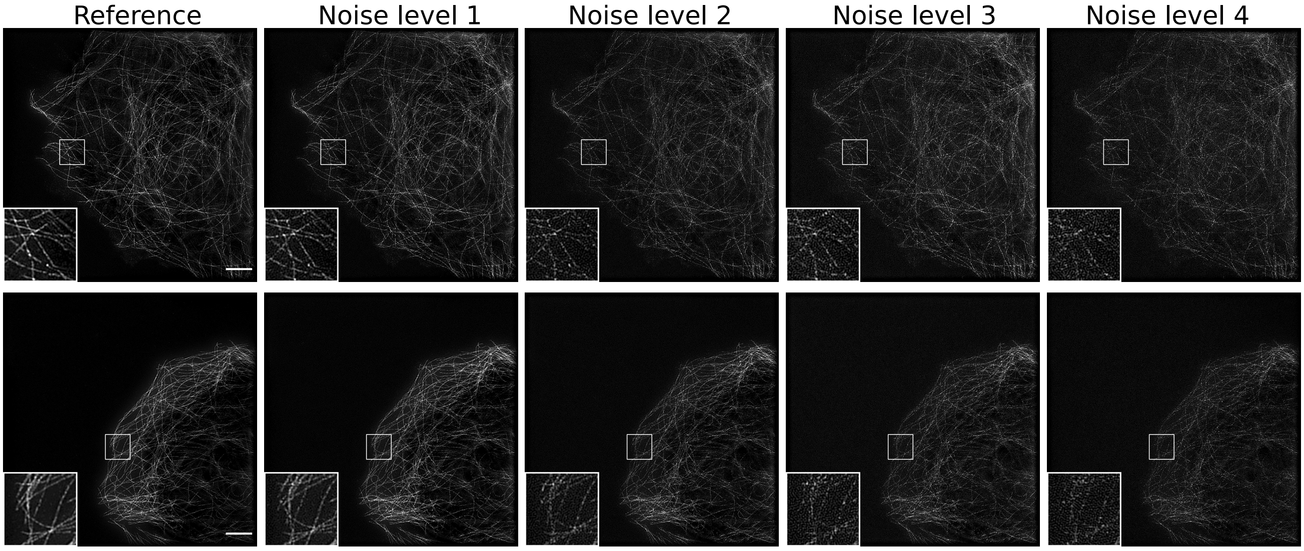

Figure 3. SR-SIM images from two different FOVs, each with different noise levels. Each noise level corresponds to a different range of timestamps, e.g., images of noise level 4 are taken at the last timestamps and contain a high level of noise. Similarly, images of noise level 1 represent early timestamps with very low noise. The reference image is recorded at timestamp 1 and has the highest signal-to-noise ratio. Scale bar: 4 µm.

raw images lasts approximately 25 ms. Each pixel contains a single 16-bit integer value captured by the microscope's camera, which is calibrated to provide a signal linear in photon count for each pixel. This is typical for scientific camera systems, but dissimilar to standard image processing, where often gamma mapping is applied between light intensity and pixel values. This dataset can be used mainly for image denoising tasks (from noisy input to output with higher signal-to-noise ratio). Therefore, the images from timestamp 1 are intended as output images (reference/ground truth), and the rest of the images can be categorized as input images as shown in Figure 2.

Dataset 2

We constructed dataset 2 by applying the fairSIM reconstruction algorithm to the raw SIM images from dataset 1. Dataset 2 contains therefore pairs of reconstructed high-resolution SR-SIM images, each pair consisting of the noisy input and the reference output. The process of the generation of input and output SR-SIM images from the raw SIM images is shown in Figure 1. The 15 raw SIM images of size 512×512 (width $\times$ height) pixels of different phases and orientations are propagated into the fairSIM algorithm to reconstruct the SR-SIM images of size 1024×1024 (width $\times$ height) pixels. During the formation of this dataset, the raw samples from timestamp 1 were used to generate the output images (i.e., reference images). We use the term 'reference images' instead of 'ground truth images' because of the SIM reconstruction artifacts in the output images of this dataset. The input samples were reconstructed by using the raw SIM images from timestamps 176 – 200. Therefore, dataset 2 is composed of 2525 reconstructed pairs of SR-SIM images with a size of 1024×1024 (width $\times$ height) pixels extracted from the 101 FOVs. In addition to these 2525 image pairs, for the last 20 FOVs we also include image pairs in the data collection where the noisy input is from timestamps 76 – 100, 126 – 150 and 176 – 200. This additional data can be used to create test sets to evaluate the robustness of denoising networks for different noise levels. In our previous work [19], we denoted data from timestamp 26 – 50 as noise level 1. Similarly, noise level 2, 3, and 4 correspond to the data from the timestamps 76 – 100, 126 – 150 and 176 – 200 (shown in Figure 3). Overall, dataset 2 is generated mainly for the denoising of SR-SIM images.

Dataset 3

Dataset 3 was created mainly for joint denoising and super-resolution tasks. The composition of this dataset is based on the stack of raw noisy input SIM images obtained from Dataset 1 of noise level 4 and the reference SR-SIM images from dataset 2 of timestamp 1. The raw noisy SIM images in the input stack represents different illumination phases and orientations. The reference samples are the reconstructed high-resolution SR-SIM images. The size and dimension of each input sample are $15 \times 512 \times 512$ (depth $\times$ width $\times$ height) pixels. The size of the output or reference sample is 1024×1024 (width $\times$ height) pixels. The input and reference samples in this dataset contain tubulin filaments as biological structures.

Dataset 4

Dataset 4 is composed of 3D-SR-SIM images showing vesicles in U2OS cells (i.e. typically round intracellular droplets) and can be used mainly for denoising tasks. Full 3D SIM raw data stacks have been passed through a 3D-enabled SIM reconstruction, extending both lateral and axial resolution of the data [15].

This data is composed of nine z-stacks, each with a varying number of depth levels (z-planes), and each captured for a different number of timestamps. In our work, we focus on the denoising along the lateral plane, therefore each z-stack is split into its single z-planes (also called "slices"). The recorded structures in the different z-planes are very different from each other, so we categorize them here as separate FOVs.[1]

As a result, the raw data of this dataset contains 175 FOVs, and each FOV is recorded for a different number of timestamps. This yields a total of 7284 pairs of input and output SR-SIM images. The size of the input and reference images is 1024×1024 (width $\times$ height) pixels. In each image pair, the output/reference image is taken from timestamp 1 of the respective FOV, whereas the input images are taken from the following timestamps and exhibit therefore a lower signal-to-noise ratio. The rational for splitting 3D data into separate 2D slices for denoising and artifact removal is as follows: The structure of the 3D SIM reconstruction algorithm (shifting and recombining lateral but not axial frequencies) makes it very likely that noise and reconstruction artifacts arise in the lateral di-

1 The filenames in the dataset contain also indices for the original z-stack.

Table 1. Description of all the datasets.

Dataset	Dataset 1	Dataset 2	Dataset 3	Dataset 4
Structure	tubulin filaments	tubulin filaments	tubulin filaments	vesicles
Microscope	SR-SIM microscopy	SR-SIM microscopy	SR-SIM microscopy	SR-SIM microscopy
Pixel Size, nm	80	40	80 (input) / 40 (ref.)	40
Number of timestamps	200	200	200	max: 99, min: 15
Fields-of-view	101	101	101	175
Input image size, pixels	512x512	1024x1024	15x512x512	1024x1024
Output/Reference image size, pixels	512x512	1024x1024	1024x1024	1024x1024
No of samples	303000	2525	2525	7284
Reconstruction	raw data	fairSIM	fairSIM	softWoRx

rections of the acquired images, not so much in the axial component. Limiting the post-processing to 2D increases speed and reduces training effort significantly. Also, the height of a 3D stack (number of slices) varies from sample to sample, while the lateral FOV is typically constant. Of course, dataset 4 would allow full 3D implementations to be tested, by recombining the z-sliced data into 3D volumes. The reconstruction of the original raw SIM images into the SR-SIM images in this dataset was performed by using the softWoRx v7 software (GE Healthcare manufacturer's software).

Data partitioning

In our experiments, in the first three datasets, the images of the last 20 FOVs were used as test samples, and the remaining 81 FOVs were used for the training set. The training set of dataset 2 is therefore composed of 2025 samples (image pairs) from 81 FOVs and the test set is composed of 500 samples from 20 FOVs. Similarly, in dataset 4 we used 5562 samples from 121 FOVs for the training set and 1380 samples from 46 FOVs for the test set; the remaining 8 FOVs were discarded because their reference images contain only noise without any meaningful structure. Datasets 1, 2, and 4 can only be used for denoising tasks, however, the data from dataset 3 can be used for both joint denoising and super-resolution. To reproduce the results of this work, we shared the source code on the GitHub repository [42], workflowhub [43], as well as the trained models at [44], and the data on GigaDB [45].

Shifted window Transformer for the denoising of SR-SIM images (SwinT-fairSIM)

The architecture of SwinT-fairSIM is composed of three main components: Compression head, Swin Transformer, and Decompression tail block, as shown in Figure 4. The architecture of SwinT-fairSIM is an extended version of the SwinIR architecture [36]. The compression head block is responsible for shallow feature extraction and the downsizing of the input images, the Swin Transformer block is based on encoder-decoder components to recover missing information and perform deep feature extraction, and the decompression tail component is used for upsampling and recovering the features in the resulting images. The compression head block is composed of three convolution layers to compress the input image features to a size of 256×256 (width × height) pixels by using a stride of size 2. The encoder-decoder Swin Transformer block is made up of several residual Swin Transformer blocks (RSTB), and each RSTB is based on few Swin Transformer layers (STL) and convolutional layers [36]. The STL is further composed of two window (W) and shifted window (SW) based multi-head self-attention (MSA) modules, followed by a multi-layer perceptron that has two fully-connected layers with GELU non-linearity [25, 35]. The shifted window partitioning strategy introduces connections between non-overlapping patches in the preceding layer and is found to be effective in a variety of computer vision tasks [36]. The Swin Transformer layer first reshapes the input (X) of size $H \times W \times C$ (height × width × channels) into $\frac{HW}{M^2} \times M^2 \times C$, where the $\frac{HW}{M^2}$ is the total number of windows or patches. Two additive based residual skip connections are also used in each STL unit. The query (Q), key (K), and value (V) matrices are then calculated for each window separately to obtain self-attention.

$$Q = XP_Q,\ K = XP_K,\ V = XP_V \tag{1}$$

In equation (1), P_Q, P_K, and P_V are the shared projection matrices across all the windows. The attention matrix is then computed by the self-attention mechanism in a local window as follows:

$$\text{Attention}(Q, K, V) = \text{SoftMax}(QK^T/\sqrt{d} + B)V \tag{2}$$

where B in equation (2) is the learnable relative positional encoding and d is the dimension of query and key features. The consecutive Swin Transformer layers are defined as:

$$\hat{X}^l = \text{W-MSA}(\text{LN}(X^{l-1})) + X^{l-1} \tag{3}$$
$$X^l = \text{MLP}(\text{LN}(\hat{X}^l)) + \hat{X}^l \tag{4}$$
$$\hat{X}^{l+1} = \text{SW-MSA}(\text{LN}(X^l)) + X^l \tag{5}$$
$$X^{l+1} = \text{MLP}(\text{LN}(\hat{X}^{l+1})) + \hat{X}^{l+1} \tag{6}$$

In equations (3)-(6), $\hat{X}^l$ and X^l denote the output features of the (S)W-MSA and MLP modules for layer l, where LN represents the LayerNorm operation.

Finally, the decompression tail block accommodates 3 transposed convolutional layers along with the output convolutional layer to transform and upsample the feature maps into the final resulting images. The necessity of head and tail block is to scale down and upsample the SR-SIM images with a size of 1024 × 1024 (width × height) pixels. In the SwinT-fairSIM method, we set the RSBT, STL, window size, and the attention head numbers to 5, 5, 8, and 120 respectively. The adjacent layers in the head and tail blocks are connected via additive skip connections and contain the same number of kernels. These blocks differentiate our architecture from the existing SwinIR architecture [36]. These blocks reduce the computational effort in the central Transformer part of the architecture and therefore adapt the Swin Transformer block to images with a high pixel count.

The name "SwinT-fairSIM" was chosen to be consistent with the names of our previously proposed CNN-based denoising methods for SR-SIM data, "Red-fairSIM" and "UNet-fairSIM" [16]. Red-fairSIM and UNet-fairSIM are described in detail in [16]. These algorithms originally worked only in conjunction with the fairSIM software [7] for SR-SIM reconstruction. In contrast, in this paper, we also use the softWoRx

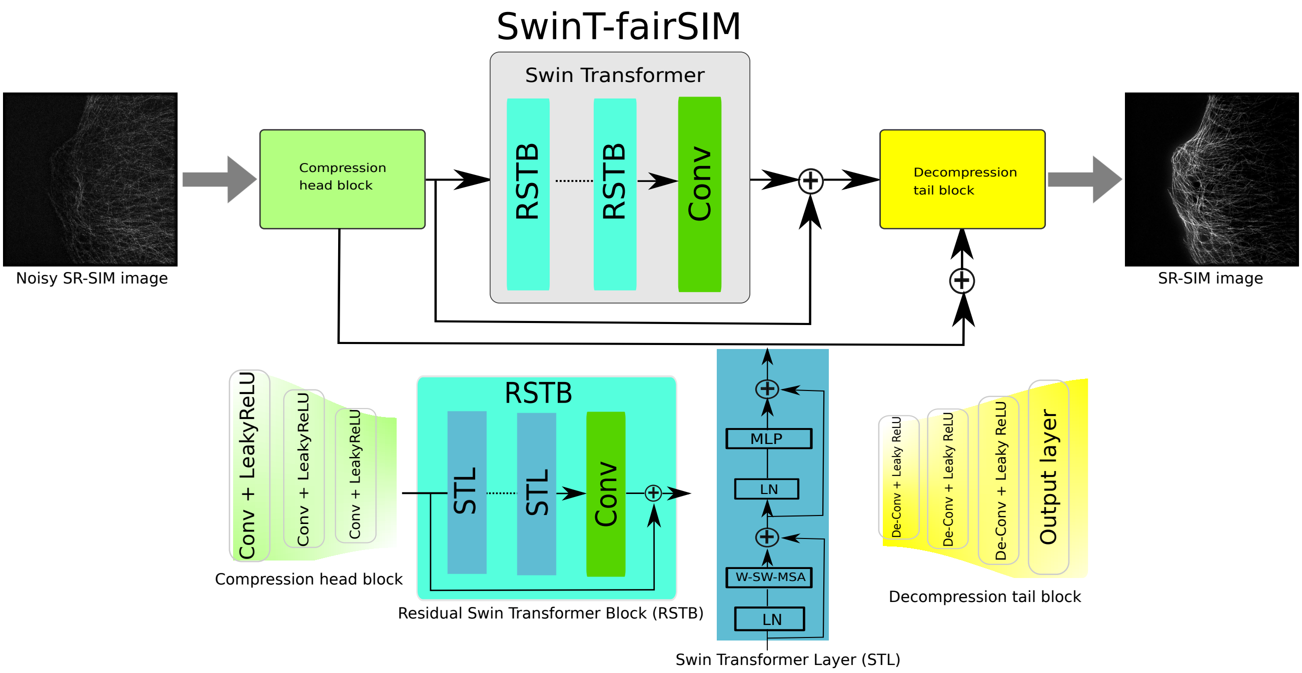

Figure 4. The architecture of the SwinT-fairSIM denoising method. The lower half of the figure provides the description of each block.

implementation of the SIM algorithm for dataset 4. However, to be consistent with the naming scheme in [16], we keep the suffix "fairSIM" in all of our deep learning-based approaches.

Transfer learning and fine-tuning

In transfer learning in general, the knowledge of a trained model from a related task that has been learned is transferred to another task from the same domain [46]. The parameters of the pre-trained model, trained on specific data and for a specific task, are transferred to different data but related task [47, 48]. The use of transfer learning in deep learning is very useful to reduce the computational demands and time complexity [49]. In addition, transfer learning is also very helpful when it comes to alleviating large dataset requirements [50].

The simplest form of transfer learning is to apply a trained model directly to another task without retraining. This direct use of a pre-trained model on new test data is called "direct transfer". Beyond this most basic approach, fine-tuning is one of the most important strategies for transferring model knowledge from one domain to another [51]. During fine-tuning, the weights of some layers of pre-trained models are preserved and the rest of the layers are retrained (i.e., "fine-tuned") [52]. The concepts of direct transfer and fine-tuning are illustrated in Figure 5. It can be seen from the schematic in Figure 5 that the weights of the intermediate layers of the pre-trained models are frozen while the rest of the layers are retrained in the fine-tuning strategy, whereas no retraining is performed in the direct transfer approach. For our work here, in the direct transfer strategy, the models are trained from scratch on dataset 2, and then the trained model is evaluated with test samples from dataset 4. Similarly, the models trained on dataset 4 are evaluated on the test images from dataset 2. In the fine-tuning strategy, models are trained from scratch on dataset 2, afterwards they are partly retrained on dataset 4 and also tested on dataset 4. Or the other way round: Complete initial training on dataset 4, afterwards fine-tuning on dataset 2 and finally evaluation on dataset 2. In this work, we performed these different training strategies with the SwinT-fairSIM, Red-fairSIM, and UNet-fairSIM algorithms [19] with datasets 2 and 4.

To perform fine-tuning on the SwinT-fairSIM algorithm, we retrained the head and tail blocks along with one adjacent RSTB block each. By doing so, about 1.9 million training parameters stayed frozen out of 4 million parameters. Regarding the CNN-based algorithms from our previous work [19], we used the following approaches to fine-tuning: Red-fairSIM is based on the residual-encoder-decoder network (RED-Net) [53]. In the fine-tuning approach for Red-fairSIM, we retrained the first and last 5 layers of the model instead of all 30 layers. By doing so, about 700K training parameters stayed frozen out of more than 1 million parameters. UNet-fairSIM is based on the UNet architecture which is based on several encoder and decoder blocks [54]. In the fine-tuning of UNet-fairSIM, we simply retrained the first and last two encoder and decoder blocks; leaving more than 29 million out of 33 million parameters frozen.

Generally, in the first step, all these algorithms were trained from scratch on datasets 2 and 4 separately for 100 epochs. The mean squared error (MSE) was used as a loss function for all training runs with the Adam optimizer. The learning rate was set to 1×10^{-4} for all training runs. In the next step, we applied direct transfer by propagating the test samples from the respective dataset not used for training the pre-trained models. In direct transfer, the test images of the dataset 2 (i.e., tubulin filaments) were propagated through the pre-trained model trained on the dataset 4 (i.e., vesicle structure) and vice versa. In the third step, the pre-trained models were fine-tuned using training data from the respective dataset not used for initial training. 30 training epochs were used for fine-tuning. The fine-tuned models were then evaluated on the test samples from the dataset used in the fine-tuning process. When fine-tuning the pre-trained models, we initially kept different numbers of layers/blocks frozen. However, we found that the best results were obtained by fine-tuning rather few trainable parameters for 30 epochs (except for SwinT-fairSIM, where a larger part of the network had to be "unfrozen"). In the final step, we compared the results of conventional training with the direct transfer and fine-tuning strategies visually, as well as in terms of peak signal-to-noise ratio (PSNR) [55] and structural

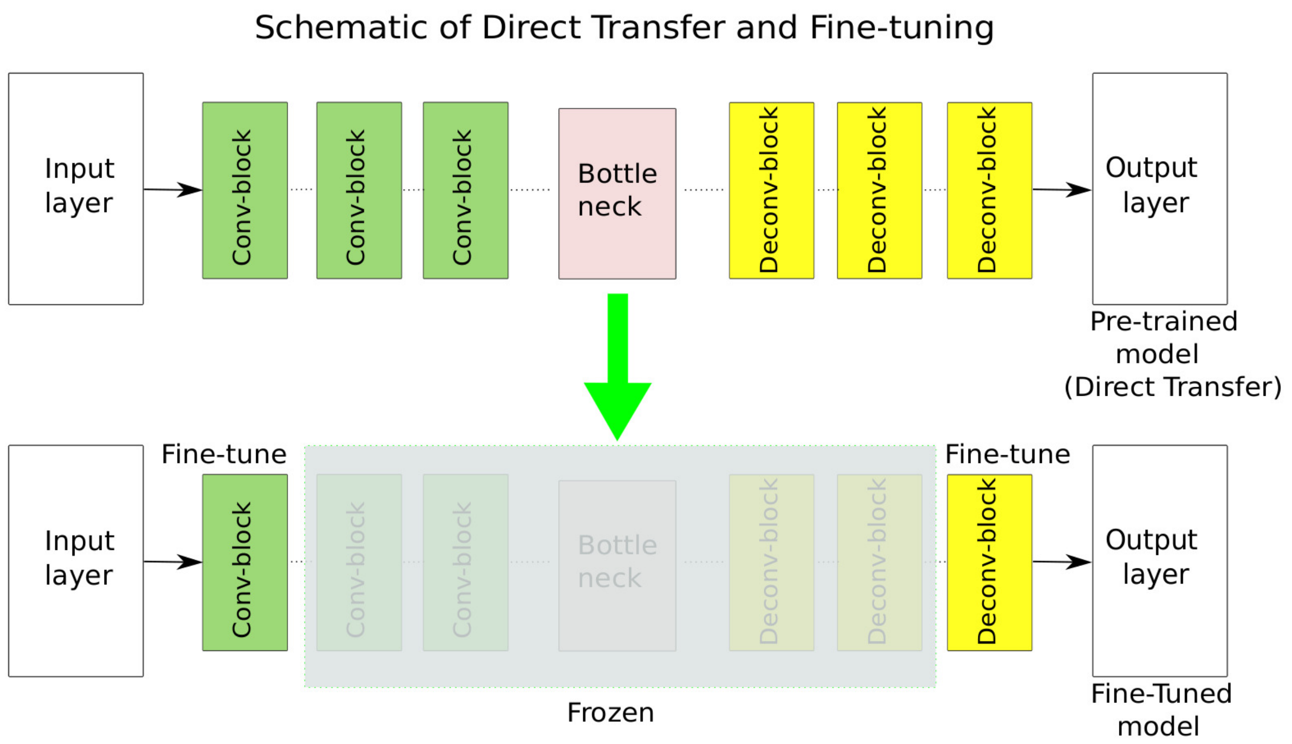

Figure 5. The schematic diagram illustrates the process of direct transfer and fine-tuning. In the first pipeline, the pre-trained model is depicted. The highlighted region in the second pipeline represents the frozen part, which remains unchanged during fine-tuning.

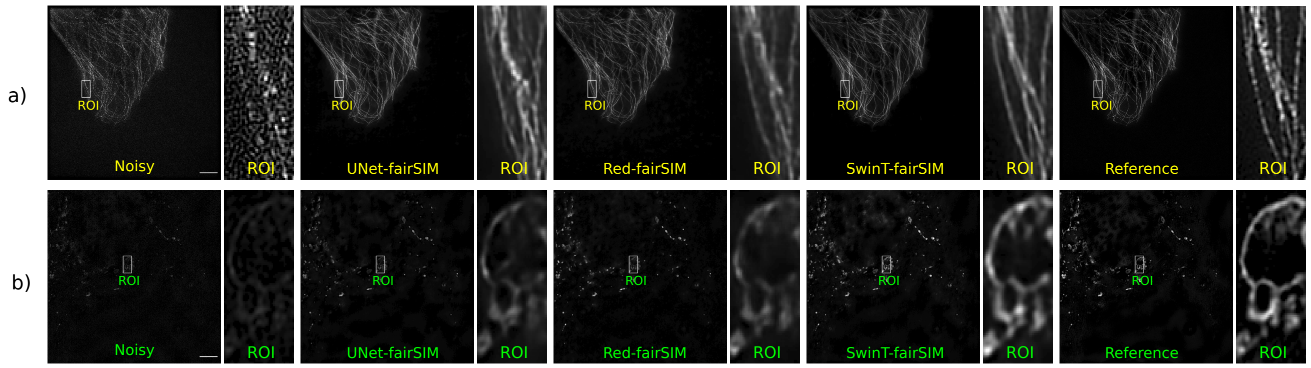

Figure 6. The results of the test samples from datasets 2 and 4 are shown in this figure in the first **(a)** and second **(b)** rows, respectively. UNet-fairSIM, Red-fairSIM, and SwinT-fairSIM outputs are shown side by side. The cropped and zoomed regions of interest (ROIs) extracted from the full-size SR-SIM images 1024×1024 pixels are displayed in the second, fourth, sixth, eighth, and tenth columns. The cropped ROIs of size 50×100 pixels were upsampled to 150×300 pixels for demonstration purposes. Scale bar: 4 µm.

similarity index measurement (SSIM) [56] values.

Results

We collected four data sets for denoising and super-resolution tasks. The overview and full characteristics of these datasets are shown in Table 1. The images in dataset 2 contain the tubulin structure along with mixed Poisson-Gaussian (MPG) noise and SR-SIM reconstruction artifacts. The images in dataset 4 contain the vesicle structure along with MPG noise and honeycomb pattern artifacts that occur when raw data carrying predominantly Poisson noise is subjected to the frequency-based SR-SIM reconstruction algorithm, which then introduces reconstruction artifacts [14].

In our previous studies [19], we already used the first three datasets for the denoising and super-resolution tasks. Here, we mainly focus on the denoising of SR-SIM images by SwinTransfomer-fairSIM and compare the results of this new method with the results of Red-fairSIM and UNet-fairSIM [19]. We also analyze two transfer learning strategies, direct transfer and fine-tuning of the pre-trained models of these network architectures.

To compare the performance of conventional training of these deep learning-based denoising methods with direct transfer and fine-tuning, we first train the SwinT-fairSIM, Red-fairSIM, and UNet-fairSIM networks separately with datasets 2 and 4 for 100 epochs. The results of all methods trained separately with both datasets are shown visually in Figure 6 and quantitatively in Table 2. The visual and quantitative results of SwinT-fairSIM show superiority over the Red-fairSIM and UNet-fairSIM methods. The resulting ROIs of both datasets from SwinT-fairSIM are more appealing and sharper than those of the other methods in Figure 6. The PSNR and SSIM values of SwinT-fairSIM are also slightly higher than those of Red-fairSIM and UNet-fairSIM. Overall, SwinT-

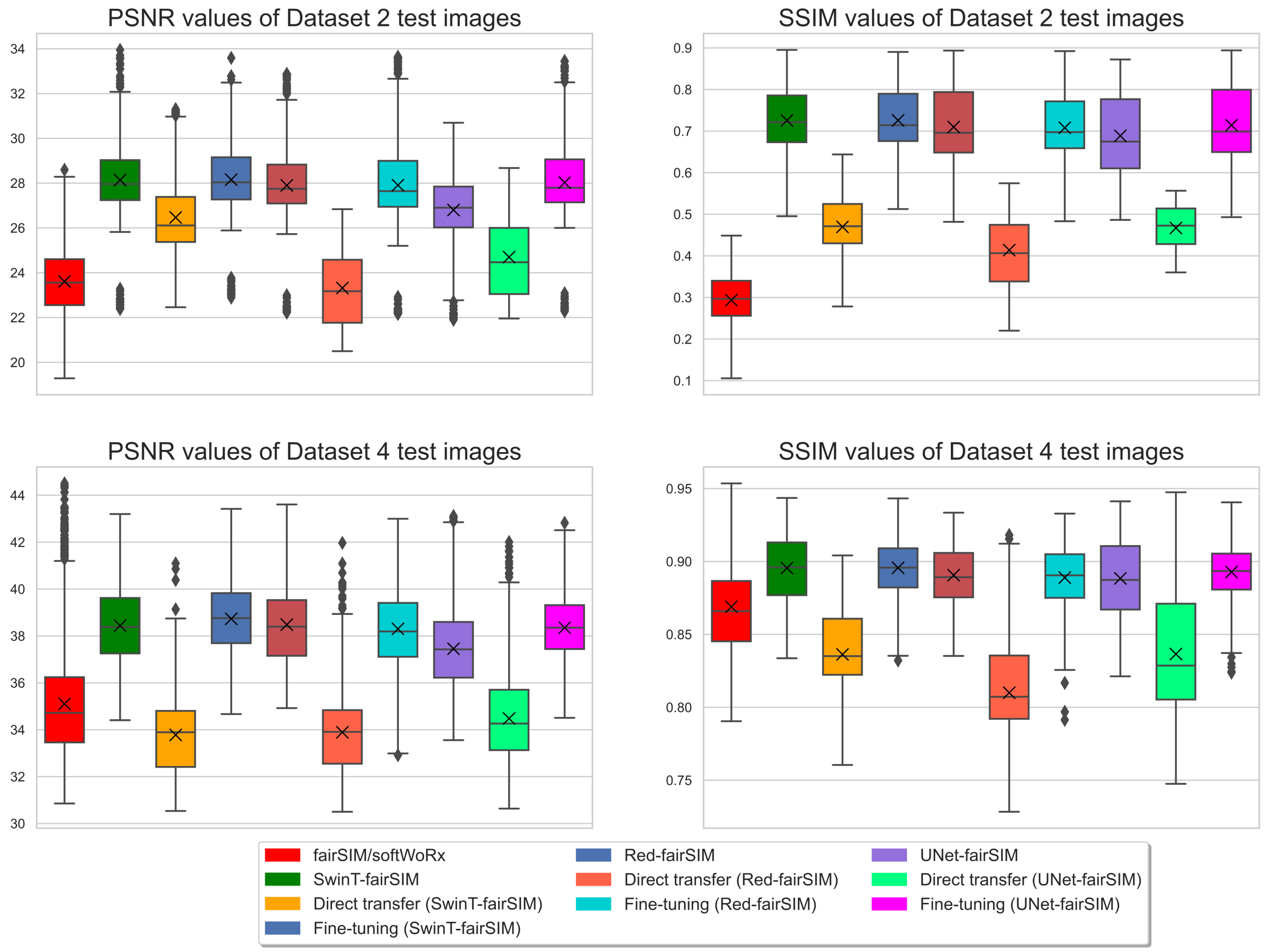

Figure 7. Boxplots of PSNR and SSIM values of test samples of dataset 2 and dataset 4 with different denoising algorithms and training strategies. The test data contains 500 images from noise level 4 for dataset 2 and 1380 images for dataset 4. The horizontal line inside each boxplot represent the median value, whereas, the black cross display the mean values. The diamond-shaped black markers display the outlier observations. The color of the boxes in each subplot refers to the methods defined in the legend box.

fairSIM outperforms its counterparts on both datasets 2 and 4 after conventional training from scratch.

In the next step, we evaluate the direct transfer strategy by using these pre-trained models. In direct transfer, the model initially trained with dataset 2 is tested with the test samples of an alternative dataset (i.e., dataset 4), and vice versa. Similarly, in the assessment of the fine-tuning strategy, we re-trained the first and last layers of the pre-trained models with the alternative data to see the improvement in the generalization power of the pre-trained models. The resulting denoised images obtained by direct transfer of models trained on vesicle structures (dataset 4) to test images containing tubulin filaments (dataset 2) are displayed within block **(a)** of Figures 8 and 9. These images clearly show that the model trained on a specific type of noise and structure (i.e., vesicle images with honey comb pattern noise) is not able to produce a refined denoised image of another structure (i.e., tubulin filaments with MPG noise). Similarly, the models initially trained on tubulin filaments from dataset 2 are not able to properly produce the denoised images of dataset 4 with different structure and noise (see block **(b)** of Figures 8 and 9). However, it can be noticed that the pre-trained models try to replicate the filamentous structure of tubulin in the vesicle data (very prominent in block **(b)** of Figure 8). This is a clear indication that these deep learning-based denoising models are not robust against different types of noise and structure. However, the outcomes of the fine-tuning approach are promising as shown in Figures 8 and 9. The ROIs 1 and 2 in column 6 of Figure 8 and also the ROIs in column 4 of Figure 9 show that the results of the fine-tuning strategy are very close to the results of training from scratch for the SwinT-fairSIM, Red-fairSIM and UNet-fairSIM algorithms.

The overall comparison of all the approaches in Figures 8 and 9 demonstrates that the fine-tuning method is crucial for the Transformer and CNN-based denoising algorithms in the case of changes in structure or noise types. Thus, fine-tuning is inevitably required to profit from knowledge transfer. Table 2 lists the average PSNR and SSIM values of the test images of the datasets 2 and 4 calculated for all the training and testing strategies of SwinT-fairSIM, Red-fairSIM, and UNet-fairSIM. Table 2 clearly points out the decline in the average PSNR and SSIM values on both datasets 2 and 4 after the application of direct transfer. However, the average PSNR and SSIM values show considerable improvement after fine-tuning. The average PSNR and SSIM values of the fine-tuned models in Table 2 are very close to the models that were trained from scratch (partly slightly better, partly slightly worse). In addition, Figure 7 shows boxplots of the PSNR and SSIM values of the test samples of both datasets with all different methods/strategies. The boxplots complement the mean values in Table 2 by depicting additional statistics. It can be observed that SwinT-fairSIM not only has often higher mean and median values, but also a

Table 2. Mean PSNR and SSIM values along with standard deviations (STD) of all experiments calculated on the noisy test images of datasets 2 and 4 (for dataset 2, noise level 4 was used). The test data contains 500 images for dataset 2 and 1380 images for dataset 4. The PSNR and SSIM values are calculated relative to the reference images, i.e. SR-SIM images reconstructed with fairSIM/softWoRx from raw SIM images with the highest signal-to-noise ratio. In the row entitled "fairSIM/softWoRx", PSNR and SSIM values are calculated for the direct fairSIM/softWoRx reconstruction of the noisy images. The rows entitled "SwinT-fairSIM","Red-fairSIM" and "UNet-fairSIM" show the results for the denoised test images after conventional training from scratch with the respective algorithms. In the direct transfer rows, the mean PSNR and SSIM values in the column "dataset 2" are calculated for models which are initially trained on dataset 4 and afterwards tested with the test samples from dataset 2. For the column "dataset 4", this is the other way round. The same principle holds for the rows for fine-tuning, only that the models are fine-tuned on the respective dataset before testing.

	Mean PSNR (STD) and SSIM (STD) values of test data			
	**dataset =2**		**dataset =4**	
	PSNR (STD)	SSIM (STD)	PSNR (STD)	SSIM (STD)
fairSIM/softWoRx	23.61 (1.54)	0.29 (0.07)	35.10 (2.71)	0.86 (0.03)
SwinT-fairSIM	28.19 (2.09)	0.72 (0.09)	38.44 (1.73)	0.89 (0.02)
Direct transfer (SwinT-fairSIM)	26.07 (1.91)	0.48 (0.07)	34.05 (2.24)	0.83 (0.03)
Fine-tuning (SwinT-fairSIM)	28.15 (1.87)	0.72 (0.08)	38.52 (1.50)	0.89 (0.02)
Red-fairSIM	27.97 (2.01)	0.71 (0.09)	38.43 (1.45)	0.89 (0.01)
Direct transfer (Red-fairSIM)	23.31 (1.68)	0.41 (0.07)	33.89 (1.93)	0.81 (0.03)
Fine-tuning (Red-fairSIM)	27.90 (2.14)	0.70 (0.09)	38.30 (1.60)	0.88 (0.02)
UNet-fairSIM	26.80 (1.65)	0.68 (0.10)	37.45 (1.79)	0.88 (0.02)
Direct transfer (UNet-fairSIM)	24.69 (1.54)	0.46 (0.05)	34.47 (2.24)	0.83 (0.04)
Fine-tuning (UNet-fairSIM)	28.02 (1.97)	0.71 (0.07)	38.35 (1.44)	0.89 (0.01)

more consistent interquartile range in all of the boxplots as shown in Figure 7. In the SSIM boxplots of dataset 2, very few outliers are noticable overall.

Compared to conventional training, the fine-tuning strategy retrains only a subset of the entire set of model parameters. In this study, we retrained about 2.1 million parameters out of 4 million parameters of SwinT-fairSIM, while for Red-fairSIM and UNet-fairSIM, out of more than 1 million and 33 million training parameters, only 295K and 3.7 million parameters were retrained, respectively. We were able to achieve results comparable to those of conventional training after only 30 epochs as opposed to 100 epochs. Training times for dataset 2 on two Nvida V100 GPUs (32 GB) were (training from scratch vs. fine-tuning):

SwinT-fairSIM: 26 h vs. 3.25 h
Red-fairSIM: 24 h vs. 3 h
UNet-fairSIM: 18 h vs. 1.5 h

Therefore, in terms of computational requirements or memory consumption, it is advantageous to use fine-tuning instead of training from scratch. Fine-tuning is beneficial when the computational resources are limited.

Discussion

In summary, the contribution of this work is threefold: First, we publish novel datasets related to SIM microscopy, then we explore a Transformer-based algorithm for the restoration of SR-SIM images. Finally, we investigate the potential of the direct transfer and fine-tuning strategies for various deep learning-based denoising algorithms. Regarding datasets, we provide four novel datasets for testing denoising and super-resolution image reconstruction strategies with tubulin filaments and vesicle structures. These datasets contain a large number of raw SIM images and reconstructed twofold super-resolved SIM images and cover different noise levels, a wide range of fields of view, and varying degrees of structural complexity. High-quality data from the real world of microscopy are highly relevant for benchmarking and evaluating current and upcoming denoising and super-resolution methods.

Regarding our work with Transformers, we suggested the SwinT-fairSIM architecture to produce high-quality SR-SIM images from low SNR inputs. Importantly, we visually and quantitatively showed that the Transformer-based method can achieve mostly better results than the CNN-based Red-fairSIM and UNet-fairSIM methods (i.e., for the quantitative results, PSNR and SSIM values, see Table 2 and the boxplots in Figure 7). We demonstrated that SwinT-fairSIM can retrieve more well-preserved cell structures and texture information than the CNN-based methods, especially in the ROIs of Figure 6. This is an important finding, since most existing SR-SIM restoration methods are based on CNNs [17, 18, 19, 21, 22, 23, 38, 39]. An explanation for the better performance of SwinT-fairSIM may be the size of the receptive fields of the different network architectures. In CNNs, the size of their receptive fields is limited by the size and stride of the filter kernels and the depth of the network. In contrast, Swin Transformers may obtain a larger receptive field by the shifted windows operation [57, 58].

In the last section of this work, we showed the limitations of Transformer- and CNN-based deep learning methods in the area of knowledge transfer when it comes to different types of noise and structure. In the direct transfer strategy, we simply evaluated the pre-trained networks with new test data. The direct transfer strategy exhibits very downgraded visual (in Figures 8 and 9) and quantitative (in Table 2 and Figure 7) results. However, the approach of fine-tuning enables these deep learning models to generalize well to other noise types and structures after retraining few layers. This holds for all learning algorithms and datasets in a similar way as shown in Figures 7, 8, 9 and in Table 2. All of the deep neural network architectures tested here are, at their core, encoder-decoder architectures (for SwinT-fairSIM, this applies at least to the

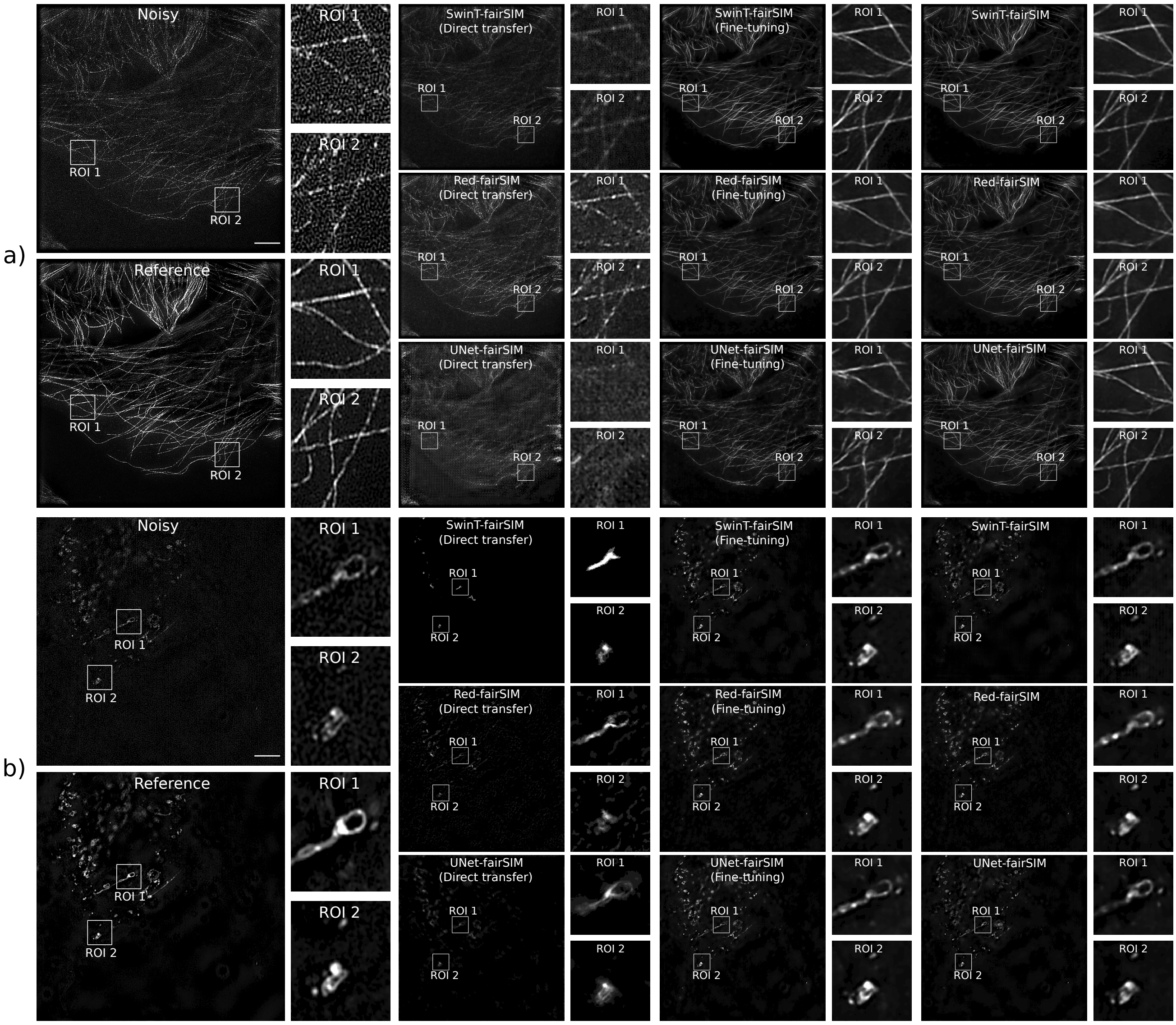

Figure 8. Blocks (**a**) and (**b**) show the results for test samples from dataset 2 (block **a**) and dataset 4 (block **b**) for different algorithms and learning strategies. The first and second columns of blocks **(a)** and **(b)** show the noisy and reference SR-SIM images, respectively, along with extracted and magnified regions of interest (ROIs). The noisy images are the test input for all algorithms. Columns 3 to 6 show the results of direct transfer and fine-tuning with SwinT-fairSIM, Red-fairSIM, and UNet-fairSIM. In direct transfer, the model is trained with dataset 2 and tested with the test sample of dataset 4 and vice versa. Similarly, in fine-tuning, the model is first pre-trained on dataset 2 and then fine-tuned and tested on dataset 4 and vice versa. Columns 7 and 8 show the results of all algorithms when trained from scratch on the respective dataset, which is also used for testing. The fourth, sixth, and eighth columns show the cropped and enlarged ROIs from the full-size SR-SIM images to the left of each ROI. The ROIs of size 100 pixels $\times$ 100 pixels have been upsampled to 300 pixels $\times$ 300 pixels for illustration purposes. Scale bar: 4 µm.

compression head block and the decompression tail block). In these architectures, the initial and final layers are responsible for handling low-level features. Noise patterns can be interpreted as low-level features that are separated from the valuable input during the encoding process so that they can be selectively suppressed at a later stage during decoding.

Accordingly, we retrained only the initial and final layers of all three types of neural networks to achieve results on par with training from scratch. It is worth mentioning that we also attempted to retrain only the first, middle, or last layers during first exploratory research, and also varied the number of retrained layers, but the results obtained in this way were not competitive. Since fine-tuning often saves computation time and often requires fewer learning samples, we conclude that fine-tuning of pre-trained models has advantages over conventional training from scratch, at least when the difference between the task domains is not huge. In contrast, direct transfer obviously fails because the learned detectors for low-level features (noise patterns and typical simple visual features of the main cell structures) are too much tuned to the original training data.

There are still some limitations of this work. First, while our datasets are useful for various image restoration tasks and contain more samples than many of the datasets previously published in the field, they are limited to two structures, tubulin filaments and vesicle structures. Second, our results indicate the superiority of SwinT-fairSIM over Red-fairSIM and UNet-fairSIM. However, this improvement leads to higher computational costs. Third, fine-tuning strategies provide similar results to training from scratch at lower cost, but it is often difficult to find task-specific pre-trained networks. Finally, slice-by-slice denoising of 3D SIM images focuses exclusively on the lateral plane; despite this limitation, it was shown that the presented approach can still be very useful for denoising 3D SIM structures.

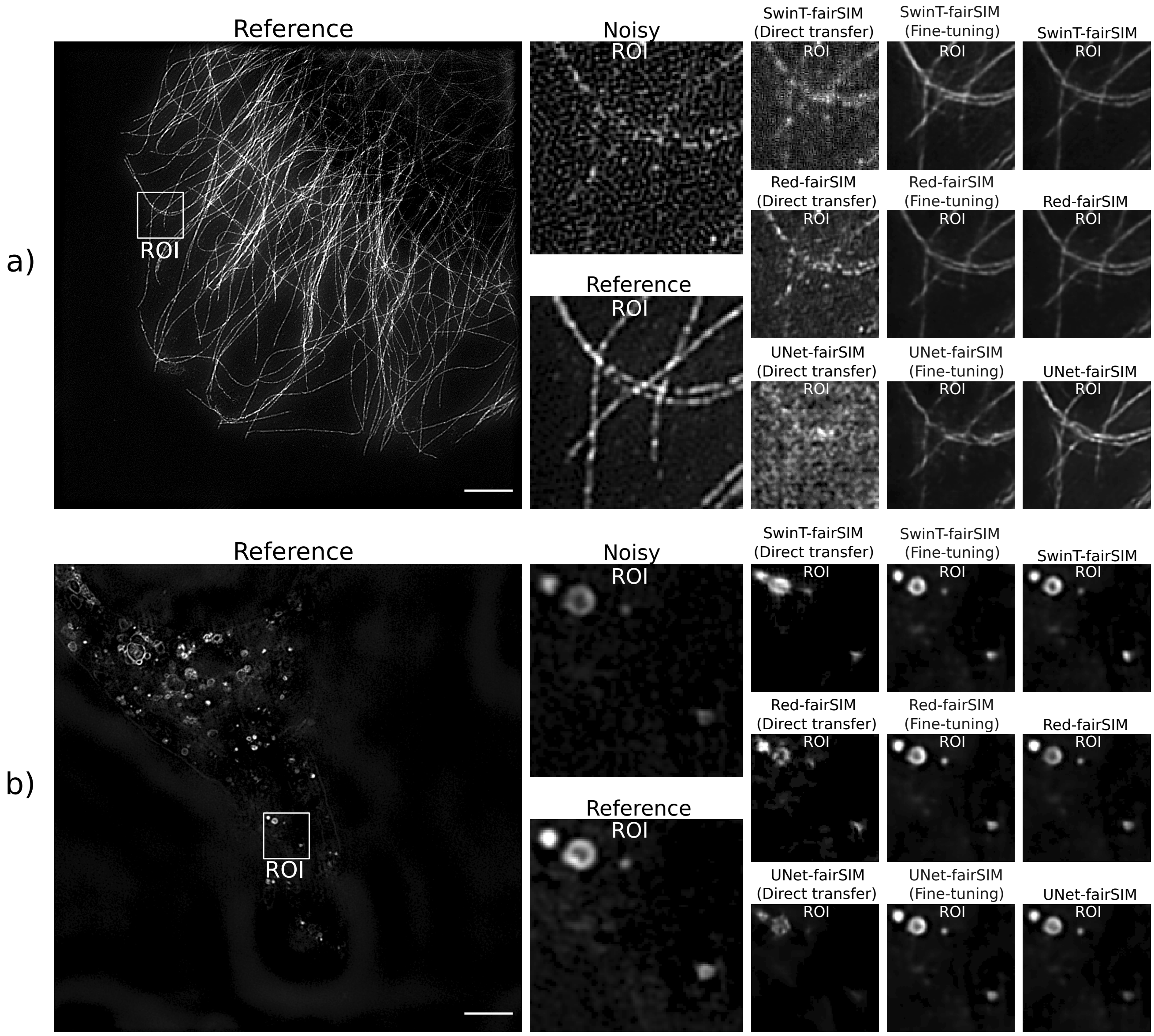

Figure 9. Two more test samples from datasets 2 and 4 are shown in this figure. Both blocks (**a**) and (**b**) contain the reference SR-SIM images along with the resultant denoised ROIs which are extracted from the full size denoised images of all the methods used in this work. The extracted ROIs are upsampled from 100 pixels × 100 pixels to 300 pixels × 300 pixels for illustration purposes. Scale bar: 4 µm.

Conclusion

In this work, we first presented a series of datasets for SIM image restoration tasks such as denoising and super-resolution. Additionally, we proposed a new algorithm based on the Swin Transformer, "SwinT-fairSIM", for the denoising of SR-SIM images. We showed that this Transformer-based algorithm outperforms CNN-based denoising algorithms both visually and quantitatively. This suggests that Transformer-based denoising algorithms can play an important role in the field of SR-SIM microscopy. Similarly, we evaluated different knowledge transfer strategies such as direct transfer and fine-tuning for Transformer- and CNN-based denoising algorithms. For the direct transfer strategy, we noticed a decline in the performance of these algorithms. However, we were able to recover the declined performance by the fine-tuning strategy. This clearly indicates that the Transformer- and CNN-based denoising methods require retraining of some initial and final layers of the pre-trained models when applied to new biological structures and noise types. This retraining requires fewer epochs than the conventional training from scratch. This holds also for the novel SwinT-fairSIM denoising algorithm which outperforms all CNN-based algorithm also in the fine-tuning regime.

Potential implications

The implementation of different deep learning methods requires a large quantity of images in order to train the underlying models. We believe that our published datasets will help the research community to develop new deep learning-based methods and evaluate the existing methods by either training from scratch or by applying fine-tuning. These datasets can be especially used for image denoising and super-resolution tasks.

Data availability

The raw and reconstructed SIM datasets described in this article are available via the GigaScience database (GigaDB) [45]. All

the datasets are distributed under the Creative Commons CC0 license. Similarly, all of the models trained during this work are freely accessible through [44].

Availability of supporting source code and requirements

- Project name:
  SwinT-fairSIM-and-knowledge-transfer [42]
- Project homepage:
  https://github.com/ZafranShah/SwinT-fairSIM-and-knowledge-transfer
- Operating system(s): Platform independent
- Programming language: Python
- Other requirements: Python 3.7.9 or higher, tensorflow-gpu 2.5.0 or higher, pillow 8.1.0, opencv-python 4.5.5.64
- License: GNU GPL
- Workflowhub.eu :
  https://doi.org/10.48546/WORKFLOWHUB.WORKFLOW.675.1
- RRID: SCR_024715

Declarations

Abbreviations

SIM: structured illumination microscopy; SR-SIM: super-resolution structured illumination microscopy; NA: numerical aperture; PSNR: peak-signal-to-noise ratio; SSIM: structural similarity index measurement; MSE: mean square error; SNR: signal-to-noise ratio; W2S: wide-field2SIM; fairSIM: free analysis and interactive reconstruction for structured illumination microscopy.

Competing Interests

The authors declare no competing interests.

Funding

This work was supported by the EFRE-NRW funding programme ”Forschungsinfrastrukturen” (grant no. 34.EFRE-0300180) and partly conducted within the framework of the project “SAIL: SustAInable Lifecycle of Intelligent SocioTechnical Systems” (grant no. NW21-059B). SAIL is receiving funding from the programme “Netzwerke 2021”, an initiative of the Ministry of Culture and Science of the State of Northrhine Westphalia. T.-C.W. and T.H. were supported by funding from the European Union’s Horizon 2020 research and innovation program under the Marie Sklodowska-Curie Grant Agreements No. 642157, project “TOLLerant”, and No. 766181. project "DeLIVER". T.H. also acknowledges funding by the Deutsche Forschungsgemeinschaft (DFG, German Science Foundation)—project number 415832635.

Author’s contributions

Z.H.S. carried out the preprocessing of datasets for the machine learning work, created the figures, and wrote the manuscript. D.T. conducted experiments to find the optimal hyperparameters for the CNN-based approaches. T.-C.W. recorded the raw SIM images of dataset 1. M.M. supported Z.H.S. in the reconstruction of data for datasets 2 and 3 with fairSIM. W.H. recorded and reconstructed the images of dataset 4. W.S. and T.H. supervised the research. W.S., T.H., M.M., and W.H. contributed to the editing of the manuscript. In addition, they discussed the experimental results together with Z.H.S. All authors discussed and agreed on the final manuscript.

Acknowledgements

The authors would like to thank Dr. Matthias Fricke from the Center for Applied Data Science (CfADS) at Bielefeld University of Applied Sciences and Arts for providing access to their GPU compute cluster. We would also like to thank Dr. Olaf Kaczmarek and Markus Klappenbach for providing access to the GPU compute cluster at Bielefeld University.

References

1. Hirvonen LM, Wicker K, Mandula O, Heintzmann R. Structured illumination microscopy of a living cell. European Biophysics Journal 2009;38(6):807–812.
2. Hell SW, Sahl SJ, Bates M, Zhuang X, Heintzmann R, Booth MJ, et al. The 2015 super-resolution microscopy roadmap. Journal of Physics D: Applied Physics 2015;48(44):443001.
3. Heintzmann R, Huser T. Super-resolution structured illumination microscopy. Chemical reviews 2017;117(23):13890–13908.
4. Demmerle J, Innocent C, North AJ, Ball G, Müller M, Miron E, et al. Strategic and practical guidelines for successful structured illumination microscopy. Nature protocols 2017;12(5):988–1010.
5. Schermelleh L, Ferrand A, Huser T, Eggeling C, Sauer M, Biehlmaier O, et al. Super-resolution microscopy demystified. Nature cell biology 2019;21(1):72–84.
6. Gustafsson MG. Surpassing the lateral resolution limit by a factor of two using structured illumination microscopy. Journal of microscopy 2000;198(2):82–87.
7. Müller M, Mönkemöller V, Hennig S, Hübner W, Huser T. Open-source image reconstruction of super-resolution structured illumination microscopy data in ImageJ. Nature communications 2016;7(1):1–6.
8. Lal A, Shan C, Xi P. Structured illumination microscopy image reconstruction algorithm. IEEE Journal of Selected Topics in Quantum Electronics 2016;22(4):50–63.
9. Brown PT, Kruithoff R, Seedorf GJ, Shepherd DP. Multicolor structured illumination microscopy and quantitative control of polychromatic light with a digital micromirror device. Biomedical Optics Express 2021;12(6):3700–3716.
10. Ströhl F, Kaminski CF. Frontiers in structured illumination microscopy. Optica 2016;3(6):667–677.
11. Zheng X, Zhou J, Wang L, Wang M, Wu W, Chen J, et al. Current challenges and solutions of super-resolution structured illumination microscopy. APL Photonics 2021;6(2):020901.
12. Huang X, Fan J, Li L, Liu H, Wu R, Wu Y, et al. Fast, long-term, super-resolution imaging with Hessian structured illumination microscopy. Nature biotechnology 2018;36(5):451–459.
13. Hoffman DP, Betzig E. Tiled reconstruction improves structured illumination microscopy. BioRxiv 2020;.
14. Smith CS, Slotman JA, Schermelleh L, Chakrova N, Hari S, Vos Y, et al. Structured illumination microscopy with noise-controlled image reconstructions. Nature methods 2021;18(7):821–828.
15. Gustafsson MG, Shao L, Carlton PM, Wang CR, Golubovskaya IN, Cande WZ, et al. Three-dimensional resolution doubling in wide-field fluorescence microscopy by structured illumination. Biophysical journal 2008;94(12):4957–4970.
16. Shah ZH, Müller M, Hammer B, Huser T, Schenck W. Im-

pact of different loss functions on denoising of microscopic images. In: 2022 International Joint Conference on Neural Networks (IJCNN) IEEE; 2022. p. 1–10.
17. Jin L, Liu B, Zhao F, Hahn S, Dong B, Song R, et al. Deep learning enables structured illumination microscopy with low light levels and enhanced speed. Nature communications 2020;11(1):1–7.
18. Chen X, Li B, Jiang S, Zhang T, Zhang X, Qin P, et al. Accelerated Phase Shifting for Structured Illumination Microscopy based on Deep Learning. IEEE Transactions on Computational Imaging 2021;7:700–712.
19. Shah ZH, Müller M, Wang TC, Scheidig PM, Schneider A, Schüttpelz M, et al. Deep-learning based denoising and reconstruction of super-resolution structured illumination microscopy images. Photonics Research 2021;9(5):B168–B181.
20. Belthangady C, Royer LA. Applications, promises, and pitfalls of deep learning for fluorescence image reconstruction. Nature methods 2019;16(12):1215–1225.
21. Qiao C, Li D, Guo Y, Liu C, Jiang T, Dai Q, et al. Evaluation and development of deep neural networks for image super-resolution in optical microscopy. Nature Methods 2021;18(2):194–202.
22. Xypakis E, Gosti G, Giordani T, Santagati R, Ruocco G, Leonetti M. Deep learning for blind structured illumination microscopy. Scientific Reports 2022;12(1):8623.
23. Liu T, Liu J, Li D, Tan S. Improving Reconstruction of Structured Illumination Microscopy Images Via Dual-Domain Learning. IEEE Journal of Selected Topics in Quantum Electronics 2023;.
24. Qiao C, Chen X, Zhang S, Li D, Guo Y, Dai Q, et al. 3D structured illumination microscopy via channel attention generative adversarial network. IEEE Journal of Selected Topics in Quantum Electronics 2021;27(4):1–11.
25. Vaswani A, Shazeer N, Parmar N, Uszkoreit J, Jones L, Gomez AN, et al. Attention is all you need. Advances in neural information processing systems 2017;30.
26. Sutskever I, Vinyals O, Le QV. Sequence to sequence learning with neural networks. Advances in neural information processing systems 2014;27.
27. Dehghani M, Gouws S, Vinyals O, Uszkoreit J, Kaiser L. Universal Transformers. In: International Conference on Learning Representations;. .
28. Vyas A, Katharopoulos A, Fleuret F. Fast transformers with clustered attention. Advances in Neural Information Processing Systems 2020;33:21665–21674.
29. Wang B, Shang L, Lioma C, Jiang X, Yang H, Liu Q, et al. On position embeddings in bert. In: International Conference on Learning Representations; 2021. .
30. Cheng X, Lin H, Wu X, Shen D, Yang F, Liu H, et al. Mltr: Multi-label classification with transformer. In: 2022 IEEE International Conference on Multimedia and Expo (ICME) IEEE; 2022. p. 1–6.
31. Carion N, Massa F, Synnaeve G, Usunier N, Kirillov A, Zagoruyko S. End-to-end object detection with transformers. In: Computer Vision–ECCV 2020: 16th European Conference, Glasgow, UK, August 23–28, 2020, Proceedings, Part I 16 Springer; 2020. p. 213–229.
32. Sun Z, Cao S, Yang Y, Kitani KM. Rethinking transformer-based set prediction for object detection. In: Proceedings of the IEEE/CVF international conference on computer vision; 2021. p. 3611–3620.
33. Chen H, Wang Y, Guo T, Xu C, Deng Y, Liu Z, et al. Pre-trained image processing transformer. In: Proceedings of the IEEE/CVF Conference on Computer Vision and Pattern Recognition; 2021. p. 12299–12310.
34. Ali AM, Benjdira B, Koubaa A, El-Shafai W, Khan Z, Boulila W. Vision transformers in image restoration: A survey. Sensors 2023;23(5):2385.
35. Liu Z, Lin Y, Cao Y, Hu H, Wei Y, Zhang Z, et al. Swin transformer: Hierarchical vision transformer using shifted windows. In: Proceedings of the IEEE/CVF international conference on computer vision; 2021. p. 10012–10022.
36. Liang J, Cao J, Sun G, Zhang K, Van Gool L, Timofte R. Swinir: Image restoration using swin transformer. In: Proceedings of the IEEE/CVF international conference on computer vision; 2021. p. 1833–1844.
37. Zhang Y, Zhu Y, Nichols E, Wang Q, Zhang S, Smith C, et al. A poisson-gaussian denoising dataset with real fluorescence microscopy images. In: Proceedings of the IEEE/CVF Conference on Computer Vision and Pattern Recognition; 2019. p. 11710–11718.
38. Zhou R, Helou ME, Sage D, Laroche T, Seitz A, Süsstrunk S. W2S: microscopy data with joint denoising and super-resolution for widefield to SIM mapping. In: European Conference on Computer Vision Springer; 2020. p. 474–491.
39. Hagen GM, Bendesky J, Machado R, Nguyen TA, Kumar T, Ventura J. Fluorescence microscopy datasets for training deep neural networks. GigaScience 2021;10(5):giab032.
40. Karras C, Smedh M, Förster R, Deschout H, Fernandez-Rodriguez J, Heintzmann R. Successful optimization of reconstruction parameters in structured illumination microscopy–a practical guide. Optics Communications 2019;436:69–75.
41. Mueller M, Free Analysis and Interactive Reconstruction for Structured Illumination Microscopy. GitHub; 2016. https://github.com/fairSIM/fairSIM.git.
42. Shah ZH, Evaluation of Swin Transformer and knowledge transfer for denoising of super-resolution structured illumination microscopy data. GitHub; 2023. https://github.com/ZafranShah/SwinT-fairSIM-and-knowledge-transfer.
43. "Evaluation of Swin Transformer and knowledge transfer for denoising of super-resolution structured illumination microscopy data". WorkflowHub. WorkflowHub.eu; 2023. https://doi.org/10.48546/WORKFLOWHUB.WORKFLOW.675.1.
44. Shah ZH, Evaluation of Swin Transformer and knowledge transfer for denoising of super-resolution structured illumination microscopy data. zenodo; 2023. https://doi.org/10.5281/zenodo.7626173.
45. Shah ZH, Müller M, Hübner W, Wang T, Telman D, Huser T, et al., Supporting data for "Evaluation of Swin Transformer and knowledge transfer for denoising of super-resolution structured illumination microscopy data". GigaScience Database; 2023. http://dx.doi.org/10.5524/102461.
46. Ching JY, Wong AKC, Chan KCC. Class-dependent discretization for inductive learning from continuous and mixed-mode data. IEEE Transactions on Pattern Analysis and Machine Intelligence 1995;17(7):641–651.
47. Hussain M, Bird JJ, Faria DR. A study on cnn transfer learning for image classification. In: UK Workshop on computational Intelligence Springer; 2018. p. 191–202.
48. Tan C, Sun F, Kong T, Zhang W, Yang C, Liu C. A survey on deep transfer learning. In: International conference on artificial neural networks Springer; 2018. p. 270–279.
49. Bengio Y. Deep learning of representations for unsupervised and transfer learning. In: Proceedings of ICML workshop on unsupervised and transfer learning JMLR Workshop and Conference Proceedings; 2012. p. 17–36.
50. Ng HW, Nguyen VD, Vonikakis V, Winkler S. Deep learning for emotion recognition on small datasets using transfer learning. In: Proceedings of the 2015 ACM on international conference on multimodal interaction; 2015. p. 443–449.
51. Nogueira K, Penatti OA, Dos Santos JA. Towards better exploiting convolutional neural networks for remote sensing

scene classification. Pattern Recognition 2017;61:539–556.
52. Yosinski J, Clune J, Bengio Y, Lipson H. How transferable are features in deep neural networks? Advances in neural information processing systems 2014;27.
53. Mao X, Shen C, Yang YB. Image restoration using very deep convolutional encoder-decoder networks with symmetric skip connections. Advances in neural information processing systems 2016;29.
54. Ronneberger O, Fischer P, Brox T. U-net: Convolutional networks for biomedical image segmentation. In: International Conference on Medical image computing and computer-assisted intervention Springer; 2015. p. 234–241.
55. Hore A, Ziou D. Image quality metrics: PSNR vs. SSIM. In: 2010 20th international conference on pattern recognition IEEE; 2010. p. 2366–2369.
56. Setiadi DRIM. PSNR vs SSIM: imperceptibility quality assessment for image steganography. Multimedia Tools and Applications 2021;80(6):8423–8444.
57. Parmar N, Vaswani A, Uszkoreit J, Kaiser L, Shazeer N, Ku A, et al. Image transformer. In: International conference on machine learning PMLR; 2018. p. 4055–4064.
58. Ranftl R, Bochkovskiy A, Koltun V. Vision transformers for dense prediction. In: Proceedings of the IEEE/CVF International Conference on Computer Vision; 2021. p. 12179–12188.
