# Supplementary material for: Evaluation of Swin Transformer and knowledge transfer for denoising of super-resolution structured illumination microscopy data
Source: Gigascience. 2024 Jan 13;13:giad109. doi: 10.1093/gigascience/giad109 (PMC10787368; doi:10.1093/gigascience/giad109)
Supplement: giad109_Response_to_Reviewer_Comments_Original_Submission [file giad109_response_to_reviewer_comments_original_submission.pdf]

Author's Response To Reviewer Comments

Dear Editor,

We thank you and the reviewers for the comments on our manuscript entitled, “Evaluation of Swin Transformer and knowledge transfer for denoising of super-resolution structured illumination microscopy data”. We appreciate the suggestions of the reviewers to strengthen our revised manuscript. We also hope that our edits and the responses we provide below satisfactorily address all your major and minor concerns. Furthermore, we have added in response to one of the reviewers a new Transformer-based SR-SIM image denoising algorithm. Therefore, we have updated the title of the manuscript accordingly. Because of this considerable extension of the presented work, we would like to request to change the category of the manuscript from technical note to research.

All the results at GigaDB are updated and the new code is uploaded to the GitHub repository. We believe that the manuscript is now suitable for publication in GigaScience.

###############################Reviewer # 1
##################################

Comment-1. To our knowledge, the "BioSR" dataset presented in reference [22] contains quite a few SIM reconstructions. While, the authors declare that "However, their collection of datasets consists of considerably fewer images and does not contain any super-resolution SIM images". The authors may do a detailed survey of the "BioSR" data set.

Response-1. We revised the text in this paragraph to clarify our statements and to provide some additional details about the BioSR dataset. Actually, the sentence "However, their collection of datasets consists of considerably fewer images and does not contain any super-resolution SIM images" was not intended to refer to the BioSR dataset. Hopefully, our writing is now better understandable.
As a side note: In comparison to the BioSR dataset collection with 2200 image pairs, our datasets consist of more than 9800 pairs of low- and high-resolution samples of 1024 x 1024 (width x height) pixels with different time stamps and noise levels. Furthermore, our datasets are ready to be used for different image restoration problems.

Comment -2. The authors may modify the bottom "(FFT)" in the "SIM Algorithm" box of Figure 1 as "(Inverse FFT)".

Response-2. We thank the reviewer and changed the respective figure.

Comment -3. We can see obvious artifacts and backgrounds in the reference images (Figure 2, magnified). Can the reference image quality be promoted further?

Response-3. All the reference images are generated using the fairSIM and SoftWorX reconstruction algorithms. These artifacts are generated by these SIM reconstruction algorithms, that is the reason we have developed our machine learning based tools that can be trained to remove such artifacts. The standard reference images can, however, not be further improved based on the traditionally used purely Wiener-filter-based algorithms. Due to this, we labeled our high-resolution output images as reference images instead of ground truth.

Comment -4. The UNet-fairSIM output in Figure 5b seems to be unreasonable and the authors may try to explain this phenomenon and solve the problem.

Response-4. The UNet-fairSIM method was not able to denoise that specific image properly but we have replaced that sample with another test sample in Figure 9 of the revised manuscript which is slightly more in favor of UNet. However, considering original Figure 5b and new Figure 9, the only condition where the UNet is completely lacking is a direct transfer from training on dataset 4 (vesicles) to inference on dataset 2 (tubulin). In our view, this is not unreasonable. As the good results for this UNet for training from scratch on dataset 4 show, this UNet was properly trained before direct transfer. Therefore, the bad results for direct transfer mainly show that the UNet is not suited for this strategy.

Comment -5. Datasets 1-3 in Table 1 contain the same sample structures and may be combined into one dataset. By the way, the authors may provide other sample structures besides tubulin filaments and vesicles.

Response-5. The idea behind datasets 1-3 is to use these datasets of real world biological structures for different image restoration tasks. For example, dataset 1 can be used to denoise raw SIM data, dataset 2 can be used to denoise SR-SIM data, and dataset 3 can be used for super-resolution tasks or joint denoising and super-resolution tasks. In our view, this is a service to the potential user. The datasets are pre-structured in such a way that they can easily be used for these different tasks.

Comment -6. The authors can transfer Table 2 into the statistical boxplot for an intuitive representation.

Response-6. We thank the reviewer for this suggestion, we added the boxplots of the respective PSNR and SSIM values of all the findings in Figure 7 of the revised manuscript.

Comment -7. There are many deep learning-based SIM reconstruction methods like DFCAN/DFGAN, the authors may try these methods besides Red-fairSIM/Unet-fairSIM.

Response-7. In our revised manuscript, we propose a novel SwinT-fairSIM architecture based on the Swin Image Restoration (SwinIR) algorithm to denoise the SR-SIM images. Further details are explained in the revised manuscript (for details please see section “Shifted window Transformer for the denoising of SR-SIM images (SwinT-fairSIM)”). In this way, we hope to meet the demands of the reviewer by including an additional state-of-the-art deep learning-based image restoration technique in our comparison.

Comment -8. The authors may provide some discussions about "why the fine-tuning output is better than direct transfer?". Does this mean that the first/last few layers of the network are critical to its output?

Response-8. “Direct transfer” is the transfer of trained weights from one dataset to another dataset of the related task, whereas, in fine-tuning some of the layers of the pretrained models are retrained instead of training the entire model from scratch. We showed in the manuscript that direct transfer of weights from tubulin to vesicle data or vice versa results in a poor outcome. Fine-tuning is crucial to achieve competitive results. In the discussion section, we added a paragraph in which the critical role of the first/last few layers is discussed in light of these results.Comment -9. The authors may provide a summary and conclusion about the work.

Response-9. We added the conclusion section in the revised manuscript.

####################### Reviewer # 2 ##

Comment-1. In the abstract, on the computer vision side, there is a large number of methods that can blindly reduce the image noise level. Also, for SIM data, there are already methods for denoising multiple structures.

Response-1. In response to this comment, we added a sentence about a Blind-SIM method (BS-CNN architecture) in the second paragraph of the introduction section with reference number [22] to the revised manuscript. We agree that blind denoising is an interesting and relevant area on its own, but we think that it is beyond the scope of the presented work. Actually, in our previous work [19], we used a blind denoising algorithm from classical computer vision in comparison to deep learning-based approaches. The deep-learning approaches, trained to a specific task and data, were clearly superior. Therefore, we decided to focus here on algorithms which are trained in the first place with specific data and tackle the research questions, how well they generalize, and what can help in improving their generalization capabilities (i.e., fine-tuning).

Comment-2. On page 2, 'leads to low SNR', the abbreviation needs to be explained.

Response-2. We thank the reviewer, and we have addressed this concern in the revised manuscript.

Comment -3. On page 2, the authors compare existing open-source datasets and enumerate the amount of data in them, so it is better to introduce the dataset presented in this paper with the amount as well.

Response-3. We describe the characteristics of the datasets along with the total number of samples in Table 1 of the revised manuscript.

Comment -4. In SIM data, the data type is 16bit per pixel, however, the denoising method, i.e. RED was originally designed for 8bit images. In addition, the PSNR calculation in the author's code calls TensorFlow's built-in functions (tf_psnr) , has the corresponding modification been made for dynamic range, or has the data been pre-processed?

Response-4. We scaled the images from 16-bit to floating values (i.e., all the pixel values are between 0.0 and 1.0) before the training process. The PSNR values are calculated in the floating point representation. The maximum intensity required as a parameter for the PSNR calculation is adjusted accordingly. After the prediction, the images are converted back to 16-bit for storage and visualization, etc.

Comment-5. The code is publicly available, but the data is not, and it is recommended that some data be made available for testing.

Response-5. We already submitted our data to the GigaScience Database, so that they are publicly available for the research community after acceptance of the manuscript.

Comment-6. How is the distance represented by each pixel (scale bar) derived in the image? For example, 80 nm in Table 1 and 8 μm in Fig.1 and 2. I suggest adding the corresponding explanation.

Response-6. 80nm and 40nm refers to the pixel sizes in the raw images (80 nm/pixel) and reconstructed images (40 nm/pixel) which are mentioned in Table 1 of the manuscript. 80 nm/pixel arises as the combination of the magnification of the optical system (microscope) and the physical pixel size of the camera. The SIM reconstruction process then doubles the number of pixels, which halves their size, as part of the frequency-spaced reconstruction step. 4μm refers to the length of the scale bars, which is set in relation to the pixel size (by a built-in ImageJ function). Scale bars are now consistently set to 4μm.

Comment -7. How is noise level defined? In the domain of machine learning, level denotes the standard deviation of Gaussian noise, which needs to be disambiguated here.

Response-7. The datasets which are presented in this manuscript are collected from a real biological sample and contain real noise which is a mixture of Gaussian-Poisson noise plus the reconstruction artifacts created by the SIM reconstruction algorithm. Therefore, these real datasets do not have any artificial or

randomly added noise (such as Gaussian or Poisson with some specific intensity level). The real noise occurs due to the detection of fewer photons from biological samples, resulting in a bad signal-to-noise ratio. However, the timestamp is considered to be a relevant factor in these datasets to distinguish the level of noise in the images. For example, in datasets 1-3, small timestamps represent low noise levels, whereas large timestamps represent high noise levels as shown in Figure 3. The noise levels are explained in the subsection on “Dataset 2” in the revised manuscript.

############################## Reviewer # 3 ##############################

Comment -1. Firstly, datasets 1-3 are based on 2D SIM reconstruction, whereas dataset 4 is based on 3D SIM reconstruction, which leads to different axial resolutions, making it difficult to test the generalization capability of the model.Response-1. The raw data of all data sets are true 3D-SIM data acquisitions. The only difference is in the way how the reconstructions are obtained. fairSIM-based reconstructions use the 15 raw images of a particular focus position to reconstruct a single slice (which we call a 2D reconstruction), while the softWoRx reconstruction always reconstructs a full 3D data set. While a full 3D reconstruction provides
higher axial resolution, optical sectioning, i.e. the capability of removing blurred out-of-focus light, can be and is achieved by both 2D and 3D reconstruction. More importantly, the lateral resolution increase is the same for 2D and 3D SIM reconstruction. This makes single-slice (2D) SIM reconstructions and full 3D SIM reconstructions similar enough to work in the same processing pipeline. During this work, we
train only 2D models with both datasets 2 and 4. Each z-plane in dataset 4 is treated as a different field-of-view (FOV). This terminology results in the conversion of dataset 4 from a 3D to a 2D dataset. Furthermore, 15 raw SIM images were taken for each plane during the collection of all data, which consists of three pattern orientations and five equally distributed phases for each angle. This rules out
the different axial resolution point.

Comment -2. Secondly, the noise level has only four levels, and the sample types are limited to two structures, which is significantly less than the 'BioSR' dataset cited as a reference in the manuscript.

Response-2. This concern is already addressed above (see Reviewer # 1, comment-1, and comment-5). The datasets provided by us have their own merits such as the large number of samples and being especially structured for common use cases in benchmarking and research.

Comment -3. Lastly, the authors need to provide a schematic of the fine-tuning strategies they used.

Response-3. We thank the reviewer for this suggestion; we now show the schematic of direct transfer and fine-tuning in Figure 5 of the revised manuscript.

Note: A pdf file attached named 'Cover_Rebuttal_GIGA-D-23-00044.pdf file' also contains all the responses.
