# Supplementary material for: Evaluation of Swin Transformer and knowledge transfer for denoising of super-resolution structured illumination microscopy data
Source: Gigascience. 2024 Jan 13;13:giad109. doi: 10.1093/gigascience/giad109 (PMC10787368; doi:10.1093/gigascience/giad109)
Supplement: giad109_Response_to_Reviewer_Comments_Revision_1 [file giad109_response_to_reviewer_comments_revision_1.pdf]

Author's Response To Reviewer Comments

Title: Evaluation of Swin Transformer and knowledge transfer for denoising of super-resolution structured illumination microscopy data

Journal Name: GigaScience
Manuscript Number: GIGA-D-23-00044

Dear Dr. Zauner,

Thank you once again for your and the reviewers' appreciation and valuable comments on our manuscript entitled, "Evaluation of Swin Transformer and knowledge transfer for denoising of super-resolution structured illumination microscopy data". We have carefully revised the manuscript according to the suggested changes. The changes in the manuscript text are highlighted by the yellow highlighter. The point-by-point responses are included in the response box.

Regarding the main concern of reviewer #3: We agree with you and reviewer #3 that the data acquired and processed using the 3D-SIM illumination and algorithm has higher axial resolution than 2D-SIM to some extent. However, the main objective of this work was to remove the noise and reconstruction artifacts from the lateral plane of 2D and 3D SIM data. The 2D slices were obtained from the 3D-SIM data using a slice-by-slice strategy. As a result, the network solely denoises the lateral planes of both types of data.

We are also grateful for your time and effort in discussing the matter with your experts. We hope that our responses address the concerns of the reviewers. We believe that the manuscript is now suitable for publication in GigaScience.

All the results at GigaDB are updated and the new code is uploaded to the GitHub repository.

#####Reviewer # 1#####

Comment #1: In the "Response-5", the authors declared that "dataset 1 can be used to denoise raw SIM data, dataset 2 can be used to denoise SR-SIM data, and dataset 3 can be used for super-resolution tasks or joint denoising and super-resolution tasks." While, dataset 1 contains the functionality provided by datasets 2-3 because the users can utilize the fairSIM and SoftWorX for reconstructing the SR-SIM images in datasets 2-3 from the raw SIM data in dataset 1. And the authors may provide other sample structures besides tubulin filaments and vesicles in the form of dataset 1.

Response #1: Thank you again for your review and constructive feedback. We believe your last suggestion is very useful, but our current circumstances limit us to these two available sample structures (i.e., tubulin filaments and vesicles). Nevertheless, we believe that these structures can be very useful for the development, evaluation, and validation of future Deep Learning-based image recovery methods in the field of SIM microscopy. In addition, we would like to point out that datasets 2 and 3 may be very useful for readers who come from the machine learning field and do not yet have experience using fairSIM or SoftWorX.

#####Reviewer # 2#####

Comment #1: The authors mentioned the pixel size in their response, but I would like to see a more

detailed explanation in the paper about the relationship between pixel size and the external parameters of the microscope setup. This is crucial information for the dataset.

Response #1: Thank you for the review and the appreciation in your feedback. The choice of pixel size in a microscope depends on two main factors: the camera pixel size, and the total magnification. Higher resolutions are achieved by using smaller camera pixels and stronger magnifications, but the former at the expense of sensitivity (larger pixels are more sensitive) and the latter at the cost of field of view (higher magnifications decrease the imaging area). The OMX microscope uses a scientific camera for wide-field detection. Thus, the effective (aka projected) pixel size in the sample plane is given by the physical pixel size of the camera and the magnification of the optical system, i.e. the combination of the objective lens and any intermediate optics. For the OMX, the camera pixel size is 6.5 μm and the magnification is 82x, yielding an effective pixel size of 6.5 μm/82 = 80nm. The choice of magnification and thus effective pixel size is not arbitrary for a high-resolution imaging system. The imaging system has an inherent maximum spatial resolution that can be detected (Abbe limit), for a sample in water (n=1.33) and at blue wavelength (425nm) which is 160nm. Since detection by a pixelated camera is a discrete sampling process, the Nyquist criterion is now used to determine the maximum pixel size allowed to fully sample a signal with 160nm spatial resolution, which is 80nm. Larger pixels would miss high-frequency information that the optical system still transmits. Smaller pixels would fully sample all information, but introduce additional noise, as each pixel contributes an amount of read noise, and thus the total number of pixels should be kept small. More effects, especially dynamic range, can enterinto a pixel size determination, but this is the basic process. As a commercial microscope system, the pixel size of the OMX is constrained by the manufacturer to 80 nm, but it is likely the follow the above reasoning.

A shorter version of this explanation is now included in the manuscript at the beginning of "Materials and Methods – SR-SIM microscopy and sample preparation".

Comment #2: If the noise was not added to the image, how were the variables controlled to ensure a consistent noise level in each capture? In low-light conditions, changes in camera parameters can significantly affect the level of noise in the captured images.

Response #2: Several imaging parameters such as exposure time and illumination intensity can be adjusted in SIM microscopy to obtain samples with a high signal-to-noise ratio. Higher photon counts (N) typically result in samples with a higher signal-to-noise ratio. In SIM imaging, we typically control for two types of noise: Poisson noise, that is the variation in the number of photons being converted into photo-electrons in each pixel, and thermal and read noise, summarizing the error in counting the photo-electrons in the digitization process. The latter, the error introduced every time when reading out the camera, is very reproducible as long as two parameters are controlled for Camera temperature (influencing thermal and sometimes read noise) and exposure time (influencing mostly thermal noise). In the data sets at hand, we thus kept constant exposure time and temperature of the camera system in the OMX by cooling liquid.
In contrast, Poisson noise is a property of both illumination intensity and the specific sample. We can and do control illumination intensity, but the sample-to-sample variations (different effectiveness of the staining, variation in photobleaching) cannot be controlled. Therefore, we believe that these variations should be part of the datasets, since they are to be expected in real data and denoising networks must be able to deal with them.

Some remarks addressing the different types of noise are now included in the manuscript at the end of "Materials and Methods – SR-SIM microscopy and sample preparation".

#####Reviewer # 3#####

Comment #1: In the revised version, the manuscript's quality has seen notable improvements, particularly due to the incorporation of results obtained using the Transformer-based SR-SIM image denoising

algorithm. However, it remains concerning that the authors have not adequately addressed my comments, resulting in persisting weaknesses within the manuscript. One significant concern is that, regardless of whether 9 or 15 raw SIM images are employed in 2D reconstruction, the axial resolution consistently falls short compared to that achieved in 3D reconstruction. Consequently, the statement asserting that "this rules out the different axial resolution difference" is entirely inaccurate. Due to these unresolved issues, I am unable to ascertain the model's generalization capability across the four datasets. Moreover, the 'BioSR' dataset and other publicly available data showcasing new deep learning techniques for SIM denoising and reconstruction seem to overshadow the significance of the four datasets presented in this manuscript. Given the low image quality demonstrated within the manuscript, my confidence in the datasets' usefulness for the benefit of the scientific community has significantly diminished.

Response #1: Thank you for your review. Our previous statement "this rules out the different axial resolution difference" was unfortunately misleading. We fully agree that when a 3D SIM dataset is acquired and processed by 3D SIM algorithms, a higher axial resolution can be expected than for 2D SIM reconstruction (to some extent independent of the acquisition mode), since the axial resolution in SIM data depends on the illumination and the SIM reconstruction scheme [Gustafsson 2008]. Nevertheless, the SIM illumination and reconstruction algorithm for 3D SIM produces more noise and pattern artifacts in the lateral (XY) direction than in the axial (Z) direction [Gustafsson 2008]. Thus, a slice-by-slice (XY) strategy can still be useful to remove noise and artifacts introduced laterally by the reconstruction process for 3D SIM data.
In our work, we focus exclusively on the lateral plane and compare the generalization ability of denoising networks of 2D and 3D SIM microscopy data in the lateral plane using individual slices. An extension into the axial direction is out of the scope of the presented work and was not intended. We improved the manuscript by making this point clear at several locations:
• End of subsection “Dataset preprocessing and image reconstruction”
• Description of dataset 4
• New final sentence of the discussion section
As for the image quality in the datasets, we tend to disagree here, with all due respect. The images are of the usual quality that can be expected as the output of a SIM acquisition and processing toolchain for the intended cell structures. In particular, the reference images with high signal-to-noise ratios clearly show this.

#####Reference#####

[Gustafsson 2008]: Gustafsson, Mats GL, et al. "Three-dimensional resolution doubling in wide-field fluorescence microscopy by structured illumination." Biophysical journal 94.12 (2008): 4957-4970.
