# Supplementary material for: Evaluation of Swin Transformer and knowledge transfer for denoising of super-resolution structured illumination microscopy data
Source: Gigascience. 2024 Jan 13;13:giad109. doi: 10.1093/gigascience/giad109 (PMC10787368; doi:10.1093/gigascience/giad109)
Supplement: giad109_Response_to_Reviewer_Comments_Revision_2 [file giad109_response_to_reviewer_comments_revision_2.pdf]

Author's Response To Reviewer Comments

Title: Evaluation of Swin Transformer and knowledge transfer for denoising of super-resolution structured illumination microscopy data
Journal Name: GigaScience
Manuscript Number: GIGA-D-23-00044

Dear Dr. Zauner,

Thank you once again for your efforts. Similarly, a special thanks to the GigaDB team for their cooperation and efforts in data publication and release. We carefully revised the manuscript to reflect the necessary changes. The editorial points are addressed below:

1) Please structure your abstract (Background - Results - Conclusions)

- We have restructured the abstract into these three parts.

2) Please add ORCIds to the title page (don't worry about the format for now - just a list in plain text is fine). We have these on our files: ORCID iDs: Zafran Hussain Shah [0000-0001-8721-3882]; Marcel Müller [0000-0002-2264-3643]; Wolfgang Hübner [0000-0002-1128-6468]; Tung-Cheng Wang; Daniel Telman; Thomas Huser [0000-0003-2348-7416]; Wolfram Schenck [0000-0003-3300-2048];

- We have added the ORCIds to the title page along with the author names. Please see the pdf of the manuscript 'Manuscript_GIGA-D-23-00044_Revisions'. The style file (i.e.,"orcidlink.sty") is also attached for your convenience.

3) Please add the GigaDB citation (see below) to the bibliography and cite it by number from the data availability section (I see you already have a "placeholder" where the GigaDB set will be cited).

- We filled all the placeholders with the GigaDB citation number.

4) Please also mention the Zenodo repo (reference [43]) again in the availability section, if it's not redundant with the GigaDb set.

- We added the reference in the zenodo repo (reference [44]) to the availability section.

5) For the code (reference [42]), please add a section "Availability of supporting source code and requirements" at the end.

- We have included a new section called "Availability of supporting source code and requirements" which provides information about the project and its requirements. The computational workflows are also registered in workflowhub.eu and DOIs is cited in this section. Similarly, we received the RRID from the Scicrunch.org database after registering our tool, which is also cited in this section of the manuscript. Regarding bio.tools, unfortunately we were not able to create an account on the bio.tools website (i.e. the link is down). I hope the registrations at scicrunch and workflowhub are sufficient for reproducibility and reuse of your tool.

6) In the data availability section, you write "All the datasets are distributed under the Creative Commons CCO waiver, with a request for attribution. The CCO permits the unrestricted reuse, distribution and

reproduction, provided the original work is properly cited."

- We removed the second sentence to make it simpler and more convenient.

We believe that the necessary points have been addressed, and the manuscript is now ready for publication.
