# Supplementary material for: Evaluation of Swin Transformer and knowledge transfer for denoising of super-resolution structured illumination microscopy data
Source: Gigascience. 2024 Jan 13;13:giad109. doi: 10.1093/gigascience/giad109 (PMC10787368; doi:10.1093/gigascience/giad109)
Supplement: giad109_Reviewer_1_Report_Original_Submission — Xiaoshuai Huang -- 3/29/2023 Reviewed [file giad109_reviewer_1_report_original_submission.pdf]

Reviewer Report

Title: Evaluation of Swin Transformer and knowledge transfer for denoising of super-resolution structured illumination microscopy data

Version: Original Submission **Date:** 3/29/2023

Reviewer name: Xiaoshuai Huang

Reviewer Comments to Author:

Summary:
This manuscript provides 4 fluorescence microscopy datasets with different noise levels to train and evaluate the deep learning (DL) based denoising and super-resolution approaches and investigate the generalization ability of DL denoising networks by direct transfer and fine-tuning. Nevertheless, the reviewer believes that a revision is necessary for the following reasons.
[revision]
(1) To our knowledge, the "BioSR" dataset presented in reference [22] contains quite a few SIM reconstructions. While, the authors declare that "However, their collection of datasets consists of considerably fewer images and does not contain any super-resolution SIM images". The authors may do a detailed survey of the "BioSR" data set.
(2) The authors may modify the bottom "(FFT)" in the "SIM Algorithm" box of Figure 1 as "(Inverse FFT)".
(3) We can see obvious artifacts and background in the reference images (Figure 2, magnified). Can the reference images quality be promoted further?
(4) The UNet-fairSIM output in figure 5b seems to be unreasonable and the authors may try to explain this phenomenon and solve the problem.
(5) Dataset 1-3 in table 1 contain same sample structures and may be combined into one dataset. By the way, the authors may provide other sample structures besides tubulin filaments and vesicles.
(6) The authors can transfer table 2 into the statistical boxplot for an intuitive representation.
(7) There are many deep learning based SIM reconstruction methods like DFCAN/DFGAN, the authors may try these methods besides Red-fairSIM/UNet-fairSIM.
(8) The authors may provide some discussions about "why the fine-tuning output is better than direct transfer?". Does this mean that the first/last few layers of the network are critical to its output?
(9) The authors may provide summary and conclusion about the work.

Methods

Are the methods appropriate to the aims of the study, are they well described, and are necessary controls included? Choose an item.

Conclusions

Are the conclusions adequately supported by the data shown? Choose an item.

Reporting Standards

Does the manuscript adhere to the journal's guidelines on minimum standards of reporting? Choose an item.

Choose an item.

Statistics

Are you able to assess all statistics in the manuscript, including the appropriateness of statistical tests used? Choose an item.

Quality of Written English

Please indicate the quality of language in the manuscript: Choose an item.

Declaration of Competing Interests

Please complete a declaration of competing interests, considering the following questions:

- Have you in the past five years received reimbursements, fees, funding, or salary from an organisation that may in any way gain or lose financially from the publication of this manuscript, either now or in the future?
- Do you hold any stocks or shares in an organisation that may in any way gain or lose financially from the publication of this manuscript, either now or in the future?
- Do you hold or are you currently applying for any patents relating to the content of the manuscript?
- Have you received reimbursements, fees, funding, or salary from an organization that holds or has applied for patents relating to the content of the manuscript?
- Do you have any other financial competing interests?
- Do you have any non-financial competing interests in relation to this paper?

If you can answer no to all of the above, write 'I declare that I have no competing interests' below. If your reply is yes to any, please give details below.

I declare that I have no competing interests

I agree to the open peer review policy of the journal. I understand that my name will be included on my report to the authors and, if the manuscript is accepted for publication, my named report including any attachments I upload will be posted on the website along with the authors' responses. I agree for my report to be made available under an Open Access Creative Commons CC-BY license (http://creativecommons.org/licenses/by/4.0/). I understand that any comments which I do not wish to be included in my named report can be included as confidential comments to the editors, which will not be published.

Choose an item.

To further support our reviewers, we have joined with Publons, where you can gain additional credit to further highlight your hard work (see: https://publons.com/journal/530/gigascience). On publication of this paper, your review will be automatically added to Publons, you can then choose whether or not to claim your Publons credit. I understand this statement.

Yes Choose an item.
