# Supplementary material for: Evaluation of Swin Transformer and knowledge transfer for denoising of super-resolution structured illumination microscopy data
Source: Gigascience. 2024 Jan 13;13:giad109. doi: 10.1093/gigascience/giad109 (PMC10787368; doi:10.1093/gigascience/giad109)
Supplement: giad109_Reviewer_2_Report_Original_Submission — Jun Li -- 3/31/2023 Reviewed [file giad109_reviewer_2_report_original_submission.pdf]

Reviewer Report

Title: Evaluation of Swin Transformer and knowledge transfer for denoising of super-resolution structured illumination microscopy data

Version: Original Submission **Date:** 3/31/2023

Reviewer name: Jun Li

Reviewer Comments to Author:

This paper introduces a contribution to the field of structured illumination microscopy by presenting a large dataset and a comprehensive evaluation of deep-learning based image denoising and super-resolution methods on various biological structures. The dataset is designed to facilitate a better understanding of the performance of these methods in the context of biological imaging.
Strength:
1. The authors have proposed a sizable dataset to facilitate the evaluation of structured illumination microscopy images.
Weakness:
1. The evaluated image denoising/super-resolution methods are considered outdated. It is recommended that the authors incorporate more state-of-the-art methods in the evaluation part, such as Real-ESRGAN and SwinIR.
2. While the authors proposed a large dataset and conducted evaluations for a better understanding of the performance of existing deep-learning based image denoising and super-resolution methods on different biological structures, this paper does not introduce any novel methods. Therefore, the novelty of this paper is limited to the dataset and evaluations themselves.
Detailed Comments
1. In the abstract, on the computer vision side, there is a large number of methods that can blindly reduce the image noise level. Also, for SIM data, there are already methods for denoising multiple structures.
2. On page 2, 'leads to low SNR', the abbreviation needs to be explained.
3. On page 2, the authors compare existing open-source datasets and enumerate the amount of data in them, so it is better to introduce the dataset presented in this paper with the amount as well.
4. In SIM data, the data type is 16bit per pixel, however, the denoising method, i.e. RED was originally designed for 8bit images. In addition, the PSNR calculation in the author's code calls TensorFlow's built-in functions ï¼ˆtf_psnrï¼‰, has the corresponding modification been made for dynamic range, or has the data been pre-processed?
5. The code is publicly available, but the data is not, and it is recommended that some data be made available for testing.
6. How is the distance represented by each pixel (scale bar) derived in the image? For example, 80 nm in Table 1 and 8 Î¼m in Fig.1 and 2. I suggest adding the corresponding explanation.
7. How is noise level defined? In the domain of machine learning, level denotes the standard deviation

of Gaussian noise, which needs to be disambiguated here.

Methods

Are the methods appropriate to the aims of the study, are they well described, and are necessary controls included? Choose an item.

Conclusions

Are the conclusions adequately supported by the data shown? Choose an item.

Reporting Standards

Does the manuscript adhere to the journal's guidelines on minimum standards of reporting? Choose an item.

Choose an item.

Statistics

Are you able to assess all statistics in the manuscript, including the appropriateness of statistical tests used? Choose an item.

Quality of Written English

Please indicate the quality of language in the manuscript: Choose an item.

Declaration of Competing Interests

Please complete a declaration of competing interests, considering the following questions:

- Have you in the past five years received reimbursements, fees, funding, or salary from an organisation that may in any way gain or lose financially from the publication of this manuscript, either now or in the future?
- Do you hold any stocks or shares in an organisation that may in any way gain or lose financially from the publication of this manuscript, either now or in the future?
- Do you hold or are you currently applying for any patents relating to the content of the manuscript?
- Have you received reimbursements, fees, funding, or salary from an organization that holds or has applied for patents relating to the content of the manuscript?
- Do you have any other financial competing interests?
- Do you have any non-financial competing interests in relation to this paper?

If you can answer no to all of the above, write 'I declare that I have no competing interests' below. If your reply is yes to any, please give details below.

I have no competing interests' below.

I agree to the open peer review policy of the journal. I understand that my name will be included on my report to the authors and, if the manuscript is accepted for publication, my named report including any attachments I upload will be posted on the website along with the authors' responses. I agree for my report to be made available under an Open Access Creative Commons CC-BY license (http://creativecommons.org/licenses/by/4.0/). I understand that any comments which I do not wish to be included in my named report can be included as confidential comments to the editors, which will not be published.

Choose an item.

To further support our reviewers, we have joined with Publons, where you can gain additional credit to further highlight your hard work (see: https://publons.com/journal/530/gigascience). On publication of this paper, your review will be automatically added to Publons, you can then choose whether or not to claim your Publons credit. I understand this statement.

Yes Choose an item.
