# Supplementary material for: Evaluation of Swin Transformer and knowledge transfer for denoising of super-resolution structured illumination microscopy data
Source: Gigascience. 2024 Jan 13;13:giad109. doi: 10.1093/gigascience/giad109 (PMC10787368; doi:10.1093/gigascience/giad109)
Supplement: giad109_Reviewer_3_Report_Original_Submission — Yicong Wu -- 4/4/2023 Reviewed [file giad109_reviewer_3_report_original_submission.pdf]

Reviewer Report

Title: Evaluation of Swin Transformer and knowledge transfer for denoising of super-resolution structured illumination microscopy data

Version: Original Submission **Date:** 4/4/2023

Reviewer name: Yicong Wu

Reviewer Comments to Author:

The manuscript attempts to evaluate the generalization capability of a CNN-based denoising deep learning network on 3D SIM images using transfer learning and fine-tuning strategies. However, the study has several weaknesses compared to some cutting-edge denoising methods designed specifically for 3D SIM images. Firstly, datasets 1-3 are based on 2D SIM reconstruction, whereas dataset 4 is based on 3D SIM reconstruction, which leads to different axial resolutions, making it difficult to test the generalization capability of the model. Secondly, the noise level has only four levels, and the sample types are limited to two structures, which is significantly less than the 'BioSR' dataset cited as a reference in the manuscript. Moreover, the authors did not discuss transfer learning and fine-tuning strategies despite their use in the same reference. Denoising and reconstructing 3D SIM images is challenging, and traditional deep learning methods often produce unsatisfactory results, as shown by the low SSIM values in this manuscript. However, recently, more robust methods, such as those that incorporate physical processes (e.g, Li, X., et al. Three-dimensional structured illumination microscopy with enhanced axial resolution. Nat Biotechnol, 2023; Qiao, C., et al. Rationalized deep learning super-resolution microscopy for sustained live imaging of rapid subcellular processes. Nat Biotechnol 41, 367-377, 2023), have been developed to address these problems, and their datasets are publicly available. Additionally, some new methods have been tested to learn from widefield to 3D SIM images, and the reference is missing in this manuscript (e.g, Li, Y., et al. Incorporating the image formation process into deep learning improves network performance. Nat Methods 19, 1427-1437, 2022). Lastly, the authors need to provide a schematic of the fine-tuning strategies they used.

Methods

Are the methods appropriate to the aims of the study, are they well described, and are necessary controls included? Choose an item.

Conclusions

Are the conclusions adequately supported by the data shown? Choose an item.

Reporting Standards

Does the manuscript adhere to the journal’s guidelines on [minimum standards of reporting?]() Choose an item.

Choose an item.

Statistics

Are you able to assess all statistics in the manuscript, including the appropriateness of statistical tests used? Choose an item.

Quality of Written English

Please indicate the quality of language in the manuscript: Choose an item.

Declaration of Competing Interests

Please complete a declaration of competing interests, considering the following questions:

- Have you in the past five years received reimbursements, fees, funding, or salary from an organisation that may in any way gain or lose financially from the publication of this manuscript, either now or in the future?
- Do you hold any stocks or shares in an organisation that may in any way gain or lose financially from the publication of this manuscript, either now or in the future?
- Do you hold or are you currently applying for any patents relating to the content of the manuscript?
- Have you received reimbursements, fees, funding, or salary from an organization that holds or has applied for patents relating to the content of the manuscript?
- Do you have any other financial competing interests?
- Do you have any non-financial competing interests in relation to this paper?

If you can answer no to all of the above, write 'I declare that I have no competing interests' below. If your reply is yes to any, please give details below.

'I declare that I have no competing interests'

I agree to the open peer review policy of the journal. I understand that my name will be included on my report to the authors and, if the manuscript is accepted for publication, my named report including any attachments I upload will be posted on the website along with the authors' responses. I agree for my report to be made available under an Open Access Creative Commons CC-BY license (http://creativecommons.org/licenses/by/4.0/). I understand that any comments which I do not wish to be included in my named report can be included as confidential comments to the editors, which will not be published.

Choose an item.

To further support our reviewers, we have joined with Publons, where you can gain additional credit to further highlight your hard work (see: https://publons.com/journal/530/gigascience). On publication of this paper, your review will be automatically added to Publons, you can then choose whether or not to claim your Publons credit. I understand this statement.

Yes Choose an item.
