# Supplementary material for: Evaluation of Swin Transformer and knowledge transfer for denoising of super-resolution structured illumination microscopy data
Source: Gigascience. 2024 Jan 13;13:giad109. doi: 10.1093/gigascience/giad109 (PMC10787368; doi:10.1093/gigascience/giad109)
Supplement: giad109_Reviewer_3_Report_Revision_1 — Yicong Wu -- 7/21/2023 Reviewed [file giad109_reviewer_3_report_revision_1.pdf]

**Reviewer Report**

**Title: Evaluation of Swin Transformer and knowledge transfer for denoising of super-resolution structured illumination microscopy data**

**Version: Revision 1** **Date:** 7/21/2023

**Reviewer name: Yicong Wu**

**Reviewer Comments to Author:**

In the revised version, the manuscript's quality has seen notable improvements, particularly due to the incorporation of results obtained using the Transformer-based SR-SIM image denoising algorithm. However, it remains concerning that the authors have not adequately addressed my comments, resulting in persisting weaknesses within the manuscript. One significant concern is that, regardless of whether 9 or 15 raw SIM images are employed in 2D reconstruction, the axial resolution consistently falls short compared to that achieved in 3D reconstruction. Consequently, the statement asserting that "this rules out the different axial resolution difference" is entirely inaccurate. Due to these unresolved issues, I am unable to ascertain the model's generalization capability across the four datasets. Moreover, the 'BioSR' dataset and other publicly available data showcasing new deep learning techniques for SIM denoising and reconstruction seem to overshadow the significance of the four datasets presented in this manuscript. Given the low image quality demonstrated within the manuscript, my confidence in the datasets' usefulness for the benefit of the scientific community has significantly diminished.

**Methods**

Are the methods appropriate to the aims of the study, are they well described, and are necessary controls included? Choose an item.

**Conclusions**

Are the conclusions adequately supported by the data shown? Choose an item.

**Reporting Standards**

Does the manuscript adhere to the journal's guidelines on minimum standards of reporting? Choose an item.

Choose an item.

**Statistics**

Are you able to assess all statistics in the manuscript, including the appropriateness of statistical tests used? Choose an item.

**Quality of Written English**

Please indicate the quality of language in the manuscript: Choose an item.

**Declaration of Competing Interests**

Please complete a declaration of competing interests, considering the following questions:

- Have you in the past five years received reimbursements, fees, funding, or salary from an organisation that may in any way gain or lose financially from the publication of this manuscript, either now or in the future?
- Do you hold any stocks or shares in an organisation that may in any way gain or lose financially from the publication of this manuscript, either now or in the future?
- Do you hold or are you currently applying for any patents relating to the content of the manuscript?
- Have you received reimbursements, fees, funding, or salary from an organization that holds or has applied for patents relating to the content of the manuscript?
- Do you have any other financial competing interests?
- Do you have any non-financial competing interests in relation to this paper?

If you can answer no to all of the above, write 'I declare that I have no competing interests' below. If your reply is yes to any, please give details below.

I declare that I have no competing interests.

I agree to the open peer review policy of the journal. I understand that my name will be included on my report to the authors and, if the manuscript is accepted for publication, my named report including any attachments I upload will be posted on the website along with the authors' responses. I agree for my report to be made available under an Open Access Creative Commons CC-BY license (http://creativecommons.org/licenses/by/4.0/). I understand that any comments which I do not wish to be included in my named report can be included as confidential comments to the editors, which will not be published.

Choose an item.

To further support our reviewers, we have joined with Publons, where you can gain additional credit to further highlight your hard work (see: https://publons.com/journal/530/gigascience). On publication of this paper, your review will be automatically added to Publons, you can then choose whether or not to claim your Publons credit. I understand this statement.

Yes Choose an item.
